# Ferric carboxymaltose versus standard-of-care oral iron to treat second-trimester anaemia in Malawian pregnant women: a randomised controlled trial

**DOI:** 10.1016/S0140-6736(23)00278-7

**Published:** 2023-05-13

**Authors:** Sant-Rayn Pasricha, Martin N Mwangi, Ernest Moya, Ricardo Ataide, Glory Mzembe, Rebecca Harding, Truwah Zinenani, Leila M Larson, Ayse Y Demir, William Nkhono, Jobiba Chinkhumba, Julie A Simpson, Danielle Clucas, William Stones, Sabine Braat, Kamija S Phiri

**Affiliations:** a Population Health and Immunity Division, Walter and Eliza Hall Institute of Medical Research, Parkville, VIC, Australia; b Diagnostic Haematology, The Royal Melbourne Hospital, Parkville, VIC, Australia; c Clinical Haematology, The Peter MacCallum Cancer Centre and The Royal Melbourne Hospital, Parkville, VIC, Australia; d Department of Medical Biology, University of Melbourne, Parkville, VIC, Australia; e Department of Medicine at the Peter Doherty Institute, University of Melbourne, Parkville, VIC, Australia; f Centre for Epidemiology and Biostatistics, Melbourne School of Population and Global Health, University of Melbourne, Parkville, VIC, Australia; g Training and Research Unit of Excellence, Blantyre, Malawi; h Department of Public Health, School of Public and Global Health, Kamuzu University of Health Sciences, Blantyre, Malawi; i The Micronutrient Forum, Healthy Mothers Healthy Babies Consortium, Washington, DC, USA; j Department of Health Promotion, Education and Behavior, Arnold School of Public Health, University of South Carolina, Columbia, SC, USA; k Laboratory for Clinical Chemistry and Haematology, Meander Medical Centre, Amersfoort, Netherlands

## Abstract

**Background:**

Anaemia affects 46% of pregnancies in Africa; oral iron is recommended by WHO but uptake and adherence are suboptimal. We tested a single dose of a modern intravenous iron formulation, ferric carboxymaltose, for anaemia treatment in Malawian pregnant women.

**Methods:**

In this open-label, individually randomised controlled trial, we enrolled women with a singleton pregnancy of 13–26 weeks' gestation in primary care and outpatient settings across two regions in southern Malawi. Women were eligible if they had capillary haemoglobin of less than 10·0 g/dL and negative malaria rapid diagnostic test. Participants were randomised by sealed envelope 1:1. Assessors for efficacy outcomes (laboratory parameters and birthweight) were masked to intervention; participants and study nurses were not masked. Participants were given ferric carboxymaltose up to 1000 mg (given once at enrolment in an outpatient primary care setting), or standard of care (60 mg elemental iron twice daily for 90 days), along with intermittent preventive malaria treatment. The primary maternal outcome was anaemia at 36 weeks' gestation. The primary neonatal outcome was birthweight. Analyses were performed in the intention-to-treat population for mothers and liveborn neonates, according to their randomisation group. Safety outcomes included incidence of adverse events during infusion and all adverse events from randomisation to 4 weeks' post partum. The trial is registered with ANZCTR, ACTRN12618001268235. The trial has completed follow-up.

**Findings:**

Between Nov 12, 2018, and March 2, 2021, 21 258 women were screened, and 862 randomly assigned to ferric carboxymaltose (n=430) or standard of care (n=432). Ferric carboxymaltose did not reduce anaemia prevalence at 36 weeks' gestation compared with standard of care (179 [52%] of 341 in the ferric carboxymaltose group *vs* 189 [57%] of 333 in the standard of care group; prevalence ratio [PR] 0·92, 95% CI 0·81 to 1·06; p=0·27). Anaemia prevalence was numerically lower in mothers randomly assigned to ferric carboxymaltose compared with standard of care at all timepoints, although significance was only observed at 4 weeks' post-treatment (PR 0·91 [0·85 to 0·97]). Birthweight did not differ between groups (mean difference –3·1 g [–75·0 to 68·9, p=0·93). There were no infusion-related serious adverse events or differences in adverse events by any organ class (including malaria; ≥1 adverse event: ferric carboxymaltose 183 [43%] of 430 *vs* standard of care 170 [39%] of 432; risk ratio 1·08 [0·92 to 1·27]; p=0·34).

**Interpretation:**

In this malaria-endemic sub-Saharan African setting, treatment of anaemic pregnant women with ferric carboxymaltose was safe but did not reduce anaemia prevalence at 36 weeks' gestation or increase birthweight.

**Funding:**

Bill & Melinda Gates Foundation (INV-010612).

## Introduction

Anaemia affects 41% of pregnancies worldwide,^1^ including 46% of pregnancies in Africa.^2^ Antenatal anaemia is associated with risks for both the mother (eg, post-partum haemorrhage and mortality)^3^, ^4^ and their baby (eg, low birthweight and prematurity).^4^ Reducing anaemia and low birthweight are global nutrition targets.^5^ WHO has estimated that approximately 50% of anaemia in pregnancy (44% in sub-Saharan Africa) is iron responsive.^6^ WHO recommends that all pregnant women receive daily oral iron supplementation, along with folic acid.^7^ However, adherence to antenatal oral iron is often suboptimal. For example, only 28·7% of pregnant women in sub-Saharan Africa consume the recommended dose.^8^ Infection (especially with *Plasmodium falciparum*) also contributes to complex determinants of antenatal anaemia in sub-Saharan Africa.^9^

Ferric carboxymaltose is a new-generation intravenous iron formulation that enables up to 1000 mg (20 mg/kg) to be safely administered in a single 15-min infusion with minimal risk of serious allergic reactions.^10^, ^11^ Ferric carboxymaltose is widely used to treat iron deficiency in high-income settings (including during pregnancy)^12^ and is increasingly used in primary care.^13^

Malawi, in southern Africa, is a low-income country where pregnant women endure numerous challenges. Approximately 45% of pregnant women in Malawi are anaemic, 10·8% of Malawian women are HIV positive,^14^ and 15–20% of Southern Malawian women have *Plasmodium* parasitemia.^15^ First antenatal visits generally occur late (between 17–22 weeks).^14^ If available, workup for anaemia is generally limited to point-of-care haemoglobin measurement and rapid diagnostic tests (RDTs) or blood film examination for plasmodium infection; testing for iron biomarkers such as ferritin is not accessible in primary care. Women face a high risk of pregnancy and perinatal complications including post-partum haemorrhage, stillbirth, premature delivery, and low birthweight.^14^, ^16^ Unless there is a contraindication, pregnant women are offered intermittent preventive therapy for malaria, usually with sulfadoxine-pyrimethamine.^14^

**Research in context**

**Evidence before this study**

A search of PubMed for “intravenous iron” AND “pregnancy” OR “antenatal” identified nine systematic reviews, which contained limited trial data available from low-income countries or modern intravenous formulations such as ferric carboxymaltose that enable high-dose iron replacement. The search was from database inception to August, 2022. There were no language restrictions. For example, a systematic review and meta-analysis of 15 trials comparing intravenous iron with oral iron in pregnancy identified advantages from intravenous iron on haematology (intravenous iron therapy led to higher maternal haemoglobin at delivery; mean difference 7·4 g/L [95% CI 3·9–11]; nine RCTs, low-quality evidence) and iron status (ferritin: mean difference 21·2 μg/L [95% CI 6·5–36·0]; three randomised controlled trials [RCTs], low-quality evidence); and low-quality evidence of the benefits on birthweight and need for blood transfusion. A network meta-analysis compared different formulations of iron in pregnancy and identified advantages from intravenous iron over oral formulations of iron in increasing haemoglobin concentration 4 weeks' post-administration (but did not evaluate this outcome before delivery). Older reviews compiled evidence indicating iron sucrose (which can only be given in low doses) provides superior haematologic and iron status outcomes compared with oral iron. We also searched all trials using ferric carboxymaltose in resource-limited settings. Only one trial assessed the use of ferric carboxymaltose in low-income settings: a study in Tanzania recruited 230 randomly assigned post-partum women to oral iron or ferric carboxymaltose and showed that women receiving the intravenous formulation had higher haemoglobin concentrations over a 12-month period. Two RCTs comparing ferric carboxymaltose with oral iron in pregnancy have been conducted: the pivotal FER-ASAP trial recruited 232 pregnant women with iron deficiency anaemia in high-income settings, and showed early superiority from ferric carboxymaltose over high-dose oral ferrous sulphate on haemoglobin concentrations, which was not sustained beyond the first 6 weeks; a smaller trial compared ferric carboxymaltose with oral iron (and intravenous iron polymaltose) in 246 pregnant women and likewise showed more rapid increases in haemoglobin and ferritin in the intravenous groups compared with oral iron. A large trial explored the use of an older intravenous iron formulation (iron sucrose) for iron deficiency anaemia in pregnancy in India: iron sucrose is limited by the low dose that can be delivered in a single infusion, necessitating recurrent visits.

**Added value of this study**

To our knowledge, the randomised trial of intravenous iron for anaemia in Malawian pregnant women (REVAMP) is the first trial to compare a modern intravenous iron formulation capable of rapidly delivering a high dose of iron over a short infusion timeframe to standard of care oral iron to treat antenatal anaemia in a low-income setting, and is also the largest trial to date of ferric carboxymaltose in pregnancy. The trial inclusion criteria were simple and designed to enable translation of results to practice in resource-limited settings. REVAMP recruited a sample size almost times the size of the pivotal FER-ASAP trial. REVAMP shows the feasibility and safety of ferric carboxymaltose in a low-income, primary-care setting where infectious exposures are intense. Our data indicate that compared with oral iron, ferric carboxymaltose markedly reduces iron deficiency anaemia for the duration of pregnancy and into the post-partum, and provides a more rapid elevation in haemoglobin concentration (over oral iron); although this advantage might not be sustained by delivery. Moreover, our data highlight the complex determinants of anaemia in this setting.

**Implications of all the available evidence**

Compared with oral iron, ferric carboxymaltose produced a profound reduction in iron deficiency and iron deficiency anaemia, and a more rapid early haematologic response. However, there was no reduction in anaemia prevalence before delivery among the overall cohort; nor did we identify functional benefits from intravenous iron, even though the study population had a high burden of maternal and neonatal pregnancy complications (eg, low birthweight and post-partum haemorrhage). Although the trial was negative for the primary outcome, REVAMP provides new information on urgent technological needs (eg, field-friendly iron status screening), and potential future important contexts for this intervention (women in the third trimester of pregnancy, settings without malaria).

Ferric carboxymaltose could present an option to treat anaemia in pregnancy in low-income settings, but the efficacy and safety of this approach in these settings are unproven. We therefore conducted a randomised controlled trial to determine whether a single dose of intravenous ferric carboxymaltose would be superior to standard-of-care (oral iron) in reducing anaemia prevalence at 36 weeks' gestation (before delivery), improve other maternal and neonatal outcomes (including birthweight), and be safe for Malawian women in their second trimester of pregnancy with moderate or severe anaemia (by capillary haemoglobin measurement) and without detectable *P falciparum* parasitemia (by RDT).

## Methods

### Study design

The randomised trial of intravenous iron for anaemia in Malawian pregnant women (REVAMP) was an open-label, parallel, individually randomised controlled trial run in primary care and outpatient settings across two centres in southern Malawi. The protocol and statistical analysis plan are included in the appendix (pp 11–220) and were published before database lock.^17^, ^18^ The trial was approved by ethics committees at the College of Medicine, University of Malawi, Zomba, Malawi; and The Walter and Eliza Hall Institute of Medical Research, Melbourne, VIC, Australia. All authors attest to the completeness and accuracy of the data and analyses and the adherence of the trial to the protocol. No support from a commercial entity (including the manufacturer of ferric carboxymaltose) was provided.

### Participants

Participants were eligible for the trial if they had a capillary haemoglobin concentration below 10·0 g/dL (moderate or severe anaemia) measured by HemoCue 301+ (HemoCue AB, Angelholm, Sweden), were *P falciparum* parasitemia negative (RDT), had a confirmed singleton pregnancy of 13–26 weeks' gestation, had no diagnosed inherited red cell disorder, and were not clinically judged to require transfusion or have another acute medical illness. Exlcusion criteria were *P falciparum* parasitemia positive (RDT), a diagnosed inherited red cell disorder, judged to require transfusion (ie, haemoglobin <5 g/dL), or another acute medical illness. We used capillary haemoglobin measurement as we judged that widespread screening with venous blood for measurement of haemoglobin and iron indices is not presently feasible in resource-limited primary-care settings and, if used, would limit the generalisability of results. HIV positivity was not an exclusion.

Women were screened for anaemia and parasitemia at antenatal clinics in health centres in Blantyre and Zomba; participants were usually screened during their first antenatal visit. If women met anaemia criteria but had a positive RDT, they were treated for malaria as per national guidelines (usually with artemether-lumefantrine)^19^ and deferred. These women could present for re-screening after at least 7 days and be enrolled if they met the eligibility criteria (with parasitemia assessed using microscopy due to persistence of RDT antigen detection). Potentially eligible women were referred to central sites in Blantyre and Zomba for fetal ultrasound to confirm singleton pregnancy and gestational age through measurement of biparietal diameter or femoral length.^20^ Oral or written informed consent was obtained from all women before screening, and written consent was obtained before being randomly assigned.

### Randomisation and masking

Using a sealed envelope containing an allocation code that was computer-generated by an independent statistician not otherwise involved in the trial, women were randomly assigned in a 1:1 ratio with random permuted blocks of size 4 or 6, stratified by site to receive standard of care or ferric carboxymaltose. The trial was not blinded as it was judged unfeasible to provide placebo infusions in this setting. However, laboratory and delivery room staff (who collected efficacy outcomes) were masked to allocation.

### Procedures

Women assigned to the intervention group received a single dose of ferric carboxymaltose (Vifor Pharma, purchased commercially) at 20 mg/kg (up to 1000 mg) diluted in 250 mL normal saline, administered intravenously over 15 mins, at the enrolment visit. Ferric carboxymaltose was infused by study nurses according to a standard operating procedure (appendix pp 2–10) in treatment rooms equipped to manage allergic reactions—ie, with medications, fluids, airway equipment, and oxygen. Women in the standard of care group were provided with oral iron (60 mg elemental iron as ferrous sulphate, twice daily for 90 days) accompanied by a similar educational message to what local providers would give. All participants received intermittent preventive treatment with sulfadoxine-pyrimethamine (IPTp-SP) at enrolment, 28 days later, and at 36 weeks' gestation unless contraindicated (eg, recent malaria therapy or HIV positive on cotrimoxazole).

Participants were followed up at scheduled visits at 28 days' post-enrolment, 36 weeks' gestation, delivery, and 4 weeks' post partum. Home visits were also scheduled fortnightly at 34, 38, and 40 weeks' gestation to collect capillary haemoglobin and count residual oral iron tablets (control group). During the trial, the study offered a 7-day-a-week service for participants, in which mothers could present for diagnosis and treatment of any symptoms. Women could attend the study centres for unscheduled visits if they became unwell. At each scheduled visit, women were also asked if they (or their neonate) had attended other health providers. This strategy enabled comprehensive capture of adverse events.

### Outcomes

The primary maternal efficacy outcome was anaemia (venous haemoglobin concentration <11·0 g/dL measured on a Sysmex XP-300 automated analyser [Sysmex, Japan]) at 36 weeks' gestation. The primary maternal outcome was changed during the trial because of guidance introduced by the local ethics committee due to the COVID-19 pandemic.^21^ The initial primary outcome was anaemia at the timepoint before delivery, measured during home visits at 34, 38, or 40 weeks' gestation or the 36-week visit on a capillary sample with a HemoCue 301+ device. Adherence to oral iron in the standard of care group was also to be monitored at the 34-week home visit. However, in May, 2020, home visits were cancelled to reduce the risk of study staff spreading SARS-CoV-2 in participants' villages. No unblinded data were seen before these changes. The trial protocol and registration were amended at the time of the change and reflected in the statistical analysis plan.

Secondary maternal outcomes included anaemia at 4 weeks' post-infusion and at delivery (venous haemoglobin <11·0 g/dL), and at 4 weeks' post partum (venous haemoglobin <12·0 g/dL). The prevalence of moderate–severe anaemia and change in haemoglobin concentration from baseline were also compared between groups at each timepoint. Iron status (iron deficiency defined as ferritin <15 μg/L or <30 μg/L if C-reactive protein >5 mg/L^22^, ^23^) and iron deficiency anaemia were also compared at each timepoint. The primary neonatal outcome was birthweight; secondary neonatal outcomes included incidence of low birthweight (birthweight <2500 g), small for gestational age^24^ and premature delivery, gestation duration, birth length, and, at 4 weeks of age, venous haemoglobin concentration (measured by Sysmex), weight, length, and corresponding Z scores.^25^

Safety outcomes included adverse events during ferric carboxymaltose infusion (assessed according to a checklist for women in the active group) and adverse events for any reason from randomisation to 4 weeks' post partum, including serious adverse events (eg, unplanned hospitalisation or death including pregnancy loss and neonatal death), clinical infection episodes, biochemical hypophosphataemia, and positive RDTs. We defined a composite severe maternal event as maternal death, intensive care admission, post-partum haemorrhage, or need for a blood transfusion. We reported abortion, stillbirth, and neonatal death individually and as a combined outcome.

The trial provided 24 h midwifery service in the delivery ward, which supported the care of trial participants (and other mothers attending the hospital), enabling measurement of neonatal outcomes.^18^

Primary and secondary efficacy outcomes and safety outcomes presented here include those listed in the prespecified statistical analysis plan in support of the primary trial results that were published before the database lock and unblinding. Other outcomes included in the protocol but not included in the statistical analysis plan include outcomes that are explorative or related to substudies.

### Statistical analysis

Sample size is based on the maternal primary outcome of proportion of women with pre-delivery anaemia (venous haemoglobin <11·0 g/dL). We estimated routine iron supplementation would reduce anaemia from 100% to 60%, on the basis of reductions in anaemia seen in cohorts of pregnant women treated with iron^26^ and systematic reviews.^4^ The Fer-ASAP trial showed a 14% reduction in absolute anaemia prevalence compared with oral iron.^27^ The trial was planned to detect a minimally clinically important difference in anaemia prevalence of 10% (50% *vs* 60%) between ferric carboxymaltose and standard of care, with 80% power. Using a two-sided alpha of 5% and 10% loss to follow up, a sample size of 862 participants (431 per group) was needed on the basis of a χ^2^ test. This sample size also provided 80% power to detect a 100 g absolute difference in birthweight between ferric carboxymaltose and standard of care, assuming a SD of 450 g and a two-sided alpha of 5%.^28^

Analysis followed a pre-specified statistical analysis plan.^17^ Maternal and neonatal efficacy outcomes were analysed according to the randomly allocated group of the woman and included all available data, targeting the intention-to-treat principle.

Maternal anaemia (primary outcome) was analysed using a mixed effects Poisson regression model. The model included fixed effects of treatment, study visit, and the treatment by study visit interaction, with a random intercept for the women and robust standard error. Similar analyses were applied to repeatedly measured dichotomous secondary maternal outcomes. Secondary maternal outcomes of Hb and log-transformed ferritin were analysed using a likelihood-based longitudinal data analysis model by Liang and Zeger,^29^ assuming a common baseline mean across the two groups, and an unstructured variance–covariance among the repeated measurements. Birthweight (primary neonatal outcome) was analysed with a linear regression model. The same analysis was used for continuous neonatal secondary outcomes. Binary secondary neonatal outcomes were analysed with a log-binomial regression model. Maternal and neonatal safety outcomes were analysed according to the treated group of the woman and included all available data. Adverse events were compared between treatment groups using a log-binomial regression model. Hypophosphataemia and inflammation were analysed using the same analyses as the primary maternal outcome. All analyses were adjusted for site (the stratification factor) as a covariate.

Results are presented as point estimates and two-sided 95% CIs. No multiplicity adjustment is applied to CIs, and these cannot be used in place of hypothesis testing. A two-sided p value <0·05 was used to indicate significance for the primary maternal outcome and the primary neonatal outcome. The Holm procedure was used to control family-wise error rate at 0·05 for the key secondary maternal outcomes at 36 weeks' gestation (haemoglobin concentration, moderate–severe anaemia, ferritin concentration, iron deficiency, and iron deficiency anaemia) and neonatal outcomes (gestation duration, birth length, composite adverse birth outcome at delivery, and infant growth at 4 weeks' post partum).^30^ No p values are presented for other maternal and neonatal secondary outcomes. Additional analyses of primary, key secondary, and other secondary outcomes included adjusted analyses for pre-specified covariates and a per-protocol analysis of participants without multiple pregnancy. The model for the primary maternal outcome was fitted under the missing-at-random assumption. An analysis based on a pattern-mixture model was conducted to assess sensitivity to outcome data missing not at random. The model for the primary neonatal outcome of birthweight was fitted under the missing-completely-at-random assumption. As the proportion of liveborn neonates missing birthweight data was considered negligible, no sensitivity analysis for missing data was conducted.^31^ Eight predefined subgroup analyses were performed on baseline characteristics: parity, HIV status, severe anaemia, iron deficiency, iron deficiency anaemia, inflammation, re-screened post-positive RDT, and site. Predefined subgroup analyses were performed for two maternal outcomes (anaemia, haemoglobin concentration) and four neonatal outcomes (birthweight, low birthweight, gestation duration, and premature birth). Analyses were performed using Stata SE, version 17.0. The trial was overseen by an independent data safety monitoring committee, and prospectively registered (ACTRN12618001268235).

### Role of the funding source

The funder of the study had no role in study design, data collection, data analysis, data interpretation, or writing of the report.

## Results

**Figure 1:**
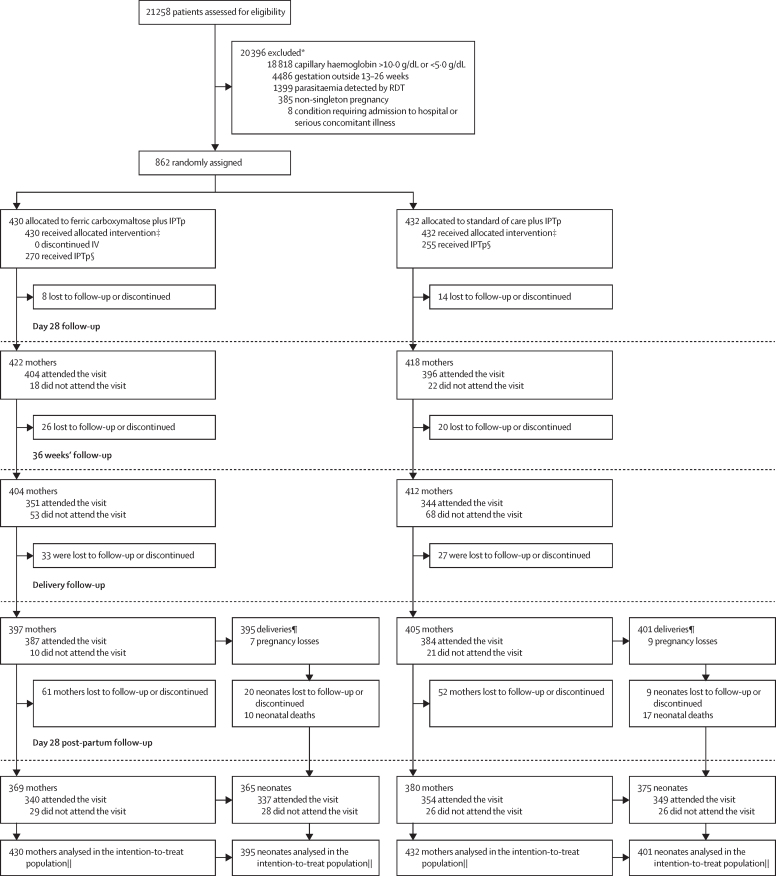
Trial profile IPTp=intermittent preventative therapy. RDT=rapid diagnostic test. *Reasons for not meeting eligibility were assessed on the questions on the eligibility data collection forms; woman can be ineligible on more than one of the eligibility criteria. †Defined as those who answered no to the question “Accepts study procedures?” at enrolment. ‡Reasons for not receiving the treatment were collected on the participant randomisation form. §Defined as those who received IPTp with suldadoxine pyrimethamine at enrolment. ¶There were four twins born in the intravenous iron group and four in the standard of care oral iron group. ||The intention-to-treat basis indicates maternal and neonatal outcomes were analysed according to randomly allocated group of the woman, and included all available data. Home visits collecting capillary haemoglobin and adherence discontinued due to COVID-19 restrictions.

Between Nov 12, 2018, and March 2, 2021, 21 258 women were screened, and 862 (4%) were randomly assigned to a study group (430 to ferric carboxymaltose group and 432 to standard of care group; figure 1). The last 4-week post-partum visit was completed on Sept 13, 2021. 18 818 (89%) of 21 258 screened participants did not pass anaemia eligibility. Among 2440 potentially eligible women with haemoglobin of less than 10·0 g/dL, 1339 (57%) were initially ineligible as they had a positive RDT. All participants in both groups received their randomly allocated treatment. Adherence to standard of care (measured by counting leftover iron tablets from 180 distribed) was compromised by cessation of home visits during the trial, and could be assessed in only 223 women; among 146 women who permitted fieldworkers to count tablets (77 refused), the median number counted was 30 (range 0–174).

Baseline characteristics, including prevalences of moderate (649 [76%] of 858) and severe (62 [7%] of 858) anaemia by venous testing, and prevalence of iron deficiency (366 [43%] of 844), inflammation (432 [51%] of 844), and HIV positivity (145 [17%] of 855) were similar between groups (table 1, appendix pp 221–22). 61% of women had documented receipt of IPTp-SP at baseline, with the remainder generally having a contraindication (usually recent therapeutic antimalarial treatment). Anaemia prevalence data were available from 674 (78%) women at 36 weeks' gestation; a further 42 (5%) women had already delivered by this timepoint. There was no significant reduction in anaemia prevalence at 36 weeks' gestation for women randomly assigned to ferric carboxymaltose compared with the standard of care group (ferric carboxymaltose: 179 [52%] of 341 *vs* standard of care: 189 [57%] of 333, prevalence ratio [PR] 0·92 [95% CI 0·81–1·06], p=0·27). Anaemia prevalence was numerically lower in mothers randomly assigned to ferric carboxymaltose compared with standard of care at all timepoints, although significance was only observed at 4-weeks' post-treatment, with effect sizes as follows: 4-weeks' post-treatment (ferric carboxymaltose: 306 [77%] of 399 *vs* standard of care: 329 [84%] of 390, PR 0·91 [95% CI 0·85–0·97]), delivery (99 [27%] of 362 *vs* 111 [31%] of 356, 0·88 [0·70–1·10]) and 4-weeks' post partum (149 [46%] of 321 *vs* 171 [54%] of 317, 0·86 [0·74–1·01]). Change from baseline haemoglobin concentrations appeared higher in women randomised to ferric carboxymaltose compared with standard of care, including at 4-weeks' post-treatment (1·32 g/dL *vs* 1·11 g/dL, mean difference 0·19 [95% CI 0·06–0·33]) and at 4-weeks' post partum (3·15 g/dL *vs* 2·85 g/dL, mean difference 0·25 [0·06–0·45]; table 2).

**Table 1:** Baseline characteristics of the participating pregnant women

		**Ferric carboxymaltose (n=430)**	**Standard of care (n=432)**
Age, years		22·2 (6·0)	22·5 (6·3)
Primiparous		232 (54%)	240 (56%)
Primigravid		230 (53%)	236 (55%)
Gestational age, weeks			
	Median	21·9	21·8
	IQR	19·6–24·1	18·9–24·1
Height, cm		155·4 (5·8)	155·2 (6·9)
Weight, kg		55·8 (8·0)	55·6 (8·6)
BMI, kg/m^2^		23·1 (2·9)	23·0 (3·2)
Religion			
	None	1/429 (<1%)	1/430 (<1%)
	Christian	293/429 (68%)	324/430 (75%)
	Muslim	132/429 (31%)	99/430 (23%)
	Other	3/429 (<1%)	6/430 (1%)
Education			
	None	1/417 (<1%)	1/413 (<1%)
	Lower primary	87/417 (21%)	88/413 (21%)
	Upper primary	172/417 (41%)	168/413 (41%)
	Lower secondary	56/417 (13%)	59/413 (14%)
	Upper secondary	91/417 (22%)	87/413 (21%)
	Tertiary	10/417 (2%)	10/413 (2%)
Marital status			
	Single	65/429 (15%)	69/430 (16%)
	Married	359/429 (84%)	350/430 (81%)
	Widowed	1/429 (<1%)	3/430 (<1%)
	Divorced or separated	4/429 (1%)	6/430 (1%)
	Other	0/429	2/430 (<1%)
Income source			
	None	22/429 (5%)	31/430 (7%)
	Subsistence farming	79/429 (18%)	72/430 (17%)
	Large scale farming	1/429 (<1%)	1/430 (<1%)
	Employed	85/429 (20%)	68/430 (16%)
	Casual work for wages	135/429 (31%)	129/430 (30%)
	Business	101/429 (24%)	123/430 (29%)
	Other	6/429 (1%)	6/430 (1%)
Re-screened post positive malaria RDT*		130/430 (30%)	124/432 (29%)
Malaria RDT positive†		6/420 (1%)	6/426 (1%)
HIV positive		71/427 (17%)	74/428 (17%)
Capillary Hb, <10 g/dL		429/429 (100%)	431/431 (100%)
Venous Hb, g/dL‡		8·83 (1·28)	8·83 (1·23)
Anaemia (based on venous Hb)			
	No (≥11 g/dL)	24/426 (6%)	18/432 (4%)
	Mild (10–<11 g/dL)	49/426 (12%)	56/432 (13%)
	Moderate (7–<10 g/dL)	322/426 (76%)	327/432 (76%)
	Severe (<7 g/dL)	31/426 (7%)	31/432 (7%)
Ferritin, μg/L§			
	Median	26·20	27·50
	Interquartile range	9·80–80·70	10·20–63·40
C-reactive protein, mg/L§			
	Median	5·20	5·20
	IQR	2·60–10·60	3·00–10·60
Iron deficient¶		180/419 (43%)	186/425 (44%)
Iron deficiency anaemia¶		169/415 (41%)	180/425 (42%)
Inflammation‖		214/419 (51%)	218/425 (51%)
Anaemia and inflammation‖		203/415 (49%)	208/425 (49%)

Participants were enrolled across two sites, Blantyre (n=139) and Zomba (n=723). Data are n (%), n/N (%), or mean (SD). Religion, education, marital status, income source, parity, gravidity, and HIV status were self-reported. RDT=rapid diagnostic test.

* If women met the anaemia criteria but had a positive RDT, they were treated for malaria as per local protocols and deferred from enrolment. These women were able to present for re-screening—no earlier than 7 days later—and be enrolled if they met the eligibility criteria (in these cases, parasitaemia was assessed using microscopy due to persistence of antigen detection via RDT).

† Malaria RDT positive was based on confirmatory RDT testing by laboratory personal on venous blood collected at enrolment.

‡ Data are missing for four participants in the ferric carboxymaltose group.

§ Data are missing for 11 participants in the ferric carboxymaltose group and seven participants in the standard of care group.

¶ Iron deficient indicates serum ferritin <15 μg/L or ferritin <30 μg/L if C-reactive protein >5 mg/L, and iron deficiency anaemia indicates Hb <11 g/dL and serum ferritin <15 μg/L or ferritin <30 μg/L if C-reactive protein >5 mg/L.

‖ Inflammation indicates C-reactive protein >5 mg/L, and anaemia and inflammation indicates Hb <11·0 g/dL and C-reactive protein >5 mg/L.

**Table 2:** Effects of ferric carboxymaltose on maternal efficacy outcomes*

		**Ferric carboxymaltose (n=430)**	**Standard of care (n=432)**	**Prevalence ratio (95% CI)**	**Mean difference (95% CI)**	**Geometric mean ratio (95% CI)**	**p value**
**Primary outcome**							
Anaemia at 36 weeks' gestation		179/341 (52%)	189/333 (57%)	0·92 (0·81 to 1·06)	..	..	0·27
**Key secondary outcomes**							
Moderate–severe anaemia at 36 weeks' gestation		67/341 (20%)	82/333 (25%)	0·81 (0·61 to 1·07)	..	..	0·14
Hb change from baseline at 36 weeks' gestation, g/dL		2·02 (1·41)	1·85 (1·49)	..	0·15 (−0·02 to 0·33)	..	0·077
Median ferritin change from baseline at 36 weeks' gestation (IQR), μg/L		59·20 (28·20–125·60)	22·30 (14·20–35·10)	..	..	2·55 (2·28 to 2·86)	<0·0001*
Iron deficient at 36 weeks' gestation		60/336 (18%)	142/341 (42%)	0·4 (0·33 to 0·55)	..	..	<0·0001*
Iron deficient anaemia at 36 weeks' gestation		29/324 (9%)	93/321 (29%)	0·30 (0·20 to 0·44)	..	..	<0·0001*
**Other secondary outcomes**							
Anaemia							
	4-weeks' post-treatment	306/399 (77%)	329/390 (84%)	0·91 (0·85 to 0·97)	..	..	NA
	Delivery	99/362 (27%)	111/356 (31%)	0·88 (0·70 to 1·10)	..	..	NA
	4-weeks' post-partum	149/321 (46%)	171/317 (54%)	0·86 (0·74 to 1·01)	..	..	NA
Moderate–severe anaemia							
	4-weeks' post-treatment	168/399 (42%)	184/390 (47%)	0·89 (0·76 to 1·04)	..	..	NA
	Delivery	42/362 (12%)	46/356 (13%)	0·89 (0·60 to 1·32)	..	..	NA
	4-weeks' post-partum	66/321 (21%)	70/317 (22%)	0·91 (0·68 to 1·23)	..	..	NA
Hb change from baseline—g/dL							
	4-weeks' post-treatment	1·32 (1·16)	1·11 (1·12)	..	0·19 (0·06 to 0·33)	..	NA
	Delivery	2·96 (1·71)	2·78 (1·85)	..	0·19 (−0·04 to 0·41)	..	NA
	4-weeks' post-partum	3·15 (1·56)	2·85 (1·58)	..	0·25 (0·06 to 0·45)	..	NA
Median ferritin change from baseline (IQR), μg/L							
	4-weeks' post-treatment	195·45 (133·05–298·50)	29·85 (19·05–44·00)	..	..	6·49 (5·98 to 7·04)	NA
	Delivery	73·10 (32·80–155·80)	34·35 (18·90–63·35)	..	..	1·88 (1·65 to 2·15)	NA
	4-weeks' post-partum	69·30 (32·00–126·10)	31·20 (14·80–62·90)	..	..	1·95 (1·70 to 2·24)	NA
Iron deficiency							
	4-weeks post-treatment	1/403 (<1%)	101/392 (26%)	0·01 (0·00 to 0·07)	..	..	NA
	Delivery	78/375 (21%)	147/376 (39%)	0·53 (0·42 to 0·66)	..	..	NA
	4-weeks' post-partum	36/293 (12%)	90/311 (29%)	0·43 (0·30 to 0·60)	..	..	NA
Iron deficiency anaemia							
	4-weeks' post-treatment	1/394 (<1%)	84/381 (22%)	0·01 (0·00 to 0·08)	..	..	NA
	Delivery	24/352 (7%)	56/343 (16%)	0·40 (0·25 to 0·62)	..	..	NA
	4-weeks' post-partum	25/276 (9%)	59/275 (21%)	0·41 (0·27 to 0·64)	..	..	NA

Data are count (%), mean (SD), or median (IQR). p values and 95% CIs presented have not been adjusted for multiple comparisons for the secondary and other maternal and neonatal outcomes. The intervals should not be used in place of hypothesis testing. A prevalence ratio of ferric carboxymaltose versus standard of care is displayed following analyses using a modified Poisson model with robust standard errors and random intercept for participant. A mean difference of ferric carboxymaltose versus standard of care is following analyses using a likelihood-based longitudinal data analysis model. A geometric mean ratio is displayed for ferritin concentration after a log base e transformation due to skewness. An absolute mean difference between ferric carboxymaltose and standard of care is displayed following fitting a linear regression model. A risk ratio of ferric carboxymaltose iron versus standard of care is displayed following analyses using a log-binomial regression model. Anaemia indicates venous Hb <11·0 g/dL up to and including delivery and venous Hb <12·0 g/dL post partum. Moderate–severe anaemia indicates venous Hb <10 g/dL up to and including delivery and venous Hb <11·0 g/dL post-partum. Iron deficient indicates serum ferritin <15 μg/L or ferritin <30 μg/L if C-reactive protein >5 mg/L, and iron deficiency anaemia indicates Hb <11 g/dL and serum ferritin <15 μg/L or ferritin <30 μg/L if C-reactive protein >5 mg/L. Hb=haemoglobin.

* The Holm procedure was applied to the secondary maternal outcomes and secondary neonatal outcomes separately. The p value for ferritin (μg/L), iron deficiency, and iron deficiency anaemia at 36 weeks' gestation remained significant after controlling for multiple comparisons with the Holm procedure.

In women randomly assigned to ferric carboxymaltose, there was a reduction in the prevalence of iron deficiency at 36 weeks' gestation compared with standard care (ferric carboxymaltose: 60 [18%] of 336 *vs* standard of care: 142 [42%] of 341, PR 0·43 [95% CI 0·33–0·55]), at delivery (78 [21%] of 375 *vs* 147 [39%] of 376, 0·53 [0·42–0·66]), and at 4-weeks' post partum (36 [12%] of 293 *vs* 90 [29%] of 311, 0·43 [0·30–0·60]). Likewise, there was a reduction in iron deficiency anaemia at 36 weeks' gestation (ferric carboxymaltose: 29 [9%] of 324 *vs* standard of care: 93 [29%] of 321, 0·30 [0·20–0·44]), delivery (24 [7%] of 352 *vs* 56 [16%] of 343, 0·40 [0·25–0·62]), and 4-weeks' post partum (25 [9%] of 276 *vs* 59 [21%] 275, 0·41 [0·27–0·64]).

Compared with standard of care, ferric carboxymaltose did not change birthweight or incidence of low birthweight (table 3). 86 (12%) neonates were born via caesarean delivery (44 [12%] of 372 in the ferric carboxymaltose group, 42 [11%] of 366 in the standard of care group). Ferric carboxymaltose did not affect neonatal haemoglobin at 4 weeks' post partum or alter neonatal growth.

**Table 3:** Effects of ferric carboxymaltose on neonatal efficacy outcomes

		**Ferric carboxymaltose (n=395)**	**Standard of care (n=401)**	**Mean difference (95% CI)**	**Risk ratio (95% CI)**	**p value**
**Key outcome**						
Birthweight, g		2893·2 (496·7)	2896·2 (515·7)	−3·1 (−75·0 to 68·9)	..	0·93
**Secondary outcomes**						
Gestation duration, weeks		39·49 (1·95)	39·41 (2·29)	0·08 (−0·22 to 0·38)	..	0·59
Composite adverse birth outcome*		158/398 (40%)	155/407 (38%)	..	1·04 (0·88 to 1·24)	0·63
Individual components of composite adverse birth outcome						
	Low birthweight (<2500 g)	67/385 (17%)	61/378 (16%)	..	1·08 (0·79 to 1·48)	NA
	Pregnancy loss or stillbirth†	7/402 (2%)	9/410 (2%)	..	0·79 (0·30 to 2·11)	NA
	Premature birth (<37 weeks GA)	31/387 (8%)	35/385 (9%)	..	0·88 (0·55 to 1·40)	NA
	Small for gestational age‡	127/381 (33%)	117/376 (31%)	..	1·07 (0·87 to 1·32)	NA
Venous Hb 4-weeks post-partum, g/dL		11·96 (1·79)	11·94 (1·85)	0·01 (−0·33 to 0·36)	..	0·94
Weight 4-weeks post-partum, g§		3907·2 (667·7)	4005·2 (701·1)	−95·1 (−212·3 to 22·1)	..	0·11
**Other secondary outcomes**						
Birth length, cm		47·58 (3·51)	47·53 (3·62)	0·05 (−0·46 to 0·55)	..	NA
Length 4-weeks' post-partum, cm§		51·93 (4·06)	51·89 (3·58)	0·03 (−0·63 to 0·69)	..	NA
Weight for age Z score 4-weeks' post-partum§		−0·79 (1·28)	−0·60 (1·26)	−0·18 (−0·40 to 0·03)	..	NA
Length for age Z score 4-weeks' post-partum§		−1·13 (2·03)	−1·14 (1·82)	0·01 (−0·33 to 0·34)	..	NA
Weight for length Z score 4-weeks' post-partum§		0·04 (1·98)	0·40 (2·09)	−0·35 (−0·71 to 0·00)	..	NA

Data are count (%) or mean (SD). p values and 95% CIs presented have not been adjusted for multiple comparisons for the secondary and other maternal and neonatal outcomes. The intervals should not be used in place of hypothesis testing. Risk ratios of ferric carboxymaltose iron versus standard of care are following analyses using a log-binomial regression model. An absolute mean difference between ferric carboxymaltose and standard of care is displayed following fitting a linear regression model. Hb=haemoglobin.

* The composite adverse birth outcome was at least one adverse birth outcome of low birthweight, pregnancy loss or stillbirth, premature birth, or small for gestational age. The total number of the composite adverse birth outcome is therefore smaller than that of the components.

† Totals consist of n=395 liveborn neonates plus n=7 pregnancy loss or stillbirth in the ferric carboxymaltose group and n=401 liveborn neonates plus n=9 pregnancy loss or stillbirth in the standard of care group.

‡ Small for gestational age is defined as a birthweight below the tenth percentile for gestational age according to INTERGROWTH-21 standards.

§ Total number of neonates reduced due to procedural missing data (version control).

**Figure 2:**
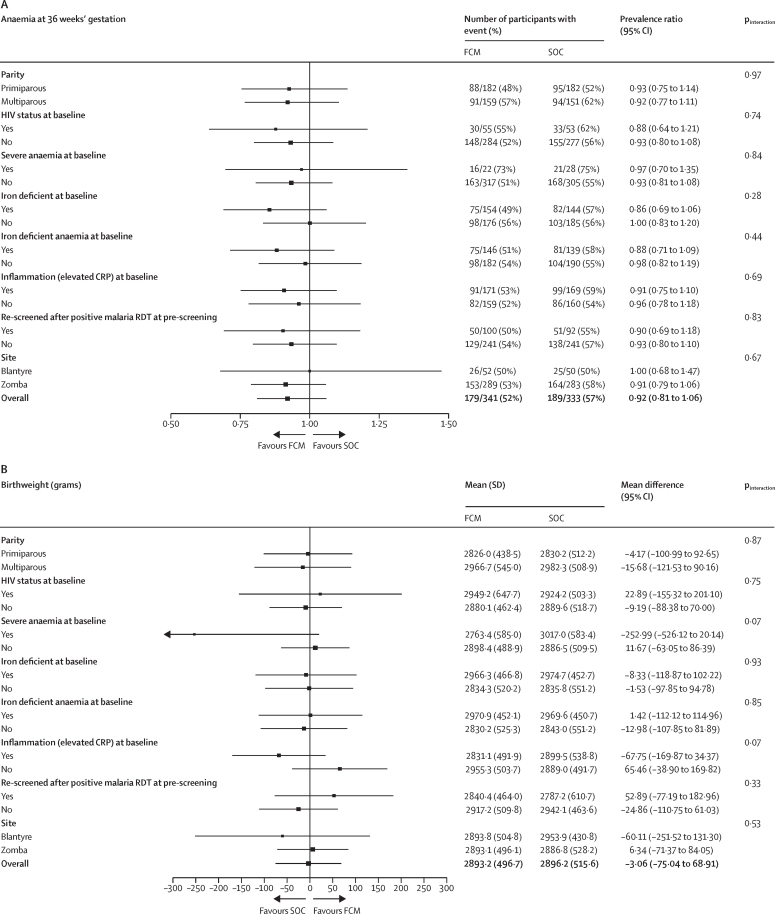
Subgroup analyses of the treatment effect on maternal anaemia at 36 weeks' gestation and birthweight according to baseline characteristics (A) A prevalence ratio of ferric carboxymaltose versus standard of care is displayed for anaemia at 36 weeks' gestation using a Poisson model with robust standard errors. Subgroup (main effect) and subgroup-by-treatment-by-visit interaction (and subgroup-by-treatment and subgroup-by-visit interaction) have been added to the model to evaluate how the treatment effect differs between subgroup categories. (B) An absolute mean difference for birthweight between ferric carboxymaltose and standard of care is displayed following fitting a linear regression model. Subgroup (main effect) and subgroup-by-treatment interactions terms have been added to the models to evaluate how the treatment effect differs between subgroup categories. The p values and 95% CIs presented have not been adjusted for multiple comparisons. The intervals cannot be used in place of hypothesis testing. FCM=ferric carboxymaltose. SOC=standard of care.

Prespecified subgroup analyses were conducted on the primary and key secondary maternal and neonatal outcomes (figure 2, appendix pp 223–30). Compared with standard of care, ferric carboxymaltose increased haemoglobin concentrations at 36 weeks' gestation among the 366 (43%) women with baseline iron deficiency and the 349 (42%) women with baseline iron deficiency anaemia. Women without baseline iron deficiency or iron deficiency anaemia at baseline who were randomly assigned to ferric carboxymaltose did not have increases in haemoglobin concentration at 36 weeks' gestation; this was reflected by larger effect sizes for the primary outcome of anaemia at 36 weeks' gestation among women with iron deficiency and iron deficient anaemia compared with women without baseline iron deficiency or iron deficiency anaemia. Baseline iron status did not influence the effect size for birthweight or other neonatal outcomes.

No women died during the trial. Adverse reactions during ferric carboxymaltose administration were reported by 28 (7%) of 430 participants; there were no serious adverse events including no serious hypersensitivity reactions (appendix p 230). From random assignment to 4 weeks' post partum, there were 353 maternal adverse events reported, with 183 (43%) of 430 women receiving ferric carboxymaltose and 170 (39%) of 432 women receiving standard of care reporting at least one adverse event (risk ratio [RR] 1·08 [95% CI 0·92–1·27], p=0·34; table 4). There was no significant difference in adverse events classified by any particular systemic organ class or preferred term, including no increase in infection; specifically, no increase in clinical malaria (ferric carboxymaltose: 26 [6%] of 430 *vs* standard of care: 20 [5%] of 432; RR 1·31 [0·74–2·30], p=0·36; table 4; appendix pp 231–36). 17 (4%) mothers who received ferric carboxymaltose had at least one serious adverse event compared with 19 (4%) mothers who received standard of care. There was no evidence of an effect of ferric carboxymaltose on the composite maternal safety outcome of death, haemorrhage, transfusion, or intensive care admission. Ferric carboxymaltose did not change the risk of pregnancy loss or stillbirth. Ferric carboxymaltose did not change biochemical inflammation (measured by C-reactive protein; table 4).

**Table 4:** Effects of ferric carboxymaltose on maternal and neonatal safety outcomes

		**Ferric carboxymaltose (n=430)**	**Standard of care (n=432)**	**Risk ratio (95% CI)**	**Prevalence ratio (95% CI)**	**p value**
Maternal safety outcome*						
Adverse events						
At least one adverse event		183 (43%)	170 (39%)	1·08 (0·92, 1·27)	..	0·34
At least one serious adverse event		17 (4%)	19 (4%)	0·90 (0·47–1·70)	..	0·75
Death		0	0	NA	NA	NA
Adverse events of special interest						
Composite severe medical event†		14 (3%)	10 (2%)	1·40 (0·63–3·12)	..	0·41
Individual components of severe medical events						
	Death	0	0	NA	NA	NA
	Haemorrhage	9 (2%)	7 (2%)	1·29 (0·49–3·43)	..	0·61
	Blood transfusion required for mother	10 (2%)	6 (1%)	1·67 (0·61–4·56)	..	0·31
	ICU care required for mother	0	0	NA	NA	NA
**Common adverse events occurring in >5% in any treatment group**‡						
System organ class						
	Infections and infestations	103 (24%)	87 (20%)	1·19 (0·92–1·52)	..	0·19
	Pregnancy, puerperium, and perinatal conditions	72 (17%)	75 (17%)	0·97 (0·72–1·30)	..	0·83
Preferred term						
	Urinary tract infection	51 (12%)	45 (10%)	1·14 (0·78–1·66)	..	0·50
	Infections and infestations—malaria	26 (6%)	20 (5%)	1·31 (0·74–2·30)	..	0·36
	Pregnancy, puerperium, and perinatal conditions—perineal tear	44 (10%)	47 (11%)	0·94 (0·64–1·38)	..	0·74
Hypophosphatemia§						
	4-weeks' post-treatment	22/403 (5%)	8/392 (2%)	..	2·67 (1·20–5·93)	0·016
	Mild	13/403 (3%)	8/392 (2%)	NA	NA	NA
	Moderate	9/403 (2%)	0	NA	NA	NA
	Severe	0	0	NA	NA	NA
36 weeks' gestation		3/336 (1%)	2/341 (<1%)	..	1·52 (0·26–9·04)	0·65
	Mild	3/336 (1%)	2/341 (<1%)	NA	NA	NA
	Moderate	0	0	NA	NA	NA
	Severe	0	0	NA	NA	NA
Delivery		6/375 (2%)	4/376 (1%)	..	1·50 (0·43–5·29)	0·53
	Mild	4/375 (1%)	3/376 (1%)	NA	NA	NA
	Moderate	2/375 (<1%)	1/376 (<1%)	NA	NA	NA
	Severe	0	0	NA	NA	NA
4-weeks' post-partum		3/293 (1%)	3/311 (1%)	..	1·06 (0·22–5·22)	0·94
	Mild	1/293 (<1%)	2/311 (<1%)	NA	NA	NA
	Moderate	1/293 (<1%)	1/311 (<1%)	NA	NA	NA
	Severe	0	0	NA	NA	NA
Inflammation¶						
	4-weeks' post-treatment	152/403 (38%)	152/392 (39%)	..	0·97 (0·82–1·16)	0·76
	36 weeks' gestation	118/336 (35%)	126/341 (37%)	..	0·95 (0·78–1·16)	0·62
Delivery		283/375 (75%)	298/376 (79%)	..	0·95 (0·88–1·03)	0·22
	4-weeks' post-partum	52/293 (18%)	56/311 (18%)	..	0·99 (0·70–1·39)	0·93
Malaria RDT positive						
	4-weeks' post-treatment	30/398 (7%)	18/386 (5%)	1·62 (0·92–2·84)	..	0·097
	36 weeks' gestation	19/339 (6%)	10/331 (3%)	1·85 (0·88–3·92)	..	0·11
	Delivery	15/313 (5%)	24/328 (7%)	0·66 (0·35–1·23)	..	0·19
	4-weeks' post-partum	9/321 (3%)	13/313 (4%)	0·69 (0·30–1·58)	..	0·38
**Neonatal safety outcome**‖						
	Adverse events					
	At least one adverse event	30/395 (8%)	35/401 (9%)	0·87 (0·55–1·39)	..	0·56
	At least one serious adverse event	12/395 (3%)	18/401 (4%)	0·68 (0·33–1·39)	..	0·29
	Death	10/395 (2%)	17/401 (4%)	0·60 (0·28–1·29)	..	0·19
	Death and pregnancy loss	17/402 (4%)	26/410 (6%)	0·67 (0·37–1·21)	..	0·18

The p values and 95% CIs presented have not been adjusted for multiple comparisons. The intervals should not be used in place of hypothesis testing. Risk ratios of ferric carboxymaltose iron versus standard of care are following analyses using a log-binomial regression model. Prevalence ratios of ferric carboxymaltose versus standard of care is displayed following analyses using a modified Poisson model with robust standard errors and random intercept for participant. CRP=C-reactive protein. ICU=intensive care unit. NA=not applicable. RD=rapid diagnostic test.

* Includes all women who were treated, presented according to treated group. This includes four mothers with multiple pregnancies.

† The composite severe medical event outcome was women with at least one severe medical event of dealth, haemorrhage, blood transfusion, or ICU care recorded for the mother. Death, haemorrhage, blood transfusion, and admission to ICU are captured across the whole trial period.

‡ Adverse events were coded using version 5.0 of the Common Terminology Criteria for Adverse Events^32^ using an appropriate Preferred Term and System Organ Class for each adverse event verbatim term.

§ Hypophosphatemia is defined as PO4 <0·80 mmol/L. Hypophosphatemia was seen at baseline in 15 (3·6%) of 418 women treated with ferric carboxymaltose and 2 (5·6%) of 425 women treated with standard of care. Mild hypophosphatemia is defined as (0·64≤PO4<0·80 mmol/L), moderate hypophosphatemia is defined as (0·32 ≤PO4 <0·64 mmol/L) and severe hypophosphatemia is defined as (PO4<0·32 mmol/L). Severity of hypophosphatemia does not have statistical testing due to sparse data.

¶ Inflammation indicates C-reactive protein >5 mg/L.

‖ Includes all liveborn neonates (with the exception of the death and pregnancy loss outcome) born to women who were treated, presented according to treated group of the mother; this includes eight twins.

Ferric carboxymaltose is recognised to induce transient hypophosphataemia.^33^, ^34^, ^35^ At 4 weeks' post-treatment, the prevalence of hypophosphataemia (serum phosphate <0·80 mmol/L) among women randomly assigned to ferric carboxymaltose was 22 (5%) of 403, compared with 8 (2%) of 392 among the standard of care group (PR 2·67 [1·20–5·93] p=0·016; table 4); this difference comprised an increase in moderate hypophosphataemia among women treated with ferric carboxymaltose. No difference in prevalence of hypophosphataemia between groups was observed at subsequent timepoints.

Among the 796 babies born alive, 27 (3%) died during the first 28 days of life: ten (3%) born to mothers who received ferric carboxymaltose and 17 (4%) to mothers who received standard of care (RR 0·60 [95% CI 0·28–1·29], p=0·19). At least one adverse event was reported in 30 (8%) neonates born to mothers randomly assigned to ferric carboxymaltose and 35 (9%) born to mothers who received standard of care (RR 0·87 [0·55, 1·39], p=0·56; table 4). There was no significant difference in neonatal adverse events classified by any particular systemic organ class or preferred term (appendix pp 231–36).

We conducted several additional analyses to confirm the robustness of the findings to the analysis assumptions or methods, which did not alter the results (appendix pp 237–45).

We did one unplanned analysis after unblinding. The original primary outcome definition was designed to characterise the effect of ferric carboxymaltose on anaemia as women reached delivery; we recognised the amended primary outcome would miss women who delivered before 36 weeks' gestation. For this reason, we analysed a post-hoc outcome: anaemia at 36 weeks' gestation or at delivery—whichever occurred sooner. Ferric carboxymaltose did not reduce the prevalence of anaemia at this post-hoc composite timepoint compared with standard of care (ferric carboxymaltose: 187 [52%] of 357 *vs* standard of care: 200 [56%] of 354, PR 0·93 [95% CI 0·81–1·06]).

## Discussion

In resource-limited setting in sub-Saharan African, where adverse pregnancy and neonatal outcomes are common and malaria is highly endemic, treatment of women with moderate or severe anaemia in the second trimester of pregnancy with a single dose of up to 1000 mg ferric carboxymaltose was not significantly superior to standard of care in improving the primary maternal outcome of anaemia at 36 weeks' gestation or the primary neonatal outcome of birthweight. However, ferric carboxymaltose reduced iron deficiency and iron deficiency anaemia. Ferric carboxymaltose might be more effective in increasing haemoglobin in women with baseline iron deficiency, although the statistical evidence for this interaction was not strong. In this resource-constrained primary-care setting where exposure to infectious diseases was intense, ferric carboxymaltose was safe.

We used pragmatic eligibility criteria for the trial so that the results could be translated into practice. We reasoned that in resource-limited primary antenatal health-care, screening with venous blood currently represents an insurmountable transformation of practice, as laboratory screening for anaemia in any form is seldom performed. Discrepancies between capillary and venous haemoglobin assessments are recognised,^36^ but 85% of women exhibited moderate or severe anaemia and 95% exhibited anaemia by venous testing. Testing for ferritin or other biomarkers of iron status is generally unavailable in field settings in resource-limited settings. Ferric carboxymaltose was highly effective in reducing the prevalence of iron deficiency anaemia throughout pregnancy and into the post partum, but the benefit appears to have been diluted by an absence of effect for those with non-iron deficiency anaemia. Our study highlights a need for field-friendly testing capabilities for causes of anaemia, including iron status.

Our findings highlight the complexity of the determinants of anaemia in this setting. Inflammation was present in more than half of women at baseline, despite testing negative for *Plasmodium* by RDT. In pregnancy, conventional RDTs for *P falciparum* might be insensitive, as parasites might sequester in the placenta.^37^ Thus, participants who had a negative RDT potentially still harboured parasitaemia. Other causes of anaemia in this population might include haemoglobinopathies; we did not test for these at baseline but we did exclude women with a known history of an inherited red cell disorder. A cross-sectional study in Malawian children revealed that 10% of them harboured homozygote α^3·7^ genotypes (which can cause mild anaemia) and about 10% carried the sickle cell gene (although this is unlikely to cause anaemia).^38^ Hookworm and schistosomiasis were not specifically treated in this trial, but might cause chronic blood loss that can contribute to iron deficiency in this population, and would be expected to be responsive to iron therapy.

The reduction in iron deficiency produced by ferric carboxymaltose could potentially provide benefits to mothers and their babies independent of effects on anaemia. 2021 data indicate that universal iron interventions in 8-month-old infants do not improve cognitive outcomes,^22^ perhaps because iron deficiency affects brain development earlier in life. Maternofetal iron deficiency has been associated with an increased risk of neurocognitive and mental health disorders in the child, including poorer neurodevelopment and school performance, slower neural processing, difficulties with planning and attention, and increased risks of socioemotional problems including anxiety.^39^ Studies exploring relationships between infant and childhood iron deficiency and neurodevelopmental disorders such as autism spectrum disorder and attention deficit hyperactivity disorder have largely been correlative and have found inconsistent evidence of association.^40^ Therefore, it will be valuable to follow the cohort to determine whether children born to women receiving ferric carboxymaltose have improved developmental trajectories and altered risk of neurodevelopmental disorders.

Despite iron therapy, and accounting for recurrent intermittent preventive therapy, approximately half of women remained anaemic by 36 weeks' gestation; this might relate to the ongoing high prevalence of inflammation (∼36%) seen at this timepoint, perhaps due to causes beyond malaria. Previous randomised controlled trials and systematic reviews have suggested a benefit from iron supplementation on birthweight.^4^, ^28^, ^41^, ^42^ Systemic inflammation might have distorted healthy maternal and placental iron homoeostasis in this population.^43^, ^44^

Systematic reviews have indicated that intravenous iron might increase infection risk compared with oral iron (or no iron);^45^ we noted a non-significant increase in infections (overall, and including malaria) among women randomly assigned to ferric carboxymaltose. Given concerns that iron-induced erythropoiesis might increase susceptibility to malaria,^46^ we ensured that women had a negative RDT at recruitment, and that they received IPTp-SP. Antimalarials (as preventives or treatments) might have controlled parasitemia, reduced anaemia, and perhaps increased birthweight across both trial groups.^47^, ^48^

Ferric carboxymaltose is known to induce transient hypophosphataemia due to FGF23-induced renal-phosphate losses;^33^ we observed a small, transient increase in the prevalence of moderate hypophosphataemia. Hypophosphataemia might have been more marked before 28 days but in any case, the effect is transient. We did not observe any serious infusion-related hypersensitivity reactions in this study, and our field team was able to administer the drug in basic primary health centres remote from a hospital and without resources such as electricity. These initial experiences indicate that the use of ferric carboxymaltose could be feasible in sub-Saharan resource-limited settings.

Several systematic reviews have indicated that intravenous iron is superior to oral iron in increasing haemoglobin and reducing anaemia prevalence during pregnancy; these reviews contain limited data from low-income countries, or from trials using modern intravenous formulations including ferric carboxymaltose.^42^ A network meta-analysis identified advantages from intravenous oral iron over oral iron formulations in increasing haemoglobin concentration 4 weeks' post-administration (but did not report this outcome closer to delivery).^49^ Few randomised trials have assessed the use of ferric carboxymaltose in low-income settings: a small study in Tanzania randomly assigned iron deficient anaemic post-partum women to oral iron or ferric carboxymaltose, and showed that sustained haemoglobin increases from ferric carboxymaltose.^50^ The FER-ASAP trial recruited iron-deficient anaemic pregnant women in high-income and middle-income settings and showed early superiority from ferric carboxymaltose over oral iron on haemoglobin concentrations, which were not sustained beyond 6 weeks.^27^ Our study is the largest trial to compare ferric carboxymaltose (or other modern iron formulations) to standard of care in pregnancy, the first in a resource-limited malaria-endemic setting, and the first to assess post-partum effects of antenatal ferric carboxymaltose.

Hepcidin regulates iron access to the plasma from the diet and the reticuloendothelial system.^51^ In pregnancy, maternal hepcidin is suppressed to facilitate increased absorption and use and to support expanded maternal erythropoiesis and fetal iron requirements. Homoeostatatic iron-induced hepcidin upregulation appears to be blunted during pregnancy,^44^ but upregulation due to inflammation seems preserved.^43^ Assessment of hepcidin levels as a potential biomarker of response to ferric carboxymaltose in this population, and the effect of ferric carboxymaltose (compared with oral iron) on hepcidin in this setting with an interacting burden of inflammation will be an important exploratory post-hoc substudy of this trial. The standard of care group comprised twice daily oral iron as ferrous sulphate, which is aligned with WHO and Malawian guidelines; stable isotope studies in non-pregnant women suggest daily oral iron might offer more efficient absorption,^52^, ^53^ although physiology might differ in pregnancy. Ongoing support to enhance antenatal oral iron programmes should continue.

Our trial has several strengths, and included women who face a high risk of adverse antenatal and perinatal outcomes (for whom optimising pregnancy outcomes is most crucial) using pragmatic eligibility criteria that could be implemented in the field. Limitations in our study design include the open-label design, but since ferric carboxymaltose is a dark solution, administering intravenous placebo would require a curtain, blindfold, or opaque tubing. Key efficacy outcomes were measured by masked personnel. Several major trials of intravenous iron in high-income settings, such as the IRONMAN study testing iron derisomaltose in heart failure^54^ and the FER-ASAP trial,^27^ similarly adopted an open-label design due to complexities of a placebo. Our study design was compromised by the COVID-19 pandemic, which necessitated urgent amendments to protect participants and their communities, resulting in changes to the primary outcome and limited ability to assess adherence in the control group. Women might also have concealed poor adherence to oral tablets to meet the expectations of health workes.^55^ The pandemic probably also affected follow-up: when reasons were given, COVID-19 concerns were the main reason cited by participants for missing scheduled visits. We used C-reactive protein as the sole biomarker to adjust ferritin for inflammation, and to estimate prevalence of inflammation. C-reactive protein is elevated in chronic inflammation and transiently after acute inflammation; use of a biomarker exhibiting sustained elevation after inflammation (such as α_1_-acid glycoprotein) might have indicated a higher prevalence of inflammation, but also uncovered a higher prevalence of iron deficiency.^56^ Defining inflammation using a higher C-reactive protein threshold would have correspondingly reduced prevalence of inflammation. Health economic analyses of the trial are planned. At present, ferric carboxymaltose remains expensive; cheaper solutions will need to be developed to enable programmes to be cost effective.

Ferric carboxymaltose can feasibly be delivered safely in resource-poor contexts where infection risk is high. Further exploration of the role that ferric carboxymaltose might play as part of the solution to the complex determinants of anaemia in sub-Saharan Africa is warranted.

## Data sharing

Underlying deidentified individual participant data encompassing the reported trial results and a data dictionary are accessible at figshare (https://doi.org/10.26188/22344973). Data are available under the terms of Creative Commons Attribution 4.0 International License (CC-BY-4.0).
