# Supplementary material for: Ferric carboxymaltose versus standard-of-care oral iron to treat second-trimester anaemia in Malawian pregnant women: a randomised controlled trial
Source: Lancet. 2023 May 13;401(10388):1595–609. doi: 10.1016/S0140-6736(23)00278-7 (PMC10193370; doi:10.1016/S0140-6736(23)00278-7)
Supplement: Supplementary appendix [file mmc1.pdf]

THE LANCET

Supplementary appendix

This appendix formed part of the original submission and has been peer reviewed. We post it as supplied by the authors.

Supplement to: Pasricha S-R, Mwangi MN, Moya E, et al. Ferric carboxymaltose versus standard-of-care oral iron to treat second-trimester anaemia in Malawian pregnant women: a randomised controlled trial. *Lancet* 2023; published online April 20. https://doi.org/10.1016/S0140-6736(23)00278-7.

Ferric Carboxymaltose for anemia in the second trimester of pregnancy in Malawian women: Supplementary Appendix Content

Appendix 1: Standard Operating Procedure for administration of Ferric Carboxymaltose	Page 2
Appendix 2: REVAMP Protocols and Statistical Analysis Plan	Page 11
Appendix 3: Supplementary Data	Page 220
Supplemental Table 1: Baseline Characteristics of the Participating Pregnant Women – (Per - Protocol Population	Page 221
Supplemental Table 2: Subgroup Analyses of Effect of Ferric Carboxymaltose on Key Maternal Outcomes at 36 Weeks' Gestation and Neonate Outcomes at Delivery According to Baseline Characteristics	Page 223
Supplemental Table 3: Maternal Safety Outcomes - Adverse Reactions During Ferric Carboxymaltose Administration	Page 230
Supplemental Table 4: Effects of Ferric Carboxymaltose on Maternal and Neonatal Adverse Events Classified by Systemic Organ Class or Preferred Term	Page 231
Supplemental Table 5: Additional Analyses on Maternal and Neonate Outcomes	Page 237

Ferric Carboxymaltose for anemia in the second trimester of pregnancy in Malawian women: Supplementary Appendix

Standard Operating Procedure for administration of Ferric Carboxymaltose

RANDOMIZED CONTROLLED TRIAL OF THE EFFECT OF INTRAVENOUS IRON ON ANAEMIA IN MALAWIAN PREGNANT WOMEN (REVAMP)

UNIVERSITY OF MALAWI

TRUE CENTRE

Training and Research Unit of Excellence Centre

**SOP TITLE: Ferinject Administration**	**VERSION: 2.0**
**SOP NUMBER: 23**	**Effective date: 01st Sep 2019**

CONTENTS

Abbreviations: 4

1. Purpose: 5
2. Allowable Exceptions: 5
3. Personnel Responsible: 5
4. Definitions: 5
5. The Drug; 5
6. Procedure: 5
7. Undesirable Effects: 8
8. Contraindications: 8
9. Warning and Precautions: 8
10. Training: 8
11. References: 8

ABBREVIATIONS

Co-PI: Co-Principle Investigator

FCM: Ferric Carboxymaltose

PI: Principle Investigator

SOP: Standard Operating Procedure

1. **PURPOSE**

This standard operating procedure is to guide the study clinical team in administering intravenous Ferinject (FCM) for the study participants.
This SOP is applicable to both Blantyre and Zomba sites

2. **ALLOWABLE EXCEPTIONS**

This SOP is meant to be followed without deviation. However, it is an allowable exception to follow procedures specified in a protocol that may deviate from this SOP.

3. **PERSONNEL RESPONSIBLE**

This guideline applies to all nursing and medical staff working within the REVAMP study. It is intended as a guide towards best practice for all members of the clinical staff involved in the administration of intravenous Ferinject. The nurse/clinician administering Ferinject is responsible for:

3.1 Maximise participant's comfort and safety
3.2 Recording participant's observations; blood pressure and pulse pre, during and post administration of Ferinject.
3.3 Partake in the venepuncture and cannulation programme and be deemed competent.
3.4 Observe, report and record any complications to study clinician, arising from all aspects of administering Ferinject
3.5 Effectively manage complications that may occur as per the guidelines

4. **DEFINITION**

Intravenous iron Ferinject solution for infusion contains iron in a stable ferric state as a complex with a carbohydrate polymer designed to release utilisable iron to the iron transport and storage proteins in the body (ferritin and transferrin).

5. **THE DRUG**

5.1. Description and presentation:
5.1.1 Ferinject® is a dark brown, non-transparent, colloidal solution in a vial (type I glass) with bromobutyl rubber stopper and aluminium cap
5.1.2 Each 10ml vial contains 500mg of elemental iron
5.1.3 Solution for intravenous use only

5.2. Storage:
5.2.1 Store in original package
5.2.2 Do not store above 30 degrees Celsius. Do not freeze, do not refrigerate
5.2.3 Shelf life is 36 months in original packaging
5.2.4 After opening of container: from a microbiological point of view, preparations for parenteral administration should be used immediately
5.2.5 After dilution with sterile 0.9% sodium chloride solution: use as soon as practicable after dilution. If storage is necessary, store at 2-8 degrees Celsius for not more than 12 hours

5.3. Drug dose:
5.3.1 Weight ≥50kg: 1000mg (maximum as single dose)
5.3.2 Weight <50kg: 20mg of iron/kg body weight

6. **THE PROCEDURE**

6.1. Equipment required for administration of Ferinject
6.1.1. Ferinject® (available in 500mgs in 10mls

5.1.1 Alcohol swabs, gauze squares
5.1.2 22 gauge (blue) cannula
5.1.3 Adhesive tape or cannula dressing
5.1.4 green needles
5.1.5 5ml and 20ml syringe
5.1.6 250ml Normal Saline 0.9%
5.1.7 Vacutainer blood bottles (if required)
5.1.8 Sharp container

5.2 Preparation Ferinject® Infusion

5.2.1 Inspect vials visually for sediment and damage before use. Only use vials containing sediment-free, homogenous solution
5.2.2 Make up infusion in 250ml bag 0.9% sodium chloride
Compatible solutions: 0.9% sodium chloride only
Volume to be removed from IV bag: Nil
Compatible containers: polyethylene and glass
5.2.3 Each vial of Ferinject is intended for single use only
5.2.4 To be administered alone

5.3 Pre-treatment requirements

5.3.1 A trained nurse or doctor should be present in the infusion location throughout the procedure to monitor for any reactions during the infusion
5.3.2 Review treatment of anaphylaxis in adults prior to commencement of infusion
5.3.3 Ensure oxygen and suction equipment are available and in working order
5.3.4 Ensure resuscitation equipment and emergency drugs, such as adrenaline and hydrocortisone are available
5.3.5 Test dose of ferric carboxymaltose is not required
5.3.6 Premedications are generally not required

5.4 Ferinject administration

5.4.1 Explain the procedure to the participants
5.4.2 Wash hands
5.4.3 Wear Personal Protective Equipment: gloves, apron
5.4.4 Monitor baseline vital signs: temperature, pulse, respiratory rate and blood pressure
5.4.5 Prepare skin with alcohol street and allow it to dry
5.4.6 Insert a 22 gauge (blue) Cannula
5.4.7 Secure cannula in position with tape or cannula dressing
5.4.8 Flush with 5mls Normal Saline to ensure patent veins
5.4.9 Administer 250ml Ferinject solution via intravenous infusion over 15 minutes
5.4.10 Observe patient for any adverse events during and immediate after Ferinject administration
5.4.11 Flush cannula with 5mls Normal Saline post administration
5.4.12 Post injection observations: repeat temperature, pulse, respiratory rate and blood pressure at completion of infusion
5.4.13 Monitor participant for 30 minutes after the completion of the infusion to assess for potential adverse effects

5.4.14 If no side effects are observed after 30 minutes no further monitoring is required. Remove cannula, apply pressure, extend and elevate the arm

7. UNDESIRABLE EFFECTS

7.1 Immediate and severe:

Anaphylactoid* reactions are rare, and occur most frequently within the first several minutes of administration and are generally characterised by sudden onset of respiratory difficulties including angioedema, wheezing, urticaria, respiratory and circulatory collapse

7.1.1 Stop the infusion immediately if any of these symptoms occur. Contact medical officer immediately.

* Anaphylactoid reactions resemble anaphylactic reactions without the involvement of IgE antibodies. Their clinical manifestations are indistinguishable requiring the same treatment

7.2 Other side effects:

7.2.1 Headache, dizziness (common)
7.2.2 Nausea, abdominal pain, diarrhoea, constipation (common)
7.2.3 Injection site reactions: pain, irritation, discolouration (common)
7.2.4 Myalgia, back pain and arthralgia (uncommon)
7.2.5 Hypotension, flushing, tachycardia (uncommon)
7.2.6 Fever, rigors, chest pain (uncommon)
7.2.7 Pruritis, urticaria, rash (uncommon)
7.2.8 Dyspnoea (rare)
7.2.9 Anaphylactic reactions (rare)

8. CONTRA-INDICATIONS

8.1 Ferinject should not be used during the first trimester of pregnancy
8.2 Known hypersensitivity to ferric carboxymaltose complex, to Ferinject or any of its excipients
8.3 Evidence of iron overload or known to be iron replete

9. WARNINGS AND PRECAUTIONS

9.1 Parentally administered iron preparations can cause hypersensitivity reactions. Therefore, equipment for cardio-pulmonary resuscitation must be available in rooms where Ferinject is being administered.
9.2 Care must be taken to prevent paravenous leakage. In case of paravenous leakage the administration of Ferinject must be stopped immediately.
9.3 Caution should be used when administering intravenous iron to Patients with a history of asthma, eczema or other atopic allergy or acute/ chronic infection.
9.4 Ferinject should not be administered concomitantly with oral preparations of iron as the absorption of iron will be reduced.

10. TRAINING

10.1 Each staff member receives or has direct access to this SOP.
10.2 New staff should be trained on Ferinject administration prior to their participation in the study specific procedures and being entered in the delegation log.
10.3 Staff members whose duties fall within this SOP scope are retrained within 7 days of the approval of each SOP revision.
10.4 Each staff member should be trained in the treatment of anaphylaxis

11. REFERENCES

11.1 Ferinject Administration brochure

Approved by

Designation	Name and Signature	Date
Principle Investigator	Kamija Phiri:	01st Nov, 2018
Team Leader	Martin Mwangi:	01st Nov, 2018

DOCUMENT CONTROL SECTION TRACKING SOP REVIEW

Purpose:

- The log records review dates of this SOP and the status of the review. The Tracking Changes and Version Control Log are completed to detail status of the review.

When

- The SOP is reviewed every year or more often when necessary.

By whom:

- The SOP is reviewed by staff directly following the SOP. The review process is overseen by PIs/designee and reviewed by review team.

Supersedes version number	Date & New number	version	Reason for review/change	Name of reviewer
Version 1.0	1st Sep 2019	Version 2.0	Routine Review	Ernest Moya

Ferric Carboxymaltose for anemia in the second trimester of pregnancy in Malawian women: Supplementary Appendix

REVAMP Protocols and Statistical Analysis Plan

Table of Contents

REVAMP PROTOCOL Version 2.1 12

REVAMP PROTOCOL Version 6.0 74

Changes to the Protocol (Between Version 2.1 and Version 6.0) 172

REVAMP Statistical Analysis Plan Version 2.0 173

Changes to the Statistical Analysis Plan 220

REVAMP PROTOCOL Version 2.1

Protocol used at trial opening

PROJECT PROPOSAL

Randomized controlled trial of the Effect of intraVenous iron on Anaemia in Malawian Pregnant women

Short Title: **REVAMP Study**

Study Identifiers:

COMREC:	WEHI REC:
[P.02/18/2357]	[####]

Co-Principal Investigators:

- **Prof Kamija Phiri, Department of Public Health, College of Medicine, University of Malawi, Private Bag 360, Chichiri, Blantyre 3, Malawi. Mobile: +265 999957 048; E-mail: kamijaphiri@gmail.com**
- **Dr Sant-Rayn Pasricha, Walter and Eliza Hall Institute of Medical Research, 1G Royal Parade, Parkville, Melbourne 3052, Australia. Mobile: +61407141570; E-mail: Pasricha.s@wehi.edu.au**

Co-Investigators: **See Page 2**

Institutions: **See Page 2**

Funder: **Bill & Melinda Gates Foundation**

Amendment History:

Date	Protocol Version	Details of Changes	Signature Principal Investigator
31 Jan 2018	1.1	Original Protocol	

**26 July, 2018**	**2.0**	**Amendment – not approved**	
**28 August, 2018**	**2.1**	**Summary of changes made:** **(*see attached amendment form*)**	

1. TITLE OF RESEARCH PROPOSAL

Randomized controlled trial of the effect of intravenous iron on anaemia in Malawian pregnant women

2. INVESTIGATORS AND INSTITUTIONAL AFFILIATIONS

2.1. INVESTIGATORS

2.1.1. *Co-principal Investigators*

- **Prof Kamija Phiri** — **Department of Public Health, College of Medicine, University of Malawi, Private Bag 360, Chichiri, Blantyre 3, Malawi. Mobile: +265 999957 048; E-mail: kphiri@medcol.mw**
- **Dr Sant-Rayn Pasricha** — **Walter and Eliza Hall Institute of Medical Research, The University of Melbourne, 1G Royal Parade, Parkville, Melbourne 3052, Australia. Mobile: +61 407 141 570; E-mail: pasricha.s@wehi.edu.au**

2.1.2. *Co-investigators*

1. **Professor Bjarne Robberstaad**[1]
2. **Professor Khalid Khan**[2]
3. **Professor Stephen Rogerson**[3]
4. **Professor William Stones**[5]

5. **Dr Kabeya Biselele[6]**

6. **Dr Martin Mwangi[5]**

7. **Dr Jobiba Chinkhumba[9]**

8. **Dr Glory Mzembe, MD[5] (PhD student)**

9. **Mr Ernest Moya[5] (PhD student)**

2.1.3. *Trial Statistician*

10. **Professor Julie Simpson[4]**

2.1.4. *Collaborators*

11. **District Health Officer, Blantyre[5]**

12. **District Health Officer, Zomba[6]**

2.2. Institutions

1. **Centre for International Health, University of Bergen, Bergen, Norway**

2. **Queen Mary University of London, London, UK**

3. **The University of Melbourne, Melbourne, Australia**

4. **Centre for Epidemiology and Biostatistics, Melbourne School of Population and Global Health, The University of Melbourne, Melbourne, Australia**

5. **Department of Public Health, College of Medicine, Blantyre, Malawi**

6. **Zomba Central Hospital, Zomba, Malawi**

7. **Blantyre District Assembly, Blantyre, Malawi**

8. **Zomba District Assembly, Zomba, Malawi**

9. **Malaria alert centre, College of Medicine, Blantyre, Malawi**

3. PROTOCOL ABSTRACT

Aim	**To determine whether IV ferric carboxymaltose (FCM) given once during the second trimester is effective and safe in improving maternal and neonatal outcomes for treatment of moderate to severe maternal anaemia among pregnant women in Blantyre and Zomba districts of Malawi.**
Research Objectives	***Primary objective*: To determine whether FCM up to 1000 mg given once during the second trimester is superior to oral iron as administered by routine health services during pregnancy in the reduction of maternal anaemia at term.**<br><br>***Secondary Objectives***<br><br>**1. To determine if FCM improves maternal anaemia at term when compared with routinely delivered oral iron.**<br><br>**2. To determine the effectiveness of FCM compared to routinely delivered oral iron on critical neonatal outcomes including birth weight (low birth weight), gestation duration (prematurity), small for gestational age and other adverse birth outcomes.**<br><br>**3. To determine the effectiveness of FCM compared to routinely delivered oral iron on maternal outcomes including haemoglobin concentration and iron deficiency (measured through iron biomarkers), ~~and~~ maternal quality of life and maternal cognitive function.**<br><br>**4. To determine the safety of FCM compared to oral iron on the prevalence of all-cause sick visits, serious maternal adverse events, clinical malaria, bacteraemia, placental malaria, malaria parasitaemia, hypophosphatemia and pathogenic vaginal bacterial flora during pregnancy.**<br><br>**5. To determine immune and molecular aetiologies that may be related to anaemia, iron deficiency and infection in pregnancy.**<br><br>**6. To determine the cost-effectiveness of FCM compared to oral iron measured through absolute and incremental health benefits and incremental provider and patient costs.**
Study Design	**18-month, Phase III open-label 2-arm parallel group randomized controlled trial recruiting pregnant women in their second trimester and following them up until 1 month postpartum. This is an effectiveness trial and will be carried out in real-life settings in health**

	facilities in Blantyre and Zomba district.
Intervention	Intervention group will receive IV ferric carboxymaltose 1000mg (for women with weight >50kg), or 20mg/kg (for women with weight <50kg) once during the second trimester.<br><br>Control group will receive oral iron 200mg ferrous sulphate (approx. 65mg elemental iron) twice daily for 90 days or the duration of pregnancy, whichever is shorter.
Primary outcome	Prevalence of maternal anaemia (Hb <11.0g/dl) pre-delivery
**Target Population**	**Inclusion criteria**<br>● Confirmed singleton pregnancy at 13-26 weeks gestation<br>● Moderate to severe anaemia not requiring an immediate blood transfusion (Hb <10g/dl)<br>● Negative malaria parasitaemia<br>● Resident in the study catchment area of Blantyre and Zomba district<br>● Plan to deliver at health facility<br>● Written informed consent (including assent if <18 years old)<br>**Exclusion criteria**<br>● Hypersensitivity to any of the study drugs<br>● Clinical symptoms of malaria or bacterial infection<br>● Any condition requiring hospitalization or serious concomitant illness<br>● Chronic illness that may adversely affect foetal growth and viability<br>● Clinically low haemoglobin requiring a blood transfusion (usually Hb <5g/dl)
**Sample size**	862 women (431 per arm)
**Primary data collection & sampling method**	Outcome assessments will be measured through:<br>1. Scheduled and unscheduled participant visits to health facilities<br>2. Laboratory molecular and biological assays

	**3. Economic evaluation for incremental costs determination**

3.1.

4. EXECUTIVE SUMMARY

Type of study: Open label two-arm Phase III individual-randomized controlled trial

Problem: Anaemia in pregnancy remains a critical global health problem, affecting 46% of pregnant women in Africa and 49% in Asia. Antenatal anaemia is associated with critical risks for both mother and child. Control of maternal anaemia, an important contributor to maternal mortality, is a key WHO nutrition target and component of the third Sustainable Development Goal. Treatment with oral iron is poorly tolerated and requires extended contact with a well-functioning health system; this is difficult to achieve and few women receive full courses of iron. A new intravenous (IV) iron formulation, ferric carboxymaltose, now routinely available and used in developed countries, is safe and provides the opportunity to give high doses of iron in a single rapid infusion. In low and middle-income countries (LMICs), intravenous iron could present a novel, innovative opportunity to rapidly cure moderate and severe antenatal anaemia, thereby improving crucial maternal and neonatal outcomes.

Objectives: To determine whether IV ferric carboxymaltose compared to standard oral iron distributed through routine antenatal services is effective and safe for treatment of moderate and severe antenatal anaemia in pregnancy, in settings where resources are limited, distribution and adherence to antenatal oral iron is challenging, and the burden of maternal anaemia, adverse neonatal outcomes, and infection are high.

Methods: This is an open label two-arm Phase III randomized controlled trial in anaemic pregnant women with the primary maternal outcome of recovery from anaemia and the important secondary neonatal outcome of birth weight, assessed by visits over pregnancy, at birth, and follow-up to 1-month post-partum. The study will have two arms: (a) IV iron plus Intermittent Preventive Treatment in pregnancy (IPTp) and (b) Oral iron – routine care plus IPTp. The IV iron (1000mg for women with weight >50kg, and 20mg/kg for women with weight <50kg) will be given once at baseline while the oral iron (65mg elemental iron twice daily) will be given for 90 days (or the duration of pregnancy, whichever is shorter). Both arms will receive sulfadoxine pyrimethamine (SP) as IPTp according to national guidelines and participants will be followed up after 4 weeks, then weekly (at home to measure Hb) from 30 weeks gestation, as well as clinic visits at 36 weeks gestation, at delivery and at 1 month postpartum. The primary maternal and neonatal outcomes will be the prevalence of anaemia (Hb<11g/dl) at delivery. The planned number of randomized participants is 862 pregnant women (431 per arm). The study has 80% power at a two-sided alpha level of 5% and incorporating a 10% loss to follow up to result to a 10% absolute improvement in anaemia cure (to a prevalence of 46%). The study will be based in antenatal clinics and centres in urban and semi-urban areas of Blantyre and Zomba in Malawi. The trial will be implemented by an international team of Malawian, Australian, UK, and Norwegian experts in clinical trials, obstetrics, anaemia, malaria, and health economics.

Expected Findings and Implications: Oral iron supplementation remains cheap while the drug cost of ferric carboxymaltose is much higher; as such, demonstration of clinical superiority must be accompanied by analysis of potential cost-effectiveness in LMICs, to justify implementation. If our data demonstrate a clear benefit from intravenous iron on maternal haematologic outcomes and

wellbeing, on critical neonatal outcomes such as birth weight and gestation duration, and on infant iron status, they will develop a clinical rationale for implementation of this intervention in routine practice. This will be supported by economic modelling which will enable us to determine a value for money for ferric carboxymaltose, which will enable governments and donors to consider the relative priority of scaling up this intervention compared to other maternal and child interventions. We will use this to identify an appropriate cost point at which this intervention would be considered implementable. Thus, results from this trial could transform the way anaemia is treated in LMICs. As a result, our study may lead to the implementation of ferric carboxymaltose as a treatment for maternal anaemia in the medium term. This could have long term benefits for maternal and child health, ultimately resulting in benefits for maternal and child survival and child development.

Dissemination: The results will be presented at local and international fora, submitted for publication in peer-reviewed scientific journals and reported to COMREC and other relevant ethics committees.

5. SCHEDULE OF ACTIVITIES

Table 1: Details of planned activities per visit

Protocol Activity	Visit 0<br>*Day 0*	Visit 1<br>*Day 0*	Visit 2<br>*Day 28 ±2d*	Visit 3<br>*34 wks ±2d gestation*	Visit 4<br>*36 wks ±2d gestation (pre-delivery)*	Visit 5 - 6<br>*-wk 38 & 40 ±2d gestation*	Visit 7<br>*Delivery ±2d*	Visit 8<br>*Postnatal Day 28 ±2d*	Sick Visit<br>*Un-scheduled*
Location of visit	HF[i]	HF	HF	Home[j]	HF	Home	HF	HF	HF
Pre-screening form completion	X								
Screening		X							
Informed consent process		X							
Medical & obstetric history		X							
Household economic data form			X						
Complete physical examination[a]		X							
Limited physical examination[b]			X		X		X	X	X
Ultrasound scan		X	X		X				
Cost data form completion		X	X	X			X		X
Piper Fatigue Scale (PFS) data form completion		X	X		X			X	X
Cognitive function tests		X	X					X	
Randomize participant		X							

Protocol Activity	Visit 0<br>*Day 0*	Visit 1<br>*Day 0*	Visit 2<br>*Day 28 ±2d*	Visit 3<br>*34 wks ±2d gestation*	Visit 4<br>*36 wks ±2d gestation*<br>*(pre-delivery)*	Visit 5 - 6<br>*-wk 38 & 40 ±2d gestation*	Visit 7<br>*Delivery ±2d*	Visit 8<br>*Postnatal Day 28 ±2d*	Sick Visit<br>*Un-scheduled*
**Location of visit**	**HF[i]**	**HF**	**HF**	**Home[j]**	**HF**	**Home**	**HF**	**HF**	**HF**
**Administer treatment**									
**IV iron**		**X**							
**Oral iron**		**X**							
**Laboratory procedures (Maternal)**									
**Full Blood Count**		**X**	**X**		**X**		**X**	**X**	**X**
**Haemoglobin**		**X**	**X**	**X**	**X**	**X**	**X**	**X**	**X**
**Malaria RDT**		**X**	**X**		**X**		**X**	**X**	**X**
**Malaria microscopy**			**X**		**X**		**X**	**X**	**X**
**Malaria filter paper for PCR**		**X**	**X**		**X**		**X**	**X**	**X**
**Serum for iron markers tests[c]**		**X**	**X**		**X**		**X**	**X**	
**Serum for inflammatory markers tests[d]**		**X**	**X**		**X**		**X**	**X**	**X**
**Phosphate**		**X**	**X**		**X**				
**Treponema Pallidum**			**X**		**X**				

Protocol Activity	Visit 0<br>*Day 0*	Visit 1<br>*Day 0*	Visit 2<br>*Day 28 ±2d*	Visit 3<br>*34 wks ±2d gestation*	Visit 4<br>*36 wks ±2d gestation*<br>*(pre-delivery)*	Visit<br>5 - 6<br>*-wk 38 & 40 ±2d gestation*	Visit 7<br>*Delivery ±2d*	Visit<br>8<br>*Postnatal Day 28 ±2d*	Sick Visit<br>*Un-scheduled*
**Location of visit**	HF[i]	HF	HF	Home[j]	HF	Home	HF	HF	HF
Urinalysis			X		X				
Vaginal swab sample collection[e]			X		X				
Stool sample collection & analysis[f]			X		X				
Placenta histology							X		
Blood culture									X[k]
Breast milk sample for iron markers								X	
**Laboratory procedures (Infant)**									
Full Blood Count									X[l]
Malaria microscopy							X	X	
Serum for iron markers tests[c]							X	X	
Malaria filter paper for PCR							X	X	
**Pregnancy outcome**							X		
**Neonatal weight**							X		

Protocol Activity	Visit 0<br>*Day 0*	Visit 1<br>*Day 0*	Visit 2<br>*Day 28 ±2d*	Visit 3<br>*34 wks ±2d gestation*	Visit 4<br>*36 wks ±2d gestation*<br>*(pre-delivery)*	Visit 5 - 6<br>*-wk 38 & 40 ±2d gestation*	Visit 7<br>*Delivery ±2d*	Visit 8<br>*Postnatal Day 28 ±2d*	Sick Visit<br>*Un-scheduled*
**Location of visit**	**HF[i]**	**HF**	**HF**	**Home[j]**	**HF**	**Home**	**HF**	**HF**	**HF**
**Complete physical examination of neonate[g]**							**X**		
**Limited physical examination of neonate[h]**								**X**	**X[l]**

[a] Complete examination: general appearance, throat, neck, thyroid, musculoskeletal, skin, lymph nodes, extremities, pulses, pulmonary, cardiac, abdominal, and neurological examination

[b] Limited examination: general appearance, brief pulmonary, cardiac, abdominal and neurological examination

[c] Serum ferritin, sTfR

[d] CRP and alpha-1 glycoprotein

[e] Reproductive tract infections: *Chlamydia trachomatis, Neisseria gonorrhoeae, Trichomonas vaginalis,* Bacterial Vaginosis

[f] Stool microscopy and microbiome analysis

[g] Complete examination: weight, length, head circumference, APGAR score, Ballard score, congenital anomaly, and complications at birth

[h] Limited examination: general appearance, brief pulmonary, cardiac, abdominal and neurological examination

[i] Visit will be at health facility

[j] Visit will be at the participant's home

[k] If clinically indicated

[l] If the neonate is unwell

TABLE OF CONTENTS

1. TITLE OF RESEARCH PROPOSAL 14
2. INVESTIGATORS AND INSTITUTIONAL AFFILIATIONS 14
   2.1. INVESTIGATORS 14
      2.1.1. *Co-principal Investigators* 14
      2.1.2. *Co-investigators* 14
      2.1.3. *Trial Statistician* 15
      2.1.4. *Collaborators* 15
   2.2. INSTITUTIONS 15
3. PROTOCOL ABSTRACT 16
4. EXECUTIVE SUMMARY 19
5. SCHEDULE OF ACTIVITIES 21
6. ABBREVIATIONS 28
7. INTRODUCTION 29
   7.1. BACKGROUND 29
   7.2. THE RATIONALE FOR THE RESEARCH PROJECT 37
8. TRIAL OBJECTIVES AND END POINTS 38
   8.1. OBJECTIVES 38
      Broad Objective 38
      Specific Objectives 38
   8.2. ENDPOINTS 38
      Primary Outcome 38
      Secondary Outcomes (Benefit in mother) 38
      Secondary Outcomes (Benefit in the neonate) 39
      Secondary Outcomes (Safety in the mother) 39
      Secondary Outcomes (Cost and cost-effectiveness) 39
9. STUDY DESIGN 40
10. PARTICIPANT INCLUSION AND EXCLUSION CRITERIA 40
11. STUDY SITES 40
12. TRIAL INTERVENTIONS 41
   12.1. ALLOCATION TO TREATMENT 41
   12.2. BREAKING THE BLIND 41
13. STUDY PROCEDURES 41
   13.1. SCREENING – VISIT 0 [DAY 0] 42
   13.2. RECRUITMENT – VISIT 1 [DAY 0] 42
      Blood sampling 42
      Study arm allocation 43
      Study drug administration 43
   13.3. 4-WEEK FOLLOW-UP – VISIT 2 [DAY 28 ±2] 43
      Blood sampling 44
      Reproductive Tract Infections Determination 44
      Urinalysis 44
      Stool Analysis 44
      Maternal quality of life and productivity 44
   13.4. VISIT 3 [34 WEEKS GESTATION ±2 DAYS] 44
   13.5. PRE-DELIVERY FOLLOW-UP – VISIT 4 [36 WEEKS GESTATION ±2 DAYS] 45
      Blood sampling 45
      Reproductive Tract Infections Determination 45
      Stool Analysis 45

Urinalysis 45
Maternal quality of life and productivity 45
13.6. VISIT 5 [38 – 40 WEEKS GESTATION ±2 DAYS] 45
13.7. DELIVERY – VISIT 7-[±2 DAYS] 45
Blood sampling 46
Placental histology 46
Cord blood 46
13.8. POST-DELIVERY – VISIT 8 [28 DAYS POSTPARTUM/POSTNATAL ±2 DAYS] 46
Blood sampling 46
Maternal quality of life and productivity 46
Cognitive function 46
Child anthropometry 47
13.9. UNSCHEDULED SICK VISIT [ANYTIME DURING STUDY FOLLOW-UP] 47
13.10. LABORATORY PROCEDURES 47
Full Blood Count 47
Malaria testing 47
Blood for culture 48
Blood sample preparation for long-term storage for iron and inflammatory marker assays 48
Vaginal and gut microbiome analysis 48
13.11. ARRANGEMENTS FOR ALLOCATING PARTICIPANTS TO TRIAL GROUPS 51

14. ASSESSMENT OF SAFETY 51

14.1. PRECAUTIONS IN DELIVERING FCM 51
14.2. DEFINITIONS OF ADVERSE EVENTS (AE) 53
14.3. RECORDING OF ADVERSE EVENTS 53
The time period for collecting AEs 53
Method of detecting AEs 53
Collection of AE data 53
Assessment of severity 53
Assessment of causality 54
Study Endpoints and symptoms anaemia 54
HIV related disease 54
Lack of efficacy and Disease Progression 54
Abnormal laboratory values 54
Overdose 54
14.4. REPORTING OF SAEs 54
Reporting by the Investigator to the Study Safety Monitor and Sponsor 54
Reporting by the sponsor 55
14.5. DATA MONITORING COMMITTEE 55

15. DATA HANDLING AND RECORD KEEPING 55

15.1. CASE REPORT FORMS (CRFs) 55
15.2. DATABASE LOCK 56

16. STATISTICAL CONSIDERATIONS 56

16.1. SAMPLE SIZE 56
16.2. DATA ANALYSIS 56
Assessment of effectiveness 56
Analysis of adverse events 57
16.3. ANALYSIS POPULATIONS 57
16.4. MISSING DATA 58
16.5. INTERIM ANALYSES AND CRITERIA FOR TERMINATION OF THE TRIAL 58

17. STUDY MANAGEMENT 58

17.1. STUDY MONITORING 58
17.2. DIRECT ACCESS TO SOURCE DATA/DOCUMENTS 59
17.3. QUALITY ASSURANCE 59
17.4. TRAINING OF STAFF 59
17.5. CHANGES TO THE PROTOCOL 59
17.6. FINANCING AND INSURANCE 59
17.7. STUDY DURATION 59
17.8. RECORD-KEEPING AND ARCHIVING 60

17.9. REPORTING AND PUBLICATION OF DATA 60

18. ETHICAL CONSIDERATIONS 60

18.1. ETHICAL REVIEW 60
18.2. ETHICAL CONDUCT OF THE STUDY 60
18.3. INFORMED CONSENT 61
18.4. RISKS TO STUDY PARTICIPANTS 61
Blood sampling 61
IV infusion 61
18.5. BENEFITS FROM PARTICIPATION 61
18.6. SUBJECT DATA PROTECTION 62
18.7. OTHER ETHICAL CONSIDERATIONS 62
Reimbursement of costs 62

19. DISSEMINATION OF RESULTS 62

20. CAPACITY BUILDING 62

22. BUDGET 63

23. REFERENCES 64

24. APPENDIX A - SAFETY AND EFFICACY OF FCM FROM RANDOMISED CONTROLLED TRIALS 67

6. ABBREVIATIONS

ANC	Antenatal Care
CoM	College of Medicine
CRF	Case Report Form
dL	Decilitre
FCM	Ferric Carboxymaltose
Hb	Haemoglobin
HC	Health Centre
HIV	Human Immunodeficiency Virus
ICTRP	International Clinical Trials Registry Platform
IPTp	Intermittent Preventive Treatment in pregnancy
IV	Intravenous
LMICs	Low and Medium-Income Countries
MCATS	Melbourne Clinical and Translational Science Platform
PCR	Polymerase Chain Reaction
QECH	Queen Elizabeth Central Hospital
RCT	Randomised Controlled Trial
RDT	Rapid Diagnostic Test
SC	Sickle Cell
SHC	Sickle Haemoglobin Cell
SP	Sulfadoxine-Pyrimethamine
UK	United Kingdom
US	United States
WHO	World Health Organisation
ZCH	Zomba Central Hospital

7. INTRODUCTION

7.1. Background

Anaemia during pregnancy remains a critical global health problem

Almost 40% of pregnant women worldwide are anaemic, including 46% of pregnant women in Africa and 49% in Asia, compared with 17% in North America [1]. Anaemia in pregnancy is associated with critical risks for both mother (e.g. life-threatening complications of post-partum haemorrhage) and child (especially prematurity and low birth weight [2], which are associated with increased risk of mortality, and reduced iron stores in infancy with increased risk of subsequent anaemia and impaired development) [3]. Recent data confirm that even in developed countries such as the UK, anaemia in pregnancy is associated with an adjusted 3.6 odds ratio for increased maternal mortality [4], and in the US is associated with impaired long-term child cognitive development [5]. Control of anaemia in women is, therefore, a key 2025 global nutrition target [6].

Low birth weight and pre-term birth have critical implications for mother and baby

Worldwide, 15%-20% of births worldwide (>20 million annually) (including 13% in sub-Saharan Africa) are low birth weight, while each year, over 1 million children die from complications of pre-term birth. Although the underlying determinants for low birth weight and prematurity are multifactorial, prevention and treatment of antenatal anaemia with iron appears a crucial intervention and considered an essential component of the 2025 WHO nutrition target of a 30% reduction in low birth weight.

Iron can benefit antenatal anaemia

Benefits and safety of iron supplementation during pregnancy in the sub-Saharan African context were most clearly exemplified in a recent placebo-controlled double-blind randomized single centre field trial in Kenyan pregnant women [7]. In this trial, oral iron supplementation increased birth weight by 150g and reduced the risk of low birth weight by 58%, lengthened gestation duration by 3.4 days and reduced the risk of premature birth by 7% and reduced the risk of maternal anaemia from 50.4% to 22%. Reassuringly, in this highly malaria-endemic setting, there was no evidence of an increase in clinical malaria, placental malaria or parasitaemia among women randomized to iron. This trial is one of the most recent examples of an extensive literature of trials evaluating oral iron in pregnancy. Systematic reviews of these studies confirm likely benefits from iron on maternal outcomes including anaemia (70% reduction), and trends towards favourable infant outcomes including increased birth weight and extended gestation duration [8]. However, the success of the Kenyan trial was likely predicated on the extremely high adherence demanded of the participants (100%), achieved by daily visits by fieldworkers. This emphasizes the importance of delivery of a full course of iron supplementation on birth outcomes.

Uptake and adherence to oral iron therapy during pregnancy is inadequate in the field

Global recommendations for management of anaemia in pregnancy in LMICs currently advise that women be treated with high dose daily oral iron (120 mg of elemental iron) supplementation for 3 months [9, 10]. However, such high doses of iron are often poorly tolerated due to significant gastrointestinal adverse effects [11] limiting adherence to this vital intervention. Moreover, delivery of iron during pregnancy requires ongoing contacts between the mother and primary health system. For example, in Malawi, fewer than 25% of pregnant women receive a full course of iron (Clophat Baleti, Personal Communication). Across Africa, only very few women receive the recommended course of antenatal iron and may present for their initial visit far into the second trimester. This limits opportunities to treat antenatal anaemia, exposing women and their babies to its consequences. Furthermore, even when delivered, oral iron frequently fails to correct anaemia in routine practice. For example, in our recent study among pregnant women in The Gambia, we provided iron to all women with Hb<10g/dL at week 20 of gestation; by week 30, 61% still had Hb<10g/dL. [12]

Ferric carboxymaltose as a new intravenous iron preparation recommended widely

Over the past decade, there have been dramatic improvements in the safety and convenience of parenteral (intravenous) iron therapies. Older forms of intravenous iron were associated with serious allergic reactions (iron dextran), required prolonged administration periods (iron polymaltose), or could only be administered in small doses (200-300mg) requiring frequent infusions (iron sucrose).

However, new parenteral intravenous agents have completely changed the landscape of iron therapy. The most established therapy is Ferric carboxymaltose (FCM), which overcomes many limitations of previous parenteral iron treatments. Ferric carboxymaltose is a new and differently engineered iron formulation. It comprises a colloidal complex of a polynuclear iron (III) oxyhydroxide core with carboxymaltose ligands. [13] Following administration, the complex is degraded into simple endogenous molecules (glucose, maltose, maltotriose, maltotetraose and iron), and iron is rapidly taken up by iron transport (transferrin) and storage (ferritin) proteins. Due to this property, FCM can be administered in a short period of time (15 min) and at large doses (up to 1000 mg in EU) [14]. Unlike iron dextran, the risk of anaphylaxis has been largely overcome, although hypersensitivity reactions may still occur. Unlike iron polymaltose, the infusion can be administered rapidly. Unlike iron sucrose, the maximum dose of iron is high enough that a single visit is sufficient for a high (usually total) dose of iron to be delivered. As currently licensed, a dose of up to 1000mg of iron (or 15-20mg/kg body weight), diluted in 250mL saline, may be administered over a 15-minute infusion. In most cases, this enables a total dose iron replacement to be achieved in a single visit. FCM has now revolutionised the treatment of iron deficiency anaemia in high-income country settings and is widely used in ambulant/ outpatient settings, emergency departments, in non-specialist clinical wards, preoperative clinics and even in remote settings. FCM has been available in Europe since its approval in 2007 and in the USA since 2009; it is currently marketed in over 50 countries. [14]

Safety and efficacy evidence of Ferric carboxymaltose in various patient populations

Rognoni et al 2015 undertook a systematic review and network meta-analysis of FCM vs both oral iron and other parenteral formulations. [15] The authors identified 21 RCTs comparing iron treatments in anaemic (or non-anaemic) patients requiring therapies for ID published between 2003-2014, although not all of these included FCM. The trials reporting safety and efficacy of FCM are summarised in Appendix A.

Regarding safety, the review authors found that overall, FCM was well tolerated and associated with a minimal risk of AEs [15]. In trials in which Adverse Events (AEs) occurred in a larger proportion of FCM-

treated patients than those receiving oral iron or placebo, the difference seldom reached the statistical significance level. Several studies found that patients who were given FCM experienced fewer drug-related gastrointestinal disorders (e.g. constipation, diarrhoea) than those treated with oral iron. However, patients treated with ferric carboxymaltose iron were more likely to develop rash, dermatitis and pruritus that generally resolved within a few minutes of the infusion. Other frequent AEs associated with ferric carboxymaltose administration were fatigue, headache and dizziness. No true cases of anaphylaxis and no deaths occurred in patients receiving FCM.

Regarding efficacy, the authors undertook a network meta-analysis to compare the efficacy of FCM as compared with oral iron and other parenteral formulations. The authors found that in terms of improving haemoglobin concentrations, FCM was superior to placebo (delta 2.1; 95 % CI 1.2–3.0), oral iron (delta 0.8; 95 % CI 0.6–0.9), intravenous ferric gluconate (delta 0.6; 95 % CI 0.2–0.9), and iron sucrose (but not reaching statistical significance). The authors found that for improvements in ferritin, FCM was superior to oral iron (delta 172.76; 95 % CI 66.7–234.4) and similar to other parenteral formulations (iron sucrose and gluconate) [15].

A previous systematic review and meta-analysis published in September 2011 evaluated published and unpublished studies of efficacy and safety of ferric carboxymaltose [16]. The review evaluated clinical trial reports and published studies comparing FCM with either other active comparators or with placebo. The authors identified fourteen studies, in which 2,348 patients had been assigned to FCM, compared with, in the control arms, 762 to placebo, 832 to oral iron and 384 to intravenous iron sucrose. Compared with oral iron, ferric carboxymaltose produced superior improvements in haemoglobin, ferritin and transferrin saturation, and was associated with a greater chance of resolution of anaemia or clinically significant improvement in haemoglobin. Maximum responses were usually achieved by 4-6 weeks. Adverse events were also evaluated.

The table below, from this manuscript, shows the risk of withdrawals and adverse events in patients receiving ferric carboxymaltose compared with those randomized to receive either control or another iron intervention. As compared with either oral iron or placebo, participants receiving FCM were slightly less likely to withdraw from the study, while there was no difference in the number of participants experiencing at least one adverse event, death, or serious adverse events between subjects receiving FCM and those receiving other interventions. However, there was a slight increase in the risk of hypotension in participants receiving FCM when compared specifically with oral iron (but not when compared with receipt of other intravenous interventions).

Table 2: Risk of Withdrawals and Adverse Events in patients receiving IV Ferric Carboxymaltose

Outcome	Comparator	Number of Trials	Number of Patients	Percent with Ferric carboxymaltose	Percent with Control	RB or RR 95% CI	NNTp 95% CI
**Withdrawals**							
All cause	All	10	3835	6.4	7.5	0.8 (0.6 to 0.9)	93 (37 to 180)
	Oral iron	6	1898	8.1	9.3	0.8 (0.6 to 1.03)	not calculated
Adverse event	All	8	3319	1.0	1.6	0.6 (0.3 to 1.02)	not calculated
	Oral iron	6	1898	1.5	1.9	0.7 (0.3 to 1.4)	not calculated
Lack of efficacy	All	7	2967	0.6	0.8	0.8 (0.4 to 1.7)	not calculated
	Oral iron	5	1546	1.1	1.2	0.8 (0.4 to 1.9)	not calculated
**Adverse events**							**NNH 95% CI**
At least 1 AE	All	8	2951	41	38	1.1 (1.0 to 1.2)	not calculated
	Oral iron	5	1539	48	53	1.0 (0.9 to 1.1)	not calculated
Death	All	10	3762	0.53	0.3	1.3 (0.5 to 3.4)	not calculated
	Oral iron	6	1891	0.38	0.0	1.7 (0.4 to 6.6)	not calculated
Serious AE	All	8	3303	2.5	2.3	1.0 (0.6 to 1.5)	not calculated
	Oral iron	6	1891	3.1	2.3	1.3 (0.7 to 2.2)	not calculated
Hypotension	All	6	2694	1.5	1.0	1.5 (0.8 to 2.7)	not calculated
	Oral iron	4	1339	1.3	0.0	4.7 (1.1 to 21)	79 (44 to 390)

Note: NNT=Number Needed to Treat; NNH=Number Needed to Harm

The table below, adapted from this meta-analysis, compares the incidence of various organ-specific side effects associated with the administration of FCM when compared with 1) oral iron as the control, and 2) IV saline as the control.

Table 3: Organ-specific side effects of ferric carboxymaltose compared with oral iron or IV saline

Outcome	Number of Trials	Number of Patients	Percent with Ferric carboxymaltose	Percent with Control	RB or RR 95% CI	NNTp/H 95% CI
			**Comparison with oral iron**			
**Body system and preferred term**						
GI disorder	5	1539	13	32	0.44 (0.36 to 0.54)	5.4 (4.4 to 7.1)
General, administrative site	5	1539	11	4	2.8 (1.9 to 4.2)	**15 (11 to 24)**
Infection, infestation	5	1539	14	12	1.2 (0.9 to 1.6)	not calculated
Metabolism, nutrition, investigation	4	1195	11	5	2.2 (1.4 to 3.4)	**17 (11 to 33)**
Nervous system	5	1539	10	9	1.3 (0.9 to 1.7)	not calculated
**Specific adverse events**						
Constipation	4	1339	3	13	0.3 (0.2 to 0.4)	9.8 (7.6 to 14)
Diarrhoea	3	906	2	5	0.5 (0.2 to 0.9)	33 (18 to 230)
Nausea/vomiting	3	906	3	10	0.4 (0.2 to 0.6)	14 (9.5 to 27)
Headache	5	1539	7	7	1.2 (0.8 to 1.7)	not calculated
			**Comparison with IV saline**			
**Body system and preferred term**						
GI disorder	2	1577	8	5	1.6 (1.1 to 2.4)	**34 (19 to 210)**
General, administrative site	2	1577	6	2	2.5 (1.5 to 4.3)	**25 (17 to 49)**
Infection, infestation	2	1577	9	6	1.1 (0.8 to 1.6)	not calculated
Nervous system	2	1577	8	6	1.2 (0.9 to 1.8)	not calculated
Respiratory system	2	1577	2	2	0.8 (0.4 to 1.5)	not calculated

Note NNTp in normal text, NNH when bold

As shown in the table above, compared with oral iron, FCM was less likely to cause constipation, diarrhoea, nausea/ vomiting, and gastrointestinal disorders in general, but was more likely to cause adverse events at the administration site. When FCM was compared to intravenous saline, FCM was associated with an increased risk of ‘GI disorder’ and local site reactions, but not with adverse effects in other organ systems.

Numerous randomised controlled trials have compared FCM with either oral iron or other forms of parenteral iron for treatment of IDA in a variety of clinical situations, including pregnancy,[17] post-partum,[18] and in a variety of clinical conditions including inflammatory bowel disease,[19-25] chronic renal failure,[26, 27] and heart failure.[28] It has also been studied in the preoperative context for optimising pre-operative haemoglobin concentrations as a component of patient blood management.[29-34] Here, we will summarise recent reviews of FCM that address key safety and efficacy endpoints.

Safety of Ferric carboxymaltose

Several studies, including one randomised controlled trial, have evaluated the efficacy and safety of FCM in pregnancy. These studies are summarised here:

Breymann et al:[17] FER-ASAP was the pivotal, Phase IIIb, open-label randomised controlled trial comparing FCM with oral iron (ferrous sulfate) for treatment of iron deficiency anaemia in pregnancy. This multicentre study was set across 8 high and middle-income countries and randomised 252 women in their second or third trimester of pregnancy to FCM (1000-1500 mg iron) or FS (200 mg iron/day for 12 weeks). The trial was designed to identify a more rapid improvement in haemoglobin concentration by 3 weeks post-infusion compared with baseline. The study showed an advantage of Hb with FCM compared with FS at 6 and 3 weeks (although the difference was not significant at 3 weeks). According to the SF-36 health survey, FCM treatment led to significant, clinically relevant improvements over FS in vitality and social functioning prior to delivery.

Safety: The incidence of Treatment-Emergent Adverse Events (TEAEs) was similar between the treatment arms: in the FCM group, 60 women (49%) experienced 165 TEAEs; in the FS group, 50 women (40%) experienced 105 TEAEs. The majority of events were mild in intensity (see table 5 below).

Table 4: Incidence of Treatment-Emergent Adverse Events (TEAEs)

Treatment-related TEAE severity, number of patients (%)	Ferric carboxymaltose (n=123)	Ferrous sulfate (n=124)
Total	60 (49)	50 (40)
Mild	43 (72)	28 (56)
Moderate	17 (28)	20 (40)
Severe	0 (0)	2 (4)

TEAE=treatment-emergent adverse event.

Overall, the most common TEAEs were nausea (6%), headache (5%) and dyspepsia (4%), and the most common TEAEs according to system organ class were “pregnancy, puerperium and perinatal conditions” in the FCM group [32 events in 26 women (21%)] and “gastrointestinal disorders” in the FS group [42 events in 25 women (20%)]. The most common treatment-related TEAEs were headache with FCM [experienced by 4 women (3%)] and nausea with FS [in 6 women (5%)], and markedly higher rates of gastrointestinal disorders were reported with FS treatment (in 16 women) compared with FCM treatment (3 women).

Table 5: TEAEs in FCM vs FS treatment arms

Treatment-related TEAE,[a] number of patients (%)	Ferric carboxymaltose (n=123)	Ferrous sulfate (n=124)
Total	14 (11)	19 (15)
Nervous system disorders	7 (6)	1 (1)
Headache	4 (3)	1 (1)
Dizziness	3 (2)	0 (0)
Dysgeusia	2 (2)	0 (0)
General disorders and administration-site conditions	4 (3)	0 (0)
Vascular disorders	2 (2)	0 (0)
Gastrointestinal disorders	3 (2)	16 (13)
Nausea	2 (2)	6 (5)
Vomiting	0 (0)	2 (2)
Constipation	0 (0)	3 (2)
Diarrhea	0 (0)	4 (3)
Abdominal pain upper	0 (0)	5 (4)
Dyspepsia	0 (0)	3 (2)

[a]According to physician's assessment; a single patient could appear in multiple classes. Percentages calculated according to treatment group.
MedDRA=Medical Dictionary for Regulatory Activities (version 16.1), TEAE=treatment-emergent adverse event.

Serious TEAEs occurred in 23 women treated with FCM (26 events) and in 10 women treated with FS (11 events); all were single events, except for "failed trial of labor", "fetal distress syndrome", "premature delivery", "premature rupture of membranes" and "threatened labour", each of which occurred in two women treated with FCM, and "premature labour", which occurred in three women treated with FS (pre-eclampsia was also reported to have occurred twice in one woman treated with FS). One serious TEAE, "bronchospasm", which was treatment-related, led to discontinuation of FCM; this event was of moderate intensity and resolved on the same day after withdrawal of study treatment. Seven women discontinued treatment with FS because of gastrointestinal TEAEs (n=5), syncope (n=1) and rash (n=1). No hypophosphatemia TEAEs were reported during this study. Eleven women (FCM, n=10; ferrous sulfate, n=1) recorded phosphate levels below the lower normal range threshold [0.6 mmol/L (2 mg/dL)]; these decreases were observed at week 3 and recovered in all the women to within the normal range by the end of the study.

All other trials have been single arm prospective trials or retrospective comparative studies.

Froessler et al undertook a prospective observational study in 65 anaemic pregnant women in South Australia, Australia, receiving ferric carboxymaltose (up to 15 mg/kg) between 24 and 40 weeks gestation (median 35 weeks).[35] The authors observed increases in haemoglobin concentrations at 3, 6 and up to 8 weeks post-infusion (see table 6 below), although in women with the most severe anaemia at baseline, improvements in haemoglobin were not sustained by 2 months post-partum.

Table 6: Increase in haemoglobin concentration at 3, 6 and 8 weeks post-infusion

	Gestational age at entry	Pre-infusion	3 weeks post infusion	6 weeks post infusion	8 weeks post infusion (post-partum)
Mild ≥95 g/L	34 (4)	102.1 (1.0) n = 31	108.3 (3.9)* n = 28	120.6 (2.9)* n = 18	113.1 (4.2)* n = 11
Moderate 90-94 g/L	36 (2)	92.6 (0.4) n = 14	105.8 (3.0)* n = 13	108.4 (3.8)* n = 5	92.7 (12.4) n = 3
Severe <90 g/L	34 (3)	83.7 (0.9) 20	100.2 (3.3)* n = 17	110.0 (8.1)* n = 5	93.3 (8.1) n = 4

Data are presented as means (SEM). *p < 0.01 compared to pre-infusion haemoglobin levels.

Safety: No serious adverse effects were recorded in any of the 65 women receiving an infusion. Minor side effects occurred in 13 (20%) patients. One patient required medication with Metoclopramide for nausea and vomiting. All other adverse events were self-limiting. Fetal heart rate monitoring did not indicate a drug-related adverse effect on the fetal heart pattern. Red blood cell transfusions were required by 3 women (4.6%) in the study cohort, all of whom had a significant peripartum haemorrhage. Adverse effects from the cohort are summarised in the ~~following~~ table 7:

Table 7: Number of women experiencing a drug related adverse event following infusion with ferric carboxymaltose (total number of women infused n= 65)

Adverse event	n (%)
Any adverse event	13 (20)
Local (injection site irritation)	
Slight burning sensation	5 (8)
Systemic	
Hypotension	1 (1.5)
Headache	4 (6)
Nausea/Vomiting	1 (1.5)
Pruritus	2 (3)

Zeba et al undertook a prospective cohort study in 260 pregnant women between 28 and 36 weeks gestation with haemoglobin <10g/dL in a private obstetric clinic in Faridpur, Bangladesh [36]. All women in this study group were treated with intravenous ferric carboxymaltose as a single dose (500-1000mg) in an infusion time of 15-20minutes. *Safety:* No serious adverse events occurred, with 13% experienced minor adverse effects such as injection site irritation, headache, nausea or vomiting etc. *Efficacy:* Compared with the Hb (mean 8.9g/dl) level before infusion, the Hb (mean 10.53g/dl) level after infusion had significantly (p<0.001) increased.

Mishra et al. undertook a single arm prospective cohort trial of FCM in Ahmedabad, India in 108 participants [37]. Women received up to 1500mg FCM in total. *Safety*: Three women experienced local reactions e.g. itching and irritation at the infusion site, while another 5 women reported systemic reactions of giddiness, headache and nausea. *Efficacy*: FCM produced an average increase of 2.1g/dL haemoglobin in 3 weeks.

Christoph P et al. undertook a retrospective analysis of 206 pregnant women who had received either FCM or iron sucrose during pregnancy in order to assess maternal safety and tolerability [38]. The authors observed that Mild adverse events occurred in 7.8% for ferric carboxymaltose and in 10.7% for iron sucrose. The authors also concluded that 'no sign for a negative effect on the fetus of iron infusion could be detected.'

Use of Ferric carboxymaltose in pregnancy

Based on these studies, licensing by international regulators including the FDA, EMA and TGA, and clinical experience, FCM has rapidly become an established treatment for anaemia in pregnancy in developed countries. For example, British Committee for Standards in Haematology 2011 guidelines recommend that parenteral iron be considered for women from the second trimester onwards with iron deficiency anaemia who fail or are intolerant of iron. [39] Many UK health services (NHS Trusts) recommend Ferric Carboxymaltose as second-line therapy for iron deficiency anaemia in pregnancy. For example, the Mid Essex NHS Trust recommends FCM for women with persistent anaemia despite 4 weeks oral iron, or with haemoglobin <80g/L on any occasion, or whom are intolerant of or non-compliant with oral iron; the guideline also recommends FCM as first-line therapy in all women who are anaemic beyond 34 weeks pregnancy. [40] In many cases, use is limited not by concerns of efficacy or safety, but by cost and health economic implications.

A 2012 review suggested that as FCM and other novel parenteral iron agents represent a 'milestone' in intravenous iron therapy, they should be considered as increasingly first-line for treatment of anaemia in pregnancy. [41] Here, the authors suggested that women with Hb<10g/dL should probably be given IV iron if they have a ferritin <30mg/L, and among women with higher ferritin levels, this represents an area of research in establishing whether IV or oral iron should be given.

However, since the publication of the FER-ASAP trial, use of IV FCM in pregnancy is becoming increasingly considered as the first line. For example, a recent influential 'How I Treat' review article in the leading haematology journal 'Blood' formalized the new position of parenteral iron as first-line therapy for iron deficiency anaemia in pregnancy. [42] Here, the authors suggest that **all** cases of anaemia in the second or third trimester receive intravenous iron.

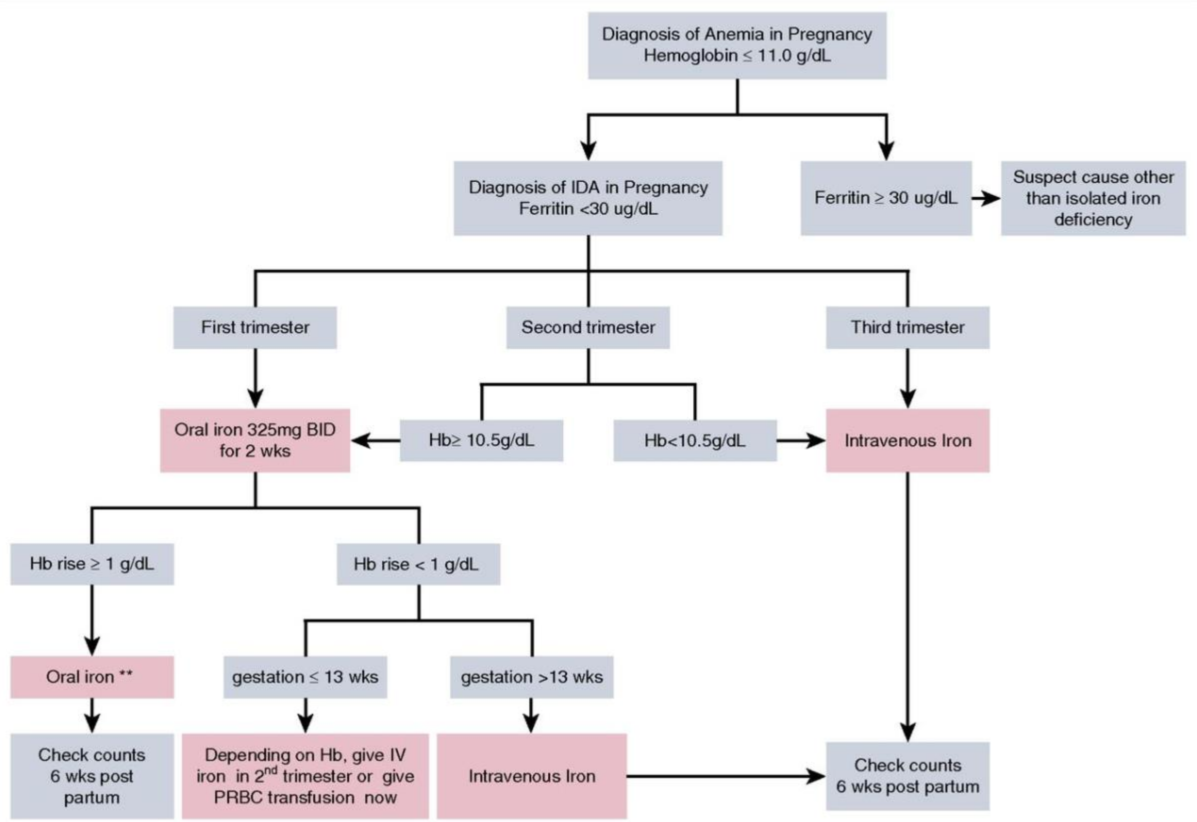

Figure 1: Decision tree on parenteral iron as first-line therapy for iron deficiency anaemia in pregnancy *[From: Achebe MM et al. How I treat anaemia in pregnancy: iron, cobalamin, and folate. Blood 2017;129:940-9].*

Thus, in high-income countries, FCM is now mainstream, routine therapy for iron deficiency anaemia in pregnancy. Since FCM is already licensed for the treatment of anaemia in pregnancy beyond the first trimester, using European MHRA guidelines, the REVAMP trial would be categorised as Type A = No higher than the risk of standard medical care.

Use of Intravenous Iron/ FCM in sub-Saharan Africa

There is clinical and research experience in the use of FCM in sub-Saharan Africa. FCM is now available in South Africa. A large randomised clinical trial comparing FCM to oral iron for treatment of post-partum anaemia is in progress in Tanzania (NCT02541708). Likewise, there is clinical experience using iron sucrose to treat anaemia in Malawi. Iron sucrose is included as a therapy for management of anaemia in 2015 Malawi Standard Treatment Guidelines (p26).

7.2. THE RATIONALE FOR THE RESEARCH PROJECT

A treatment, which could rapidly restore maternal iron stores to treat maternal anaemia, could optimise maternal and infant outcomes and be of immense value in LMICs.

Modern formulations of intravenous iron are safe and enable total dose iron infusion. Ferric carboxymaltose (Ferinject/Injectafer) is approved in Europe, Australia and the United States for treatment of iron deficiency anaemia in men and women of all ages including pregnant women. Following administration, iron is released from the carboxydrate shell and rapidly taken up by iron transport (transferrin) and storage (ferritin) proteins. This drug is not associated with risks of anaphylaxis; low-grade effects such as joint pains, flushing and biochemical hypophosphataemia are commonly observed but well-tolerated [45]. A ferric carboxymaltose 1000mg elemental iron dose can be administered in 250mL over 15 minutes, without test doses or safety monitoring.

Ferric carboxymaltose is approved for use in pregnancy in the second and third trimesters, and its use is increasingly recommended in complex patients. For example, British guidelines for management of antenatal iron deficiency indicate that parenteral iron should be considered in the second trimester onwards in women who are iron deficient who are intolerant to or fail to respond to iron [39]. Ferric carboxymaltose is increasingly recommended as first-line therapy for all pregnant women with moderate or severe anaemia [42].

Ferric carboxymaltose is superior to oral iron for anaemia in pregnancy in developed settings. Efficacy and safety for treatment of anaemia in pregnancy of IV ferric carboxymaltose compared with oral iron has been demonstrated in a recent open-label randomized international multicenter Phase III trial [46] which recruited 252 pregnant women with iron deficiency anaemia across 7 developed countries and randomized them to IV FCM (dose based on degree of anaemia and weight) or very high dose oral iron (ferrous sulfate, 100mg elemental iron twice daily). Pregnant women treated with FCM were twice as likely to correct their anaemia (odds ratio: 2.06, P=0.031), and the median time to achieving this correction was shorter (3.4 vs. 4.3 weeks). FCM also improved iron stores and maternal vitality and social functioning prior to delivery compared with oral. In this trial (undertaken in high-income countries, and with high dose oral iron as a comparator), effects on birth outcomes were not observed. Oral iron caused more gastrointestinal effects compared to intravenous. Low PO4 was seen in 10/126 patients receiving IV iron

compared to no reports in patients receiving oral iron, but clinically symptomatic hypophosphataemia was not seen.

In LMICs, where adherence and efficacy of oral iron are potentially inadequate, intravenous ferric carboxymaltose presents a novel, innovative opportunity to rapidly correct moderate and severe antenatal anaemia in a single dose, improving maternal and neonatal outcomes.

8. TRIAL OBJECTIVES AND END POINTS

8.1. Objectives

Broad Objective

To determine whether FCM up to 1000mg given once during the second trimester is superior to oral iron as administered by routine health services during pregnancy in the reduction of maternal anaemia at term.

Specific Objectives

1. To determine the effectiveness of FCM on maternal anaemia at delivery, when compared with routinely delivered oral iron.
2. To determine the effectiveness of FCM compared to routinely delivered oral iron on critical neonatal outcomes including birth weight (low birth weight), gestation duration (prematurity), small for gestational age and other adverse birth outcomes.
3. To determine the effectiveness of FCM compared to routinely delivered oral iron on maternal outcomes including haemoglobin concentration and iron deficiency (measured through iron biomarkers), ~~and~~ maternal quality of life and maternal cognitive function.
4. To determine the safety of IV iron compared to oral iron on the prevalence of all-cause sick visits, serious maternal adverse events, clinical malaria, bacteraemia, placental malaria, malaria parasitaemia, hypophosphatemia and pathogenic vaginal bacterial flora during pregnancy.
5. To determine immune and molecular aetiologies that may be related to anaemia, iron deficiency and infection in pregnancy.
6. To determine the cost-effectiveness of IV iron compared to oral iron measured through absolute and incremental health provider and patient costs.

8.2. ENDPOINTS

In order to make the case for implementation of this more complicated intervention, it is essential to demonstrate the superiority of IV iron for the health of both the mother and new-born. Likewise, it is crucial the intervention be shown to be safe, with specific regard to the risk of infection.

Primary Outcome

Prevalence of maternal anaemia (Hb <11.0g/dl) at delivery.

Secondary Outcomes (Benefit in mother)

1. Maternal quality of life, using the Piper Fatigue Scale (PFS) at 36 weeks gestation.
2. Maternal cognitive function at 4 weeks post randomisation and 4 weeks postpartum.
3. Maternal Hb at 4 weeks post randomisation and at delivery.
4. Maternal iron deficiency (defined by i) ferritin<15mg/L, and ii) sTfR/Ferritin index – assay dependent cut-off) at 4 weeks post randomisation and 36 weeks gestation.
5. Maternal anaemia (Hb<11g/dL) and haemoglobin concentration at 4 weeks postpartum

6. Maternal iron deficiency and iron indices at 4 weeks postpartum.
7. The incidence of severe anaemia requiring a blood transfusion
8. The incidence of severe medical events: haemorrhage, sepsis, shock, need for transfusion, ICU or prolonged admission, or mortality, both individually and as a composite outcome.

Secondary Outcomes (Benefit in the neonate)

9. Birth weight – measured in grams within 24 hours after birth
10. Placental weight and its ratio to birth weight
11. Gestational-age adjusted birth weight
12. Small for gestation age
13. Low birth weight – defined as birth weight less than 2500 g (up to and including 2499g)
14. Abortion – defined as a pregnancy loss before 28 completed weeks of gestation
15. Stillbirth – defined as the birth of a baby showing no signs of life after 28 weeks of gestation (>28 weeks)
16. Gestation duration (in days)
17. Premature births – defined as the neonate born prior to 37 completed weeks of gestation (including 36weeks and 6 days).
18. Cord venous blood Hb, ferritin, and sTfR-ferritin index levels.
19. Infant haemoglobin concentration and iron indices 1 month after delivery.

Secondary Outcomes (Safety in the mother)

20. Adverse events related to administration of the intervention
21. The incidence of all-cause sick visits to the clinic
22. The incidence of diarrhoea sick visits to the clinic
23. The incidence of clinical malaria-specific sick visits to the clinic
24. The incidence of placental malaria at delivery
25. The incidence of peripheral parasitaemia by 36 weeks of gestation
26. The incidence of cord blood parasitaemia at delivery
27. Prevalence of malaria parasitaemia at 4 weeks post randomisation and at 36 weeks' gestation
28. Prevalence of bacteraemia at 4 weeks post randomisation and at 36 weeks' gestation
29. Prevalence of hypophosphatemia (clinical and biochemical) at 4 weeks post randomisation and at 36 weeks' gestation.
30. Prevalence of maternal inflammation, defined by elevated C-reactive protein and alpha-1 glycoprotein levels, at 4 weeks post randomisation and at 36 weeks gestation.
31. Prevalence of pathogenic vaginal bacterial flora and gut microbiome at 4 weeks post randomisation and at 36 weeks' gestation.

Secondary Outcomes (Cost and cost-effectiveness)

32. Health systems costs of providing the treatment and follow-up for the intervention and comparator
33. Direct and indirect patient costs of receiving the intervention and the comparator
34. Indirect patient costs of disease (productivity consequences)
35. Incremental cost-effectiveness of FCM compared to oral iron given to pregnant women in Malawi.

9. STUDY DESIGN

This will be a Phase III open-label 2-arm parallel group randomized controlled trial recruiting pregnant women in their second trimester and following them up until 1 month postpartum. Participants will be recruited from antenatal clinics in urban and semi-urban areas and randomized to receive IV iron once or oral iron for 90 days or the duration of pregnancy, whichever is shorter. An open-label (rather than blinded) trial was chosen as it was deemed that it would be ethically and operationally challenging to provide placebo infusions to women during pregnancy. In particular, ferric carboxymaltose is black in colour, and thus would require either use of opaque tubing, a curtain and veil to conceal the tubing or a blindfold for the participant – none of which are likely to be feasible in practice. The recent pivotal RCT of intravenous versus oral iron in pregnancy employed an open-label design.

10. PARTICIPANT INCLUSION AND EXCLUSION CRITERIA

Participants meeting the following criteria will be included in the trial:

1. Confirmed singleton pregnancy at 13-26 weeks gestation
2. Moderate to severe anaemia not requiring an immediate blood transfusion (Hb <10g/dl)
3. Negative malaria parasitaemia
4. Resident in the study catchment area of Blantyre and Zomba district
5. Plan to deliver at a health facility
6. Written informed consent (including assent if <18 years old)

Participants with the following criteria will be excluded:

1. Hypersensitivity to any of the study drugs
2. Clinical symptoms of malaria or bacterial infection
3. Any condition requiring hospitalization or serious concomitant illness
4. Chronic illness that may adversely affect foetal growth and viability
5. Clinically low haemoglobin requiring a blood transfusion (usually Hb <5g/dl)
6. Pre-eclampsia

11. STUDY SITES

The is a multicentre study to be carried out in the following sites:

Zomba Central Hospital (ZCH), in southern Malawi will be the main coordinating site, with 3 satellite health centres (within a 5-minute drive distance) where participants will be screened. This is a well-established clinical research site with all resources available to recruit eligible participants, prepare and administer the study drugs, monitor safety, treat adverse effects, and measure trial outcomes (22,230 women above 18 years, 4173 births per year, 13% with Hb <10g/dL respectively). This site recently participated in a large multicentre trial of antenatal anti-malarial treatment (IPTp), demonstrating the capability to undertake such studies [47].

Our second site will be in Blantyre (the hub of health research in Malawi) and will involve three major health centres (HCs) namely Limbe HC, Bangwe HC, and Ndirande HC which are all less than 10 kilometres apart. One of the health centres (Limbe HC) will be used as the recruitment site with the other two as

satellite screening sites. Between them, they have 1113 new ANC attendees and 454 deliveries per month. Some of the laboratory analyses will be done on site e.g. haemoglobin and malaria tests while other lab tests will be done at the CoM Blantyre campus. The laboratory and pharmacy facilities at the CoM Blantyre campus have previously supported large trials. In Malawi, the average gestation at first ANC visit is 22 weeks, with 61% presenting by 24 weeks.

12. TRIAL INTERVENTIONS

This is an open-label trial and the intervention group will receive an IV iron given once at recruitment while the control group will receive oral iron through the routine health care system, given over 90 days or for the duration of the pregnancy, whichever comes first. Participants will be assigned to receive the following:

1. **IV iron treatment course**: intravenous ferric carboxymaltose 1000mg (weight >50kg) or 20mg/kg (weight <50kg) given over 15min once at recruitment (Day 0);

OR

2. **Oral iron treatment course**: oral iron- 200mg ferrous sulphate (approx. 65mg elemental iron) twice daily for 90 days (reaching a total dose of approx. 11.7g) or the remaining duration of pregnancy, whichever is shorter. Note that previous studies have estimated that iron absorption for ferrous sulfate in pregnancy approaches 20% from the second to third trimester [48], and thus this dose should ultimately be *higher* than the IV iron dose if adherence is 100%.

Both groups will receive IPTp with SP, 1500mg sulfadoxine and 75mg pyrimethamine (3 fixed tablets of SP strength at 500mg/25mg) given at least 3 times during pregnancy, at least 4 weeks apart. As part of safety assessment, the IPTp SP course at 4 weeks post-randomization will be directly observed by study staff. Otherwise, subsequent IPTp treatment courses will be administered under real-life programmatic conditions.

The oral iron will be given under real-life health service delivery conditions where the participant is given 3 months oral iron at presentation to the antenatal clinic. This strategy is being employed as it is a key hypothesis in this trial that there is a reduced effect of oral iron on maternal anaemia due to poor adherence to the full course of treatment. Hence, this an effectiveness trial.

Each participant will additionally receive an insecticide-treated bed nets as per standard national guidelines through the health facility.

12.1. Allocation to Treatment

The investigator's knowledge of treatment must not influence the decision to recruit a particular participant or affect the order in which participants are recruited.

12.2. Breaking the Blind

The trial is open label. However, the laboratory scientists will be blinded to the treatment of the participant in conducting the laboratory assays.

13. STUDY PROCEDURES

The recruitment and follow-up schedule is shown in Figure 2.

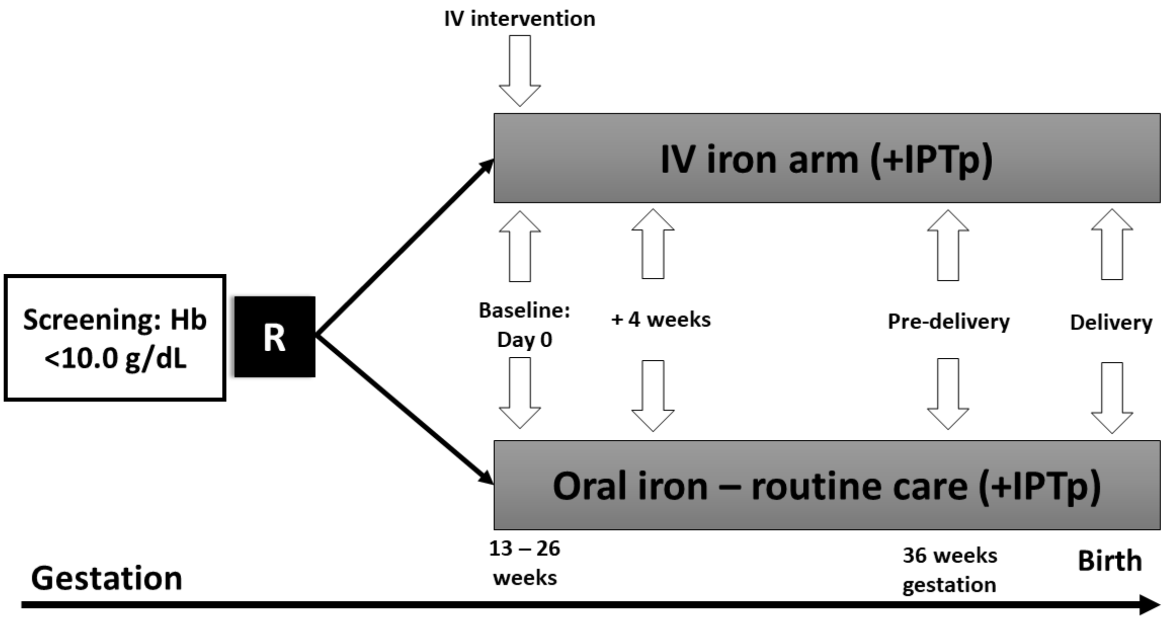

Figure 2: Study Schema

13.1.Screening – Visit 0 [Day 0]

Each morning at screening sites, ~~the~~ a government-employed nurse ~~that has been~~ trained to screen for study participants will begin with a health talk and include general information about the study (importance, aims and procedures). Health talks are routinely given to ANC attendees before the clinic begins. The screening nurse will then, as she/he processes ANC attendees, screen for potential participants by noting the residency, gestation on clinical examination and Hb. Hb is measured routinely for all pregnant women presenting for their first ANC visit. She will complete a pre-screening form and then communicate to the recruitment site if there are potential participants. She will also request the potential participant to avail herself for an ultrasound scan (within two days from the initial pre-screening visit). A study vehicle will transport the pre-screened participants to the recruitment centre for further screening procedures including an ultrasound to confirm pregnancy and determine gestation age (i.e., crown-rump length for less than or equal to 16 weeks, and femur length and head and abdominala circumference for more than 16 weeks), before obtaining consent. If a vehicle is not available, transport costs will be reimbursed. The results of the ultrasound scan will be used if at all a discrepancy with the date of last menstrual cycle will arise.

13.2.Recruitment – Visit 1 [Day 0]

If all the screening criteria including giving consent are met, then the study nurse will assign the participant a Study Number. This will be from a list of consecutive numbers specific for each recruitment site (Blantyre or Zomba). Participants will be administered a questionnaire to collect demographic, medical information, cost (travel and treatment) information, ~~and~~ wellbeing and cognitive function before having a full physical examination by a clinician.

Blood sampling

A 10ml venous blood sample will be collected for Full blood count (FBC) and reticulocyte count, iron (ferritin, sTfR) phosphate and inflammatory markers (C-reactive protein and alpha-1 glycoprotein). Malaria parasitaemia will be sought by Rapid Diagnostic Test. A filter paper sample of blood will be taken and

stored for malaria PCR. For consistency with measurement of Hb (as a primary outcome) we shall require to take a finger-prick blood sample and use a Hemocue to determine the Hb.

Study arm allocation

The study nurse will then retrieve the sealed specific Study Number-marked envelope containing the study arm allocation and open it to allocate the participant study arm. The assigned study number will be recorded in the CRF (Clinical Record Form) and Pharmacy Log.

Study drug administration

For those allocated to the IV iron arm, they will be given Ferric carboxymaltose 20mg/kg up to 1000mg (women 50kg or above) in 250mL normal saline, administered intravenously over 15 minutes. The study drug will be diluted by the pharmacist following the pharmaceutical procedures which shall be additionally outlined in a specific SOP. The study drug will be provided to the clinician for administration. An intravenous cannula will be inserted following standard aseptic procedure. Skin will be cleaned with ethanol and a sterile cannula will be inserted into the forearm or hand by a skilled clinician, and the cannula will be fixed in place with a sterile tegaderm or clinical tape. The participant will be monitored over the 15 minutes of the infusion for any adverse events and if they develop these will be attended to promptly and treated according to standard hospital management guidelines. The participant will be observed for a further 45 minutes. Following completion, the cannula will be removed and placed in a biohazard container, and a band-aid applied to the arm. We will follow universal precautions whilst working with sharps. Administration of iron will be done in a room equipped with resources to manage a severe allergic reaction. Each room will have a 'crash' trolley which will contain adrenaline, hydrocortisone, intravenous fluids and antihistamines. It will also contain airway equipment (laryngoscope and endotracheal tubes) for emergencies. Oxygen will be available in the room. The contents and expiry of the crash trolley and availability of oxygen will be confirmed each day by the study staff prior to administration of the drug. A clinician assigned to the trial will be in the treatment room when infusions are given. For those allocated to the oral iron arm, they will be given oral iron supplements to be taken twice daily for 90 days (or the duration of pregnancy, whichever is shorter). Women in the oral iron arm will not be encouraged to take the tablets more than routine antenatal advice as we want this arm to reflect as current practice as possible. Women in the IV iron arm will be instructed not to take oral iron; this will also be noted in the woman's passport so as to notify any other healthcare professionals she seeks care from during her pregnancy. All pregnant women will receive an insecticide-treated net (ITN) and IPTp first dose as standard policy in Malawi for the prevention of malaria in pregnancy from the ANC. Participants will then be dropped home by one of the research assistants to enable him/her to collect a detailed residency map in order to ease tracing of defaulters. Participants will be requested to return to the research clinic (at recruitment site) at any time that they feel ill during their pregnancy, otherwise, they will be encouraged to attend all scheduled ANC visits at their local health centre. All participants will be requested to deliver at the research site health facility or notify the research staff if in labour or delivery occurs at any other health facility.

13.3. 4-WEEK FOLLOW-UP – VISIT 2 [DAY 28 ±2]

All participants will be requested to return to the research clinic after 28(±2) days. They will be examined (as per standard ANC procedures) including an ultrasound scan before collecting a venous blood sample. If

they are ill relevant laboratory tests will be performed and they will be managed as per standard hospital guidelines. The filter paper is to allow for malaria genotyping. The participant will then be given SP 3 tablets as standard IPTp policy guidelines for Malawi as a directly observed therapy. Household economic data will also be collected. Participants will then be allowed to return home and instructed to continue their monthly ANC visits at their local health centre (these will normally be the health centres where they were screened).

Blood sampling

A 10ml venous blood sample for FBC, reticulocytes count, iron indices (ferritin, sTfR), CRP, alpha-1 glycoprotein, phosphate and malaria parasitaemia (using RDT and microscopy). Some of the blood will be put on filter paper for malaria PCR and for resistance testing for positive samples. Blood will be sent for culture if bacteraemia is clinically suspected. For consistency with measurement of Hb (as primary outcome) we shall require to take a finger-prick blood sample and use a Hemocue to determine the Hb.

Reproductive Tract Infections Determination

A serum sample will be collected for Treponema Pallidum testing. A vaginal swab will be done for bacterial vaginosis (Gram stain) and T. vaginalis and an endocervical swab for *C. trachomatis* and *N. gonorrhoeae*. If any diseases are detected, women will be notified and treated according to national guidelines. Samples will also be stored for microbiome analysis.

Urinalysis

Urine sample for urinalysis will be done and will include determination of parasites (using slide microscopy), glucose and proteins (using urine dipsticks).

Stool Analysis

A sample of stool will be collected for microscopy and stored for microbiome analysis

Maternal quality of life and productivity

Maternal quality of life will be measured using the Piper Fatigue Scale (PFS). This is a self-report instrument designed to measure fatigue. Mothers will be asked about the degree to which they are able to undertake their usual activities during the previous week to assess productivity consequences, due to their condition.

Cognitive function

Maternal cognition will be assessed using a digit span forward and backward test, and a mental rotation test.

13.4. VISIT 3 [34 WEEKS GESTATION ±2 DAYS]

Participants will be visited in their home on a bi-weekly basis from 34 weeks gestational age by a research nurse who will take a finger-prick sample of blood to determine the Hb level using a Hemocue. If the participant is unwell during these visits they will be referred to visit the health facility (research clinic).

13.5. Pre-delivery follow-up – Visit 4 [36 weeks gestation ±2 days]

All participants will be requested to return to the research clinic at 36 weeks gestation. They will undergo a physical examination (as per standard ANC procedures) including ultrasound scan and a quality of life measure. Adherence to iron will also be determined in the oral iron study arm. They will further have some biological samples collected as follows:

Blood sampling

A 10ml venous blood sample will be collected for FBC, reticulocytes count, iron indices (ferritin, sTfR), CRP alpha-1 glycoprotein, phosphate, malaria parasitaemia (using RDT and microscopy). Some of the blood will be put on filter paper for malaria PCR and for resistance testing for positive samples. For consistency with measurement of Hb (as a primary outcome) we shall require to take a finger-prick blood sample and use a Hemocue to determine the Hb.

Reproductive Tract Infections Determination

A serum sample will be collected for Treponema Pallidum testing. Per vaginal swab will be done for bacterial vaginosis (Gram stain) and T. vaginalis and an endocervical swab for *C. trachomatis* and *N. gonorrhoeae*. If any diseases are detected, women will be notified and treated according to national guidelines. Samples will also be stored for microbiome analysis.

Stool Analysis

A sample of stool will be collected for microscopy and further stored for microbiome analysis.

Urinalysis

Urine sample for urinalysis will be done and will include determination of parasites (using slide microscopy), glucose and proteins (using urine dipsticks).

Maternal quality of life and productivity

Maternal quality of life will be measured using the Piper Fatigue Scale (PFS). This is a–12-item self-report instrument designed to measure fatigue. Mothers will be asked about the degree to which they are able to undertake their usual activities during the previous week to assess productivity consequences, due to their condition. Mothers will also be requested to provide any remaining oral iron tablets (if they had been randomized to this arm) for a pill count for adherence assessment.

13.6. VISIT 5 [38 – 40 WEEKS GESTATION ±2 days]

Participants will be visited in their home each week until delivery by a research nurse who will take a finger-prick sample of blood to determine the Hb level using a Hemocue. If the participant is unwell during these visits they will be referred to visit the health facility (research clinic).

13.7. Delivery – Visit 7-[±2 days]

All participants will be encouraged to return to the recruitment health centres for delivery unless the index pregnancy is defined as a high-risk pregnancy requiring delivery at the tertiary referral hospital (QECH and ZCH). Participants delivering at home or at other health facilities will be encouraged to come within 24 hours to the research site for assessment. The study will have 24-hour cover of the delivery suite at the recruitment centres so that all data on deliveries are captured. Detailed physical examination will be done on admission and detailed clinical data on the progress of the delivery. Maternal venous blood sample will

be taken (for laboratory tests as below). Delayed cord clamping procedures will be standardized. Immediately after delivery, we will record Apgar scores, and the neonate will have a full physical examination including measurement of birthweight, length, and details of any congenital malformations. We will record whether the birth was vaginal, vaginal but assisted, or caesarean, and the indications for assisted/ caesarean births; we will also record maternal adverse events, including haemorrhage, and episiotomy. Cost information (travel and treatment) will also be collected.

Blood sampling

Maternal venous blood sample (10ml) will be collected for FBC, reticulocytes count, iron indices (ferritin, sTfR), CRP, AGP and malaria RDT and microscopy and filter paper for malaria PCR and for resistance testing for positive samples. For consistency with measurement of Hb (as a primary outcome) we shall require to take a finger-prick blood sample and use a Hemocue to determine the Hb.

Placental histology

A histological full thickness placental sample about 2 cm long x 1 cm wide will be collected into 40 ml of 10% neutral buffered formalin for malaria histological staining and examination, a cord blood smear for malaria microscopy. A placental blood smear will also be collected for microscopy, and a filter paper for malaria PCR. The placenta will be weighed and results recorded in the CRF.

Cord blood

A cord blood sample will be collected for haemogobin, a smear for malaria microscopy and PCR, and iron indices.

13.8. Post-Delivery – Visit 8 [28 days Postpartum/Postnatal ±2 days]

All participants will be requested to return to the research clinic together with their infants for a detailed medical examination of both the mother and the baby.

Blood sampling

Maternal venous blood sample (10ml) will be collected for FBC, reticulocytes count, iron indices (ferritin, sTfR), CRP alpha-1 glycoprotein, and malaria RDT, microscopy and filter paper for malaria PCR for resistance testing only for positive samples. For consistency with measurement of Hb (as a primary outcome) we shall require to take a finger-prick blood sample and use a Hemocue to determine the Hb.

Similarly, a venous blood sample of 2-3ml will be collected from the infant for, malaria RDT, microscopy and filter paper for PCR, and most importantly iron indices determination.

Maternal quality of life and productivity

Maternal quality of life will be measured using the Piper Fatigue Scale (PFS). This is a 12-item self-report instrument designed to measure fatigue. Mothers will be asked about the degree to which they are able to undertake their usual activities during the previous week to assess productivity consequences due to their condition.

Cognitive function

Maternal cognition will be assessed using a digit span forward and backward test, and a mental rotation test.

Child anthropometry

Infant weight, length and head circumference will be measured using established, calibrated tools.

13.9. Unscheduled Sick Visit [Anytime during study follow-up]

Participants will be encouraged to attend the research clinic when sick. The participants will be managed according to standard ANC management guidelines. Blood sample for malaria RDT (and microscopy if RDT positive) will be taken. Some of the blood will be put on filter paper for malaria PCR and for resistance testing for positive samples. Blood will be sent for culture if bacteraemia is clinically suspected. Cost information will also be collected.

13.10. Laboratory procedures

At each of the two recruitment sites (Limbe in Blantyre and ZCH in Zomba), we shall have a small laboratory with a lab technician where initial sample processing will be done. The site labs will have small laboratory equipment for sample collection, separation and storage (at -20°C). They will have a microscope and area for staining of malaria and other slides before reading. Lab technicians will use SOPs for all their laboratory processing of samples. These SOPs will be developed by senior laboratory technologist and reviewed by principal investigators. Samples will be transferred for long-term storage to the CoM, Blantyre campus where there is -80°C freezer. This temperature of the freezer is monitored daily and there are backup systems in case it breaks down. Blood for culture will be sent to the Malawi-Liverpool Research Laboratories also at the CoM in Blantyre for processing. They have a quality assured blood culture processing system which will be used for a fee. Some samples will be exported out of the country for more advanced assays that are not currently available in Malawi.

Full Blood Count

A Coulter® HmX haematology analyser (Beckman Coulter ™ Company, Miami Florida, USA) will be used for full blood count to determine the Hb level and red cell indices. For reticulocyte count determination, whole blood will be mixed with reticulocyte stain, then left to stand for 10min before being double read under a microscope.

Malaria testing

Testing for malaria will be done using an RDT following manufactures guidelines. Whole blood will be placed on a RDT test strip and read. If positive, a thick blood smear will be prepared for parasite count and a thin blood smear for species differentiation (although we expect >97% to be P. falciparum). The smears will be stained with May-Grunwald-Giemsa using standard techniques. All smears will be double read by certified laboratory technicians (each reading blinded of the others results). Where there are discrepant results (positive/negative or a 50% difference in the parasite counts), these slides will be read by a third person.

Placental histology will be examined for malaria using standard approaches. Placental biopsies will be fixed in formalin, embedded in paraffin and 5 mm sections cut onto glass slides. After rehydration slides will be stained with Giemsa and/or haematoxylin and eosin and passed through graded alcohols and coverslipped. Examination by microscopy will note the presence of malaria-infected erythrocytes, malaria pigment in fibrin, and malaria pigment in host leukocytes in the placental blood spaces. Placentas will be classed as

uninfected; past infection (malaria pigment, no parasites); or active infections (parasites with or without malaria pigment).

Blood for culture

A venous blood sample (1 to 2 ml) will be inoculated into BACTEC Myco/F-Lytic culture vials and incubated in a BACTEC 9050 automated culture system (Becton Dickinson) for 56 days. Sub-culturing, susceptibility testing, and isolate identification will be performed by standard techniques [49].

Blood sample preparation for long-term storage for iron and inflammatory marker assays

Blood will be drawn using standard venipuncture techniques and serum separated from the blood cells as soon as possible. Samples will be allowed to clot for one hour at room temperature, centrifuged for 10 minutes at 4 °C, and serum extracted. Serum C-reactive protein and alpha-1 glycoprotein, ferritin, sTfR will be analysed on Modular P800 and Modular Analytics E170 systems (Roche). Blood will also be stored in appropriate preservatives to enable further analysis of immune molecular aetiologies related to anaemia, infection and birth weight outcomes in pregnancy, which will be carried out at the Walter and Eliza Hall Institute of Medical Research, in Melbourne, Australia.

Vaginal and gut microbiome analysis

Vaginal and faecal infections will be tested using conventional microscopy and culture. Culture-free analysis of the microbiota will also be undertaken at the Walter and Eliza Hall Institute of Medical Research in Melbourne, Australia or partners, using approaches such as 16S rRNA gene sequencing on microbial DNA.

Table 8: Overview of (laboratory) measurements

Measurements		MEASUREMENTS on the MOTHER									
			Time Point								
	Sample type	Test	Screening (visit 0)	Enrolment (visit 1)	Day 28±2 (v2)	Week 36 (v4)	Week (HV3/5/6) wk34, 38 & 40	Delivery (visit 7)	DAY 28±2 Post-partum (visit 8)	Unscheduled or sick visits	Is the test locally available?
01.	Capillary blood (finger prick)	Haemoglobin (Hb)	x				x			If needed	Yes
02.		Malaria RDTs (mRDT)	x							x	Yes
03.		Malaria PCR									No
04.		Resistance testing if Filter paper is mRDT positive								x	No
05.		rapid HIV test	x								Yes
06.		Bioline® rapid syphilis test	x								Yes
07.	Venous blood	FBC (full blood count)		x	x	x		x	x	x	Yes
08.		reticulocytes count		x	x	x		x	x		Yes
09.		Hb (by HemoCue)		x	x	x		x	x	x	Yes
		Hb (by haematology analyser)			x	x		x	x		
		Malaria RDTs (mRDT)			x	x		x	x	x	
		Malaria PCR		x	x	x		x	x	x	
		Resistance testing if Filter paper is mRDT positive		x	x	x		x	x	x	
		Bioline® rapid syphilis test				x					
10.		Ferritin (serum)		x	x	x		x	x		No
11.		sTfR (serum)		x	x	x		x	x		No
12.		Folate		x	x	x		x	x		No
13.		Cobalamin (vit B12)		x	x	x		x	x		No
14.		Phosphate (serum)		x	x	x		x	x		No
15.		Retinol/ tocopherol		x	x	x		x	x		No
16.		CRP (C-reactive protein)		x	x	x		x	x		No
17.		alpha-1 glycoprotein									No
18.		Treponema Pallidum test (serum)			x	x					No
19.		blood culture test if bacteraemia clinically suspected			x	x		x	x	x	Sourced (yes)
20.	Vaginal swab	for bacterial vaginosis (Gram stain) and T. vaginalis tests			x	x					No

			MEASUREMENTS on the MOTHER								
Measurements			Time Point								
	Sample type	Test	Screening (visit 0)	Enrolment (visit 1)	Day 28±2 (v2)	Week 36 (v4)	Week (HV3/5/6) wk34, 38 & 40	Delivery (visit 7)	DAY 28±2 Post-partum (visit 8)	Unscheduled or sick visits	Is the test locally available?
21.	Endocervical swab	for *C. trachomatis* and *N. gonorrhoeae*			x	x					No
22.	Urine sample	for microscopic parasites analysis			x	x					Yes
23.		glucose and protein analysis using dipstick			x	x					Yes
24.	Stool sample	for microbiome analysis using a microscope			x	x					No
25.	Placental biopsy	for malaria histological staining and examination						x			No
26.	Placental blood smear	for microscopic malaria test						x			Yes
27.		for malaria PCR						x			No
28.	Cord blood	malaria microscopy						x			Yes
29.		Hb						x			Yes
30.		malaria PCR						x			No
31.		Ferritin						x			No
32.		sTfR									
		MEASUREMENTS on the INFANT									
33.	Venous blood	malaria RDT							x	x	Yes
34.		Malaria microscopy							x		
35.		Filter paper for malaria PCR							x	x	No
		Hb							x		
36.		Ferritin (serum)							x		No
37.		sTfR (serum)							x		

FBC, full blood count; HV, home visit; Hb, haemoglobin; mRDTs, malaria rapid diagnostic tests; sTfR, serum transferrin receptor; CRP, C-reactive protein; AGP, alpha 1-acid glycoprotein

13.11. ARRANGEMENTS FOR ALLOCATING PARTICIPANTS TO TRIAL GROUPS

Screening will occur at one of the centres, and participants will then be requested to attend one of the two research centres (Limbe Health Centre or ZCH) for consent, randomisation and initial treatment with IV iron or administration of oral iron. A randomization code for treatment arms will be generated by the Trial Statistician in Melbourne and individual participant codes will be pre-packed (independently) in envelopes and sealed and held securely at research sites. The randomization code will be stratified according to the research site and gravidae. The eligible participants who have met all inclusion/exclusion criteria will receive study medication assigned to the next available randomization number at the Day 0 during the first study visit. The study medication shall be determined after the research staff open the specific participant envelope which will prescribe the participant's group allocation.

14. ASSESSMENT OF SAFETY

14.1. PRECAUTIONS IN DELIVERING FCM

As discussed above, there remains a risk of hypersensitivity reactions associated with infusion of FCM. For this reason, FCM continues to only be available for infusion in health care centres, where appropriate facilities are available for treatment of any severe reactions, even though these are rare.

Standard warning text is required by the FDA to be included in all the prescribing information of all IV irons (i.e. not just FCM, but all formulations, irrespective of the specific risk associated with that particular formulation) marketed in the USA, advising that patients may present with shock, clinically significant hypotension, loss of consciousness, or collapse. The text states that it is necessary to monitor patients for signs and symptoms of hypersensitivity during and after IV iron administration for at least 30 min and until clinically stable following completion of the infusion. In Australia, it is still routine to administer FCM only in contexts where equipment for management of hypersensitivity reactions are in place: i.e. with staff trained in the management of these reactions, and drugs (steroids such as hydrocortisone, antihistamines such as chlorpheniramine, and adrenaline). It is likewise routine to ask patients to remain in the treatment centre for 30 minutes post infusion to ensure their safety. We will follow these protocols when delivering FCM to women in our trial. Both trial sites are attached to large hospitals which can support the trial staff and, if necessary, provide urgent medical care. FCM administration will be overseen by research specific medical doctors in Blantyre and Zomba.

Protocol for delivering FCM

- FCM will be administered as an infusion in 250mL of 0.9% sodium chloride
- Infusion rate will be 250mL over 15 minutes.
- The patency of the IV cannula must be confirmed before commencing any infusion to ensure there is no extravasation of iron, which can cause a permanent stain. Connect a 50mL 0.9% sodium chloride flush and allow this to infuse by gravity. If the saline does not infuse freely, there is swelling or discomfort this cannula must not be used for the IV FC.
- All women should be observed for signs of adverse reaction which may be acute or delayed (see below).

- A medical officer will be available and able to reach the infusion room within 5 minutes.
- Common adverse reactions to FCM include:
    - Headache, dizziness
    - Injection site reactions
    - Hypertension
    - Elevated liver enzymes
    - Hypophosphataemia
- Less common reactions include:
    - Nausea, abdominal pain, constipation, diarrhoea
    - Hypersensitivity including anaphylaxis
    - Paraesthesia and dysguesia
    - Hypotension
    - Tachycardia
    - Flushing
    - Back, joint or muscle pain
    - Pruritis and urticaria
    - Pyrexia, fatigue and malaise
    - Dyspnoea and bronchospasm
    - Syncope
- A member of the study nursing staff will remain in the same room as the patient during the infusion and for the 30 minutes following patient, to ensure any adverse serious reaction is rapidly detected and that the infusion can either be stopped or other appropriate treatments given.
- Vital signs to be monitored prior to infusion, 5 minutes after the start of infusion, at end of infusion, every prior to discharge, and if the participants feel unwell:
    - Heart Rate
    - Blood Pressure
    - Oxygen Saturations
    - Respiratory Rate

- Conscious State
- Temperature

Fetal Heart Rate will be monitored prior to infusion, at end of infusion and prior to discharge.

14.2. Definitions of Adverse events (AE)

Adverse Events (AE), adverse reactions (AR), unexpected adverse reactions, severity and serious adverse events/reactions will be defined in accordance with the EU guideline titles: 'Detailed guidance on the collection, verification and presentation of adverse reaction reports arising from clinical trials on medicinal products for human use, April 2004.' [50, 51] SUSARS are defined as all suspected adverse reactions related to the investigational product.

14.3. Recording of adverse events

The time period for collecting AEs

Non-serious adverse events and serious adverse events (SAE) will be collected from the time consent is given until the participant completes the study (the final visit or withdrawal).

Method of detecting AEs

At scheduled clinic visits, methods of detecting AEs will include AEs spontaneously reported by the subject and/or observed by the study staff, as well as AEs identified by means of a standard question to the subject such as "have you experienced any health problems since the last visit/ the last questioning?".

Collection of AE data

AEs will be recorded in the adverse event section of the CRF. For each AE, a description of the event, date of onset and resolution (and if applicable time), its severity, whether it constitutes a SAE or not, any action taken (e.g. other treatment given, change to study treatment, follow-up laboratory tests) and the outcome (continuing or resolved), will be given along with the investigator's assessment of relationship to study drug. Details of changes to the dosage schedule, any other treatment given or follow-up laboratory tests should be recorded on the appropriate pages of the CRF

All SAEs must be reported according to procedures described in the section 'reporting of serious adverse events'.

If a diagnosis of the subject's condition has been made then the diagnosis should be recorded as the adverse event. However if a diagnosis of the subject's condition has not been made then the individual signs or symptoms should be recorded separately.

If an AE changes in frequency or severity during the study period, a new record of the AE should be recorded in the CRF.

Assessment of severity

The investigator will use clinical judgement to make an assessment of the severity of each AE.

Assessment of causality

The investigator will use clinical judgement to assess the relationship between investigational product and the occurrence of each adverse event. Alternative causes such as natural history of the underlying diseases, concomitant therapy, other risk factors and the temporal relationship of the event to the investigational product will be considered and investigated.

The investigator will make an assessment of the causality of the AE by answering "Yes" or "No" to the question "Is there a reasonable possibility of a causal relationship between the study drug and the AE?" in the CRF.

Study Endpoints and symptoms anaemia

Adverse events judged to be symptoms of anaemia or meeting the definition of any of the study outcome (severe anaemia) will not be recorded as adverse events unless they fulfil the criteria for an SAE or result in discontinuation of study treatment.

HIV related disease

In HIV infected subjects, adverse events judged to be expected because of HIV-related disease will not be recorded as adverse events unless they fulfil the criteria for a SAE or result in discontinuation of study treatment.

Lack of efficacy and Disease Progression

Any deterioration in the subject's condition after the subject has been enrolled in the study will be discussed with / assessed by the principal investigator. Where the deterioration is considered by the principal investigator to constitute a progression of anaemia or lack of effectiveness this will not be considered an AE unless it fulfils the criteria for an SAE or results in discontinuation of study treatment. Symptoms of unexpected disease progression (as assessed by the investigator) should be recorded as AEs.

Abnormal laboratory values

The duplicate reporting of laboratory tests as both laboratory findings and adverse events in the CRF should be avoided. Abnormal laboratory tests results will not be reported as AEs in the CRF unless they fulfil the criteria for a SAE or result in discontinuation of study treatment. They will be evaluated in the overall safety analysis**.** If an abnormal laboratory value is associated with clinical signs and symptoms, the sign/symptom should be reported as an AE while the associated test result is recorded in the appropriate CRF section**.**

Overdose

Use of study medication in doses in excess of that specified under the section on Trial Interventions should be recorded as an AE. An overdose without associated symptoms should be recorded as an AE of "Overdose".

14.4. Reporting of SAEs

Reporting by the Investigator to the Study Safety Monitor and Sponsor

The investigators must report all SAEs that occur during the course of the study to the Sponsor within 24 hours of the investigational site becoming aware of it. All SAEs have to be reported, whether or not considered causally related to the investigational product or study procedure(s). All SAEs will be reported

on the SAE form provided. The reporting will be made by research medical doctors (Dr Chikondi Chapuma for in Blantyre site, Dr Glory Mzembe for and Zomba sites.

Follow-up information on SAEs must also be reported by the investigator within the same timeframe. In case of doubt about whether an event fulfils serious criteria, the case should be reported to the safety monitor who will assess whether the event should be reported as an SAE.

At both study sites, Blantyre and Zomba, there will be a Co-investigator will be assigned to be the Safety Monitor. In Zomba, it will be Dr Kabeya Biselele, a consultant Obstetrician with years of experiencing working in Malawi and other African countries managing pregnant women. He has also been a co-investigator and study physician on a large Pfizer-funded trial investigating the use of azithromycin-chloroquine in the prevention of malaria in pregnancy. In Blantyre, the Safety Monitor will be Prof William Stones, a practising consultant obstetrician with experience working in resource-poor settings and also administering FCM in a Kenyan population. The safety monitors shall be available for consultation in an emergency but shall also oversee and advise on the clinical care SOPs and guidelines within the trial. They will also be part of the Trial Steering Committee and hence provide any clinic care-related advice to the trial executive decision-making body.

Reporting by the sponsor

The sponsor is responsible for reporting SAEs and other safety issues to the Data Monitoring Committee (DMC) and the Ethics committees in an expedited manner in accordance with the EU guidelines. The sponsor will also inform all other investigators concerned of relevant information about SAEs that could adversely affect the safety of subjects.

14.5. Data Monitoring Committee

An independent Data Monitoring Committee (DMC) has been set up to review on a regular basis, safety and efficacy data of the ongoing trial. The sponsor will report all SAEs and other relevant safety information to the DMC on an expedited basis. The DMC will review tables of cumulative SAEs, primary and secondary endpoints in a blinded manner at regular intervals during the first six months of the trial and six monthly thereafter. The DMC will remain blinded when presented with any interim analysis results unless the DMC judges that for safety reason the study blind should be broken. In the advent of clear evidence of effectiveness or harm, the DMC will recommend to the sponsor and investigators whether to continue, modify, or terminate the trial on ethical grounds.

The DMC will comprise of international experts in clinical trials, obstetrics, epidemiology and statistics.

DATA HANDLING AND RECORD KEEPING

14.6. Case report Forms (CRFs)

Data collected from the subjects will be recorded in digital - Open Data Kit (ODK) Case Report Forms (CRFs) using tablets An Instruction manual on the completion of the CRF will be provided to investigators and relevant study site personnel. It will be the responsibility of the investigator to ensure that the data in the CRFs are accurate and complete. If any data are not available, omissions will be indicated on the CRFs. The CRF will automatically sign off the user and lock upon completion. Relevant hard copy patient hospital files will be scanned for reference and stored digitally, securely. All CRFs will be checked by the investigator or

an authorised personnel for accuracy and completeness before uploading to the server. All corrections will be done by the investigator or authorised personnel; corrections will be automatically saved with tracked changes. The data will then be uploaded on the server.

Data entry and validation

Data will be entered as they become available into the MS access database using double data entry. Data verification and validation will be performed until the database corresponds with the data collected in the CRFs. Data query sheets will be raised and distributed by the data manager to the study team for resolution in a timely manner. Query resolutions will be stored together with the original CRFs.

14.7. Database Lock

When the validation process is concluded the data will be declared and documented as clean. Once all decisions on the evaluability of the data from each individual subject have been made and documented, the treatment code will be broken and included in the database and the database will be locked.

15. STATISTICAL CONSIDERATIONS

15.1. Sample size

Sample size is calculated based on the maternal primary outcome of anaemia at delivery. Haider *et al* identified a 50% reduction in anaemia prevalence in women receiving oral iron. In the proposed study, all women in the trial will have moderate to severe anaemia (Hb<10g/dL) at baseline. In a cohort of pregnant women in the Gambia, we observed that 60% of pregnant women with Hb<10g/dL at 20 weeks' gestation who then receive iron interventions continue to have Hb<10g/dL by 30 weeks. The Fer-ASAP trial demonstrated a 14% reduction in absolute anaemia prevalence compared with oral iron. We hypothesise that routine iron supplementation will reduce anaemia prevalence from 100% to 60% (as seen in the Gambia, and similar to data from Haider *et al*). We hypothesize that ferric carboxymaltose will result in a 10% absolute improvement in anaemia cure (to a prevalence of 50%), as discussed above. To show this with 80% power at a two-sided alpha level of 5%, and incorporating a 10% loss to follow-up, we expect to require a total sample size of 862 pregnant women or 431 per arm is needed (using Pearson's chi-square test). This sample size allows 86.5% power to detect the clinically meaningful effect size (100g) for birth weight. Finally, 80% power for anaemia and 86.5% power for birthweight means our trial will have 69% power to demonstrate both outcomes (if they are independent).

Participants who discontinue the study will not be replaced. Recruitment will be competitive between the sites.

15.2. Data analysis

A detailed study statistical analysis plan for the final analysis will be drawn up during the course of the study before the unblinding of data at database lock.

Assessment of effectiveness

A detailed statistical analysis plan describing the proposed analyses will be finalised before the trial database is locked for final analysis. Analyses will be undertaken on an intention-to-treat basis (maternal outcomes: all women randomized; neonatal outcomes: all live-borns with the exception of the stillbirth outcome). Descriptive statistics will be presented for all outcomes, by treatment groups across the follow-

up time points. The primary maternal outcome anaemia at pre-delivery (based on the capillary Hb closest prior to delivery) will be analysed using a binomial log-linear regression, with the oral iron group as the reference, and incorporating clinic and treatment in the model. The primary maternal hypothesis will be evaluated by obtaining the estimate of the risk ratio of IV iron versus oral iron and a 95% confidence interval. The secondary neonatal outcome birthweight will be analysed by fitting a linear regression model with a model specification similar to that of the primary maternal outcome. This hypothesis will be evaluated by obtaining the estimate of the absolute difference in birth weight between IV iron and oral iron and a 95% confidence interval. Other secondary binary outcomes will be analysed similarly as the primary maternal outcome and other secondary continuous outcomes as the birthweight outcome, where applicable also taking into account in the model the repeated data collection of the outcome. Missing values in all outcomes will be reported across treatment groups and follow-up time points. Multiple imputations under the missing at random assumption will be performed as a secondary analysis for handling missing data in the primary maternal and neonate outcome; results will be compared with the primary complete case analysis to investigate the robustness of the findings to missing data.

Analysis of adverse events

Adverse reactions will be reported and tabulated for each treatment arm, overall and per body system and adverse event, on an intention to treat basis. Treatment-emergent adverse events are defined as adverse events that had an onset day on or after the day of the first dose of study medication. Adverse events that have missing onset dates will be considered to be treatment emergent. No formal statistical testing will be undertaken. All laboratory data will be listed.

Health economics analyses

An economic evaluation will be done to consider the cost-effectiveness of IV iron over the standard of care. The economic evaluation will estimate absolute and incremental health provider and patient costs as well as health benefits of the two study arms. The provider costs will include costs related to delivering the interventions and will be collected prospectively at the facilities included in the study. Patient cost data including indirect disease costs (productivity), will be collected as part of the routine trial CRFs. Effectiveness, compliance and health-related quality of life associated with intravenous iron versus standard of care will be based on primary trial data. A novel decision model will be developed to combine these data, supplemented by best available secondary data when required. This Markov model will take into account outcomes related to both the mothers and the children. Uncertainties related to single parameters will be captured using one-way sensitivity analyses, while overall uncertainty related to its predictions will be analysed using probabilistic sensitivity analyses. Finally, the model will be used to estimate in a threshold analysis a cost for the drug at which use of IV ferric carboxymaltose could be feasibly implemented in practice; this estimate will form the basis for future discussions with manufacturers and donors.

15.3. Analysis Populations

Patients will be considered as ‘completed the study’ if they satisfied all study entry criteria, have received the treatment and have attended all visits till delivery as prescribed by the protocol and provided relevant biological samples as defined for these visits. A discontinuation is any subject who enters the study (i.e., gives informed consent), but does not complete the study and who took at least one dose of study

medication at recruitment. If a subject gives informed consent and then does not enter the study (i.e. is not allocated a Study Number) they will be classified as a screen failure.

The Intention-to-treat population is defined as all patients who have received at least one dose of study medication (at recruitment) and will be included in the intention-to-treat analysis regardless of whether they completed the study. The per-protocol population is defined as all patients in the intention-to-treat population who have completed the study (i.e. attended all scheduled study visits).

15.4. Missing Data

Every effort will be made to minimise the amount of missing data in the trial. Whenever possible, information on the reason for missing data will be obtained. No adjustments will be made for missing data. Missing values in all outcomes will be reported across treatment groups and follow-up time points. Multiple imputations under the missing at random assumption will be performed as a secondary analysis for handling missing data in the primary maternal and neonate outcome; results will be compared with the primary complete case analysis to investigate the robustness of the findings to missing data.

15.5. Interim analyses and criteria for termination of the trial

Due to the short recruitment period of the trial, an interim analysis has not been planned. However, the sponsor reserves the right to temporarily suspend or prematurely discontinue this study at any time for reasons including, but are not limited to, safety or ethical issues or severe non-compliance. If the sponsor determines such action is needed, it will discuss this with the investigator. When feasible, the sponsor will provide advance notification to the investigator of the impending action prior to it taking effect. The sponsor will promptly inform all the Ethics committee and regulatory bodies and provide the reason for the suspension or termination.

16. STUDY MANAGEMENT

16.1. Study Monitoring

The Research Support Centre at the College of Medicine will perform independent monitoring of the study on behalf of the sponsor. Prior to subject enrolment, the monitor will visit the study site to determine the adequacy of facilities, review the protocol and data collection procedures and discuss the responsibilities of the investigator and other study site personnel.

During the study, the monitor will have regular site contacts, including conducting on-site visits to:

1. Confirm that the study is being performed according to the protocol, ICH GCP and applicable regulations, data are being accurately recorded in the CRFs, samples are being appropriately collected and stored, and that investigational product accountability is being performed.
2. Conduct source data verification
3. Confirm facilities remain acceptable
4. Provide information and support to the investigators
5. Evaluate study progress

Upon completion of the study the monitor will visit the study site to verify that all CRFs are completed and collected, all data queries have been resolved and filed, conduct final accountability, reconciliation and arrangements for investigational product and verify all study site records are complete. The PI and relevant staff will be available at monitoring visits and agree to allocate sufficient time to the monitor to discuss any issues and address their resolution.

16.2. Direct Access to Source Data/Documents

The investigator agrees to allow the sponsor and/or its representatives, including the monitor, the DMC, the IRB/IEC, the regulatory body direct access to source data and other relevant documents.

16.3. Quality Assurance

Authorised representatives of the sponsor, an IEC/IRB or regulatory authority may visit the study site to perform audits or inspections, including source data verification.

16.4. Training of Staff

The PI is responsible for the conduct of the study at this Study Site, including delegation of specified study responsibilities, and training of study staff. The PI will maintain a record of all individuals involved in the study (medical, nursing and other staff). The PI will ensure that all persons assisting with the trial receive the appropriate training about the protocol, the investigational product(s) and their trial-related duties and functions. During the study the regular spot checks will be conducted to assess the performance of study site staff members and re-training provided where necessary.

16.5. Changes to the Protocol

No change will be made to the approved protocol without the agreement of the sponsor. If it is necessary for the protocol to be amended, the protocol amendment will be submitted to the IRB/IEC for approval before implementation. Any change to the informed consent form must also be approved by the sponsor and IRB/IEC, before the revised form is used. The sponsor will distribute amendments to the PI, who in turn is responsible for the distribution of these documents to the staff at his/her study site.

16.6. Financing and Insurance

Funding for this study will be provided by Bill & Melinda Gates Foundation through a grant to the Walter and Eliza Hall Institute of Medical Research, Melbourne, Australia. The College of Medicine will act as sponsor and the PI will purchase a liability insurance policy that covers this study.

16.7. Study DURATION

The study will start as soon as all pre-study activities and documentation are completed and the IRB/IEC and Regulatory Authority have approved the protocol. The total duration of the trial is 18 months, which includes including 12 months of recruitment, 6 month of additional follow-up phase as per Gantt chart below:

Table 9: Study time plan

	2018											2019												2020			
**ACTIVITY**	F	M	A	M	J	J	A	S	O	N	D	J	F	M	A	M	J	J	A	S	O	N	D	J	F	M	A
Site preparation	■	■	■																								
Participant recruitment				■	■	■	■	■	■	■	■	■	■	■	■												
Participant follow-up																■	■	■	■	■	■						
Data analysis																						■	■	■			
Report writing																									■	■	■

16.8. Record-keeping and Archiving

During the study, an Investigator Site File will be used to store documentation pertaining to the study and it will be kept in a secure location with access only to authorised individuals. It is the PI's responsibility to continuously update the Investigator file. It must be available to the Monitor during monitoring visits.

Following study closure, the investigator study file, CRFs, medical records and other source documents must be retained at the study site per regulatory obligations (two years after the last marketing application of the study drug in the European Union) and thereafter destroyed only after agreement with the sponsor. Documents with a limited shelf life (e.g. printouts on light/heat sensitive paper) will be copied and verified by signing and dating.

16.9. Reporting and publication of data

The results of the study will be submitted and discussed with the local and national medical authorities. They will then be presented at national and international conferences and submitted for publication in peer-reviewed journals, in accordance with the sponsor' publication policy.

17. ETHICAL CONSIDERATIONS

17.1. Ethical review

This study will be submitted to the following institutional review boards and independent ethics committees (IRB/IEC).

1. College of Medicine, Blantyre, Malawi

2. Walter and Eliza Hall Institute of Medical Research, Melbourne, Australia

Before initiating the trial, written and dated approval/favourable opinion must be obtained from the IRB/IECs. Protocol amendments will be submitted to the IRB/IEC for approval before implementation. Progress reports, SUSAR reports and safety reports will be submitted to the IRB/IEC in accordance with local requirements.

17.2. Ethical conduct of the study

This study will be conducted in accordance with The International Conference on Harmonisation of Technical Requirements for Registration of Pharmaceuticals for Human Use (ICH) (ICH) guidelines for "good clinical practice" (GCP) and all applicable regulatory requirements, including, where applicable, the 1996 version of the Declaration of Helsinki.

17.3. INFORMED CONSENT

Informed consent will be obtained from each participant before conducting any study related procedure. The PI will ensure that that the participant (and the subject's parent/guardian in case of assent) is given full and adequate oral and written information about the study. Information will be provided in the local language of the participant. The participant should be given the opportunity to ask questions and allowed time to consider the information provided.

Informed consent will be documented by the use of a written consent form signed by the participant and the person who conducted the informed consent discussion. If the participant is unable to write their signature, then a thumbprint may be used. If the participant is unable to read the information her/himself, full and comprehensive information will be communicated to the participant in the presence of a witness. The witness will be an independent third party i.e. a person not connected with the conduct of the trial. The witness will sign the informed consent form to attest that the information in the consent form was accurately explained to and apparently understood by the participant and that informed consent was freely given. Each original signed informed consent will be kept on file by the investigator. A copy of the informed consent form will be provided to the participant.

17.4. RISKS TO STUDY PARTICIPANTS

All efforts will be made to minimise pain, discomfort, and fear for the children.

Blood sampling

The amount of blood taken for the study is minimal, totalling approximately up to 10 ml per visit over the study period (an additional 5 ml may be taken during sick visits for clinically-relevant laboratory analyses). Blood will be collected in most cases through a venepuncture and where indicated a finger prick. A small bruise or mild pain on the finger or the venepuncture site from where the blood is taken may develop. Only well trained and fully qualified laboratory staff will be hired for this project. Only new disposable needles and lancets are used for the blood taking procedures, and these will be discarded immediately after their use.

IV infusion

Participants allocated to the intervention arm will receive an intravenous infusion. In this case, there will similarly undergo a venepuncture where a cannula will be inserted to allow the infusion of the iron over a period of 15 minutes. A small bruise or mild pain on the venepuncture site may develop. As discussed above, anaphylaxis is not a major risk of ferric carboxymaltose there will always be an emergency resuscitation tray during this procedure. A fully qualified clinician will be available during the entire infusion process (and for a further 45 min after) to monitor the condition of the participant. The cannula will not be removed until the trial participant is certified as well with normal blood pressure and heart rate prior to discharge home.

17.5. BENEFITS FROM PARTICIPATION

Participants enrolled in the study will receive close monitoring of their condition for the study period. A dedicated clinician will see subjects at recruitment and scheduled follow-up visits, for full medical assessments. If a subject has an illness in between scheduled visits, the subject will be seen by the study

clinician and treated at the study clinic if the illness is deemed to be study related or referred to other clinics if other care is required.

17.6. Subject Data Protection

Study data will be stored in computer database maintaining confidentiality in accordance with data legislation. The subject's names will not be recorded in the database but will be identified by subject number. Authorised members of the sponsor, clinical research organisation, DMC, IEC/IRB and regulatory authority may review the subjects' medical records. This is explained in the informed consent form

17.7. Other ethical considerations

Reimbursement of costs

The study will provide payment for all study drugs, study procedures, study related visits and reasonable medical expenses that may be incurred as a direct result of the study. This includes compensation for transport for each study visit. Where applicable the participant will be provided with or reimbursed for, refreshments when a scheduled appointment requires them to be out of their home at meal times. In Malawi, in-patient hospital care is free of charge.

18. DISSEMINATION OF RESULTS

At the end of the study, the results will first be disseminated to national policymakers, government departments, academics from local research institutions and universities, COMREC and professional bodies in Malawi at the national stakeholders' meeting or research dissemination conferences to be held in the country. Subject to the findings of the study and based on consensus emerging at these meetings, we will support national policymakers to develop the necessary tools and guidelines to guide national and district level health providers to implement the strategy within hospital services and the health system more broadly.

Research results will also be disseminated to the global research community, technical agencies, and international government bodies via peer-reviewed journals and at international scientific fora. We will also inform other international organisations and funders of large-scale anaemia control initiatives which aim to improve anaemia at regional and local levels and are instrumental in supporting countries to implement anaemia control policies in low and middle-income countries.

19. CAPACITY BUILDING

Research capacity in research in Malawi will be enhanced by the provision of training and mentorship for research staff. By running this study, capacity in trial management will be enhanced. The research study will strengthen the clinical skills of health workers in managing pregnant women with anaemia. There will be 1-2 PhD candidates who will conduct their research as part of this project. Partners from the different institutions forming this research network will jointly supervise them.

In addition, there will be a post-doctoral research scientist who will work closely with the study PIs in the conduct of the study. In the process, the post-doctoral research scientist will be able to carry out various research projects from this study that will be published in peer-reviewed journals. Lastly, all study staff will undertake Good Clinical Practice (GCP) training before starting to work on the research project.

20.

21. BUDGET

PERSONNEL	Year 1	Year 2	Year 3	Total
**Scientific Team**				
Project Administration	122,796	126,480	87,633	336,909
Data Management	22,476	28,317	10,464	61,257
Clinical Personnel	143,154	174,220	10,357	327,731
Laboratory Personnel	28,275	38,415	9,817	76,507
**Total - Personnel**	**316,701**	**367,431**	**118,270**	**802,403**
**EQUIPMENT**				
Vehicle - 4x4	108,000			**108,000**
Motor-bike	11,000			**11,000**
Desk-top computer	7,200			**7,200**
Laptops	10,400			**10,400**
Printer & accessories	2,200			**2,200**
Hemocue machine	3,300			**3,300**
Ultrasound machine	50,000			**50,000**
Small clinical equipment	9,000		9,000	**18,000**
Small laboratory equipment	10,000		10,000	**20,000**
**Total - Equipment**	**211,100**	**-**	**19,000**	**230,100**
**CONSUMABLES**				
Lab assays	6,720	20,160	40,320	**67,200**
Lab consumables	2,040	6,800	4,760	**13,600**
RDT cuvettes	2,000	6,000	4,000	**12,000**
FBC reagents	5,520	5,520	5,520	**16,560**
FBC machine maintenance	4,000	4,000	4,000	**12,000**
Fuel - bikes & Cars	8,280	11,040	5,520	**24,840**
Maintenance & insurance	10,400	10,400	10,400	**31,200**
Participant costs	5,120	6,400	1,280	**12,800**
Communications & internet	6,456	6,456	6,456	**19,368**
Stationary	6,720	6,720	3,360	**16,800**
Office refurbishments/rental	8,000	1,000	1,000	**10,000**
Study Monitoring	4,000	10,000	6,000	**20,000**
Trial Insurance	50,000	-	-	**50,000**
Ethics submission fee - Malawi	150	-	-	**150**
Regulatory submission fee - Malawi	6,000	-	-	**6,000**
Regulatory annual submission fee	-	1,000	1,000	**2,000**
Short Courses e.g. GCP	5,400	1,350	1,350	**8,100**
Annual team meetings	1,300	1,300	1,300	**3,900**
**TOTAL - Consumables**	**132,106**	**98,146**	**96,266**	**326,518**
**TOTAL - Overheads (10%)**	**65,991**	**46,558**	**23,354**	**135,902**
**GRAND TOTAL ($)**	**725,898**	**512,135**	**256,890**	**1,494,923**

22. REFERENCES

1. World Health Organization, *The global prevalence of anaemia in 2011*. 2015, Geneva: World Health Organization.
2. Haider, B.A., et al., *Anaemia, prenatal iron use, and risk of adverse pregnancy outcomes: systematic review and meta-analysis.* BMJ, 2013. **346**: p. f3443.
3. WHO/UNICEF/UNU, *Iron Deficiency Anaemia: Assessment, Prevention, and Control. A guide for programme managers*. 2001, Geneva: World Health Organization.
4. Nair, M., M. Knight, and J.J. Kurinczuk, *Risk factors and newborn outcomes associated with maternal deaths in the UK from 2009 to 2013: a national case-control study.* BJOG, 2016. **123**(10): p. 1654-62.
5. Drassinower, D., et al., *The effect of maternal haematocrit on offspring IQ at 4 and 7 years of age: a secondary analysis.* BJOG, 2016.
6. World Health Organization, *Global Nutrition Targets 2025: Anaemia Policy Brief*. 2014, World Health Organization: Geneva.
7. Mwangi, M.N., et al., *Effect of Daily Antenatal Iron Supplementation on Plasmodium Infection in Kenyan Women: A Randomized Clinical Trial.* JAMA, 2015. **314**(10): p. 1009-20.
8. Pena-Rosas, J.P., et al., *Daily oral iron supplementation during pregnancy.* Cochrane Database Syst Rev, 2015(7): p. CD004736.
9. WHO, *Essential Nutrition Actions: Improving maternal, newborn, infant and young child health and nutrition*. 2013, Geneva: World Health Organization.
10. World Health Organization, *Iron and folate supplementation*. Integrated management of pregnancy and childbirth (IMPAC). 2006, Geneva: World Health Organization.
11. Low, M.S., et al., *Daily iron supplementation for improving anaemia, iron status and health in menstruating women.* Cochrane Database Syst Rev, 2016. **4**: p. CD009747.
12. Bah, A., et al., *Serum Hepcidin Concentrations Decline during Pregnancy and May Identify Iron Deficiency: Analysis of a Longitudinal Pregnancy Cohort in The Gambia.* J Nutr, 2017. **147**(6): p. 1131-1137.
13. Lyseng-Williamson, K.A. and G.M. Keating, *Ferric carboxymaltose: a review of its use in iron-deficiency anaemia.* Drugs, 2009. **69**(6): p. 739-56.
14. Friedrisch, J.R. and R.D. Cancado, *Intravenous ferric carboxymaltose for the treatment of iron deficiency anemia.* Rev Bras Hematol Hemoter, 2015. **37**(6): p. 400-5.
15. Rognoni, C., et al., *Efficacy and Safety of Ferric Carboxymaltose and Other Formulations in Iron-Deficient Patients: A Systematic Review and Network Meta-analysis of Randomised Controlled Trials.* Clin Drug Investig, 2016. **36**(3): p. 177-94.
16. Moore, R.A., et al., *Meta-analysis of efficacy and safety of intravenous ferric carboxymaltose (Ferinject) from clinical trial reports and published trial data.* BMC blood disorders, 2011. **11**(1): p. 4.
17. Breymann, C., et al., *Ferric carboxymaltose vs. oral iron in the treatment of pregnant women with iron deficiency anemia: an international, open-label, randomized controlled trial (FER-ASAP).* J Perinat Med, 2017. **45**(4): p. 443-453.
18. Van Wyck, D.B., et al., *Intravenous ferric carboxymaltose compared with oral iron in the treatment of postpartum anemia: a randomized controlled trial.* Obstet Gynecol, 2007. **110**(2 Pt 1): p. 267-78.
19. Cortes, X., et al., *Safety of ferric carboxymaltose immediately after infliximab administration, in a single session, in inflammatory bowel disease patients with iron deficiency: a pilot study.* PLoS One, 2015. **10**(5): p. e0128156.
20. Laass, M.W., et al., *Effectiveness and safety of ferric carboxymaltose treatment in children and adolescents with inflammatory bowel disease and other gastrointestinal diseases.* BMC Gastroenterol, 2014. **14**: p. 184.
21. Evstatiev, R., et al., *Ferric carboxymaltose prevents recurrence of anemia in patients with inflammatory bowel disease.* Clin Gastroenterol Hepatol, 2013. **11**(3): p. 269-77.
22. Befrits, R., et al., *Anemia and iron deficiency in inflammatory bowel disease: an open, prospective, observational study on diagnosis, treatment with ferric carboxymaltose and quality of life.* Scand J Gastroenterol, 2013. **48**(9): p. 1027-32.

23. Beigel, F., et al., *Iron status and analysis of efficacy and safety of ferric carboxymaltose treatment in patients with inflammatory bowel disease.* Digestion, 2012. **85**(1): p. 47-54.
24. Evstatiev, R., et al., *FERGIcor, a randomized controlled trial on ferric carboxymaltose for iron deficiency anemia in inflammatory bowel disease.* Gastroenterology, 2011. **141**(3): p. 846-853 e1-2.
25. Kulnigg, S., et al., *A novel intravenous iron formulation for treatment of anemia in inflammatory bowel disease: the ferric carboxymaltose (FERINJECT) randomized controlled trial.* Am J Gastroenterol, 2008. **103**(5): p. 1182-92.
26. Ponikowski, P., et al., *The impact of intravenous ferric carboxymaltose on renal function: an analysis of the FAIR-HF study.* Eur J Heart Fail, 2015. **17**(3): p. 329-39.
27. Szczech, L.A., et al., *Randomized evaluation of efficacy and safety of ferric carboxymaltose in patients with iron deficiency anaemia and impaired renal function (REPAIR-IDA): rationale and study design.* Nephrol Dial Transplant, 2010. **25**(7): p. 2368-75.
28. Anker, S.D., et al., *Ferric carboxymaltose in patients with heart failure and iron deficiency.* N Engl J Med, 2009. **361**(25): p. 2436-48.
29. Khalafallah, A.A., et al., *Intravenous ferric carboxymaltose versus standard care in the management of postoperative anaemia: a prospective, open-label, randomised controlled trial.* Lancet Haematol, 2016. **3**(9): p. e415-25.
30. Nores, J.A., *The Efficacy of IV Ferric Carboxymaltose in the Perioperative Management of Moderate to Severe Iron Deficiency Anemia.* J Minim Invasive Gynecol, 2015. **22**(6S): p. S211-S212.
31. Borstlap, W.A.A., et al., *Multicentre randomized controlled trial comparing ferric(III)carboxymaltose infusion with oral iron supplementation in the treatment of preoperative anaemia in colorectal cancer patients.* BMC Surg, 2015. **15**: p. 78.
32. *A multicentre comparative study on the efficacy of intravenous ferric carboxymaltose and iron sucrose for correcting preoperative anaemia in patients undergoing major elective surgery British Journal of Anaesthesia, 2015; 107(3): 477-78, DOI 10.1093/bja/aer242.* Br J Anaesth, 2015. **115**(1): p. 154.
33. Bernabeu-Wittel, M., et al., *Ferric carboxymaltose with or without erythropoietin for the prevention of red-cell transfusions in the perioperative period of osteoporotic hip fractures: a randomized contolled trial. The PAHFRAC-01 project.* BMC Musculoskelet Disord, 2012. **13**: p. 27.
34. Bisbe, E., et al., *A multicentre comparative study on the efficacy of intravenous ferric carboxymaltose and iron sucrose for correcting preoperative anaemia in patients undergoing major elective surgery.* Br J Anaesth, 2011. **107**(3): p. 477-8.
35. Froessler, B., et al., *Intravenous ferric carboxymaltose for anaemia in pregnancy.* BMC Pregnancy Childbirth, 2014. **14**: p. 115.
36. D, Z., et al., *Intravenous Iron Treatment in Pregnancy: Ferric Carboxymaltose for Correction of Iron Deficiency Anaemia.* Faridpur Med. Coll. J., 2017. **12**(2): p. 54-57.
37. Mishra, V., et al., *Role of Intravenous Ferric Carboxy-maltose in Pregnant Women with Iron Deficiency Anaemia.* J Nepal Health Res Counc, 2017. **15**(2): p. 96-99.
38. Christoph, P., et al., *Intravenous iron treatment in pregnancy: comparison of high-dose ferric carboxymaltose vs. iron sucrose.* J Perinat Med, 2012. **40**(5): p. 469-74.
39. Pavord, S., et al., *UK guidelines on the management of iron deficiency in pregnancy.* Br J Haematol, 2012. **156**(5): p. 588-600.
40. Rao, A., et al., *FERINJECT IN PREGNANCY AND THE POSTPARTUM PERIOD FOR TREATING IRON DEFICIENCY ANAEMIA*. 2015, Mid Essex Hospital Services NHS Trust.
41. Khalafallah, A.A. and A.E. Dennis, *Iron deficiency anaemia in pregnancy and postpartum: pathophysiology and effect of oral versus intravenous iron therapy.* J Pregnancy, 2012. **2012**: p. 630519.
42. Achebe, M.M. and A. Gafter-Gvili, *How I treat anemia in pregnancy: iron, cobalamin, and folate.* Blood, 2017. **129**(8): p. 940-949.
43. Daru, J., N.A. Cooper, and K.S. Khan, *Systematic review of randomized trials of the effect of iron supplementation on iron stores and oxygen carrying capacity in pregnancy.* Acta Obstet Gynecol Scand, 2016. **95**(3): p. 270-9.
44. Favrat, B., et al., *Evaluation of a single dose of ferric carboxymaltose in fatigued, iron-deficient women--PREFER a randomized, placebo-controlled study.* PLoS One, 2014. **9**(4): p. e94217.

45. Bailie, G.R., N.A. Mason, and T.G. Valaoras, *Safety and tolerability of intravenous ferric carboxymaltose in patients with iron deficiency anemia.* Hemodial Int, 2010. **14**(1): p. 47-54.
46. Breymann, C., et al., *Ferric carboxymaltose vs. oral iron in the treatment of pregnant women with iron deficiency anemia: an international, open-label, randomized controlled trial (FER-ASAP).* J Perinat Med, 2016.
47. Kimani, J., et al., *Efficacy and Safety of Azithromycin-Chloroquine versus Sulfadoxine-Pyrimethamine for Intermittent Preventive Treatment of Plasmodium falciparum Malaria Infection in Pregnant Women in Africa: An Open-Label, Randomized Trial.* PLoS One, 2016. **11**(6): p. e0157045.
48. Bothwell, T.H., *Iron requirements in pregnancy and strategies to meet them.* Am J Clin Nutr, 2000. **72**(1 Suppl): p. 257S-264S.
49. Graham, S.M., et al., *Clinical presentation of non-typhoidal Salmonella bacteraemia in Malawian children.* Trans R Soc Trop Med Hyg, 2000. **94**(3): p. 310-4.
50. European Commission. *Detailed guidance on the collection, verification and presentation of adverse reaction reports arising from clinical trials on medicinal products for human use, Revision 1, ENTR/CT 3. http://eudract.emea.eu.int/docs/Detailed%20guidance%20collection%20of%20adverse%20events.pdf*. Single market : management & legislation for consumer goods. Pharmaceuticals : regulatory framework and market authorisations 2004; Available from: http://eudract.emea.eu.int/docs/Detailed%20guidance%20collection%20of%20adverse%20events.pdf
51. European Commission. *Detailed guidance on the European database of Suspected Unexpected Serious Adverse Reactions (Eudravigilance - Clinical Trial Module), Revision 1, ENTR/CT 4. http://eudract.emea.eu.int/docs/Detailed%20guidance%20SUSAR.pdf* Single market : management & legislation for consumer goods. Pharmaceuticals : regulatory framework and market authorisations 2004; Available from: http://eudract.emea.eu.int/docs/Detailed%20guidance%20SUSAR.pdf
52. Bager, P. and J.F. Dahlerup, *Randomised clinical trial: oral vs. intravenous iron after upper gastrointestinal haemorrhage--a placebo-controlled study.* Aliment Pharmacol Ther, 2014. **39**(2): p. 176-87.
53. Breymann, C., et al., *Comparative efficacy and safety of intravenous ferric carboxymaltose in the treatment of postpartum iron deficiency anemia.* Int J Gynaecol Obstet, 2008. **101**(1): p. 67-73.
54. Onken, J.E., et al., *Ferric carboxymaltose in patients with iron-deficiency anemia and impaired renal function: the REPAIR-IDA trial.* Nephrol Dial Transplant, 2014. **29**(4): p. 833-42.
55. Onken, J.E., et al., *A multicenter, randomized, active-controlled study to investigate the efficacy and safety of intravenous ferric carboxymaltose in patients with iron deficiency anemia.* Transfusion, 2014. **54**(2): p. 306-15.
56. Qunibi, W.Y., et al., *A randomized controlled trial comparing intravenous ferric carboxymaltose with oral iron for treatment of iron deficiency anaemia of non-dialysis-dependent chronic kidney disease patients.* Nephrol Dial Transplant, 2011. **26**(5): p. 1599-607.
57. Schatz, U., et al., *Iron deficiency and its management in patients undergoing lipoprotein apheresis. Comparison of two parenteral iron formulations.* Atheroscler Suppl, 2013. **14**(1): p. 115-22.
58. Seid, M.H., et al., *Ferric carboxymaltose injection in the treatment of postpartum iron deficiency anemia: a randomized controlled clinical trial.* Am J Obstet Gynecol, 2008. **199**(4): p. 435 e1-7.
59. Van Wyck, D.B., et al., *Large-dose intravenous ferric carboxymaltose injection for iron deficiency anemia in heavy uterine bleeding: a randomized, controlled trial.* Transfusion, 2009. **49**(12): p. 2719-28.

23. APPENDIX A - Safety and Efficacy of FCM from randomised controlled trials

Safety and Efficacy of FCM from randomised controlled trials – from Rognoni et al[15]

Study (year)	Patients	Condition	Inclusion Criteria	Intervention arm	Control arm	Efficacy	Safety
Bager et al. (2014)[52]	97	Non-variceal acute upper gastrointestinal bleeding	Hb <12 g/dl for women and <13 g/dl for men	Intravenous ferric carboxymaltose (1000 mg; single infusion)	Oral ferrous sulphate (100 mg; twice daily; 3 months) or intravenous placebo (saline)	At week 13, mean Hb (g/dl) in ferric carboxymaltose group was 13.9 (13.4–14.3) vs. 13.5 (12.9–14.1) in oral iron and 11.5 (10.3–12.9) in placebo ($p < 0.01$)	Re-admission for bleeding was reported in 2.4 % of patients in both the oral and intravenous groups (p = NS). Admission due to cardiovascular symptoms was reported in 4.9, 4.8 and 7.1 % of oral, intravenous and placebo groups, respectively (p = NS)
Bailie et al. (2010)[45]	559	Iron deficiency anaemia (various conditions)	Hb <12 g/dl; TSAT ≤25 %; serum ferritin <300 ng/ml (CKD and IBD patients) or ≤100 ng/ml (other)	Intravenous ferric carboxymaltose (1000 mg; single infusion)	Intravenous placebo (normal saline, 100–250 ml)	NR	During the first 24 h of the treatment, at least one TEAE was experienced by 15.0 % of subjects after receiving ferric carboxymaltose and 11.4 % after receiving placebo ($p = 0.066$). During the 7-day treatment period, at least one TEAE was experienced by 29.3 % of the subjects after receiving ferric carboxymaltose and 19.7 % after receiving placebo ($p < 0.001$). The most common events in the ferric carboxymaltose group were headache (5.4 %), nausea (3.8 %) and dizziness (1.8

							%)
Breymann et al. (2008)[53]	349	Post-partum anaemia	Hb ≤10.5 g/dl	Intravenous ferric carboxymaltose (1000 mg; up to 3 weekly doses)	Oral ferrous sulphate (100 mg; twice per day; 12 weeks)	At week 12, Hb levels increased to a mean of 130.4 g/l in ferric carboxymaltose vs. 128.9 g /l in ferrous sulphate (*p* = NS). Mean ferritin was 161.2 µg/l vs. 43.3 µg/l (*p* < 0.0001)	Overall, AEs were experienced by 26.0 % in ferric carboxymaltose vs. 22.2 % in ferrous sulphate (*p* = 0.51). Statistically significant differences (ferric carboxymaltose vs. ferrous sulphate) were seen in gastrointestinal disorders (3.5 vs. 10.3 %; *p* = 0.015), general disorders and administration site conditions (6.2 vs.0.0 %; *p* = 0.003), and musculoskeletal and connective tissue disorders (0.0 vs. 2.6 %; *p* = 0.039). Constipation was less common in ferric carboxymaltose group (0.4 vs. 6.8 %). Two patients in ferric carboxymaltose group and none in the control group experienced severe AEs (i.e. hypersensitivity). There were no cases of anaphylactic shock/reaction
Evstatiev et al. (2013)[21]	256	Inflammatory bowel disease	Hb ≥12 g/dl in women and ≥13 g/dl in men	Intravenous ferric carboxymaltose (500 mg; single infusion)	Placebo (saline solution; 500 mg)	Anaemia recurred in 26.7 % of patients given ferric carboxymaltose vs. 39.4 % given placebo. Time to anaemia recurrence was longer in the ferric carboxymaltose group (*p* = 0.049). Ferritin and TSAT increased by 30.3 µg/l and 0.6 %, respectively, in ferric carboxymaltose group; the same parameters decreased by 36.1 µg/l and 4.0 %,	AEs were reported in 59.0 % in ferric carboxymaltose group vs. 50.5 % of placebo group, and serious AEs in 6.7 and 8.1 %, respectively. The most common AEs were ulcerative colitis-specific symptoms and nasopharyngitis (7.8 vs. 7.3 %). Gastrointestinal symptoms and flares of ulcerative colitis were somewhat less frequent with ferric carboxymaltose compared with placebo: 20.0 vs 28.3 % (*p* = 0.17) and 6.7 vs. 12.1 % (*p* = 0.18), respectively. No anaphylactic reaction

						respectively, in placebo group	was reported and no death occurred
Evstatiev et al. (2011)[24]	85	Inflammatory bowel disease	Hb = 7–12 g/dl (women) and 7–13 g/dl (men)	Intravenous ferric carboxymaltose (500 or 1000 mg; up to 3 infusions)	Intravenous iron sucrose (200 mg; up to 11 infusions, twice weekly)	More patients with ferric carboxymaltose than iron sucrose achieved Hb response (65.8 vs. 53.6 %; *p* = 0.004) or Hb normalisation (72.8 vs. 61.8 %; *p* = 0.015)	The frequency of treatment-related AEs was comparable between the two groups (*p* = 0.413). One treatment-related serious AE (i.e. pulmonary embolism) was reported in ferric carboxymaltose group. Skin and subcutaneous tissue disorders such as rash, dermatitis and pruritus were reported in 3.7 % of ferric carboxymaltose patients and 0.8 % of the iron sucrose group (*p* = 0.063). No true hypersensitivity reactions were reported
Favrat et al. (2014)[44]	94	Fatigue in premenopausal, non-anaemic women	Serum ferritin <50 µg/l and TSAT <20 %; Hb ≥115 g/l. Piper Fatigue Scale (PFS) score ≥5	Intravenous ferric carboxymaltose (1000 mg; single infusion)	Intravenous placebo (saline, 1000 mg)	Fatigue was reduced in 65.3 % (ferric carboxymaltose) and 52.7 % (placebo) of patients (OR = 1.68; *p*= 0.03). All ferric carboxymaltose -treated patients (vs. 86 % in placebo group) had haemoglobin levels ≥120 g/l	TEAEs were experienced by 57.2 % of ferric carboxymaltose and 49.0 % of placebo-treated patients (*p* = 0.16). Five ferric carboxymaltose -treated patients reported severe AEs (in four, the events were considered drug-related)
Kulnigg et al. (2008)[25]	200	Inflammatory bowel disease	Hb ≤10 g/dl; TSAT <20 % or serum ferritin <100 µg/l	Intravenous ferric carboxymaltose (maximum 1000 mg repeated weekly until deficit was corrected)	Oral ferrous sulphate (100 mg; twice daily; 12 weeks)	The median Hb improved from 8.7 to 12.3 g/dl (ferric carboxymaltose) vs. from 9.1 to 12.1 g/dl (ferrous sulphate, *p* = 0.70). Median ferritin increased from 5.0 to 43.5 µg/l (ferric carboxymaltose) vs. from 6.5 to 28.5 µg/l (ferrous sulphate)	Treatment-related AEs occurred in 28.5 % (ferric carboxymaltose) and 22.2 % (ferrous sulphate) of patients, respectively. Most commonly reported (>2 % of patients overall) treatment-related AEs for ferric carboxymaltose and ferrous sulphate respectively were abdominal pain (2.9 vs. 3.2 %), nausea (2.2 vs. 4.8 %), headache (2.9 vs. 1.6 %) and diarrhoea (0.7 vs. 6.3 %)
Onken et	2584	Non-dialysis-	Hb ≤11.5 g/dl;	Intravenous	Intravenou	The mean Hb increase was	During the study, at least one drug-

**al. (2014)[54]**		**dependent CKD**	**glomerular filtration rate (GFR) <60 ml/min/1.73 m^2**	**ferric carboxymaltose (750 mg; twice weekly)**	**s iron sucrose (200 mg; five infusions in 14 days)**	**1.13 g/dl (ferric carboxymaltose) vs. 0.92 g/dl (iron sucrose; 95 % CI 0.13–0.28). More patients in the ferric carboxymaltose group achieved Hb increase ≥1.0 g/dl (48.6 vs. 41.0 %; 95 % CI 3.6–11.6)**	**related TEAE occurred in 298 of 1276 (23.4 %) subjects in ferric carboxymaltose group and 202 of 1285 (15.7 %) subjects in the iron sucrose group. The most common events were nausea (8.6 % in ferric carboxymaltose group vs. 1.6 % in iron sucrose), hypertension (4.6 vs. 2.0 %), flushing (3.0 vs. 0.1 %), dizziness (2.4 vs. 1.2 %) and dysgeusia (2.4 vs. 1.2 %). The majority of drug-related TEAEs were mild or moderate in severity. At least one serious AE was experienced by 202 of 1276 (15.8 %) participants receiving ferric carboxymaltose and 197 of 1285 (15.3 %) participants receiving iron sucrose (*p* = 0.74), with congestive heart failure being the most commonly reported (2.4 vs. 2.3 %; *p* = 0.90)**
**Onken et al. (2014)[55]**	**507 (A vs. B)<br><br>504 (C vs. D)**	**Iron deficiency anaemia (various conditions)**	**Hb ≤11 g/dl; ferritin <100 ng/ml (or <300 ng/ml when TSAT <30 %)**	**(A) or (C) intravenous ferric carboxymaltose (750 mg; twice weekly)**	**(B) Oral ferrous sulphate (325 mg; three times a day; 14 days); (D) intravenous iron standard-of-care (IVSC)**	**Mean (±SD) Hb was significantly higher in group A than in group B: 1.57 (±1.19) vs. 0.80 (±0.80) g/dl (*p* = 0.001). Group C vs. group D: Hb = 2.90 (±1.64) vs. 2.16 (±1.25) g/dl (*p* = 0.001)**	**During the treatment phase, at least one drug-related TEAE was experienced by 22.8 % of participants in Group A, 6.3 % in Group B, 25.3 % in Group C, and 26.5 % in Group D. The most common events were hypophosphatemia (Group C 5.5 %; Group A 3.7 %), nausea (Group A 4.1 %; Group D 3.3 %), protocol-defined hypotension (Group D 3.7 %), and constipation (Group B 3.2 %). Serious events were described in eight (3.3 %) patients in group A, 10 (4.0 %) in group B, 17 (6.7 %) in group C, and 16 (6.5 %) in group D. Hypersensitivity events were reported in eight participants: three (0.8 %) were in group A, two (0.8 %) were in group C and six (2.4**

							%) in group D
Quinibi et al. (2011)[56]	55	Non-dialysis-dependent CKD	Hb ≤11 g/dl; TSAT ≤25 %; serum ferritin ≤300 ng/ml; glomerular filtration rates ≤45 ml/min/1.73 m^2	Intravenous ferric carboxymaltose (1000 mg; up to two additional doses at two-week intervals)	Oral ferrous sulphate (325 mg; 3 times daily; 56 days)	Mean increase in Hb was 0.95 ± 1.12 (ferric carboxymaltose) vs. 0.50 ± 1.23 g/dl (ferrous sulphate; *p* = 0.005); mean increase in ferritin was 432 ± 189 vs. 18 ±45 ng/ml (*p* < 0.001); mean increase in TSAT was 13.6 ± 11.9 % vs. 6.1 ± 8.1 % (*p* < 0.001)	The proportion of subjects who experienced at least one possibly drug-related AE was significantly lower in ferric carboxymaltose group compared with the oral iron group: 2.7 % in ferric carboxymaltose group and 26.2 % in the oral iron group (*p* = 0.0001). The most commonly AEs the ferric carboxymaltose group were peripheral oedema (6.1 %), hyperkalaemia (4.1 %), urinary tract infection (3.4 %), hypotension (3.4 %), bronchitis, headache and infusion site reaction (2.0 % each). The most commonly experienced AEs in the oral iron group were constipation (17.5 %), nausea (4.9 %), diarrhoea, upper respiratory tract infection (3.9 % each), discoloured faeces and gastrointestinal haemorrhage (2.9 % each). Serious AEs were recorded in 13 (8.8 %) subjects in ferric carboxymaltose group and ten (9.7 %) in the oral iron group
Schatz et al. (2013)[57]	50	Apheresis-related anaemia	Serum ferritin <100 µg/l; or ferritin <300 µg/l; TSAT <20 %	Intravenous ferric carboxymaltose (500–1000 mg; single infusion)	Intravenous ferric gluconate (62.5 mg; once weekly until deficit was corrected)	Serum ferritin (mean) at week 8: 117.9 µg/l in ferric gluconate vs. 140.2 µg/l in ferric carboxymaltose (*p* = 0.4). TSAT: 22 % (ferric gluconate) vs. 26.4 % (ferric carboxymaltose; *p* = 0.02)	There were no serious AEs in either group, especially no anaphylactic shocks. Six patients per group showed AEs, mainly gastrointestinal distress and flu-like symptoms. All AEs were mild, transient and self-limiting. There were no injection site reactions in either group

Seid et al. (2008)[58]	89	Post-partum anaemia	Hb ≤10 g/dl	Intravenous ferric carboxymaltose (1000 mg; weekly for a maximum of 2500 mg)	Oral ferrous sulphate (325 mg; 3 times daily; 6 weeks)	Ferric carboxymaltose -treated patients were significantly more likely to achieve Hb >12 g/dl (91 vs. 67 %; $p < 0.0001$) in a shorter time period (14 vs. 27 days; $p < 0.0002$) and attain higher TSAT (36 vs. 28 %) and ferritin (647 vs. 12 ng/ml) levels ($p < 0.0001$)	Patients reporting one or more drug-related AEs were lower in ferric carboxymaltose (10.6 %) than in oral ferrous sulphate (21.8 %) group. Urticaria (2 %) was the only AE occurring in ≥2 % of the ferric carboxymaltose -treated patients. Four (2.8 %) subjects in ferric carboxymaltose group and four (2.7 %) subjects in the oral iron group experienced at least one serious AE, none of which was considered to be related to study medication or led to premature discontinuation
Van Wyck et al. (2009)[59]	477	Uterine bleeding	Hb ≤11 g/dl; serum ferritin ≤100 ng/ml; TSAT ≤25 %	Intravenous ferric carboxymaltose (maximum 1000 mg repeated weekly to achieve a total replacement dose)	Oral ferrous sulphate (325 mg; 3 times daily; 6 weeks)	Compared to ferrous sulphate, more patients assigned to ferric carboxymaltose achieved correction (Hb ≥12 g/dl) of anaemia (73 % vs. 50 %; $p < 0.001$) and experienced greater improvement in symptoms of fatigue ($p < 0.05$)	No hypotensive or serious drug-related AEs were reported in either treatment group. There were no deaths. Patients assigned to oral iron therapy were more likely to experience drug-related gastrointestinal complaints, particularly constipation (14.2 vs. 3.0 %), diarrhoea (4.4 vs. 1.7 %), nausea (11.9 vs. 3.5 %), and vomiting (3.1 vs. 0.4 %). Patients assigned to intravenous iron therapy were more likely to report transient fatigue (2.2 vs. 0 %), headache (6.5 vs. 4.4 %), dizziness (2.2 vs. 0.4 %), dysgeusia (2.6 vs. 0.9 %), and rash (2.2 vs. 0 %)
Van Wyck et al. (2007)[18]	52	Post-partum anaemia	Hb ≤10 g/dl	Intravenous ferric carboxymaltose (maximum 1000 mg repeated	Oral ferrous sulphate (325 mg; 3 times daily; 6	Patients assigned to intravenous ferric carboxymaltose compared with those assigned to oral iron were more likely to achieve Hb >12 g/dl (90.5 vs. 68.6	No serious drug-related AEs occurred in either treatment group. Patients assigned to oral iron therapy were more likely to report gastrointestinal complaints, particularly constipation (11.2 vs. 3.4 %; $p = 0.07$), diarrhoea (3.9 vs. 0

				**weekly to achieve a total replacement dose)**	**weeks)**	**%; *p* < 0.001)**	**%; *p* = 0.015) and nausea (7.3 vs. 1.1 %; *p* = 0.006) in comparison to ferric carboxymaltose. Patients assigned to intravenous ferric carboxymaltose were more likely to experience skin disorders (5.2 vs. 2.2 %; *p* = 0.164), principally mild pruritus and rash that usually resolved within 15 min from infusion**

REVAMP PROTOCOL Version 6.0
Protocol used at trial close

SUMMARY OF PROPOSED CHANGES TO REVAMP PROTOCOL VERSION 5.0 TO VERSION 6.0

1. Summary of main adjustments to protocol, by section:

Section	Version 5.0 text	Changes made for version 6.0	Reason	Main justification for protocol amendment
2.1.2		*Inclusion of<br>14. Dr Louise Randall[10]<br>15. Dr Mark Seal[3,11]<br>16. Dr Peter Simm[11]*	Additional co-investigators	Co-investigators related to the addition of the REVAMP-Bone and REVAMP-Neuro Substudies.
2.1.3		*Inclusion of<br>18. Ms Sabine Braat[3,4,10]<br>19. Dr Rebecca Harding[10]*	Additional Trial Statisticians	Trial Statisticians
2.2		*Inclusion of<br>10. Walter and Eliza Hall Institute for Medical Research, Melbourne, Australia<br>11. Murdoch Children's Research Institute, Melbourne, Australia*	Additional Institutions related to the new co-investigators	Addition of the Institutions related to the new co-investigators
3.0	*Primary Objective: To determine whether FCM up to 1000 mg given once during the second trimester is superior to oral iron as administered by routine health services during pregnancy in the reduction of maternal anaemia at term.*	*Primary Objective: To determine whether FCM up to 1000 mg given once during the second trimester is superior to oral iron as administered by routine health services during pregnancy in the reduction of maternal anaemia at 36 weeks' gestation.*	Specification of the primary time point for which to determine the impact of FCM.	Clarification of the primary time point for which to determine the impact of FCM.
3.0	*Secondary Objective:<br>To determine if FCM improves maternal*	*Secondary Objective:<br>To determine if FCM*	Listing of all time points for which to determine the	Clarification of all time points for which to

	anaemia at term when compared with routinely delivered oral iron.	*improves maternal anaemia at 4 weeks' post-randomisation, 36 weeks' gestation, delivery, delivery and one-month post-partum when compared with routinely delivered oral iron.*	impact of FCM .	determine the impact of FCM
4.0	*The primary maternal and neonatal outcomes will be the prevalence of anaemia (Hb<11g/dl) at delivery.*	*The primary maternal and neonatal outcomes will be the prevalence of anaemia (Hb<11g/dl) at 36 weeks' gestation.*	Specification of the primary time point for which to determine the impact of FCM.	Clarification of the primary time point for which to determine the impact of FCM.
5.0		*Insertion of new events into the schedule table.*	Additional planned activities at visits due to the addition of the REVAMP-Neuro and REVAMP-Bone Substudies.	Addition of REVAMP-Neuro and REVAMP-Bone Substudies
8.1.1	*To determine whether FCM up to 1000mg given once during the second trimester is superior to oral iron as administered by routine health services during pregnancy in the reduction of maternal anaemia at term.*	*To determine whether FCM up to 1000mg given once during the second trimester is superior to oral iron as administered by routine health services during pregnancy in the reduction of maternal anaemia at 36 weeks' gestation.*	Specification of the primary time point for which to determine the impact of FCM.	Clarification of the primary time point for which to determine the impact of FCM.

8.1.2	*To determine the effectiveness of FCM on maternal anaemia at delivery, when compared with routinely delivered oral iron.*	*To determine the effectiveness of FCM on maternal anaemia at 36 weeks' gestation, when compared with routinely delivered oral iron.*	Specification of the primary time point for which to determine the impact of FCM.	Clarification of the primary time point for which to determine the impact of FCM.
8.1.2		*To determine the impact of FCM on maternal bone health and on infant phosphate regulation and infant bone health (sub-set of 200 mothers and their infants).*	Addition of REVAMP-Bone	Addition of REVAMP-Bone
8.2.1	*Prevalence of maternal anaemia (Hb <11.0g/dl) at delivery.*	*Prevalence of maternal anaemia (Hb <11.0g/dl) at 36 weeks' gestation.*	Specification of the primary time point for which to determine the impact of FCM.	Clarification of the primary time point for which to determine the impact of FCM.
8.2.2	*The incidence of severe medical events: haemorrhage, sepsis, shock, need for transfusion, ICU or prolonged admission, or mortality, both individually and as a composite outcome.*	*The incidence of severe medical events: haemorrhage, need for transfusion, ICU or prolonged admission, or mortality, both individually and as a composite outcome.*	Removal of sepsis and shock as clinical safety outcomes	Sepsis and shock can be extremely hard to diagnose clinically. We want to ensure that the data used for the safety analysis is reliable and of high-quality.

8.2.3	*Birth weight – measured in grams within 24 hours of birth*	*Birth weight and birth length – measured in grams and centimetres within 24 hours of birth*	The birth length measurement is required for subsequent infant biometric analyses.	To align with standard definitions.
8.2.3	*Gestation duration (in days)*	*Gestation duration (in weeks)*	Gestation duration (in weeks) is the standard accepted definition.	To align with standard definitions.
8.2.3		*Infant neurodevelopment at 12 months of age (brain structure parameters measured by low field portable MRI technology) in a sub-set of 60 infants.*	Addition of the REVAMP-Neuro	Addition of the REVAMP-Neuro
8.2.4	*Prevalence of hypophosphatemia (clinical and biochemical) at 4 weeks' post randomisation and at 36 weeks' gestation*	*Prevalence of hypophosphatemia (clinical and biochemical) at baseline, 4 weeks' post randomisation, 36 weeks' gestation and at 3 months postpartum.*	The additional timepoints give a more complete measurement of hypophosphatemia.	Extended examination of prevalence of hypophosphatemia

8.2.4		*Prevalence of bone health dysregulation (determined by bone health markers) at baseline, 4 weeks' post randomisation, 36 weeks' gestation, 3 months post-partum and at 6 months post-partum.*	Addition of REVAMP-Bone	Addition of REVAMP-Bone
8.2.5		*Prevalence of hypophosphatemia at 3, 6, 9 and 12 months of age*<br><br>*Prevalence of bone health dysregulation (determined by bone health markers) at 3, 6, 9 and 12 months of age*<br><br>*Prevalence of radiological Rickets at 6 and 12 months of age*	Addition of REVAMP-Bone	Addition of REVAMP-Bone
13.2.3	*A 10ml venous blood sample will be collected for Full blood count (FBC), iron (ferritin, sTfR) phosphate and inflammatory markers (C-reactive protein and alpha-1 glycoprotein) and Treponema pallidum.*	*A 10ml venous blood sample will be collected for Full blood count (FBC), iron (ferritin, sTfR), hepcidin, phosphate, markers of bone health (e.g., vitamin D, calcium, alkaline phosphatase (ALP),*	Addition of bone health markers as part of REVAMP-Bone	Addition of REVAMP-Bone

		procollagen type 1 N-terminal propeptide (P1NP), C1 Telopeptide, parathyroid hormone (PTH) and fibroblast growth factor-23 (FGF23)), inflammatory markers (C-reactive protein and alpha-1 glycoprotein) and Treponema pallidum.		
13.3.3	*A 10ml venous blood sample for FBC, iron indices (ferritin, sTfR), CRP, alpha-1 glycoprotein, phosphate, Treponema pallidum and malaria parasitaemia (using RDT and microscopy).*	*A 10ml venous blood sample for FBC, iron indices (ferritin, sTfR), CRP, alpha-1 glycoprotein, hepcidin, phosphate, markers of bone health (e.g., calcium, alkaline phosphatase (ALP), procollagen type 1 N-terminal propeptide (P1NP), C1 Telopeptide, parathyroid hormone (PTH) and fibroblast growth factor-23 (FGF23)), Treponema pallidum and malaria parasitaemia (using RDT and microscopy).*	Addition of bone health markers as part of the REVAMP-Bone	Addition of REVAMP-Bone

13.5.3	*A 10ml venous blood sample will be collected for FBC, iron indices (ferritin, sTfR), CRP alpha-1 glycoprotein, phosphate, Treponema pallidum, malaria parasitaemia (using RDT and microscopy).*	*A 10ml venous blood sample will be collected for FBC, iron indices (ferritin, sTfR), CRP alpha-1 glycoprotein, hepcidin, phosphate, markers of bone health (e.g., calcium, alkaline phosphatase (ALP) and fibroblast growth factor-23 (FGF23)), Treponema pallidum, malaria parasitaemia (using RDT and microscopy).*	Addition of bone health markers as part of REVAMP-Bone	Addition of REVAMP-Bone
13.8.11		*Consent for Involvement in the REVAMP-Bone Substudy*<br>*After provision of detailed information and the opportunity to ask questions (and to discuss with their family), mothers (N=200) will be invited to provide written informed consent for their participation and the participation of their infant (N=200) in the REVAMP-Bone Substudy. Participants providing consent will continue through the study visits described*	Addition of REVAMP-Bone	Addition of REVAMP-Bone

		below.		
13.8.12		*Consent for involvement in the REVAMP-Neuroimaging Substudy.* *After provision of detailed information and the opportunity to ask questions (and to discuss with their family), mothers (N=60) will be invited to provide written informed consent for the participation of their inflant (N=60) in the REVAMP-Neuroimaging Substudy. Participants providing consent will continue through the study visits described below.*	Addition of REVAMP-Neuro	Addition REVAMP-Neuro
13.9.3	*Maternal venous blood sample (10ml) will be collected for FBC, iron indices (ferritin, sTfR), CRP alpha-1 glycoprotein, and malaria RDT, microscopy and filter paper for malaria PCR for resistance testing only for positive samples.*	*Maternal venous blood sample (10ml) will be collected for FBC, iron indices (ferritin, sTfR), CRP, alpha-1 glycoprotein, hepcidin, phosphate, markers of bone health (e.g.,*	Addition of bone health markers as part of REVAMP-Bone	Addition of REVAMP-Bone

		calcium, alkaline phosphatase (ALP), procollagen type 1 N-terminal propeptide (P1NP), C1 Telopeptide, parathyroid hormone (PTH) and fibroblast growth factor-23 (FGF23)), and malaria RDT, microscopy and filter paper for malaria PCR for resistance testing only for positive samples.		
13.9.3	*Similarly, a venous blood sample of up to 3ml will be collected from the infant for Hb, malaria RDT, microscopy and filter paper for PCR, iron indices determination and immunological assessment.*	*Similarly, a venous blood sample of up to 3ml will be collected from the infant for Hb, malaria RDT, microscopy and filter paper for PCR, iron indices determination, hepcidin, phosphate, markers of bone health (e.g., vitamin D, calcium, alkaline phosphatase (ALP), and fibroblast growth factor-23 (FGF23)) and immunological assessment.*	Addition of bone health markers as part of REVAMP-Bone	Addition of REVAMP-Bone

13.10.3	*Maternal venous blood sample (10ml) will be collected for FBC, iron indices (ferritin, sTfR), CRP alpha-1 glycoprotein, and malaria RDT, microscopy and filter paper for malaria PCR for resistance testing only for positive samples.*	*Maternal venous blood sample (10ml) will be collected for FBC, iron indices (ferritin, sTfR), CRP alpha-1 glycoprotein, phosphate, markers of bone health (e.g., calcium and alkaline phosphatase (ALP)) and malaria RDT, microscopy and filter paper for malaria PCR for resistance testing only for positive samples.*	Addition of bone health markers as part of REVAMP-Bone	Addition of REVAMP-Bone
13.10.3	*Similarly, a venous blood sample of up to 3ml will be collected from the infant for Hb, malaria RDT, microscopy and filter paper for PCR, iron indices determination and immunological assessment.*	*Similarly, a venous blood sample of up to 3ml will be collected from the infant for Hb, malaria RDT, microscopy and filter paper for PCR, iron indices determination, hepcidin, phosphate, markers of bone health (e.g., calcium, alkaline phosphatase (ALP), vitamin D and fibroblast growth factor-23 (FGF23)) and immunological assessment.*	Addition of bone health markers as part of REVAMP-Bone	Addition of REVAMP-Bone

13.10.10		*Child bone health*<br><br>*In a subset of children who have been consented for the REVAMP-Bone Substudy (N=200), we will take x-rays of wrist and knee to identify the presence of radiological Ricketts using standard techniques. The use of x-rays is a safe and non-invasive method for the assessment of bones.*	Addition of REVAMP-Bone	Addition of REVAMP-Bone
13.11.3	*Similarly, a venous blood sample of up to 3ml will be collected from the infant for Hb, malaria RDT, microscopy and filter paper for PCR, iron indices determination and immunological assessment.*	*Similarly, a venous blood sample of up to 3ml will be collected from the infant for Hb, malaria RDT, microscopy and filter paper for PCR, iron indices determination, hepcidin, phosphate, biological markers of bone health (e.g., calcium and fibroblast growth factor-23 (FGF23)) and immunological assessment.*	Addition of bone health markers as part of REVAMP-Bone	Addition of REVAMP-Bone

13.12.3	*Similarly, a venous blood sample of up to 3ml will be collected from the infant for Hb, malaria RDT, microscopy and filter paper for PCR, iron indices determination and immunological assessment.*	*Similarly, a venous blood sample of up to 3ml will be collected from the infant for Hb, malaria RDT, microscopy and filter paper for PCR, iron indices determination, hepcidin. phosphate, biological markers of bone health (e.g., calcium, fibroblast growth factor-23 (FGF23), vitamin D and alkaline phosphatase (ALP)) and immunological assessment.*	Addition of bone health markers as part of REVAMP-Bone	Addition of REVAMP-Bone
13.12.7		*In a subset of infants (N=60), we will measure structural neurodevelopment using low field portable MRI technology (see Neurocognitive Assessments used in Infant Follow-Up section below).*	Addition of REVAMP-Neuro	Addition of REVAMP-Neuro
13.12.8		*Child bone health*<br><br>*In a subset of children who have been consented for the REVAMP-Bone Substudy (N=200), we will take x-rays*	Addition of REVAMP-Bone	Addition of REVAMP-Bone

		of wrist and knee to identify the presence of radiological Ricketts using standard techniques. The use of x-rays is a safe and non-invasive method for the assessment of bones.		
Table 8		*Addition of hepcidin and markers of bone health in both maternal and infant laboratory tests.*	Addition of bone health markers as part of REVAMP-Bone.<br>Addition of hepcidin as an additional marker of iron regulation.	Addition REVAMP-Bone
15.5		*Low Field portable MRI (Hyperfine)*<br>*Neuroimaging offers an opportunity to assess the impact of high dose iron during gestation on child neurodevelopment.*<br>*At 12 months of age, in a subset of children (N=60), we will obtain structural images of the brain using low field portable MRI technology (Hyperfine). Low field portable MRI technology for neuroimaging has been established by Hyperfine, and has been adapted for*	Addition of REVAMP-Neuro	Addition of REVAMP-Neuro

		low-income settings. Images will be analysed using software developed by international partnerships. Images will be taken in Zomba, Malawi. Data will be uploaded and shared with our team in Melbourne and analysed by the Developmental Imaging team at the Murdoch Children's Research Institute, Melbourne. All analyses will be undertaken by staff blinded to the intervention arm and other exposures of the participant. In addition, experienced paediatric radiologists from the Royal Children's Hospital, Melbourne, will provide a clinical MRI report for every scan undertaken.		
19.1	*Sample size is calculated based on the maternal primary outcome of anaemia at delivery.*	*Sample size is calculated based on the maternal primary outcome of anaemia at 36 weeks' gestation.*	Specification of the primary time point for which to determine the impact of FCM.	Clarification of the primary time point for which to determine the impact of FCM.

19.2.1	*The primary maternal outcome anaemia at pre-delivery (based on the capillary Hb closest prior to delivery) will be analysed using a binomial log-linear regression, with the oral iron group as the reference, and incorporating clinic and treatment in the model. The primary maternal hypothesis will be evaluated by obtaining the estimate of the risk ratio of IV iron versus oral iron and a 95% confidence interval.*	*The primary maternal outcome anaemia will be analysed using a binomial log-linear regression, with the oral iron group as the reference, and incorporating clinic and treatment in the model. The primary maternal hypothesis will be evaluated by obtaining the estimate of the risk ratio of IV iron versus oral iron and a 95% confidence interval at 36 weeks' gestation.*	Clarification that the statistical model will analyse anaemia collected during the trial and obtain the effect size for anaemia at 36 week's gestation to evaluate the primary hypothesis.	To account for the repeated measurements of anaemia
19.2.1	Other secondary binary outcomes will be analysed similarly as the primary maternal outcome and other secondary continuous outcomes as the birthweight outcome where applicable also taking into account in the model the repeated data collection of the outcome.	Other secondary binary outcomes will be analysed similarly as the primary maternal outcome and other secondary continuous outcomes as the birthweight outcome. Where applicable, models will take into account the repeated data collection of the outcome.	Clarification that all models will account for repeated data collection, when applicable.	To account for the repeated measurements of anaemia

19.4	*Multiple imputations under the missing at random assumption will be performed as a secondary analysis for handling missing data in the primary maternal and neonate outcome; results will be compared with the primary complete case analysis to investigate the robustness of the findings to missing data.*	*Multiple imputations under the missing at random assumption will be performed as a secondary analysis for handling missing data in the primary maternal and neonate outcome; results will be compared with the primary analysis to investigate the robustness of the findings to missing data.*	Clarifying of the primary analysis to which to compare multiple imputation.	To account for the repeated measurements of anaemia

2. Minor editing changes/corrections have also been made to the protocol and have been highlighted (or struck out) in the attached documents.

PROJECT PROPOSAL

Randomized controlled trial of the Effect of intraVenous iron on Anaemia in Malawian Pregnant women

Short Title: **REVAMP Study**

Study Identifiers:

**COMREC: [P.02/18/2357]**	**WEHI REC: 18/02**	**PMPB: PMRA/CTRC/25052018100**

Co-Principal Investigators:

Prof Kamija Phiri, Department of Public Health, College of Medicine, University of Malawi, Private Bag 360, Chichiri, Blantyre 3, Malawi. Mobile: +265 999957 048; E-mail: kamijaphiri@gmail.com

Dr Sant-Rayn Pasricha, Walter and Eliza Hall Institute of Medical Research, 1G Royal Parade, Parkville, Melbourne 3052, Australia. Mobile: +61407141570; E-mail: Pasricha.s@wehi.edu.au

Co-Investigators: **See Page 3**

Institutions: **See Page 3**

Funder: **Bill & Melinda Gates Foundation**

Amendment History:			
Date	Protocol Version	Details of Changes	Signature of Principal Investigator
31 Jan 2018	1.1	Original approved Protocol	
26 Jul, 2018	2.0	First amendment – number of follow-up visits reduced from 17 to 8	
28 Aug, 2018	2.1	Resubmission of first amendment - Amendments approved	
31 Dec, 2018	3.0	Second Amendment – extension of follow up from 1 month postpartum to 12 months postpartum	
24 May 2019	3.1	Resubmission of second amendment – amendments approved	
22 May 2020	4.0	Adjustment to COVID-19 pandemic in response to COMREC Covid 19 Research Guidelines.	
30 July 2020	5.0	Adjustment to the statistical analysis section of the protocol and to the Maternal Psychological Health assessment tool. Inclusion of additional testing for maternal breast milk and malaria parasite parameters.	
November 2021	6.0	Addition of Bone Substudy – REVAMP-Bone. Expansion of testing for bone health markers, as well as radiological examination of infants.<br><br>Addition of Neuroimaging Substudy – REVAMP-Neuro. Addition of neurodevelopmental assessment tool (low field Magnetic Resonance Imaging).	

1. TITLE OF RESEARCH PROPOSAL

Randomized controlled trial of the effect of intravenous iron on anaemia in Malawian pregnant women

2. INVESTIGATORS AND INSTITUTIONAL AFFILIATIONS

2.1. INVESTIGATORS

2.1.1. *Co-principal Investigators*

- **Prof Kamija Phiri** — **Department of Public Health, College of Medicine, University of Malawi, Private Bag 360, Chichiri, Blantyre 3, Malawi. Mobile: +265 999957 048; E-mail: kphiri@medcol.mw**
- **Dr Sant-Rayn Pasricha** — **Walter and Eliza Hall Institute of Medical Research, The University of Melbourne, 1G Royal Parade, Parkville, Melbourne 3052, Australia. Mobile: +61 407 141 570; E-mail: pasricha.s@wehi.edu.au**

2.1.2. *Co-investigators*

13. **Professor Bjarne Robberstaad[1]**
14. **Professor Khalid Khan[2]**
15. **Professor Stephen Rogerson[3]**
16. **Professor William Stones[5]**
17. **Dr Kabeya Biselele[6]**
18. **Dr Martin Mwangi[5]**
19. **Dr Jobiba Chinkhumba[9]**
20. **Dr Glory Mzembe, MD[5] (PhD student)**
21. **Mr Ernest Moya[5] (PhD student)**
22. **Dr Leila Larson[3]**
23. **Dr Stefan Bode[3]**
24. **Dr Katherine Johnson[3]**
25. **Dr Ricardo Ataide[3]**
26. **Dr Louise Randall[10]**
27. **Dr Mark Seal[3,11]**
28. **Dr Peter Simm[11]**

2.1.3. *Trial Statisticians*

29. **Professor Julie Simpson[4]**
30. **Ms Sabine Braat [3,4,10]**
31. **Dr Rebecca Harding[10]**

2.1.4. *Collaborators*

32. **District Health Officer, Blantyre[5]**
33. **District Health Officer, Zomba[6]**

2.2. INSTITUTIONS

10. **Centre for International Health, University of Bergen, Bergen, Norway**
11. **Queen Mary University of London, London, UK**
12. **The University of Melbourne, Melbourne, Australia**
13. **Centre for Epidemiology and Biostatistics, Melbourne School of Population and Global Health,**

The University of Melbourne, Melbourne, Australia
14. Department of Public Health, College of Medicine, Blantyre, Malawi
15. Zomba Central Hospital, Zomba, Malawi
16. Blantyre District Assembly, Blantyre, Malawi
17. Zomba District Assembly, Zomba, Malawi
18. Malaria alert centre, College of Medicine, Blantyre, Malawi
19. Walter and Eliza Hall Institute for Medical Research, Melbourne, Australia
20. Murdoch Children's Research Institute, Melbourne, Australia

3. PROTOCOL ABSTRACT

Aim	**To determine whether IV ferric carboxymaltose (FCM) given once during the second trimester is effective and safe in improving maternal, neonatal and infant outcomes for treatment of moderate to severe maternal anaemia among pregnant women in Blantyre and Zomba districts of Malawi.**
Research Objectives	***Primary objective*: To determine whether FCM up to 1000 mg given once during the second trimester is superior to oral iron as administered by routine health services during pregnancy in the reduction of maternal anaemia at 36 weeks' gestation.**<br>***Secondary Objectives***<br>**7. To determine if FCM improves maternal anaemia at 4 weeks' post-randomisation, 36 weeks' gestation, ~~term~~ delivery and one month post-partum when compared with routinely delivered oral iron.**<br>**8. To determine the effectiveness of FCM compared to routinely delivered oral iron on critical neonatal outcomes including birth weight (low birth weight), gestation duration (prematurity), small for gestational age and other adverse birth outcomes.**<br>**9. To determine the effectiveness of FCM compared to routinely delivered oral iron on maternal outcomes including haemoglobin concentration and iron deficiency (measured through iron biomarkers), maternal quality of life and maternal cognitive function.**<br>**10. To determine the safety of FCM compared to oral iron on the prevalence of all-cause sick visits, serious maternal adverse events, clinical malaria, bacteraemia, placental malaria, malaria parasitaemia, hypophosphatemia and pathogenic vaginal bacterial flora during pregnancy.**<br>**11. To determine immune and molecular aetiologies that may be related to anaemia, iron deficiency and infection in pregnancy.**<br>**12. To determine the cost-effectiveness of FCM compared to oral iron measured through absolute and incremental health benefits and incremental provider and patient costs.**<br>**13. To determine the effectiveness of FCM compared to oral iron in**

	improving infant haematological, iron status, cognitive, growth and development, and immunological outcomes.<br>14. To determine the long-term effects of FCM compared to oral iron on maternal wellbeing after giving birth
Study Design	**30-month, Phase III open-label 2-arm parallel group randomized controlled trial recruiting pregnant women in their second trimester and following them up until 12 months postpartum.**<br>**This is an effectiveness trial and will be carried out in real-life settings in health facilities in Blantyre and Zomba district.**
Intervention	**Intervention group will receive IV ferric carboxymaltose 1000mg (for women with weight >50kg), or 20mg/kg (for women with weight <50kg) once during the second trimester.**<br>**Control group will receive oral iron 200mg ferrous sulphate (approx. 65mg elemental iron) twice daily for 90 days or the duration of pregnancy, whichever is shorter.**
Primary outcome	**Prevalence of maternal anaemia (Hb <11.0g/dl) at 36 weeks' gestation**
Target Population	***Inclusion criteria***<br>1. Confirmed singleton pregnancy at 13-26 weeks' gestation<br>2. Moderate to severe anaemia not requiring an immediate blood transfusion (Hb <10g/dl)<br>3. Negative malaria parasitaemia<br>4. Resident in the study catchment area of Blantyre and Zomba district<br>5. Plan to deliver at a health facility<br>6. Written informed consent (including assent if <18 years old)<br>***Exclusion criteria***<br>1. Hypersensitivity to any of the study drugs<br>2. Clinical symptoms of malaria or bacterial infection<br>3. Any condition requiring hospitalization or serious concomitant illness<br>4. Chronic illness that may adversely affect foetal growth and viability<br>5. Clinically low haemoglobin requiring a blood transfusion (usually Hb <5g/dl)<br>6. Pre-Eclampsia (at screening)
Sample size	**862 women (431 per arm)**

Primary data collection & sampling method	**Outcome assessments will be measured through:**<br>**4. Scheduled and unscheduled participant visits to health facilities**<br>**5. Laboratory molecular and biological assays**<br>**6. Economic evaluation for incremental costs determination**

3.1.

4. EXECUTIVE SUMMARY

Type of study: Open label two-arm Phase III individual-randomized controlled trial

Problem: Anaemia in pregnancy remains a critical global health problem, affecting 46% of pregnant women in Africa and 49% in Asia. Antenatal anaemia is associated with critical risks for both mother and child. Control of maternal anaemia, an important contributor to maternal mortality, is a key WHO nutrition target and component of the third Sustainable Development Goal. Treatment with oral iron is poorly tolerated and requires extended contact with a well-functioning health system; this is difficult to achieve and few women receive full courses of iron. A new intravenous (IV) iron formulation, ferric carboxymaltose, now routinely available and used in developed countries, is safe and provides the opportunity to give high doses of iron in a single rapid infusion. In low and middle-income countries (LMICs), intravenous iron could present a novel, innovative opportunity to rapidly cure moderate and severe antenatal anaemia, thereby improving crucial maternal and neonatal outcomes.

Objectives: To determine whether IV ferric carboxymaltose compared to standard oral iron distributed through routine antenatal services is effective and safe for treatment of moderate and severe antenatal anaemia in pregnancy, in settings where resources are limited, distribution and adherence to antenatal oral iron is challenging, and the burden of maternal anaemia, adverse neonatal outcomes, and infection are high.

Methods: This is an open label two-arm Phase III randomized controlled trial in anaemic pregnant women with the primary maternal outcome of recovery from anaemia and the important secondary neonatal outcome of birth weight, assessed by visits over pregnancy, at birth, and follow-up to 12 months' post-partum. The study will have two arms: (a) IV iron plus Intermittent Preventive Treatment in pregnancy (IPTp) and (b) Oral iron – routine care plus IPTp. The IV iron (1000mg for women with weight >50kg, and 20mg/kg for women with weight <50kg) will be given once at baseline while the oral iron (65mg elemental iron twice daily) will be given for 90 days (or the duration of pregnancy, whichever is shorter). Both arms will receive sulfadoxine pyrimethamine (SP) as IPTp according to national guidelines and participants will be followed up after 4 weeks, then bi-weekly (at home to measure Hb) from 34 weeks' gestation, as well as clinic visits at 36 weeks' gestation, at delivery and at 1 month postpartum. The primary maternal and neonatal outcomes will be the prevalence of anaemia (Hb<11g/dl) at 36 weeks' gestation ~~delivery.~~ The planned number of randomized participants is 862 pregnant women (431 per arm). The study has 80% power at a two-sided alpha level of 5% and incorporating a 10% loss to follow up to result to a 10% absolute improvement in anaemia cure (to a prevalence of 46%). The study will be based in antenatal clinics and centres in urban and semi-urban areas of Blantyre and Zomba in Malawi. The trial will be implemented by an international team of Malawian, Australian, UK, and Norwegian experts in clinical trials, obstetrics, anaemia, malaria, and health economics.

Anaemia in pregnancy may influence longer-term health of the mother and her baby. The investigators have received additional funding to extend the follow up period of the women and babies in order to assess longer-term benefits and safety of this approach. Women and babies will be followed up until 12-months post-partum (infant age 12 months), and will undergo extra study visits at 3, 6, 9 and 12 months. Mothers and babies will undergo testing for haematologic, iron, nutritional, infection and immunologic status. Women will undergo testing for wellbeing and depression, and babies will undergo cognitive testing.

Expected Findings and Implications: Oral iron supplementation remains cheap while the drug cost of ferric carboxymaltose is much higher; as such, demonstration of clinical superiority must be accompanied by analysis of potential cost-effectiveness in LMICs, to justify implementation. If our data demonstrate a clear benefit from intravenous iron on maternal haematologic outcomes and wellbeing, on critical neonatal outcomes such as birth weight and gestation duration, on infant iron status, and perhaps on long term outcomes including child cognitive development, growth, immunologic function, and postpartum maternal wellbeing, they will develop a strong clinical rationale for implementation of this intervention in routine practice. This will be supported by economic modelling which will enable us to determine a value for money for ferric carboxymaltose, which will enable governments and donors to consider the relative priority of scaling up this intervention compared to other maternal and child interventions. We will use this to identify an appropriate cost point at which this intervention would be considered implementable. Thus, results from this trial could transform the way anaemia is treated in LMICs. As a result, our study may lead to the implementation of ferric carboxymaltose as a treatment for maternal anaemia in the medium term. This could have long term benefits for maternal and child health, ultimately resulting in benefits for maternal and child survival and child development.

Dissemination: The results will be presented at local and international fora, submitted for publication in peer-reviewed scientific journals and reported to COMREC and other relevant ethics committees.

5. SCHEDULE OF ACTIVITIES

Table 1: Details of planned activities per visit

Protocol Activity	Visit 0<br>*Day 0*	Visit 1<br>*Day 0*	Visit 2<br>*Day 28 ±2 days*	Visit 3<br>*34 wks ±2 days gestation*	Visit 4<br>*36 wks ±2 days gestation (pre-delivery)*	Visit 5 – 6<br>*38 & 40 wks gestation ± 2 days*	Visit 7<br>*Delivery ±2 days*	Visit 8<br>*Day 28 ± 2 days Post-partum*	Visit 9<br>*3 months ±14 Days Post-partum*	Visit 10<br>*6 months ± 14 Days Post-partum*	Visit 11<br>*9 months ± 14 Days Post-partum*	Visit 12<br>*12 months ± 14 Days Post-partum*	Sick /Un scheduled Visit
Location of visit	HF[i]	HF	HF	Home[j]	HF	Home	HF	HF	HF	HF	HF	HF	HF
Pre-screening form completion	X												
Screening		X											
Informed consent process		X											
Medical & obstetric history		X											
Household economic data form			X										
Household food insecurity									X	X	X	X	
Complete physical examination[a]		X											
Limited physical examination[b]			X		X		X	X	X	X	X	X	X
Ultrasound scan		X	X		X								X
Cost data form completion		X					X						X
Piper Fatigue Scale (PFS) data form completion		X	X		X			X					
Cognitive function tests		X	X					X					
Maternal psychological health: DASS-21 and MIBS data forms completion		X[n]						X	X	X	X	X	
Edinburg postpartum depression scale (EPDS)		X[n]							X				
Self-Reporting Questionnaire		X[n]											
Randomize participant		X											
Administer treatment													
IV iron		X											
Oral iron		X											
Laboratory procedures (Maternal)													
Full Blood Count		X	X		X		X	X	X	X	X	X	X

Protocol Activity	Visit 0<br><br>*Day 0*	Visit 1<br><br>*Day 0*	Visit 2<br><br>*Day 28 ±2 days*	Visit 3<br><br>*34 wks ±2 days gestation*	Visit 4<br><br>*36 wks ±2 days gestation (pre-delivery)*	Visit 5 – 6<br><br>*38 & 40 wks gestation ± 2 days*	Visit 7<br><br>*Delivery ±2 days*	Visit 8<br><br>*Day 28 ± 2 days Post-partum*	Visit 9<br><br>*3 months ±14 Days Post-partum*	Visit 10<br><br>*6 months ± 14 Days Post-partum*	Visit 11<br><br>*9 months ± 14 Days Post-partum*	Visit 12<br><br>*12 months ± 14 Days Post-partum*	Sick /Un scheduled Visit
Location of visit	HF[i]	HF	HF	Home[j]	HF	Home	HF	HF	HF	HF	HF	HF	HF
Capillary Haemoglobin		X	X	X	X	X	X	X	X	X	X	X	X
Malaria RDT		X	X		X		X	X	X	X	X	X	X
Malaria microscopy		X	X		X		X	X	X	X	X	X	X
Malaria filter paper for PCR		X	X		X		X	X	X	X	X	X	X
Serum for iron markers tests[c]		X	X		X		X	X	X	X	X	X	
Serum for inflammatory markers tests[d]		X	X		X		X	X	X	X	X	X	
Phosphate		X	X		X		X	X	X	X			
Serum for bone health markers[o, p]		X	X		X				X	X			
Treponema Pallidum		X	X		X								
Urinalysis			X		X								
Vaginal swab sample collection[e]			X		X								
Stool sample collection & analysis[f]			X		X			X					
Placenta histology							X						
Blood culture													X[k]
Breast milk sample for iron markers								X					
Laboratory procedures (Infant)													
Haemoglobin by Hemocue®							X[m]	X	X	X	X	X	
Full Blood Count							X[m]	X	X	X	X	X	X[l]
Malaria rapid diagnostic test (MRDT)							X[m]	X	X	X	X	X	X
Malaria microscopy							X[m]	X	X	X	X	X	X
Serum for iron markers tests[c]							X[m]	X	X	X	X	X	

Protocol Activity	Visit 0<br><br>*Day 0*	Visit 1<br><br>*Day 0*	Visit 2<br><br>*Day 28 ±2 days*	Visit 3<br><br>*34 wks ±2 days gestation*	Visit 4<br><br>*36 wks ±2 days gestation (pre-delivery)*	Visit 5 – 6<br><br>*38 & 40 wks gestation ± 2 days*	Visit 7<br><br>*Delivery ±2 days*	Visit 8<br><br>*Day 28 ± 2 days Post-partum*	Visit 9<br><br>*3 months ±14 Days Post-partum*	Visit 10<br><br>*6 months ± 14 Days Post-partum*	Visit 11<br><br>*9 months ± 14 Days Post-partum*	Visit 12<br><br>*12 months ± 14 Days Post-partum*	Sick /Un scheduled Visit
Location of visit	HF[i]	HF	HF	Home[j]	HF	Home	HF	HF	HF	HF	HF	HF	HF
Phosphate							X	X	X	X	X	X	
Serum for bone health markers[o,q]									X	X	X	X	
Radiological examination of wrist and knee (x-rays)[o]										X		X	
Malaria filter paper for PCR							X[m]	X	X	X	X	X	
Stool collection							X	X		X		X	
Pregnancy outcome							X						
Complete physical examination of neonate[g]							X						
Limited physical examination of infant[h]								X	X	X	X	X	X[l]
Child anthropometry: weight, length, head circumference							X	X	X	X	X	X	
Child vaccination and Vit A supplementation status								X	X	X	X	X	
Child dietary diversity								X	X	X	X	X	
Stimulating caregiving										X			
Infant neurodevelopment using electroencephalography (EEG) and Bayley Scales of Infant and Toddler Development										X		X	
Infant neurodevelopment using auditory brainstem responses (ABRs)							X	X		X			
Infant neurodevelopment using low filed MRI technology (Hyperfine)[r]												X	
Non-invasive Hb by Rad-67 device		X	X		X								

[a] Complete examination: general appearance, throat, neck, thyroid, musculoskeletal, skin, lymph nodes, extremities, pulses, pulmonary, cardiac, abdominal, and neurological examination
[b] Limited examination: general appearance, brief pulmonary, cardiac, abdominal and neurological examination
[c] Serum ferritin, sTfR; hepcidin
[d] CRP and alpha-1 glycoprotein
[e] Reproductive tract infections: *Chlamydia trachomatis, Neisseria gonorrhoeae, Trichomonas vaginalis,* Bacterial Vaginosis
[f] Stool microscopy and microbiome analysis
[g] Complete examination: weight, length, head circumference, APGAR score, Ballard score, congenital anomaly, and complications at birth
[h] Limited examination: general appearance, brief pulmonary, cardiac, abdominal and neurological examination
[i] Visit will be at health facility
[j] Visit will be at the participant's home
[k] If clinically indicated
[l] If the neonate is unwell
[m] Cord blood
[n] Only in a sub-set of 50 women
[O] Only in a sub-set of 200 women (laboratory procedures (maternal)) and their 200 infants (laboratory procedures (infant))
[p] Vitamin D (Visit 1); calcium (Visits 1, 2, 4, 9 and 10); alkaline phosphatase, ALP (Visits 1, 2, 4, 9 and 10); procollagen type 1 N-terminal propeptide, P1NP (Visits 1, 2 and 9); C1 Telopeptide (Visits 1, 2 and 9); Parathyroid hormone, PTH (Visits 1, 2 and 9); fibroblast growth factor-23, FGF23 (Visits 1, 2, 4 and 9).
[q] Fibroblast growth factor-23, FGF23 (Visits 9, 10, 11 and 12); calcium (Visits 9, 10, 11 and 12); vitamin D (Visits 9, 10 and 12); alkaline phosphatase, ALP (Visits 9, 10 and 12).
[r] Only in a sub-set of 60 children

TABLE OF CONTENTS

1. TITLE OF RESEARCH PROPOSAL 93

2. INVESTIGATORS AND INSTITUTIONAL AFFILIATIONS 93

2.1. INVESTIGATORS 93
2.1.1. Co-principal Investigators *93*
2.1.2. Co-investigators *93*
2.1.3. Trial Statisticians *93*
2.1.4. Collaborators *93*
2.2. INSTITUTIONS 93

3. PROTOCOL ABSTRACT 94

4. EXECUTIVE SUMMARY 97

5. SCHEDULE OF ACTIVITIES 99

6. ABBREVIATIONS 107

7. INTRODUCTION 108

7.1. BACKGROUND 108
7.2. THE RATIONALE FOR THE RESEARCH PROJECT 117

8. TRIAL OBJECTIVES AND END POINTS 118

8.1. OBJECTIVES 118
8.1.1. Broad Objective 118
8.1.2. Specific Objectives 118
8.2. ENDPOINTS 119
8.2.1. Primary Outcome 119
8.2.2. Secondary Outcomes (Benefit in mother) 119
8.2.3. Secondary Outcomes (Benefit in the neonate and infant) 120
8.2.4. Secondary Outcomes (Safety in the mother) 121
8.2.5. Secondary Outcomes (Safety in the infant) 121
8.2.6. Secondary Outcomes (Cost and cost-effectiveness) 122
8.2.7. Secondary Outcomes (diagnostic test accuracy of SpHb) 122

9. STUDY DESIGN 122

10. PARTICIPANT INCLUSION AND EXCLUSION CRITERIA 122

11. STUDY SITES 123

12. TRIAL INTERVENTIONS 123

12.1. ALLOCATION TO TREATMENT 124
12.2. BREAKING THE BLIND 124

13. STUDY PROCEDURES 124

13.1. VISIT 0 [DAY 0]- SCREENING 125
13.2. VISIT 1 [DAY 0]- RECRUITMENT 125
13.2.1. Maternal quality of life and productivity 125
13.2.1.1 Validation of the Piper Fatigue Scale (PFS) 125
13.2.2. Cognitive function 126
13.2.3. Blood sampling 126
13.2.4. SpHb Measurement 126
13.2.5. Study arm allocation 126
13.2.6. Study drug administration 126
13.3. VISIT 2 [DAY 28 ±2]-- 4-WEEK FOLLOW-UP 127
13.3.1. Maternal quality of life and productivity 127
13.3.2. Cognitive function 127

13.3.3. *Blood sampling* 127
13.3.4. *SpHb Measurement* 128
13.3.5. *Reproductive Tract Infections Determination* 128
13.3.6. *Urinalysis*128
13.3.7. *Stool Analysis* 128
13.4. VISIT 3 [34 WEEKS GESTATION ±2 DAYS] 128
13.5. VISIT4 [36 WEEKS GESTATION ±2 DAYS]-- PRE-DELIVERY FOLLOW-UP 128
13.5.1. *Maternal quality of life and productivity* 128
13.5.2. *Cognitive function* 128
13.5.3. *Blood sampling* 129
13.5.4. *SpHb Measurement* 129
13.5.5. *Reproductive Tract Infections Determination* 129
13.5.6. *Urinalysis*129
13.5.7. *Stool analysis* 129
13.6. VISIT 5 -6 [38 AND 40 WEEKS GESTATION ±2 DAYS] 129
13.7. VISIT 7 – DELIVERY [+1 DAY] 129
13.7.1. *Blood sampling* 130
13.7.2. *Placental histology*130
13.7.3. *Cord blood* 130
13.7.4. *Infant neurodevelopment* 130
13.7.5. *Stool sample collection* 130
13.8. VISIT 8-- 28 DAYS POSTPARTUM/POSTNATAL ±2 DAYS] 131
13.8.1. *Maternal quality of life and productivity* 131
13.8.2. *Maternal cognitive function* 131
13.8.3. *Blood sampling* 131
13.8.4. *Child anthropometry* 131
13.8.5. *Child vaccination status* 131
13.8.6. *Infant neurodevelopment* 131
13.8.7. *Child dietary diversity* 131
13.8.8. *Maternal psychological health* 132
13.8.9. *Stool sample collection* 132
13.8.10. *Consent for involvement in the follow up (extension study)* 132
13.8.11. *Consent for Involvement in the REVAMP Bone Substudy* 132
13.8.12. *Consent for involvement in the REVAMP NeuroImaging Substudy* 132
13.9. VISIT 9-- 3 MONTHS POST-PARTUM (+/- 14 DAYS) 132
13.9.1. *Household food insecurity* 132
13.9.2. *Maternal psychological health* 132
13.9.3. *Blood sampling* 133
13.9.4. *Child anthropometry* 133
13.9.5. *Child vaccination status* 133
13.9.6. *Child dietary diversity* 133
13.10. VISIT 10-- 6 MONTHS POST-PARTUM (+/- 14 DAYS) 133
13.10.1. *Household food insecurity* 133
13.10.2. *Maternal psychological health* 133
13.10.3. *Blood sampling* 133
13.10.4. *Child anthropometry* 134
13.10.5. *Child vaccination and vitamin A supplementation status* 134
13.10.6. *Child dietary diversity* 134
13.10.7. *Stimulating caregiving* 134
13.10.8. *Infant neurodevelopment* 134
13.10.9. *Stool sample collection* 134
13.11. VISIT 11-- 9 MONTHS POST-PARTUM (+/- 14 DAYS) 135
13.11.1. *Household food insecurity* 135
13.11.2. *Maternal psychological health* 135
13.11.3. *Blood sampling* 135
13.11.4. *Child anthropometry* 135
13.11.5. *Child vaccination and vitamin A supplementation status* 135
13.11.6. *Child dietary diversity* 135
13.12. VISIT 12: 12 MONTHS POST-PARTUM (+/- 14 DAYS) 135

13.12.1. Household food insecurity 135
13.12.2. Maternal psychological health 135
13.12.3. Blood sampling 136
13.12.4. Child anthropometry 136
13.12.5. Child vaccination and vitamin A supplementation status 136
13.12.6. Child dietary diversity 136
13.12.7. Infant neurodevelopment 136
13.13. UNSCHEDULED SICK VISIT [ANYTIME DURING STUDY FOLLOW-UP] 136

14. LABORATORY PROCEDURES 137

14.1. FULL BLOOD COUNT 137
14.2. MALARIA TESTING 137
14.3. BLOOD FOR CULTURE 138
14.4. BLOOD SAMPLE PREPARATION FOR LONG-TERM STORAGE FOR IRON AND INFLAMMATORY MARKER ASSAYS 138
14.5. VAGINAL AND GUT MICROBIOME ANALYSIS 138

15. NEURO-COGNITIVE ASSESSMENTS USED IN INFANT FOLLOW UP 142

16. ARRANGEMENTS FOR ALLOCATING PARTICIPANTS TO TRIAL GROUPS 144

17. ASSESSMENT OF SAFETY 145

17.1. PRECAUTIONS IN DELIVERING FCM 145
17.2. DEFINITIONS OF ADVERSE EVENTS (AE) 146
17.3. RECORDING OF ADVERSE EVENTS 146
17.3.1. The time period for collecting aEs 146
17.3.2. Method of detecting aEs 147
17.3.3. Collection of AE data 147
17.3.4. Assessment of severity 147
17.3.5. Assessment of causality 147
17.3.6. Study Endpoints and symptoms anaemia 147
17.3.7. HIV related disease 148
17.3.8. Lack of efficacy and Disease Progression 148
17.3.9. Abnormal laboratory values 148
17.3.10. Overdose 148
17.4. REPORTING OF SAES 148
17.4.1. Reporting by the Investigator to the Study Safety Monitor and Sponsor 148
17.4.2. Reporting by the sponsor 149
17.5. DATA MONITORING COMMITTEE 149

18. DATA HANDLING AND RECORD KEEPING 149

18.1. CASE REPORT FORMS (CRFS) 149
18.2. DATA ENTRY AND VALIDATION 150
18.3. DATABASE LOCK 150

19. STATISTICAL CONSIDERATIONS 150

19.1. SAMPLE SIZE 150
19.2. DATA ANALYSIS 150
19.2.1. Assessment of effectiveness 150
19.2.2. Analysis of adverse events 151
19.2.3. Health economics analyses 151
19.3. ANALYSIS POPULATIONS 152
19.4. MISSING DATA 152
19.5. INTERIM ANALYSES AND CRITERIA FOR TERMINATION OF THE TRIAL 152

20. STUDY MANAGEMENT 152

20.1. STUDY MONITORING 152
20.2. DIRECT ACCESS TO SOURCE DATA/DOCUMENTS 153
20.3. QUALITY ASSURANCE 153
20.4. TRAINING OF STAFF 153

20.5. CHANGES TO THE PROTOCOL 153
20.6. FINANCING AND INSURANCE 153
20.7. STUDY DURATION 154
20.8. RECORD-KEEPING AND ARCHIVING 154
20.9. REPORTING AND PUBLICATION OF DATA 154

21. ETHICAL CONSIDERATIONS 154

21.1. ETHICAL REVIEW 154
21.2. ETHICAL CONDUCT OF THE STUDY 155
21.3. INFORMED CONSENT 155
21.4. RISKS TO STUDY PARTICIPANTS 155
21.4.1. Blood sampling 155
21.4.2. Intravenous infusion 156
21.5. BENEFITS FROM PARTICIPATION 156
21.6. SUBJECT DATA PROTECTION 156
21.7. OTHER ETHICAL CONSIDERATIONS 156
21.7.1. Reimbursement of costs 156

22. DISSEMINATION OF RESULTS 157

23. CAPACITY BUILDING 157

25. BUDGET 158

26. REFERENCES 160

27. APPENDIX A–- SAFETY AND EFFICACY OF FCM FROM RANDOMISED CONTROLLED TRIALS 165

6. ABBREVIATIONS

ABRs	Auditory Brainstem Responses
ANC	Antenatal Care
CoM	College of Medicine
CRF	Case Report Form
DASS-21	Depression Anxiety Stress Scale-21
dL	Decilitre
EEG	Electroencephalography
EPDS	Edinburg Postpartum Depression Scale
FCM	Ferric Carboxymaltose
Hb	Haemoglobin
HC	Health Centre
HIV	Human Immunodeficiency Virus
ICTRP	International Clinical Trials Registry Platform
IPTp	Intermittent Preventive Treatment in pregnancy
IV	Intravenous
LMICs	Low and Medium-Income Countries
MCATS	Melbourne Clinical and Translational Science Platform
MRI	Magnetic Resonance Imaging
PCR	Polymerase Chain Reaction
QECH	Queen Elizabeth Central Hospital
RCT	Randomised Controlled Trial
RDT	Rapid Diagnostic Test
SC	Sickle Cell
SHC	Sickle Haemoglobin Cell
SRQ	Self-Reporting Questionnaire
SP	Sulfadoxine-Pyrimethamine
SpHb	Pulse Oximetry Hemoglobin
TEAs	Treatment-Emergent Adverse Events
UK	United Kingdom
US	United States
WHO	World Health Organisation
ZCH	Zomba Central Hospital

7. INTRODUCTION

7.1. BACKGROUND

7.1.1 Anaemia during pregnancy remains a critical global health problem

Almost 40% of pregnant women worldwide are anaemic, including 46% of pregnant women in Africa and 49% in Asia, compared with 17% in North America [1]. Anaemia in pregnancy is associated with critical risks for both mother (e.g. life-threatening complications of post-partum haemorrhage) and child (especially prematurity and low birth weight [2], which are associated with increased risk of mortality, and reduced iron stores in infancy with increased risk of subsequent anaemia and impaired development) [3]. Recent data confirm that even in developed countries such as the UK, anaemia in pregnancy is associated with an adjusted 3.6 odds ratio for increased maternal mortality [4], and in the US is associated with impaired long-term child cognitive development [5]. Control of anaemia in women is, therefore, a key 2025 global nutrition target [6].

7.1.2 Low birth weight and pre-term birth have critical implications for mother and baby

Worldwide, 15%-20% of births (>20 million annually) (including 13% in sub-Saharan Africa) are low birth weight, while each year, over 1 million children die from complications of pre-term birth. Although the underlying determinants for low birth weight and prematurity are multifactorial, prevention and treatment of antenatal anaemia with iron appears a crucial intervention and considered an essential component of the 2025 WHO nutrition target of a 30% reduction in low birth weight.

7.1.3 Iron can benefit antenatal anaemia

Benefits and safety of iron supplementation during pregnancy in the sub-Saharan African context were most clearly exemplified in a recent placebo-controlled double-blind randomized single centre field trial in Kenyan pregnant women [7]. In this trial, oral iron supplementation increased birth weight by 150g and reduced the risk of low birth weight by 58%, lengthened gestation duration by 3.4 days and reduced the risk of premature birth by 7% and reduced the risk of maternal anaemia from 50.4% to 22%. Reassuringly, in this highly malaria-endemic setting, there was no evidence of an increase in clinical malaria, placental malaria or parasitaemia among women randomized to iron. This trial is one of the most recent examples of an extensive literature of trials evaluating oral iron in pregnancy. Systematic reviews of these studies confirm likely benefits from iron on maternal outcomes including anaemia (70% reduction), and trends towards favourable infant outcomes including increased birth weight and extended gestation duration [8]. However, the success of the Kenyan trial was likely predicated on the extremely high adherence demanded of the participants (100%), achieved by daily visits by fieldworkers. This emphasizes the importance of delivery of a full course of iron supplementation on birth outcomes.

7.1.4 Uptake and adherence to oral iron therapy during pregnancy is inadequate in the field

Global recommendations for management of anaemia in pregnancy in LMICs currently advise that women be treated with high dose daily oral iron (120 mg of elemental iron) supplementation for 3 months [9, 10]. However, such high doses of iron are often poorly tolerated due to significant gastrointestinal adverse effects [11] limiting adherence to this vital intervention. Moreover, delivery of iron during pregnancy requires ongoing contacts between the mother and primary health system. For example, in Malawi, fewer than 25% of pregnant women receive a full course of iron (Clophat Baleti, Personal Communication). Across Africa, only very few women receive the recommended course of antenatal iron and may present for their initial visit far into the second trimester. This limits opportunity to treat antenatal anaemia, exposing women and their babies to its consequences. Furthermore, even when delivered, oral iron frequently fails to correct anaemia in routine practice. For example, in our recent study among pregnant women in The Gambia, we provided iron to all women with Hb<10g/dL at week 20 of gestation; by week 30, 61% still had Hb<10g/dL. [12]

7.1.5 Ferric carboxymaltose as a new intravenous iron preparation recommended widely

Over the past decade, there have been dramatic improvements in the safety and convenience of parenteral (intravenous) iron therapies. Older forms of intravenous iron were associated with serious allergic reactions (iron dextran), required prolonged administration periods (iron polymaltose), or could only be administered in small doses (200-300mg) requiring frequent infusions (iron sucrose).

However, new parenteral intravenous agents have completely changed the landscape of iron therapy. The most established therapy is Ferric carboxymaltose (FCM), which overcomes many limitations of previous parenteral iron treatments. Ferric carboxymaltose is a new and differently engineered iron formulation. It comprises a colloidal complex of a polynuclear iron (III) oxyhydroxide core with carboxymaltose ligands. [13] Following administration, the complex is degraded into simple endogenous molecules (glucose, maltose, maltotriose, maltotetraose and iron), and iron is rapidly taken up by iron transport (transferrin) and storage (ferritin) proteins. Due to this property, FCM can be administered in a short period of time (15 min) and at large doses (up to 1000 mg in EU) [14]. Unlike iron dextran, the risk of anaphylaxis has been largely overcome, although hypersensitivity reactions may still occur. Unlike iron polymaltose, the infusion can be administered rapidly. Unlike iron sucrose, the maximum dose of iron is high enough that a single visit is sufficient for a high (usually total) dose of iron to be delivered. As currently licensed, a dose of up to 1000mg of iron (or 15-20mg/kg body weight), diluted in 250mL saline, may be administered over a 15-minute infusion. In most cases, this enables a total dose iron replacement to be achieved in a single visit. FCM has now revolutionised the treatment of iron deficiency anaemia in high-income country settings and is widely used in ambulant/ outpatient settings, emergency departments, in non-specialist clinical wards, preoperative clinics and even in remote settings. FCM has been available in Europe since its approval in 2007 and in the USA since 2009; it is currently marketed in over 50 countries. [14]

7.1.6 Safety and efficacy evidence of Ferric carboxymaltose in various patient populations

Rognoni et al 2015 undertook a systematic review and network meta-analysis of FCM vs both oral iron and other parenteral formulations. [15] The authors identified 21 RCTs comparing iron treatments in anaemic (or non-anaemic) patients requiring therapies for ID published between 2003-2014, although not all of these included FCM. The trials reporting safety and efficacy of FCM are summarised in Appendix A.

Regarding safety, the review authors found that overall, FCM was well tolerated and associated with a minimal risk of aEs [15]. In trials in which Adverse Events (aEs) occurred in a larger proportion of FCM-treated patients than those receiving oral iron or placebo, the difference seldom reached the statistical significance level. Several studies found that patients who were given FCM experienced fewer drug-related gastrointestinal disorders (e.g. constipation, diarrhoea) than those treated with oral iron. However, patients treated with ferric carboxymaltose iron were more likely to develop rash, dermatitis and pruritus that generally resolved within a few minutes of the infusion. Other frequent aEs associated with ferric carboxymaltose administration were fatigue, headache and dizziness. No true cases of anaphylaxis and no deaths occurred in patients receiving FCM.

Regarding efficacy, the authors undertook a network meta-analysis to compare the efficacy of FCM as compared with oral iron and other parenteral formulations. The authors found that in terms of improving haemoglobin concentrations, FCM was superior to placebo (delta 2.1; 95 % CI 1.2–3.0), oral iron (delta 0.8; 95 % CI 0.6–0.9), intravenous ferric gluconate (delta 0.6; 95 % CI 0.2–0.9), and iron sucrose (but not reaching statistical significance). The authors found that for improvements in ferritin, FCM was superior to oral iron (delta 172.76; 95 % CI 66.7–234.4) and similar to other parenteral formulations (iron sucrose and gluconate) [15].

A previous systematic review and meta-analysis published in September 2011 evaluated published and unpublished studies of efficacy and safety of ferric carboxymaltose [16]. The review evaluated clinical trial reports and published studies comparing FCM with either other active comparators or with placebo. The authors identified fourteen studies, in which 2,348 patients had been assigned to FCM, compared with, in the control arms, 762 to placebo, 832 to oral iron and 384 to intravenous iron sucrose. Compared with oral iron, ferric carboxymaltose produced superior improvements in haemoglobin, ferritin and transferrin saturation, and was associated with a greater chance of resolution of anaemia or clinically significant improvement in haemoglobin. Maximum responses were usually achieved by 4-6 weeks. Adverse events were also evaluated.

The table below, from this manuscript, shows the risk of withdrawals and adverse events in patients receiving ferric carboxymaltose compared with those randomized to receive either control or another iron intervention. As compared with either oral iron or placebo, participants receiving FCM were slightly less likely to withdraw from the study, while there was no difference in the number of participants experiencing at least one adverse event, death, or serious adverse events between subjects receiving FCM and those receiving other interventions. However, there was a slight increase in the risk of hypotension in participants receiving FCM when compared specifically with oral iron (but not when compared with receipt of other intravenous interventions).

Table 2: Risk of Withdrawals and Adverse Events in patients receiving IV Ferric Carboxymaltose

Outcome	Comparator	Number of Trials	Number of Patients	Percent with Ferric carboxymaltose	Percent with Control	RB or RR 95% CI	NNTp 95% CI
**Withdrawals**							
All cause	All	10	3835	6.1	7.5	0.8 (0.6 to 0.9)	93 (37 to 180)
	Oral iron	6	1898	8.1	9.3	0.8 (0.6 to 1.03)	not calculated
Adverse event	All	8	3319	1.0	1.6	0.6 (0.3 to 1.02)	not calculated
	Oral iron	6	1898	1.5	1.9	0.7 (0.3 to 1.4)	not calculated
Lack of efficacy	All	7	2967	0.6	0.8	0.8 (0.4 to 1.7)	not calculated
	Oral iron	5	1546	1.1	1.2	0.8 (0.4 to 1.9)	not calculated
**Adverse events**							**NNH 95% CI**
At least 1 AE	All	8	2951	41	38	1.1 (1.0 to 1.2)	not calculated
	Oral iron	5	1539	48	53	1.0 (0.9 to 1.1)	not calculated
Death	All	10	3762	0.53	0.3	1.3 (0.5 to 3.4)	not calculated
	Oral iron	6	1891	0.38	0.0	1.7 (0.4 to 6.6)	not calculated
Serious AE	All	8	3303	2.5	2.3	1.0 (0.6 to 1.5)	not calculated
	Oral iron	6	1891	3.1	2.3	1.3 (0.7 to 2.2)	not calculated
Hypotension	All	6	2694	1.5	1.0	1.5 (0.8 to 2.7)	not calculated
	Oral iron	4	1339	1.3	0.0	4.7 (1.1 to 21)	79 (44 to 390)

Note: NNT=Number Needed to Treat; NNH=Number Needed to Harm

The table below, adapted from this meta-analysis, compares the incidence of various organ-specific side effects associated with the administration of FCM when compared with 1) oral iron as the control, and 2) IV saline as the control.

Table 3: Organ-specific side effects of ferric carboxymaltose compared with oral iron or IV saline

Outcome	Number of Trials	Number of Patients	Percent with Ferric carboxymaltose	Percent with Control	RB or RR 95% CI	NNTp/H 95% CI
**Comparison with oral iron**						
**Body system and preferred term**						
GI disorder	5	1539	13	32	0.44 (0.36 to 0.54)	5.4 (4.4 to 7.1)
General, administrative site	5	1539	11	4	2.8 (1.9 to 4.2)	**15 (11 to 24)**
Infection, infestation	5	1539	14	12	1.2 (0.9 to 1.6)	not calculated
Metabolism, nutrition, investigation	4	1195	11	5	2.2 (1.4 to 3.4)	**17 (11 to 33)**
Nervous system	5	1539	10	9	1.3 (0.9 to 1.7)	not calculated
**Specific adverse events**						
Constipation	4	1339	3	13	0.3 (0.2 to 0.4)	9.8 (7.6 to 14)
Diarrhoea	3	906	2	5	0.5 (0.2 to 0.9)	33 (18 to 230)
Nausea/vomiting	3	906	3	10	0.4 (0.2 to 0.6)	14 (9.5 to 27)
Headache	5	1539	7	7	1.2 (0.8 to 1.7)	not calculated
**Comparison with IV saline**						
**Body system and preferred term**						
GI disorder	2	1577	8	5	1.6 (1.1 to 2.4)	**34 (19 to 210)**
General, administrative site	2	1577	6	2	2.5 (1.5 to 4.3)	**25 (17 to 49)**
Infection, infestation	2	1577	9	6	1.1 (0.8 to 1.6)	not calculated
Nervous system	2	1577	8	6	1.2 (0.9 to 1.8)	not calculated
Respiratory system	2	1577	2	2	0.8 (0.4 to 1.5)	not calculated

Note NNTp in normal text, NNH when bold

As shown in the table above, compared with oral iron, FCM was less likely to cause constipation, diarrhoea, nausea/ vomiting, and gastrointestinal disorders in general, but was more likely to cause adverse events at the administration site. When FCM was compared to intravenous saline, FCM was associated with an increased risk of 'GI disorder' and local site reactions, but not with adverse effects in other organ systems.

Numerous randomised controlled trials have compared FCM with either oral iron or other forms of parenteral iron for treatment of IDA in a variety of clinical situations, including pregnancy,[17] post-partum,[18] and in a variety of clinical conditions including inflammatory bowel disease,[19-25] chronic renal failure,[26, 27] and heart failure.[28] It has also been studied in the preoperative context for optimising pre-operative haemoglobin concentrations as a component of patient blood management.[29-34] Here, we will summarise recent reviews of FCM that address key safety and efficacy endpoints.

7.1.7 Safety of Ferric carboxymaltose

Several studies, including one randomised controlled trial, have evaluated the efficacy and safety of FCM in pregnancy. These studies are summarised here:

Breymann et al: [17] FER-ASAP was the pivotal, Phase III, open-label randomised controlled trial comparing FCM with oral iron (ferrous sulphate) for treatment of iron deficiency anaemia in pregnancy. This multicentre study was set across 8 high and middle-income countries and randomised 252 women in their second or third trimester of pregnancy to FCM (1000-1500 mg iron) or FS (200 mg iron/day for 12 weeks). The trial was designed to identify a more rapid improvement in haemoglobin concentration by 3 weeks' post-infusion compared with baseline. The study showed an advantage of Hb with FCM compared with FS at 6 and 3 weeks (although the difference was not significant at 3 weeks). According to the SF-36 health survey, FCM treatment led to significant, clinically relevant improvements over FS in vitality and social functioning prior to delivery.

Safety: The incidence of Treatment-Emergent Adverse Events (TEAEs) was similar between the treatment arms: in the FCM group, 60 women (49%) experienced 165 TEAEs; in the FS group, 50 women (40%) experienced 105 TEAEs. The majority of events were mild in intensity (see table 5 below).

Table 4: Incidence of Treatment-Emergent Adverse Events (TEAEs)

Treatment-related TEAE severity, number of patients (%)	Ferric carboxymaltose (n=123)	Ferrous sulfate (n=124)
Total	60 (49)	50 (40)
Mild	43 (72)	28 (56)
Moderate	17 (28)	20 (40)
Severe	0 (0)	2 (4)

TEAE=treatment-emergent adverse event.

Overall, the most common TEAEs were nausea (6%), headache (5%) and dyspepsia (4%), and the most common TEAEs according to system organ class were "pregnancy, puerperium and perinatal conditions" in the FCM group [32 events in 26 women (21%)] and "gastrointestinal disorders" in the FS group [42 events in 25 women (20%)]. The most common treatment-related TEAEs were headache with FCM [experienced by 4 women (3%)] and nausea with FS [in 6 women (5%)], and markedly higher rates of gastrointestinal disorders were reported with FS treatment (in 16 women) compared with FCM treatment (3 women).

Table 5: TEAEs in FCM vs FS treatment arms

Treatment-related TEAE,[a] number of patients (%)	Ferric carboxymaltose (n=123)	Ferrous sulfate (n=124)
Total	14 (11)	19 (15)
Nervous system disorders	7 (6)	1 (1)
Headache	4 (3)	1 (1)
Dizziness	3 (2)	0 (0)
Dysgeusia	2 (2)	0 (0)
General disorders and administration-site conditions	4 (3)	0 (0)
Vascular disorders	2 (2)	0 (0)
Gastrointestinal disorders	3 (2)	16 (13)
Nausea	2 (2)	6 (5)
Vomiting	0 (0)	2 (2)
Constipation	0 (0)	3 (2)
Diarrhea	0 (0)	4 (3)
Abdominal pain upper	0 (0)	5 (4)
Dyspepsia	0 (0)	3 (2)

[a]According to physician's assessment; a single patient could appear in multiple classes. Percentages calculated according to treatment group.
MedDRA=Medical Dictionary for Regulatory Activities (version 16.1). TEAE=treatment-emergent adverse event.

Serious TEAEs occurred in 23 women treated with FCM (26 events) and in 10 women treated with FS (11 events); all were single events, except for "failed trial of labour", "foetal distress syndrome", "premature delivery", "premature rupture of membranes" and "threatened labour", each of which occurred in two women treated with FCM, and "premature labour", which occurred in three women treated with FS (pre-eclampsia was also reported to have occurred twice in one woman treated with FS). One serious TEAE, "bronchospasm", which was treatment-related, led to discontinuation of FCM; this event was of moderate intensity and resolved on the same day after withdrawal of study treatment. Seven women discontinued treatment with FS because of gastrointestinal TEAEs (n=5), syncope (n=1) and rash (n=1). No hypophosphatemia TEAEs were reported during this study. Eleven women (FCM, n=10; ferrous sulphate, n=1) recorded phosphate levels below the lower normal range threshold [0.6 mmol/L (2 mg/dL)]; these decreases were observed at week 3 and recovered in all the women to within the normal range by the end of the study.

All other trials have been single arm prospective trials or retrospective comparative studies. *Froessler et al* undertook a prospective observational study in 65 anaemic pregnant women in South Australia, Australia, receiving ferric carboxymaltose (up to 15 mg/kg) between 24 and 40 weeks' gestation (median 35 weeks). [35] The authors observed increases in haemoglobin concentrations at 3, 6 and up to 8 weeks' post-infusion (see table 6 below), although in women with the most severe anaemia at baseline, improvements in haemoglobin were not sustained by 2 months postpartum.

Table 6: Increase in haemoglobin concentration at 3, 6 and 8 weeks' post-infusion

	Gestational age at entry	Pre-infusion	3 weeks post infusion	6 weeks post infusion	8 weeks post infusion (post-partum)
Mild ≥95 g/L	34 (4)	102.1 (1.0) n = 31	108.3 (3.9)* n = 28	120.6 (2.9)* n = 18	113.1 (4.2)* n = 11
Moderate 90-94 g/L	36 (2)	92.6 (0.4) n = 14	105.8 (3.0)* n = 13	108.4 (3.8)* n = 5	92.7 (12.4) n = 3
Severe <90 g/L	34 (3)	83.7 (0.9) 20	100.2 (3.3)* n = 17	110.0 (8.1)* n = 5	93.3 (8.1) n = 4

Data are presented as means (SEM). *p < 0.01 compared to pre-infusion haemoglobin levels.

Safety: No serious adverse effects were recorded in any of the 65 women receiving an infusion. Minor side effects occurred in 13 (20%) patients. One patient required medication with Metoclopramide for nausea and vomiting. All other adverse events were self-limiting. Foetal heart rate monitoring did not indicate a drug-related adverse effect on the fetal heart pattern. Red blood cell transfusions were required by 3 women (4.6%) in the study cohort, all of whom had a significant peripartum haemorrhage. Adverse effects from the cohort are summarised in the table 7:

Table 7: Number of women experiencing a drug related adverse event following infusion with ferric carboxymaltose (total number of women infused n= 65)

Adverse event	n (%)
Any adverse event	13 (20)
Local (injection site irritation)	
Slight burning sensation	5 (8)
Systemic	
Hypotension	1 (1.5)
Headache	4 (6)
Nausea/Vomiting	1 (1.5)
Pruritus	2 (3)

Zeba et al undertook a prospective cohort study in 260 pregnant women between 28 and 36 weeks' gestation with haemoglobin <10g/dL in a private obstetric clinic in Faridpur, Bangladesh [36]. All women in this study group were treated with intravenous ferric carboxymaltose as a single dose (500-1000mg) in an infusion time of 15-20minutes. *Safety:* No serious adverse events occurred, with 13% experienced minor adverse effects such as injection site irritation, headache, nausea or vomiting etc. *Efficacy:* Compared with the Hb (mean 8.9g/dl) level before infusion, the Hb (mean 10.53g/dl) level after infusion had significantly (p<0.001) increased.

Mishra et al. undertook a single arm prospective cohort trial of FCM in Ahmedabad, India in 108 participants [37]. Women received up to 1500mg FCM in total. *Safety*: Three women experienced local reactions e.g. itching and irritation at the infusion site, while another 5 women reported systemic reactions of giddiness, headache and nausea. *Efficacy*: FCM produced an average increase of 2.1g/dL haemoglobin in 3 weeks.

Christoph P et al. undertook a retrospective analysis of 206 pregnant women who had received either FCM or iron sucrose during pregnancy in order to assess maternal safety and

tolerability [38]. The authors observed that Mild adverse events occurred in 7.8% for ferric carboxymaltose and in 10.7% for iron sucrose. The authors also concluded that “no sign for a negative effect on the fetus of iron infusion could be detected”.

7.1.8 Use of Ferric carboxymaltose in pregnancy

Based on these studies, licensing by international regulators including the FDA, EMA and TGA, and clinical experience, FCM has rapidly become an established treatment for anaemia in pregnancy in developed countries. For example, British Committee for Standards in Haematology 2011 guidelines recommend that parenteral iron be considered for women from the second trimester onwards with iron deficiency anaemia who fail or are intolerant of iron. [39] Many UK health services (NHS Trusts) recommend Ferric Carboxymaltose as second-line therapy for iron deficiency anaemia in pregnancy. For example, the Mid Essex NHS Trust recommends FCM for women with persistent anaemia despite 4 weeks’ oral iron, or with haemoglobin <80g/L on any occasion, or whom are intolerant of or non-compliant with oral iron; the guideline also recommends FCM as first-line therapy in all women who are anaemic beyond 34 weeks’ pregnancy. [40] In many cases, use is limited not by concerns of efficacy or safety, but by cost and health economic implications.

A 2012 review suggested that as FCM and other novel parenteral iron agents represent a ‘milestone’ in intravenous iron therapy, they should be considered as increasingly first-line for treatment of anaemia in pregnancy. [41] Here, the authors suggested that women with Hb<10g/dL should probably be given IV iron if they have a ferritin <30mg/L, and among women with higher ferritin levels, this represents an area of research in establishing whether IV or oral iron should be given.

However, since the publication of the FER-ASAP trial, use of IV FCM in pregnancy is becoming increasingly considered as the first line. For example, a recent influential ‘How I Treat’ review article in the leading haematology journal ‘Blood’ formalized the new position of parenteral iron as first-line therapy for iron deficiency anaemia in pregnancy. [42] Here, the authors suggest that **all** cases of anaemia in the second or third trimester receive intravenous iron.

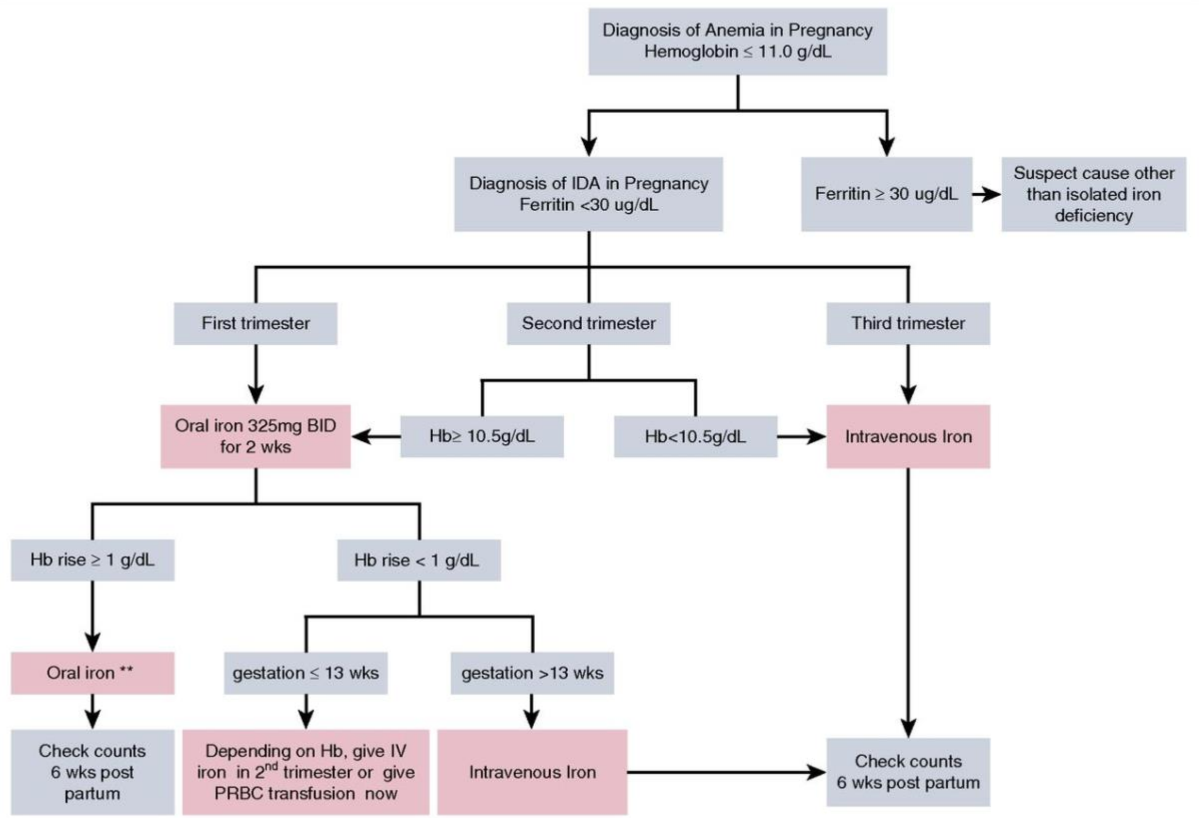

Figure 1: Decision tree on parenteral iron as first-line therapy for iron deficiency anaemia in pregnancy *[From: Achebe MM et al. How I treat anaemia in pregnancy: iron, cobalamin, and folate. Blood 2017; 129:940-9].*

Thus, in high-income countries, FCM is now mainstream, routine therapy for iron deficiency anaemia in pregnancy. Since FCM is already licensed for the treatment of anaemia in pregnancy beyond the first trimester, using European MHRA guidelines, the REVAMP trial would be categorised as Type A = No higher than the risk of standard medical care. Importantly, although FCM has entered routine care, there remains little data on the effects of antenatal FCM (or IV iron) on post-partum health or long term infant health and growth and development.

7.1.9 Use of Intravenous Iron/ FCM in sub-Saharan Africa

There is clinical and research experience in the use of FCM in sub-Saharan Africa. FCM is now available in South Africa. A large randomised clinical trial comparing FCM to oral iron for treatment of post-partum anaemia is in progress in Tanzania (NCT02541708). Likewise, there is clinical experience using iron sucrose to treat anaemia in Malawi. Iron sucrose is included as a therapy for management of anaemia in 2015 Malawi Standard Treatment Guidelines (p26).

7.1.10 Measurement of haemoglobin

7.1.10.1 Background

Assessment of haemoglobin concentration is required to diagnose anaemia and stratify care for women in pregnancy. However, in many low-income settings, infrastructure for haemoglobin assessment remains highly limited. Haemoglobin is commonly measured using

capillary blood on an instrument such as a HemoCue, or using a venous sample on an automated analyser. Access to both technologies can be limited outside major settings and research sectors. In recent years, Masimo Corporation (USA) have developed a technique for non-invasive measurement of haemoglobin status using a pulse oximetry approach (SpHb). This technique is now being introduced to clinical management in some clinical settings in high income countries. Data from the Masimo study collected at five different centers on healthy and sick children using Rad-67 spot check demonstrated good accuracy. With 319 subjects and 660 samples, the limits of agreement between SpHb versus Laboratory Haematology Analyser was -1.82 to 2.07. [43] The findings are not different to limits of agreement between Invasive Point-of-care Device versus Laboratory Haematology Analyser (-2.5 to 1.98) using 283 subjects and 283 samples. This offers great promise in low income settings where access to laboratories is limited. It would also minimise the use of needles, reducing discomfort for patients and ameliorating health care worker infection risk. However, this technology has not yet been validated or prospectively investigated in pregnancy, or in a sub-Saharan African population. Specific measurement in pregnancy is critical prior to implementation as factors such as blood volume, oedema and capillarisation can change readings, meaning existing software settings and diagnostic accuracy definitions may require revision.

7.1.10.2 Procedure (for more information, refer to the attached procedure manual)

SpHb measurements will be performed at pre-defined visits: recruitment (Visit 1, Day 0), 4-week follow-up (Visit 2, Day 28 ± 2 days) and at the pre-delivery follow-up (Visit 4, 36 weeks' gestation ± 2 days), 10 minutes before/after the blood samples are taken. Subjects that have any injury, deformity, nail polish or any other abnormality interfering with sensor application will not be included in this sub-study. To ensure proper use of sensor and device, the person obtaining the SpHb measurement will use the SpHb measurement procedures for using the sensor and device. This will help ensure accurate measurement of SpHb. The SpHb measurement will not be used to make any medical or study decisions. Kindly find attached to this protocol, the equipment brochure, Food and Drug Administration (FDA) clearance and the equipment manual for your reference.

7.2. THE RATIONALE FOR THE RESEARCH PROJECT

A treatment, which could rapidly restore maternal iron stores to treat maternal anaemia, could optimise maternal and infant outcomes and be of immense value in LMICs.

7.2.1 Modern formulations of intravenous iron are safe and enable total dose iron infusion Ferric carboxymaltose (Ferinject/Injectafer) is approved in Europe, Australia and the United States for treatment of iron deficiency anaemia in men and women of all ages including pregnant women. Following administration, iron is released from the carboxydrate shell and rapidly taken up by iron transport (transferrin) and storage (ferritin) proteins. This drug is not associated with risks of anaphylaxis; low-grade effects such as joint pains, flushing and biochemical hypophosphatemia are commonly observed but well-tolerated [46]. A ferric carboxymaltose 1000mg elemental iron dose can be administered in 250mL over 15 minutes, without test doses or safety monitoring.

Ferric carboxymaltose is approved for use in pregnancy in the second and third trimesters, and its use is increasingly recommended in complex patients. For example, British guidelines for management of antenatal iron deficiency indicate that parenteral iron should be considered in the second trimester onwards in women who are iron deficient who are intolerant to or fail to respond to iron [39]. Ferric carboxymaltose is increasingly recommended as first-line therapy for all pregnant women with moderate or severe anaemia [42].

7.2.2 Ferric carboxymaltose is superior to oral iron for anaemia in pregnancy in developed settings

Efficacy and safety for treatment of anaemia in pregnancy of IV ferric carboxymaltose compared with oral iron has been demonstrated in a recent open-label randomized international multicentre Phase III trial [47] which recruited 252 pregnant women with iron deficiency anaemia across 7 developed countries and randomized them to IV FCM (dose based on degree of anaemia and weight) or very high dose oral iron (ferrous sulphate, 100mg elemental iron twice daily). Pregnant women treated with FCM were twice as likely to correct their anaemia (odds ratio: 2.06, P=0.031), and the median time to achieving this correction was shorter (3.4 vs. 4.3 weeks). FCM also improved iron stores and maternal vitality and social functioning prior to delivery compared with oral. In this trial (undertaken in high-income countries, and with high dose oral iron as a comparator), effects on birth outcomes were not observed. Oral iron caused more gastrointestinal effects compared to intravenous. Low PO4 was seen in 10/126 patients receiving IV iron compared to no reports in patients receiving oral iron, but clinically symptomatic hypophosphatemia was not seen. Extended post-partum follow-up of the mothers and infants to assess for longer-term effects of the interventions were not reported in this study.

In LMICs, where adherence and efficacy of oral iron are potentially inadequate, intravenous ferric carboxymaltose presents a novel, innovative opportunity to rapidly correct moderate and severe antenatal anaemia in a single dose, improving maternal and neonatal outcomes.

8. TRIAL OBJECTIVES AND END POINTS

8.1. OBJECTIVES

8.1.1. *Broad Objective*

To determine whether FCM up to 1000mg given once during the second trimester is superior to oral iron as administered by routine health services during pregnancy in the reduction of maternal anaemia at 36 weeks' gestation ~~term~~.

8.1.2. *Specific Objectives*

7. To determine the effectiveness of FCM on maternal anaemia at ~~delivery~~ 36 weeks' gestation, when compared with routinely delivered oral iron.

8. To determine the effectiveness of FCM compared to routinely delivered oral iron on critical neonatal outcomes including birth weight (low birth weight), gestation duration (prematurity), small for gestational age and other adverse birth outcomes.
9. To determine the effectiveness of FCM compared to routinely delivered oral iron on maternal outcomes including haemoglobin concentration and iron deficiency (measured through iron biomarkers), maternal quality of life and maternal cognitive function.
10. To determine the safety of IV iron compared to oral iron on the prevalence of all-cause sick visits, serious maternal adverse events, clinical malaria, bacteraemia, placental malaria, malaria parasitaemia, hypophosphatemia and pathogenic vaginal bacterial flora during pregnancy.
11. To determine immune and molecular aetiologies that may be related to anaemia, iron deficiency and infection in pregnancy.
12. To determine the cost-effectiveness of IV iron compared to oral iron measured through absolute and incremental health provider and patient costs.
13. To determine the effectiveness of FCM compared to oral iron in improving longer-term infant haematologic, iron status, neurodevelopmental, growth and immunological outcomes.
14. To determine the long-term effects of FCM compared to oral iron on maternal wellbeing after giving birth
15. To assess the association between maternal haemoglobin and iron status after giving birth and mother-child interaction
16. To assess the diagnostic test accuracy of non-invasive haemoglobin measurement (SpHb) compared with Hb measured by venous blood, in pregnant women.
17. To determine the impact of FCM on maternal bone health and on infant phosphate regulation and infant bone health (sub-set of 200 mothers and their infants).

8.2. ENDPOINTS

In order to make the case for implementation of this more complicated intervention, it is essential to demonstrate the superiority of IV iron for the health of both the mother and new-born. Likewise, it is crucial the intervention be shown to be safe, with specific regard to the risk of infection.

8.2.1. *Primary Outcome*

Prevalence of maternal anaemia (Hb <11.0g/dl) at 36 weeks' gestation.

8.2.2. *Secondary Outcomes (Benefit in mother)*

36. Maternal quality of life, using the Piper Fatigue Scale (PFS) at 36 weeks' gestation and 1 month post-delivery.
37. Maternal cognitive function at 4 weeks' post randomisation and 1 month post-delivery.
38. Maternal Hb at 4 weeks' post randomisation, 36 weeks' gestation and prior to delivery.

39. Maternal iron deficiency (defined by i) ferritin<15mg/L, and ii) sTfR/Ferritin index – assay dependent cut-off, iii CRP) at 4 weeks' post randomisation and 36 weeks' gestation.
40. Maternal anaemia (Hb<11g/dL) and haemoglobin concentration at 1, 3, 6, 9 and 12 months post-delivery.
41. Maternal iron deficiency, iron indices and CRP at 1, 3, 6, and 12 months post-delivery.
42. The incidence of severe anaemia requiring a blood transfusion
43. The incidence of severe medical events: haemorrhage, ~~sepsis, shock,~~ need for transfusion, ICU or prolonged admission, or mortality, both individually and as a composite outcome.
44. Incidence of maternal psychological health problems at 1, 3, 6 and 12 months post-delivery measured by Depression, Anxiety and Stress Scale (DASS-21) and EPDS.
45. Determinants of maternal psychological health problems after giving birth
46. Mother-child interaction at 1, 3 and 6 months postpartum measured by Parent/Caregiver Involvement Scale

8.2.3. *Secondary Outcomes (Benefit in the neonate and infant)*

47. Birth weight and birth length – measured in grams and centimetres within 24 hours of birth
48. Placental weight and its ratio to birth weight
49. Gestational-age adjusted birth weight
50. Small for gestation age
51. Low birth weight – defined as birth weight less than 2500 g (up to and including 2499g)
52. Abortion – defined as a pregnancy loss before 28 completed weeks of gestation
53. Stillbirth – defined as the birth of a baby showing no signs of life after 28 weeks of gestation (>28 weeks)
54. Gestation duration (in weeks)
55. Premature births – defined as the neonate born prior to 37 completed weeks of gestation (including 36weeks and 6 days).
56. Cord venous blood Hb, ferritin, and sTfR-ferritin index levels
57. Infant haemoglobin concentration at 1, 3, 6, 9 and 12 months of age.
58. Infant iron status at 1, 3, 6, 9 and 12 months of age.
59. Infant growth (weight for age, length for age, weight for length, head circumference) at 1, 3, 6, 9 and 12 months of age.
60. Infant immunologic function at 3, 6 and 12 months of age
61. Infant neurodevelopment at 6 and 12 months of age (measured by Bayley Scales of Infant Development and Event Related Potentials (ERPs)).
62. Infant neurodevelopment within 48 hours of delivery, at 1 month of age, and 6 months of age (measured by Auditory Brainstem Responses).

63. Infant neurodevelopment at 12 months of age (brain structure parameters measured by low field portable MRI technology) in a sub-set of 60 infants.

8.2.4. *Secondary Outcomes (Safety in the mother)*

64. Adverse events related to administration of the intervention
65. The incidence of all-cause sick visits to the clinic (antenatal and postpartum reported separately)
66. The incidence of diarrhoea sick visits to the clinic (antenatal and postpartum reported separately)
67. The incidence of clinical malaria-specific sick visits to the clinic (antenatal and postpartum reported separately)
68. The incidence of placental malaria at delivery
69. The incidence of peripheral parasitaemia by 36 weeks of gestation
70. The incidence of cord blood parasitaemia at delivery
71. Prevalence of malaria parasitaemia at 4 weeks' post randomisation and at 36 weeks' gestation
72. Prevalence of hypophosphatemia (clinical and biochemical) at baseline, 4 weeks' post randomisation, ~~and~~ 36 weeks' gestation and at 3 months postpartum.
73. Prevalence of maternal inflammation, defined by elevated C-reactive protein and alpha-1 glycoprotein levels, at 4 weeks' post randomisation and at 36 weeks' gestation.
74. Prevalence of pathogenic vaginal bacterial flora and gut microbiome at 4 weeks' post randomisation and at 36 weeks' gestation.
75. Prevalence of bone health dysregulation (determined by bone health markers) at baseline, 4 weeks' post randomisation, 36 weeks' gestation, 3 months post-partum and at 6 months post-partum.

8.2.5. *Secondary Outcomes (Safety in the infant)*

46. The incidence of all-cause sick visits to the clinic
47. The incidence of diarrhoea sick visits to the clinic
48. The incidence of clinical malaria-specific sick visits to the clinic
49. Incidence of peripheral parasitaemia by 12 months of age
50. Prevalence of malaria parasitaemia at 1, 3, 6, 9 and 12 months of age
51. Prevalence of pathogenic gut and upper respiratory microbiota at 1, 3, 6, 9 and 12 months of age
52. Prevalence of inflammation (CRP) at 1, 3, 6, 9 and 12 months of age
53. Prevalence of hypophosphatemia at 3, 6, 9 and 12 months of age
54. Prevalence of bone health dysregulation (determined by bone health markers) at 3, 6, 9 and 12 months of age
55. Prevalence of radiological Rickets at 6 and 12 months of age

8.2.6. *Secondary Outcomes (Cost and cost-effectiveness)*

45. Health systems costs of providing the treatment and follow-up for the intervention and comparator
46. Direct and indirect patient costs of receiving the intervention and the comparator
47. Indirect patient costs of disease (productivity consequences)
48. Incremental cost-effectiveness of FCM compared to oral iron given to pregnant women in Malawi

8.2.7. *Secondary Outcomes (diagnostic test accuracy of SpHb)*

49. Agreement between SpHb and venous Hb (measured by automated analyser) in pregnant women.
50. Sensitivity and specificity of SpHb for a diagnosis of i) anaemia, ii) moderate anaemia, and iii) severe anaemia, subcategorised by second and third trimester.

9. STUDY DESIGN

This will be a Phase III open-label 2-arm parallel group randomized controlled trial recruiting pregnant women in their second trimester and following them up until 1 month postpartum. Participants will be recruited from antenatal clinics in urban and semi-urban areas and randomized to receive IV iron once or oral iron for 90 days or the duration of pregnancy, whichever is shorter. An open-label (rather than blinded) trial was chosen as it was deemed that it would be ethically and operationally challenging to provide placebo infusions to women during pregnancy. In particular, ferric carboxymaltose is black in colour, and thus would require either use of opaque tubing, a curtain and veil to conceal the tubing or a blindfold for the participant – none of which are likely to be feasible in practice. The recent pivotal RCT of intravenous versus oral iron in pregnancy employed an open-label design.

10. PARTICIPANT INCLUSION AND EXCLUSION CRITERIA

Participants meeting the following criteria will be included in the trial:

7. Confirmed singleton pregnancy at 13-26 weeks' gestation
8. Moderate to severe anaemia not requiring an immediate blood transfusion (Hb <10g/dl)
9. Negative malaria parasitaemia
10. Resident in the study catchment area of Blantyre and Zomba district
11. Plan to deliver at a health facility
12. Written informed consent (including assent if <18 years old)

Participants with the following criteria will be excluded:

7. Hypersensitivity to any of the study drugs
8. Clinical symptoms of malaria or bacterial infection
9. Any condition requiring hospitalization or serious concomitant illness
10. Chronic illness that may adversely affect foetal growth and viability

11. Clinically low haemoglobin requiring a blood transfusion (usually Hb <5g/dl)
12. Pre-eclampsia

11. STUDY SITES

The is a multicentre study to be carried out in the following sites:

Zomba Central Hospital (ZCH), in southern Malawi will be the main coordinating site, with 3 main satellite health centres (within a 5-minute drive distance) and other health centres as approved by the District Health Office where participants will be screened. This is a well-established clinical research site with all resources available to recruit eligible participants, prepare and administer the study drugs, monitor safety, treat adverse effects, and measure trial outcomes (22,230 women above 18 years, 4173 births per year, 13% with Hb <10g/dL respectively). This site recently participated in a large multicentre trial of antenatal anti-malarial treatment (IPTp), demonstrating the capability to undertake such studies [48].

Our second site will be in Blantyre (the hub of health research in Malawi) ~~at~~ and will involve three major health centres (HCs) namely Limbe HC, Bangwe HC, and Ndirande HC which are all less than 10 kilometres apart and other health centres as approved by the District Health Office. One of the health centres (Limbe HC) will be used as the recruitment site with the other two as satellite screening sites. Between them, they have 1113 new ANC attendees and 454 deliveries per month. Some of the laboratory analyses will be done on site e.g. haemoglobin and malaria tests while other lab tests will be done at the CoM Blantyre campus. The laboratory and pharmacy facilities at the CoM Blantyre campus have previously supported large trials. In Malawi, the average gestation at first ANC visit is 22 weeks, with 61% presenting by 24 weeks.

12. TRIAL INTERVENTIONS

This is an open-label trial and the intervention group will receive an IV iron given once at recruitment while the control group will receive oral iron through the routine health care system, given over 90 days or for the duration of the pregnancy, whichever comes first. Participants will be assigned to receive the following:

1. **Intravenous iron treatment course**: intravenous ferric carboxymaltose 1000mg (weight >50kg) or 20mg/kg (weight <50kg) given over 15min once at recruitment (Day 0);

OR

2. **Oral iron treatment course**: oral iron- 200mg ferrous sulphate (approx. 65mg elemental iron) twice daily for 90 days (reaching a total dose of approx. 11.7g) or the remaining duration of pregnancy, whichever is shorter.

Note that previous studies have estimated that iron absorption for ferrous sulphate in pregnancy approaches 20% from the second to third trimester [49], and thus this dose should ultimately be *higher* than the IV iron dose if adherence is 100%.

Both groups will receive IPTp with SP, 1500mg sulfadoxine and 75 mg pyrimethamine (3 fixed tablets of SP strength at 500mg/25mg) given at least 3 times during pregnancy, at least 4 weeks apart. As part of safety assessment, the IPTp SP course at 4 weeks' post-randomization will be directly observed by study staff, and the baseline dose will also be directly administered by study staff if the participant did not receive it through their health service. Otherwise, subsequent IPTp treatment courses will be administered under real-life programmatic conditions.

The oral iron will be given under real-life health service delivery conditions where the participant is given 3 months' oral iron at presentation to the antenatal clinic. This strategy is being employed as it is a key hypothesis in this trial that there is a reduced effect of oral iron on maternal anaemia due to poor adherence to the full course of treatment. Hence, this an effectiveness trial.

Each participant will additionally receive an insecticide-treated bed nets as per standard national guidelines through the health facility.

12.1. ALLOCATION TO TREATMENT

The investigator's knowledge of treatment must not influence the decision to recruit a particular participant or affect the order in which participants are recruited.

12.2. BREAKING THE BLIND

The trial is open label. However, the laboratory scientists will be blinded to the treatment of the participant in conducting the laboratory assays.

13. STUDY PROCEDURES

The recruitment and follow-up schedule, incorporating extended follow up of the infants and non-invasive haemoglobin measurements (SpHb), is shown in Figure 2.

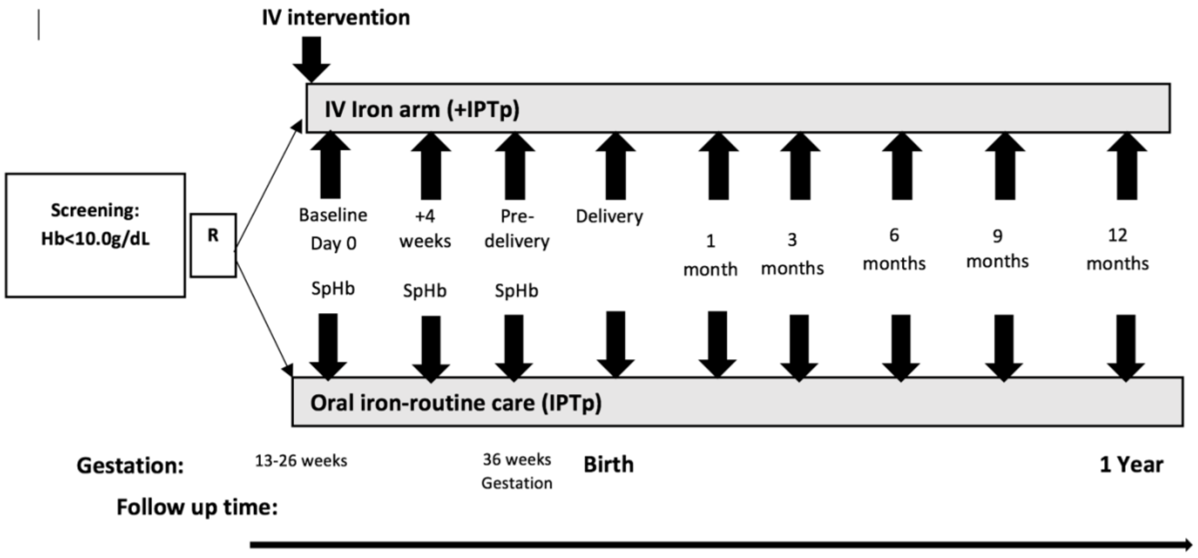

Figure 2: Study Schema

13.1. VISIT 0 [DAY 0]- SCREENING

Each morning at screening sites, a government-employed nurse trained to screen for study participants will begin with a health talk and include general information about the study (importance, aims and procedures). Health talks are routinely given to ANC attendees before the clinic begins. The screening nurse will then, as she/he processes ANC attendees, screen for potential participants by noting the residency, gestation age on clinical examination and Hb. Hb is measured routinely for all pregnant women presenting for their first ANC visit. She will complete a pre-screening form and then communicate to the recruitment site if there are potential participants. She will also request the potential participant to avail herself for an ultrasound scan (within two days from the initial pre-screening visit). A study vehicle will transport the pre-screened participants to the recruitment centre for further screening procedures including an ultrasound to confirm pregnancy and determine gestation age (i.e., crown-rump length for less than or equal to 16 weeks, and femur length and head and abdominal circumference for more than 16 weeks), before obtaining consent. If a vehicle is not available, transport costs will be reimbursed. The results of the ultrasound scan will be used if at all a discrepancy with the date of last menstrual cycle will arise.

13.2. VISIT 1 [DAY 0]- RECRUITMENT

If all the screening criteria including giving consent are met, then the study nurse will assign the participant a Study Number. This will be from a list of consecutive numbers specific for each recruitment site (Blantyre or Zomba). Participants will be administered a questionnaire to collect demographic, medical information, cost and time (travel and treatment) information, and-wellbeing and cognitive function before having a full physical examination by a clinician.

13.2.1. *Maternal quality of life and productivity*

Maternal quality of life will be measured using the Piper Fatigue Scale (PFS). This is a 12 item self-report instrument designed to measure fatigue. Mothers will be asked about the degree to which they are able to undertake their usual activities during the previous week to assess productivity consequences due to their condition.

13.2.1.1 Validation of the Piper Fatigue Scale (PFS)

In a subset of 50 women, we will administer a short version of the Depression, Anxiety and Stress Scale (DASS-21) and the Self-Reporting Questionnaire (SRQ) which provide a self-report measure of depression, anxiety and stress. The scores from these tests will be compared to those from the Piper Fatigue Scale (PFS), in order to examine concurrent validity of the PFS in the context of Malawi.

13.2.2. *Cognitive function*

Maternal cognition will be assessed using a digit span forward and backward test, and a mental rotation test.

13.2.3. *Blood sampling*

A 10ml venous blood sample will be collected for Full blood count (FBC), iron (ferritin, sTfR), hepcidin, phosphate, markers of bone health (e.g., vitamin D, calcium, alkaline phosphatase (ALP), procollagen type 1 N-terminal propeptide (P1NP), C1 Telopeptide, parathyroid hormone (PTH) and fibroblast growth factor-23 (FGF23)), ~~and~~ inflammatory markers (C-reactive protein and alpha-1 glycoprotein) and *Treponema pallidum*. Malaria parasitaemia will be sought by Rapid Diagnostic Test and microscopy. A filter paper sample of blood will be taken and stored for malaria PCR. For consistency with measurement of Hb (as a primary outcome) we shall require to take a finger-prick blood sample and use a HemoCue to determine the Hb.

13.2.4. *SpHb Measurement*

Haemoglobin will also be assessed non-invasively (pulse oximetry - SpHb) using the Rad-67 device.

13.2.5. *Study arm allocation*

The study nurse will then retrieve the sealed specific Study Number-marked envelope containing the study arm allocation and open it to allocate the participant study arm. The assigned study number will be recorded in the CRF (Clinical Record Form) and Pharmacy Log.

13.2.6. *Study drug administration*

For those allocated to the IV iron arm, they will be given Ferric carboxymaltose 20mg/kg up to 1000mg (women 50kg or above) in 250mL normal saline, administered intravenously over 15 minutes. The study drug will be diluted by the pharmacist following the pharmaceutical procedures which shall be additionally outlined in a specific SOP. The study drug will be provided to the clinician for administration. An intravenous cannula will be inserted following standard aseptic procedure. Skin will be cleaned with ethanol and a sterile cannula will be inserted into the forearm or hand by a skilled clinician, and the cannula will be fixed in place with a sterile tegaderm or clinical tape. The participant will be monitored over the 15 minutes of the infusion for any adverse events and if they develop these will be attended to promptly and treated according to standard hospital management guidelines. The participant will be observed for a further 45 minutes. Following completion, the cannula will be removed and placed in a biohazard container, and a band-aid applied to the arm. We will follow universal precautions whilst working with sharps. Administration of iron will be done in a room equipped with resources to manage a severe allergic reaction. Each room will have a 'crash' trolley which will contain adrenaline, hydrocortisone, intravenous fluids and antihistamines. It will also contain airway equipment (laryngoscope and endotracheal tubes) for emergencies. Oxygen will be available in the room. The contents and expiry of the crash trolley and availability of

oxygen will be confirmed each day by the study staff prior to administration of the drug. A clinician assigned to the trial will be in the treatment room when infusions are given. For those allocated to the oral iron arm, they will be given oral iron supplements to be taken twice daily for 90 days (or the duration of pregnancy, whichever is shorter). Women in the oral iron arm will not be encouraged to take the tablets more than routine antenatal advice as we want this arm to reflect as current practice as possible. Women in the IV iron arm will be instructed not to take oral iron; this will also be noted in the woman's passport so as to notify any other healthcare professionals she seeks care from during her pregnancy. All pregnant women will receive an insecticide-treated net (ITN) and IPTp first dose as standard policy in Malawi for the prevention of malaria in pregnancy from the ANC. Participants will then be dropped home by one of the research assistants to enable him/her to collect a detailed residency map in order to ease tracing of defaulters. Participants will be requested to return to the research clinic (at recruitment site) at any time that they feel ill during their pregnancy, otherwise, they will be encouraged to attend all scheduled ANC visits at their local health centre. All participants will be requested to deliver at the research site health facility or notify the research staff if in labour or delivery occurs at any other health facility.

13.3. VISIT 2 [DAY 28 ±2] - 4-WEEK FOLLOW-UP

All participants will be requested to return to the research clinic after 28(±2) days. They will be examined (as per standard ANC procedures) including an ultrasound scan before collecting a venous blood sample. If they are ill, relevant laboratory tests will be performed and they will be managed as per standard hospital guidelines. The participant will then be given SP 3 tablets as standard IPTp policy guidelines for Malawi as a directly observed therapy. Household economic data will also be collected. Participants will then be allowed to return home and instructed to continue their monthly ANC visits at their local health centre (these will normally be the health centres where they were screened).

13.3.1. *Maternal quality of life and productivity*

Maternal quality of life will be measured using the Piper Fatigue Scale (PFS). This is a 12 item self-report instrument designed to measure fatigue. Mothers will be asked about the degree to which they are able to undertake their usual activities during the previous week to assess productivity consequences due to their condition.

13.3.2. *Cognitive function*

Maternal cognition will be assessed using a digit span forward and backward test, and a mental rotation test.

13.3.3. *Blood sampling*

A 10ml venous blood sample for FBC, iron indices (ferritin, sTfR), CRP, alpha-1 glycoprotein, hepcidin, phosphate, markers of bone health (e.g., calcium, alkaline phosphatase (ALP), procollagen type 1 N-terminal propeptide (P1NP), C1 Telopeptide, parathyroid hormone (PTH) and fibroblast growth factor-23 (FGF23)), *Treponema pallidum* and malaria

parasitaemia (using RDT and microscopy). Some of the blood will be put on filter paper for malaria PCR and for resistance testing for positive samples.

For consistency with measurement of Hb (as primary outcome) we shall require to take a finger-prick blood sample and use a HemoCue to determine the Hb.

13.3.4. *SpHb Measurement*

Haemoglobin will be assessed non-invasively (pulse oximetry - SpHb) using the Rad-67 device.

13.3.5. *Reproductive Tract Infections Determination*

A serum sample will be collected for *Treponema Pallidum* testing. A vaginal swab will be done for bacterial vaginosis (Gram stain) and *T. vaginalis* and an endocervical swab for *C. trachomatis* and *N. gonorrhoeae*. If any diseases are detected, women will be notified and treated according to national guidelines. Samples will also be stored for microbiome analysis.

13.3.6. *Urinalysis*

Urine sample for urinalysis will be done and will include determination of parasites (using slide microscopy), glucose and proteins (using urine dipsticks).

13.3.7. *Stool Analysis*

A sample of stool will be collected for microscopy and stored for microbiome analysis

13.4. VISIT 3 [34 WEEKS GESTATION ±2 DAYS]

Participants will be visited in their home on a bi-weekly basis from 34 weeks' gestational age by a research nurse who will take a finger-prick sample of blood to determine the Hb level using a HemoCue. The research nurse will also ask the participant for the pill pack and he or she will count the number of pills the participant has left in order to determine adherence to iron in the oral iron study arm. If the participant is unwell during these visits they will be referred to visit the health facility (research clinic).

13.5. VISIT4 [36 WEEKS GESTATION ±2 DAYS] - PRE-DELIVERY FOLLOW-UP

All participants will be requested to return to the research clinic at 36 weeks' gestation. They will undergo a physical examination (as per standard ANC procedures) including ultrasound scan and a quality of life measure. They will further have some biological samples collected.

13.5.1. *Maternal quality of life and productivity*

Maternal quality of life will be measured using the Piper Fatigue Scale (PFS). This is a 12-item self-report instrument designed to measure fatigue. Mothers will be asked about the degree to which they are able to undertake their usual activities during the previous week to assess productivity consequences, due to their condition.

13.5.2. *Cognitive function*

Maternal cognition will be assessed using a digit span forward and backward test, and a mental rotation test.

13.5.3. *Blood sampling*

A 10ml venous blood sample will be collected for FBC, iron indices (ferritin, sTfR), CRP alpha-1 glycoprotein, hepcidin, phosphate, markers of bone health (e.g., calcium, alkaline phosphatase (ALP) and fibroblast growth factor-23 (FGF23)), *Treponema pallidum*, malaria parasitaemia (using RDT and microscopy). Some of the blood will be put on filter paper for malaria PCR and for resistance testing for positive samples.

For consistency with measurement of Hb (as a primary outcome) we shall require to take a finger-prick blood sample and use a HemoCue to determine the Hb.

13.5.4. *SpHb Measurement*

Haemoglobin will be assessed non-invasively (pulse oximetry - SpHb) using the Rad-67 device.

13.5.5. *Reproductive Tract Infections Determination*

A serum sample will be collected for Treponema Pallidum testing. Per vaginal swab will be done for bacterial vaginosis (Gram stain) and T. vaginalis and an endocervical swab for *C. trachomatis* and *N. gonorrhoeae*. If any diseases are detected, women will be notified and treated according to national guidelines. Samples will also be stored for microbiome analysis.

13.5.6. *Urinalysis*

Urine sample for urinalysis will be done and will include determination of parasites (using slide microscopy), glucose and proteins (using urine dipsticks).

13.5.7. *Stool analysis*

A sample of stool will be collected for microscopy and further stored for microbiome analysis.

13.6. VISIT 5 -6 [38 AND 40 WEEKS GESTATION ±2 DAYS]

Participants will be visited in their home bi-weekly until delivery by a research nurse who will take a finger-prick sample of blood to determine the Hb level using a HemoCue. If the participant is unwell during these visits they will be referred to visit the health facility (research clinic).

13.7. VISIT 7 – DELIVERY [+1 DAY]

All participants will be encouraged to return to the recruitment centres for delivery (unless the index pregnancy is defined as a high-risk pregnancy requiring delivery at the tertiary referral hospital (QECH) for Blantyre site). Participants delivering at home or at other health facilities will be encouraged to come within 24 hours to the research site for assessment. The study will have 24-hour cover of the delivery suite at the recruitment centres so that all data on deliveries are captured. Detailed physical examination will be done on admission and detailed clinical data on the progress of the delivery. Maternal venous blood sample will be taken (for laboratory tests as below). Delayed cord clamping procedures will be standardized. Immediately after delivery, we will record Apgar scores, and the neonate will have a full physical examination including measurement of

birthweight, length, head circumference and details of any congenital malformations. We will record whether the birth was vaginal, vaginal but assisted, or caesarean, and the indications for assisted/ caesarean births; we will also record maternal adverse events, including haemorrhage, and episiotomy. Cost information (travel and treatment) will also be collected.

13.7.1. *Blood sampling*

Maternal venous blood sample (10ml) will be collected for FBC, iron indices (ferritin, sTfR), CRP, AGP, Phosphate and malaria RDT and microscopy and filter paper for malaria PCR and for resistance testing for positive samples. For consistency with measurement of Hb (as a primary outcome) we shall require to take a finger-prick blood sample and use a HemoCue to determine the Hb.

13.7.2. *Placental histology*

A histological full thickness placental sample about 2 cm long x 1 cm wide will be collected into 40 ml of 10% neutral buffered formalin for malaria histological staining and examination, a cord blood smear for malaria microscopy. A placental blood smear will also be collected for microscopy, and a filter paper for malaria PCR. The placenta will be weighed and results recorded in the CRF. Sections of placenta will be stored and may be used for subsequent analyses related to biochemical, immune and metabolic maternal, neonatal or infant/ child health parameters. Mothers will be informed these samples may be used and that if assays are not available in Malawi, they may be sent overseas for analysis.

13.7.3. *Cord blood*

A cord blood sample will be collected for haemogobin, a smear for malaria microscopy and PCR, and iron indices. Samples will be stored biochemical, immunological and metabolic

13.7.4. *Infant neurodevelopment*

We will measure Auditory Brainstem Responses (ABRs) within 48 hours of delivery. ABRs are reflective of maturation of auditory pathway at various levels (e.g. acoustic nerve level to brainstem level). There is a rapid maturation of the ABR during the perinatal period that is influenced by the degree of myelination, neuronal development, synaptic function, and axonal growth in the auditory nervous system (i.e., changes we expect to be influenced by level of iron). This assessment does not require any participation by the child, indeed it is possible when the child is sleeping in their mother's arms. A signal (sound) is provided to the child, and the response is measured. The test is non-invasive, and only requires fitting of electrodes on the scalp. It does not pose any risk to the child and take less than 20 minutes per child. Local clinicians and nurses will be trained on how to administer these tests.

13.7.5. *Stool sample collection*

A sample of stool (meconium) will be collected from the new-born for microscopy and stored for microbiome analysis.

13.8. VISIT 8 - 28 DAYS POSTPARTUM/POSTNATAL ±2 DAYS]

All participants will be requested to return to the research clinic together with their infants for a detailed medical examination of both the mother and the baby.

13.8.1. *Maternal quality of life and productivity*

Maternal quality of life will be measured using the Piper Fatigue Scale (PFS). This is a 12-item self-report instrument designed to measure fatigue. Mothers will be asked about the degree to which they are able to undertake their usual activities during the previous week to assess productivity consequences due to their condition.

13.8.2. *Maternal cognitive function*

Maternal cognition will be assessed using a digit span forward and backward test, and a mental rotation test.

13.8.3. *Blood sampling*

Maternal venous blood sample (10ml) will be collected for FBC, iron indices (ferritin, sTfR), CRP alpha-1 glycoprotein, phosphate and malaria RDT, microscopy and filter paper for malaria PCR for resistance testing only for positive samples. For consistency with measurement of Hb (as a primary outcome) we shall require to take a finger-prick blood sample and use a HemoCue to determine the Hb. We will also collect 2ml of expressed breast milk for iron indices measurements. These samples will be stored in order to assess other biochemical, immunological and metabolic factors associated with maternal, neonatal or infant/child health.

Similarly, a venous blood sample of up to 2ml will be collected from the infant for, malaria RDT, microscopy and filter paper for PCR, iron indices determination and immunological assessment.

13.8.4. *Child anthropometry*

Infant weight, length and head circumference will be measured using established, calibrated tools.

13.8.5. *Child vaccination status*

Details of child's vaccination status will be recorded from medical records.

13.8.6. *Infant neurodevelopment*

We will measure Auditory Brainstem Responses (ABRs) while the child is sleeping. A signal (sound) is provided to the child, and the response is measured. The test is non-invasive, and only requires fitting of electrodes on the scalp. It does not pose any risk to the child and take less than 20 minutes per child.

13.8.7. *Child dietary diversity*

Dietary diversity will be assessed using a caregiver reported survey on food groups consumed in the past 7 days for children who have started complementary foods.

13.8.8. *Maternal psychological health*

Maternal psychologic al health will be measured using the short version of Depression, Anxiety and Stress Scale (DASS-21) [50], and Edinburg Postpartum Depression Scale (EPDS) that provide a self-report measure of depression, anxiety and stress. We will also ask information on psychiatric history, family relationship, social support, stressful events, infants wellbeing, and breast feeding pattern.

13.8.9. *Stool sample collection*

A sample of stool will be collected from mother and child for microscopy and stored for microbiome analysis

13.8.10. *Consent for involvement in the follow up (extension study)*

After provision of detailed information and the opportunity to ask questions (and to discuss with their family), mothers who have not yet been consented for the REVAMP-Extend component of the trial will be invited to provide written informed consent for ongoing involvement in the REVAMP extension cohort study. Participants providing consent will continue through the study visits described below. For participants declining further involvement, the 1-month postpartum visit will be the final study visit. For participants who provided consent to the trial and the extension at baseline, we will not repeat consent but will ensure they continue to understand study procedures and are happy to participate.

13.8.11. *Consent for Involvement in the REVAMP-Bone Substudy*

After provision of detailed information and the opportunity to ask questions (and to discuss with their family), mothers (N=200) will be invited to provide written informed consent for their participation and the participation of their infant (N=200) in the REVAMP-Bone Substudy. Participants providing consent will continue through the study visits described below.

13.8.12. *Consent for involvement in the REVAMP-Neuro Substudy*

After provision of detailed information and the opportunity to ask questions (and to discuss with their family), mothers (N=60) will be invited to provide written informed consent for the participation of their infant (N=60) in the REVAMP-Neuro Substudy. Participants providing consent will continue through the study visits described below.

13.9. VISIT 9 - 3 MONTHS POST-PARTUM (+/- 14 DAYS)

All participants will be requested to return to the research clinic together with their infants for a detailed medical examination of both the mother and the baby.

13.9.1. *Household food insecurity*

Household food insecurity will be assessed through caregiver's report with the use of the cross-culturally validated Household Food Insecurity and Access Scale.

13.9.2. *Maternal psychological health*

Maternal psychological health will be measured using the short version of Depression, Anxiety and Stress Scale (DASS-21) and EPDS. These tools provide a self-report measure of

depression, anxiety and stress. Mother-child interaction will be assessed by Mother-Infant Bonding Scale (MIBS), which is a modified version of the Postpartum Bonding Questionnaire.

13.9.3. *Blood sampling*

Maternal venous blood sample (10ml) will be collected for FBC, iron indices (ferritin, sTfR), CRP, alpha-1 glycoprotein, hepcidin, phosphate, markers of bone health (e.g., calcium, alkaline phosphatase (ALP), procollagen type 1 N-terminal propeptide (P1NP), C1 Telopeptide, parathyroid hormone (PTH) and fibroblast growth factor-23 (FGF23)), and malaria RDT, microscopy and filter paper for malaria PCR for resistance testing only for positive samples. For consistency with measurement of Hb (as a primary outcome) we shall require to take a finger-prick blood sample and use a HemoCue to determine the Hb. Similarly, a venous blood sample of up to 3ml will be collected from the infant for Hb, malaria RDT, microscopy and filter paper for PCR, iron indices determination, hepcidin, phosphate, markers of bone health (e.g., vitamin D, calcium, alkaline phosphatase (ALP), and fibroblast growth factor-23 (FGF23)) and immunological assessment.

13.9.4. *Child anthropometry*

Infant weight, length and head circumference will be measured using established, calibrated tools.

13.9.5. *Child vaccination status*

Details of child's vaccination status will be recorded from medical records.

13.9.6. *Child dietary diversity*

Dietary diversity will be assessed using a caregiver reported survey on food groups consumed in the past 7 days for children who have started complementary foods.

13.10. VISIT 10 - 6 MONTHS POST-PARTUM (+/- 14 DAYS)

All participants will be requested to return to the research clinic together with their infants for a detailed medical examination of both the mother and the baby.

13.10.1. *Household food insecurity*

Household food insecurity will be assessed through caregiver's report with the use of the cross-culturally validated Household Food Insecurity and Access Scale.

13.10.2. *Maternal psychological health*

Maternal psychological health will be measured using the short version of Depression, Anxiety and Stress Scale (DASS-21) and EPDS. These tools provide a self-report measure of depression, anxiety and stress. Mother-child interaction will be assessed by MIBS.

13.10.3. *Blood sampling*

Maternal venous blood sample (10ml) will be collected for FBC, iron indices (ferritin, sTfR), CRP alpha-1 glycoprotein, phosphate, markers of bone health (e.g., calcium and alkaline phosphatase (ALP)) and malaria RDT, microscopy and filter paper for malaria PCR for resistance testing only for positive samples. For consistency with measurement of Hb (as a

primary outcome) we shall require to take a finger-prick blood sample and use a HemoCue to determine the Hb.

Similarly, a venous blood sample of up to 3ml will be collected from the infant for Hb, malaria RDT, microscopy and filter paper for PCR, iron indices determination, hepcidin, phosphate, markers of bone health (e.g., calcium, alkaline phosphatase (ALP), vitamin D and fibroblast growth factor-23 (FGF23)) and immunological assessment.

13.10.4. *Child anthropometry*

Infant weight, length and head circumference will be measured using established, calibrated tools.

13.10.5. *Child vaccination and vitamin A supplementation status*

Details of child's vaccination and vitamin A supplementation status will be recorded from medical records.

13.10.6. *Child dietary diversity*

Dietary diversity will be assessed using a caregiver reported survey on food groups consumed in the past 7 days for children who have started complementary foods. [52]

13.10.7. *Stimulating caregiving*

The Family Care Indicators, a 9-item caregiver-report measure will be used to assess stimulating caregiving. [53]

13.10.8. *Infant neurodevelopment*

We will measure Auditory Brainstem Responses (ABRs) while the child is sleeping. A signal (sound) is provided to the child, and the response is measured. The test is non-invasive, and only requires fitting of electrodes on the scalp. It does not pose any risk to the child and take less than 20 minutes per child.

We will measure Event Related Potentials (ERPs) using electroencephalography (EEG) methods.

We will measure Bayley Scales of Infant Development

13.10.9. *Stool sample collection*

A sample of stool will be collected from child for microscopy and stored for microbiome analysis

13.10.10 Child bone health

In a subset of children who have been consented for the REVAMP-Bone Substudy (N=200), we will take x-rays of wrist and knee to identify the presence of radiological Ricketts using standard techniques. The use of x-rays is a safe and non-invasive method for the assessment of bones.

13.11. VISIT 11 - 9 MONTHS POST-PARTUM (+/- 14 DAYS)

All participants will be requested to return to the research clinic together with their infants for a detailed medical examination of both the mother and the baby.

13.11.1. *Household food insecurity*

Household food insecurity will be assessed through caregiver's report with the use of the cross-culturally validated Household Food Insecurity and Access Scale.

13.11.2. *Maternal psychological health*

Maternal psychological health will be Maternal measured using the short version of Depression, Anxiety and Stress Scale (DASS-21) and EPDS. These tools provide a self-report measure of depression, anxiety and stress.

13.11.3. *Blood sampling*

Maternal venous blood sample (10ml) will be collected for FBC, iron indices (ferritin, sTfR), CRP alpha-1 glycoprotein, and malaria RDT, microscopy and filter paper for malaria PCR for resistance testing only for positive samples. For consistency with measurement of Hb (as a primary outcome) we shall require to take a finger-prick blood sample and use a HemoCue to determine the Hb. Similarly, a venous blood sample of up to 3ml will be collected from the infant for Hb, malaria RDT, microscopy and filter paper for PCR, iron indices determination, hepcidin, phosphate, biological markers of bone health (e.g., calcium and fibroblast growth factor-23 (FGF23)) and immunological assessment.

13.11.4. *Child anthropometry*

Infant weight, length and head circumference will be measured using established, calibrated tools.

13.11.5. *Child vaccination and vitamin A supplementation status*

Details of child's vaccination and vitamin A supplementation status will be recorded from medical records.

13.11.6. *Child dietary diversity*

Dietary diversity [50] will be assessed using a caregiver reported survey on food groups consumed in the past 7 days for babies who have started complementary foods.

13.12. VISIT 12: 12 MONTHS POST-PARTUM (+/- 14 DAYS)

All participants will be requested to return to the research clinic together with their infants for a detailed medical examination of both the mother and the baby.

13.12.1. *Household food insecurity*

Household food insecurity will be assessed through caregiver's report with the use of the cross-culturally validated household food insecurity and access scale.

13.12.2. *Maternal psychological health*

Maternal psychological health will be measured using the short version of Depression, Anxiety and Stress Scale (DASS-21) that provides a self-report measure of depression, anxiety and stress. Mother-child interaction will be assessed using MIBS.

13.12.3. *Blood sampling*

Maternal venous blood sample (10ml) will be collected for FBC, reticulocytes count, iron indices (ferritin, sTfR), CRP alpha-1 glycoprotein, and malaria RDT, microscopy and filter paper for malaria PCR for resistance testing only for positive samples. For consistency with measurement of Hb (as a primary outcome) we shall require to take a finger-prick blood sample and use a HemoCue to determine the Hb.

Similarly, a venous blood sample of up to 3ml will be collected from the infant for Hb, malaria RDT, microscopy and filter paper for PCR, iron indices determination, hepcidin. phosphate, biological markers of bone health (e.g., calcium, fibroblast growth factor-23 (FGF23), vitamin D and alkaline phosphatase (ALP)) and immunological assessment.

13.12.4. *Child anthropometry*

Infant weight, length and head circumference will be measured using established, calibrated tools.

13.12.5. *Child vaccination and vitamin A supplementation status*

Details of child's vaccination and vitamin A supplementation status will be recorded from medical records.

13.12.6. *Child dietary diversity*

Dietary diversity [50] will be assessed using a caregiver reported survey on food groups consumed in the past 7 days for babies who have started complementary foods.

13.12.7. *Infant neurodevelopment*

We will measure Event Related Potentials (ERPs) using electroencephalography (EEG) methods (see Neurocognitive Assessments Used in Infant Follow-Up section below). We will measure Bayley Scales of Infant Development (see Neurocognitive Assessments used in Infant Follow-Up section below). In a subset of infants (N=60), we will measure structural neurodevelopment using low field portable MRI technology (see Neurocognitive Assessments used in Infant Follow-Up section below).

13.12.8. *Child bone health*

In a subset of children who have been consented for the REVAMP Bone Substudy (N=200), we will take x-rays of wrist and knee to identify the presence of radiological Ricketts using standard techniques. The use of x-rays is a safe and non-invasive method for the assessment of bones.

13.13. UNSCHEDULED SICK VISIT [ANYTIME DURING STUDY FOLLOW-UP]

Participants will be encouraged to attend the research clinic when sick. The participants will be managed according to national standard treatment guidelines. Blood sample for malaria RDT (and microscopy if RDT positive) will be taken. Some of the blood will be put on filter paper for malaria PCR and for resistance testing for positive samples. Blood will be

sent for culture if bacteraemia is clinically suspected. Cost information will also be collected.

14. LABORATORY PROCEDURES

At each of the two recruitment sites (Limbe in Blantyre and ZCH in Zomba), we shall have a small laboratory with a lab technician where initial sample processing will be done. The site labs will have small laboratory equipment for sample collection, separation and storage (at -20°C). They will have a microscope and area for staining of malaria and other slides before reading. Lab technicians will use SOPs for all their laboratory processing of samples. These SOPs will be developed by senior laboratory technologist and reviewed by principal investigators. Samples will be transferred for long-term storage to the CoM, Blantyre campus where there is -80°C freezer. This temperature of the freezer is monitored daily and there are backup systems in case it breaks down. Blood for culture will be sent to the Malawi-Liverpool Research Laboratories also at the CoM in Blantyre for processing. They have a quality assured blood culture processing system which will be used for a fee. Some samples will be exported out of the country for more advanced assays that are not currently available in Malawi.

14.1. FULL BLOOD COUNT

A Sysmex haematology analyser will be used for full blood count to determine the Hb level and red cell indices. For reticulocyte count determination, whole blood will be mixed with reticulocyte stain, then left to stand for 10min before being double read under a microscope.

14.2. MALARIA TESTING

Testing for malaria will be done using an RDT following manufactures guidelines. Whole blood will be placed on a RDT test strip and read. If positive, a thick blood smear will be prepared for parasite count and a thin blood smear for species differentiation (although we expect >97% to be P. falciparum). The smears will be stained with May-Grunewald-Giemsa using standard techniques. All smears will be double read by certified laboratory technicians (each reading blinded of the others results). Where there are discrepant results (positive/negative or a 50% difference in the parasite counts), these slides will be read by a third person. Blood from participants in whom parasites are detected can be further analysed for parasite clonality, levels of parasite gene expression and the abundance and characteristics of parasite proteins.

Placental histology will be examined for malaria using standard approaches. Placental biopsies will be fixed in formalin, embedded in paraffin and 5 mm sections cut onto glass slides. After rehydration slides will be stained with Giemsa and/or haematoxylin and eosin and passed through graded alcohols and coverslipped. Examination by microscopy will note the presence of malaria-infected erythrocytes, malaria pigment in fibrin, and malaria pigment in host leukocytes in the placental blood spaces. Placentas will be classed as

uninfected; past infection (malaria pigment, no parasites); or active infections (parasites with or without malaria pigment).

14.3. BLOOD FOR CULTURE

A venous blood sample (1 to 2 ml) will be inoculated into BACTEC Myco/F-Lytic culture vials and incubated in a BACTEC 9050 automated culture system (Becton Dickinson) for 56 days. Sub-culturing, susceptibility testing, and isolate identification will be performed by standard techniques [55].

14.4. BLOOD SAMPLE PREPARATION FOR LONG-TERM STORAGE FOR IRON AND INFLAMMATORY MARKER ASSAYS

Blood will be drawn using standard venipuncture techniques and serum separated from the blood cells as soon as possible. Samples will be allowed to clot for one hour at room temperature, centrifuged for 10 minutes at 4 °C, and serum extracted. Serum C-reactive protein and alpha-1 glycoprotein, ferritin, sTfR will be analysed on Modular P800 and Modular Analytics E170 systems (Roche). Blood will also be stored in appropriate preservatives to enable further analysis of immune molecular aetiologies related to anaemia, infection and birth weight outcomes in pregnancy, which will be carried out at the Walter and Eliza Hall Institute of Medical Research, in Melbourne, Australia.

14.5. VAGINAL AND GUT MICROBIOME ANALYSIS

Vaginal and faecal infections will be tested using conventional microscopy and culture. Culture-free analysis of the microbiota will also be undertaken at the Walter and Eliza Hall Institute of Medical Research in Melbourne, Australia or partners, using approaches such as 16S rRNA gene sequencing on microbial DNA.

Table 8: Overview of (laboratory) measurements

Measurements		MEASUREMENTS on the MOTHER													
		Time Point													
	Sample type	Test	Screening (visit 0)	Enrolment (visit 1)	Day 28±2 (v2)	Week 36 (v4)	Week (HV3/5/6) wk34, 38 & 40	Delivery (visit 7)	DAY 28±2 Post-partum (visit 8)	3 month (visit 9) ±14 days	6 month (visit 10) ±14 days	9 month (visit 11) ±14 days	12 month (visit 12) ±14 days	Unscheduled or sick visits	Is the test locally available?
**01.**	**Capillary blood (finger prick)**	Haemoglobin (Hb)	X	X	X	X	X	X	X	X	X	X	X	X	Yes
**02.**		Malaria RDTs (mRDT)	X		X	X		X	X	X	X	X	X	X	Yes
**03.**		Resistance testing if Filter paper is mRDT positive	X	X	X	X	X	X		X	X	X	X	X	No
**04.**	**Venous blood**	FBC (full blood count)		X	X	X		X	X	X	X	X	X	X	Yes
**05.**		Malaria microscopy		X	X	X		X	X	X*	X*	X*	X*	X	Yes
**06.**		Hb (by HemoCue)		X	X	X		X	X	X	X	X	X	X	Yes
**07.**		Hb (by Sysmex analyser)			X	X		X	X	X	X	X	X		Yes
		Hb (by Coulter Counter)		X	X	X									
**08.**		Blood culture												X#	Yes
**09.**		Malaria PCR		X	X	X		X	X	X	X	X	X	X	No
**10.**		Resistance testing if Filter paper is mRDT positive		X	X	X		X	X	X	X	X	X	X	No
**11.**		Ferritin (serum)		X	X	X		X	X	X	X	X	X		No
**12.**		sTfR (serum)		X	X	X		X	X	X	X	X	X		No
**13.**		Folate		X	X	X		X	X	X	X	X	X		No
**14.**		Cobalamin (Vit B12)		X	X	X		X	X	X	X	X	X		No
**15.**		Phosphate (serum)		X	X	X		X	X	X	X	X	X		No
**16.**		Retinol/ tocopherol		X	X	X		X	X	X	X	X	X		No
**17.**		CRP (C-reactive protein)		X	X	X		X	X	X	X	X	X		No
**18.**		Alpha-1 glycoprotein		X	X	X		X	X	X	X	X	X		No
**19.**		Treponema Pallidum test	X	X	X	X									No
**20.**		FGF23		X	X	X				X					No
**21.**		Calcium		X	X	X				X	X				No
**22.**		Vitamin D		X											No
**23.**		Alkaline Phosphatase (ALP)		X	X	X				X	X				No
**24.**		Procollagen type 1 N		X	X					X					No

Measurements		MEASUREMENTS on the MOTHER													
		Time Point													
	**Sample type**	**Test**	**Screening (visit 0)**	**Enrolment (visit 1)**	**Day 28±2 (v2)**	**Week 36 (v4)**	**Week (HV3/5/6) wk34, 38 & 40**	**Delivery (visit 7)**	**DAY 28±2 Post-partum (visit 8)**	**3 month (visit 9) ±14 days**	**6 month (visit 10) ±14 days**	**9 month (visit 11) ±14 days**	**12 month (visit 12) ±14 days**	**Unscheduled or sick visits**	**Is the test locally available?**
		propeptide (P1NP)													
**25.**		C1 Telopeptide		X	X					X					No
**26.**		Parathyroid hormone (PTH)		X	X					X					No
**27.**		Hepcidin		X	X	X				X					No
**28.**		blood culture test if bacteraemia clinically suspected			X	X		X	x	X	X	X	X	X	Yes
**29.**	**Vaginal swab**	for bacterial vaginosis (Gram stain) and T. vaginalis tests			X	X									No
**30.**	**Endocervical swab**	for *C. trachomatis* and *N. gonorrhoeae*			X	X									No
**31.**	**Urine sample**	for microscopic parasites analysis			X	X									Yes
**32.**		glucose and protein analysis using dipstick			X	X									Yes
**33.**	**Stool sample**	for microbiome analysis			X	X			X						No
**34.**	**Placental biopsy**	for Malaria histological staining and examination						X							No
**35.**	**Placental blood smear**	for microscopic malaria test						X							Yes
**36.**		for malaria PCR						X							No
**37.**	**Breastmilk sample**	For iron markers							X						No
**38.**	**Rad-67**	Non-invasive Hb		X	X	X									Yes
		MEASUREMENTS on the INFANT													
**39.**	**Cord blood**	Malaria microscopy						X							Yes
**40.**		Hb by Hemocue®						X							Yes
**41.**		Full Blood Count						X							Yes
**42.**		Malaria PCR						X							No
**43.**		Ferritin						X							No
**44.**		STfR						X							No

Measurements		MEASUREMENTS on the MOTHER													
		Time Point													
	Sample type	Test	Screening (visit 0)	Enrolment (visit 1)	Day 28±2 (v2)	Week 36 (v4)	Week (HV3/5/6) wk34, 38 & 40	Delivery (visit 7)	DAY 28±2 Post-partum (visit 8)	3 month (visit 9) ±14 days	6 month (visit 10) ±14 days	9 month (visit 11) ±14 days	12 month (visit 12) ±14 days	Unscheduled or sick visits	Is the test locally available?
45.	Stool sample	For Microbiome analysis						X	X		X		X		No
46.	Venous blood	malaria RDT							X	X	X	X	X	X	Yes
47.		Malaria microscopy							X	X	X	X	X	X*	Yes
48.		Malaria PCR							X	X	X	X	X	X	No
49.		Hb by Hemocue							X	X	X	X	X		Yes
50.		Ferritin (serum)							X	X	X	X	X		No
51.		sTfR (serum)							X	X	X	X	X		No
52.		Full Blood Count							X	X	X	X	X		Yes
53.		CRP (C-reactive protein)							X	X	X	X	X		Yes/No
54.		Alpha-1 glycoprotein							X	X	X	X	X		No
55.		Phosphate								X	X	X	X		No
56.		FGF23								X	X	X	X		No
57.		Calcium								X	X	X	X		No
58.		Vitamin D								X	X		X		No
59.		Alkaline Phosphatase (ALP)								X	X		X		No
60.		hepcidin								X	X	X	X		No
61.	Xray	Wrist and knee x-rays for radiological Ricketts assessment									X		X		Yes
62.	Low Field MRI	Low Field MRI of brain											X		Yes
		FBC, full blood count; HV, home visit; Hb, haemoglobin; mRDTs, malaria rapid diagnostic tests; sTfR, serum transferrin receptor; CRP, C-reactive protein; AGP, alpha 1-acid glycoprotein; FGF23, fibroblast growth factor-23; ALP, alkaline phosphatase; P1NP, procollagen Type-1 N propeptide; PTH, parathyroid hormaone; MRI, magnetic resonance imaging													

*If MRDT positive
#if indicated

15. NEURO-COGNITIVE ASSESSMENTS USED IN INFANT FOLLOW UP

15.1 Background

We will use highly sensitive assessments of memory and executive control to examine the effects of iron on children's brain development. Beyond the important effects of iron on the brain, we will also examine the neural mechanisms underlying cognitive development and how infection exposures and environmental adversity in this context may shape neural development. Event Related Potentials (ERPs) are ideal measures of infant cognitive function because they are sensitive to, and reflective of, brain functions directly affected by iron. They are able to detect subtle yet meaningful delays that have long-term consequences. [56-58] ERPs are neurophysiologic measures that rely on recordings of the brain's electrical activity via electroencephalogram (EEG) in response to stimuli. ERPs can be recorded noninvasively at the scalp surface in minutes. They are used in routine clinical assessments in children in tertiary care hospitals across Australia, America, and Europe, typically in the investigation and diagnosis of epilepsy and seizure disorders. [59-60]

Observational studies have reported significant associations between iron status in young children and ERP-derived neural correlates of memory and executive control. [61-63] For instance, Geng et al [63] reported that 2-month-old Chinese children who were iron sufficient at birth were able to recognize their mother's voice from a stranger's voice whereas iron deficient children were not, as indicated by the ERP late slow wave component in frontal-central and parietal-occipital locations of the scalp. Another study in American children found that, compared to iron deficient children, iron sufficient 9-month-olds showed a larger attentional response to their mother's face compared to a stranger's, indicated by the ERP negative component. [61] They also reported a better updating of memory for the stranger's face, indicated by the positive slow wave component. Children with iron deficiency anaemia showed similar patterns only at 12 months of age, consistent with delayed cognitive development in this group. [61] However, to date, no randomized controlled trial (RCT) of iron interventions has used ERPs.

Differences in cognitive functioning detected using ERPs early in life have been shown to be predictive of later cognition. [58,64,65] For instance, follow up studies of children with and without iron deficiency anaemia in the first year of life found significant differences using electrophysiology in the auditory and visual systems of the brain at 4 years of age and inhibitory control at 10 years of age. [64-65] Results from longitudinal studies support the hypothesis that iron deficiency in early life causes hypomyelination and has long term detrimental effects on brain function which can be detected with sensitive measures such as ERPs.

We will use ERPs to measure the impartial and direct effects of iron on brain development, by examining functions and areas of the brain which are especially sensitive to iron, to ensure we capture meaningful effects of iron supplementation on early cognition. Our study intends to build on observational ERP literature, leverage an existing rigorously controlled field RCT, to be the first study to examine the effects of antenatal high dose iron interventions on ERP-derived cognitive functions in young children.

15.2 Procedures

ERPs will be measured in response to auditory and visual stimuli, recorded from 32 scalp electrode sites, using established procedures. The study centers will have a designated ERP testing room, outfitted with the equipment, dark curtains, an air conditioning unit, and a screen for visual stimuli.

When caregivers and their children return for the 6 and 12 months visits at which ERPs will be measured, they will be walked through each procedure in detail. They will be shown a video detailing the procedure and objectives of the research. Children will be fitted with an ERP cap (resembling a swim cap) and seated on their mothers' laps in front of a screen. Testing will begin after a period of familiarization with the cap, screen, instrument, and with the tester. We intend to spend no more than 30 minutes with each child to avoid poor data collection when a child is tired.

At birth, one and six months of age; we will assess children's Auditory Brainstem Responses (ABRs). ABRs are reflective of maturation of auditory pathway at various levels (e.g. acoustic nerve level to brainstem level). There is a rapid maturation of the ABR during the perinatal period that is influenced by the degree of myelination, neuronal development, synaptic function, and axonal growth in the auditory nervous system (i.e., changes we expect to be influenced by level of iron).

At 6 and 12 months of age; we will run ERP paradigms of memory and executive function. Visual and auditory stimuli for the ERP tasks will be culturally appropriate. The order of the ERP tasks will alternate between children to ensure that the order does not bias performance. On a daily basis, recorded data will be shared with the team in Australia, where it will be processed (to separate adequate from poor EEG data), and averaged for components of interest. We will have regular Skype meetings between the Malawian and Australian team members to discuss and troubleshoot any issues.

In the cases where a child is not able to complete the ERP session, they will be invited to return the following day for a repeat session.

15.3 Feasibility

Electro physiologic methods have advanced greatly in recent years and simplifications to equipment and technique mean these methods are now portable and can be used to assess

brain development even in remote, low income settings, like Zomba and Blantyre. ERPs have been used in a field-based study in Pakistan and are currently being used in other resource-limited settings, such as The Gambia and South Africa. We are presently undertaking a large ERP study as part of a major trial of iron interventions in rural Bangladesh.

15.4 Behavioural child development measurements

At 6 and 12 months of age, we will administer the Bayley Scales of Infant and Toddler Development III and a behavioural test of memory and attention. The results from the behavioural tests will be used to assess other domains of child development than those gathered using ERPs (i.e., fine and gross motor, cognitive, language, and socio-emotional development), and will be used to corroborate the ERP results. The use of the Bayley Scales allows us to describe the findings of the interventions to policymakers, clinicians and other end users of the trial results.

15.5 Low Field portable MRI (Hyperfine)

Neuroimaging offers an opportunity to assess the impact of high dose iron during gestation on child neurodevelopment. At 12 months of age, in a subset of children (N=60), we will obtain structural images of the brain using low field portable MRI technology (Hyperfine). Low field portable MRI technology for neuroimaging has been established by Hyperfine, and has been adapted for low-income settings. Images will be analysed using software developed by international partnerships. Images will be taken in Zomba, Malawi. Data will be uploaded and shared with our team in Melbourne and analysed by the Developmental Imaging team at the Murdoch Children's Research Institute, Melbourne. All analyses will be undertaken by staff blinded to the intervention arm and other exposures of the participant. In addition, experienced paediatric radiologists from the Royal Children's Hospital, Melbourne, will provide a clinical MRI report for every scan undertaken.

16. ARRANGEMENTS FOR ALLOCATING PARTICIPANTS TO TRIAL GROUPS

Screening will occur at one of the centres, and participants will then be requested to attend one of the two research centres (Limbe Health Centre or ZCH) for consent, randomisation and initial treatment with IV iron or administration of oral iron. A randomization code for treatment arms will be generated by an independent statistician in Melbourne and individual participant codes will be pre-packed (independently) in envelopes and sealed and held securely at research sites. The randomization code will be stratified according to the research site. The eligible participants who have met all inclusion/exclusion criteria will receive study medication assigned to the next available randomization number at the Day 0 during the first study visit. The study medication shall

be determined after the research staff open the specific participant envelope which will prescribe the participant's group allocation.

17. ASSESSMENT OF SAFETY

17.1. PRECAUTIONS IN DELIVERING FCM

As discussed above, there remains a risk of hypersensitivity reactions associated with infusion of FCM. For this reason, FCM continues to only be available for infusion in health care centres, where appropriate facilities are available for treatment of any severe reactions, even though these are rare.

Standard warning text is required by the FDA to be included in all the prescribing information of all IV irons (i.e. not just FCM, but all formulations, irrespective of the specific risk associated with that particular formulation) marketed in the USA, advising that patients may present with shock, clinically significant hypotension, loss of consciousness, or collapse. The text states that it is necessary to monitor patients for signs and symptoms of hypersensitivity during and after IV iron administration for at least 30 min and until clinically stable following completion of the infusion. In Australia, it is still routine to administer FCM only in contexts where equipment for management of hypersensitivity reactions are in place: i.e. with staff trained in the management of these reactions, and drugs (steroids such as hydrocortisone, antihistamines such as chloropheniramine, and adrenaline). It is likewise routine to ask patients to remain in the treatment centre for 30 minutes' post infusion to ensure their safety. We will follow these protocols when delivering FCM to women in our trial. Both trial sites are attached to large hospitals which can support the trial staff and, if necessary, provide urgent medical care. FCM administration will be overseen by research specific medical doctors in Blantyre and Zomba.

Protocol for delivering FCM

- FCM will be administered as an infusion in 250mL of 0.9% sodium chloride
- Infusion rate will be 250mL over 15 minutes.
  - The patency of the IV cannula must be confirmed before commencing any infusion to ensure there is no extravasation of iron, which can cause a permanent stain. Connect a 50mL 0.9% sodium chloride flush and allow this to infuse by gravity. If the flush does not work, do not use the line.
- All women should be observed for signs of adverse reaction which may be acute or delayed (see below).
- A medical officer will be available and able to reach the infusion room within 5 minutes
- Common adverse reactions to FCM include:
  - Headache, dizziness
  - Injection site reactions
  - Hypertension

- Elevated liver enzymes
- Hypophosphataemia

- Less common reactions include:
    - Nausea, abdominal pain, constipation, diarrhoea
    - Hypersensitivity including anaphylaxis
    - Paraesthesia and dysguesia
    - Hypotension
    - Tachycardia
    - Flushing
    - Back, joint or muscle pain
    - Pruritis and urticaria
    - Pyrexia, fatigue and malaise
    - Dyspnoea and bronchospasm
    - Syncope
- A member of the study nursing staff will remain in the same room as the patient during the infusion and for the 30 minutes following patient, to ensure any adverse serious reaction is rapidly detected and that the infusion can either be stopped or other appropriate treatments given.
- Vital signs to be monitored prior to infusion, 5 minutes after the start of infusion, at end of infusion, every prior to discharge, and if the participants feel unwell:
    - Heart Rate
    - Blood Pressure
    - Oxygen Saturations
    - Respiratory Rate
    - Conscious State
    - Temperature
- Foetal Heart Rate will be monitored prior to infusion, at end of infusion and prior to discharge.

17.2. DEFINITIONS OF ADVERSE EVENTS (AE)

Adverse Events (AE), adverse reactions (AR), unexpected adverse reactions, severity and serious adverse events/reactions will be defined in accordance with the EU guideline titles: 'Detailed guidance on the collection, verification and presentation of adverse reaction reports arising from clinical trials on medicinal products for human use, April 2004.' [65, 66] SUSARS are defined as all suspected adverse reactions related to the investigational product.

17.3. RECORDING OF ADVERSE EVENTS

17.3.1. *The time period for collecting AEs*

Non-serious adverse events and serious adverse events (SAE) will be collected from the time consent is given until the participant completes the study (the final visit or withdrawal).

17.3.2. *Method of detecting AEs*

At scheduled clinic visits, methods of detecting AEs will include AEs spontaneously reported by the subject and/or observed by the study staff, as well as AEs identified by means of a standard question to the subject such as "have you experienced any health problems since the last visit/ the last questioning?".

17.3.3. *Collection of AE data*

AEs will be recorded in the adverse event section of the CRF. For each AE, a description of the event, date of onset and resolution (and if applicable time), its severity, whether it constitutes a SAE or not, any action taken (e.g. other treatment given, change to study treatment, follow-up laboratory tests) and the outcome (continuing or resolved), will be given along with the investigator's assessment of relationship to study drug. Details of changes to the dosage schedule, any other treatment given or follow-up laboratory tests should be recorded on the appropriate pages of the CRF. All SAEs must be reported according to procedures described in the section 'reporting of serious adverse events'.

If a diagnosis of the subject's condition has been made, then the diagnosis should be recorded as the adverse event. However if a diagnosis of the subject's condition has not been made then the individual signs or symptoms should be recorded separately.

If an AE changes in frequency or severity during the study period, a new record of the AE should be recorded in the CRF.

17.3.4. *Assessment of severity*

The investigator will use clinical judgement to make an assessment of the severity of each AE.

17.3.5. *Assessment of causality*

The investigator will use clinical judgement to assess the relationship between investigational product and the occurrence of each adverse event. Alternative causes such as natural history of the underlying diseases, concomitant therapy, other risk factors and the temporal relationship of the event to the investigational product will be considered and investigated.

The investigator will make an assessment of the causality of the AE by answering "Yes" or "No" to the question "Is there a reasonable possibility of a causal relationship between the study drug and the AE?" in the CRF.

17.3.6. *Study Endpoints and symptoms anaemia*

Adverse events judged to be symptoms of anaemia or meeting the definition of any of the study outcome (severe anaemia) will not be recorded as adverse events unless they fulfil the criteria for an SAE or result in discontinuation of study treatment.

17.3.7. *HIV related disease*

In HIV infected subjects, adverse events judged to be expected because of HIV-related disease will not be recorded as adverse events unless they fulfil the criteria for a SAE or result in discontinuation of study treatment.

17.3.8. *Lack of efficacy and Disease Progression*

Any deterioration in the subject's condition after the subject has been enrolled in the study will be discussed with / assessed by the principal investigator. Where the deterioration is considered by the principal investigator to constitute a progression of anaemia or lack of effectiveness this will not be considered an AE unless it fulfils the criteria for an SAE or results in discontinuation of study treatment. Symptoms of unexpected disease progression (as assessed by the investigator) should be recorded as AEs.

17.3.9. *Abnormal laboratory values*

The duplicate reporting of laboratory tests as both laboratory findings and adverse events in the CRF should be avoided. Abnormal laboratory tests results will not be reported as AEs in the CRF unless they fulfil the criteria for a SAE or result in discontinuation of study treatment. They will be evaluated in the overall safety analysis**.** If an abnormal laboratory value is associated with clinical signs and symptoms, the sign/symptom should be reported as an AE while the associated test result is recorded in the appropriate CRF section**.**

17.3.10. *Overdose*

Use of study medication in doses in excess of that specified under the section on Trial Interventions should be recorded as an AE. An overdose without associated symptoms should be recorded as an AE of "Overdose".

17.4. REPORTING OF SAES

17.4.1. *Reporting by the Investigator to the Study Safety Monitor and Sponsor*

The investigators must report all SAEs that occur during the course of the study to the Sponsor within 24 hours of the investigational site becoming aware of it. All SAEs have to be reported, whether or not considered causally related to the investigational product or study procedure(s). All SAEs will be reported on the SAE form provided. The reporting will be made by research medical doctors in Blantyre and Zomba sites.

Follow-up information on SAEs must also be reported by the investigator within the same timeframe. In case of doubt about whether an event fulfils serious criteria, the case should be reported to the safety monitor who will assess whether the event should be reported as an SAE.

At both study sites, Blantyre and Zomba, there will be a Co-investigator assigned to be the Safety Monitor. In Zomba, it will be Dr Kabeya Biselele, a consultant Obstetrician with years of experiencing working in Malawi and other African countries managing pregnant women. He has also been a co-investigator and study physician on a large Pfizer-funded trial investigating the use of azithromycin-chloroquine in the prevention of malaria in

pregnancy. In Blantyre, the Safety Monitor will be Prof William Stones, a practising consultant obstetrician with experience working in resource-poor settings and also administering FCM in a Kenyan population. The safety monitors shall be available for consultation in an emergency but shall also oversee and advise on the clinical care SOPs and guidelines within the trial. They will also be part of the Trial Steering Committee and hence provide any clinic care-related advice to the trial executive decision-making body.

17.4.2. *Reporting by the sponsor*

The sponsor is responsible for reporting SAEs and other safety issues to the Data Monitoring Committee (DMC) and the Ethics committees in an expedited manner in accordance with the EU guidelines. The sponsor will also inform all other investigators concerned of relevant information about SAEs that could adversely affect the safety of subjects.

17.5. DATA MONITORING COMMITTEE

An independent Data Monitoring Committee (DMC) has been set up to review on a regular basis, safety and efficacy data of the ongoing trial. The sponsor will report all SAEs and other relevant safety information to the DMC on an expedited basis. The DMC will review tables of cumulative SAEs, primary and secondary endpoints in a blinded manner at regular intervals during the first six months of the trial and six monthly thereafter. The DMC will remain blinded when presented with any interim analysis results unless the DMC judges that for safety reason the study blind should be broken. In the advent of clear evidence of effectiveness or harm, the DMC will recommend to the sponsor and investigators whether to continue, modify, or terminate the trial on ethical grounds.

The DMC will comprise of international experts in clinical trials, obstetrics, epidemiology and statistics.

18. DATA HANDLING AND RECORD KEEPING

18.1. CASE REPORT FORMS (CRFS)

Data collected from the subjects will be recorded in digital - Open Data Kit (ODK) Case Report Forms (CRFs) using tablets. An Instruction manual on the completion of the CRF will be provided to investigators and relevant study site personnel. It will be the responsibility of the investigator to ensure that the data in the CRFs are accurate and complete. If any data are not available, omissions will be indicated on the CRFs. The CRF will automatically sign off the user and lock upon completion. Relevant hard copy patient hospital files will be scanned for reference and stored digitally, securely. All CRFs will be checked by the investigator or authorised personnel for accuracy and completeness before uploading to the server. All corrections will be done by the investigator or authorised personnel; corrections will be automatically saved with tracked changes. The data will then be uploaded on the server.

18.2. DATA ENTRY AND VALIDATION

Data will be entered as they become available into the MS access database using double data entry. Data verification and validation will be performed until the database corresponds with the data collected in the CRFs. Data query sheets will be raised and distributed by the data manager to the study team for resolution in a timely manner. Query resolutions will be stored together with the original CRFs.

18.3. DATABASE LOCK

When the validation process is concluded, the data will be declared and documented as clean. Once all decisions on the evaluability of the data from each individual subject have been made and documented, the treatment code will be broken and included in the database and the database will be locked.

19. STATISTICAL CONSIDERATIONS

19.1. SAMPLE SIZE

Sample size is calculated based on the maternal primary outcome of anaemia at 36 weeks' gestation ~~delivery~~. Haider *et al* identified a 50% reduction in anaemia prevalence in women receiving oral iron. In the proposed study, all women in the trial will have moderate to severe anaemia (Hb<10g/dL) at baseline. In a cohort of pregnant women in the Gambia, we observed that 60% of pregnant women with Hb<10g/dL at 20 weeks' gestation who then receive iron interventions continue to have Hb<10g/dL by 30 weeks. The Fer-ASAP trial demonstrated a 14% reduction in absolute anaemia prevalence compared with oral iron. We hypothesise that routine iron supplementation will reduce anaemia prevalence from 100% to 60% (as seen in the Gambia, and similar to data from Haider *et al*). We hypothesize that ferric carboxymaltose will result in a 10% absolute improvement in anaemia cure (to a prevalence of 50%), as discussed above. To show this with 80% power at a two-sided alpha level of 5%, and incorporating a 10% loss to follow-up, we expect to require a total sample size of 862 pregnant women or 431 per arm is needed (using Pearson's chi-square test). This sample size allows 86.5% power to detect the clinically meaningful effect size (100g) for birth weight. Finally, 80% power for anaemia and 86.5% power for birthweight means our trial will have 69% power to demonstrate both outcomes (if they are independent).

Participants who discontinue the study will not be replaced. Recruitment will be competitive between the sites.

19.2. DATA ANALYSIS

A detailed study statistical analysis plan for the final analysis will be drawn up during the course of the study before the unblinding of data at database lock.

19.2.1. *Assessment of effectiveness*

A detailed statistical analysis plan describing the proposed analyses will be finalised before the trial database is locked for final analysis. Analyses will be undertaken on an intention-

to-treat basis (maternal outcomes: all women randomized; neonatal outcomes: all live-borns with the exception of the stillbirth outcome). Descriptive statistics will be presented for all outcomes, by treatment groups across the follow-up time points. The primary maternal outcome anaemia ~~at pre-delivery (based on the capillary Hb closest prior to delivery)~~ will be analysed using a binomial log-linear regression, with the oral iron group as the reference, and incorporating clinic and treatment in the model. The primary maternal hypothesis will be evaluated by obtaining the estimate of the risk ratio of IV iron versus oral iron and a 95% confidence interval at 36 weeks' gestation. The secondary neonatal outcome birthweight will be analysed by fitting a linear regression model with a model specification similar to that of the primary maternal outcome. This hypothesis will be evaluated by obtaining the estimate of the absolute difference in birth weight between IV iron and oral iron and a 95% confidence interval. Other secondary binary outcomes will be analysed similarly as the primary maternal outcome and other secondary continuous outcomes as the birthweight outcome. ~~w~~Where applicable, models will ~~also~~ take~~ing~~ into account ~~in the model~~ the repeated data collection of the outcome.

19.2.2. *Analysis of adverse events*

Adverse reactions will be reported and tabulated for each treatment arm, overall and per body system and adverse event. Treatment-emergent adverse events are defined as adverse events that had an onset day on or after the day of the first dose of study medication. Adverse events that have missing onset dates will be considered to be treatment emergent.

19.2.3. *Health economics analyses*

An economic evaluation will be done to consider the cost-effectiveness of IV iron over the standard of care. The economic evaluation will estimate absolute and incremental health provider and patient costs as well as health benefits of the two study arms. The provider costs will include costs related to delivering the interventions and will be collected prospectively at the facilities included in the study. Patient cost data including indirect disease costs (productivity), will be collected as part of the routine trial CRFs. Effectiveness, compliance and health-related quality of life associated with intravenous iron versus standard of care will be based on primary trial data. A novel decision model will be developed to combine these data, supplemented by best available secondary data when required. This Markov model will take into account outcomes related to both the mothers and the children. Uncertainties related to single parameters will be captured using one-way sensitivity analyses, while overall uncertainty related to its predictions will be analysed using probabilistic sensitivity analyses. Finally, the model will be used to estimate in a threshold analysis a cost for the drug at which use of IV ferric carboxymaltose could be feasibly implemented in practice; this estimate will form the basis for future discussions with manufacturers and donors.

19.3. ANALYSIS POPULATIONS

Patients will be considered as 'completed the study' if they satisfied all study entry criteria, have received the treatment and have attended all visits till delivery as prescribed by the protocol and provided relevant biological samples as defined for these visits. A discontinuation is any subject who enters the study (i.e., gives informed consent), but does not complete the study. If a subject gives informed consent and then does not enter the study (i.e. is not allocated a Study Number) they will be classified as a screen failure.

The Intention-to-treat population is defined as all patients who have been randomised regardless of whether they completed the study.

19.4. MISSING DATA

Every effort will be made to minimise the amount of missing data in the trial. Whenever possible, information on the reason for missing data will be obtained. No adjustments will be made for missing data. Missing values in all outcomes will be reported across treatment groups and follow-up time points. Multiple imputations under the missing at random assumption will be performed as a secondary analysis for handling missing data in the primary maternal and neonate outcome; results will be compared with the primary ~~complete case~~ analysis to investigate the robustness of the findings to missing data.

19.5. INTERIM ANALYSES AND CRITERIA FOR TERMINATION OF THE TRIAL

Due to the short recruitment period of the trial, an interim analysis has not been planned. However, the sponsor reserves the right to temporarily suspend or prematurely discontinue this study at any time for reasons including, but are not limited to, safety or ethical issues or severe non-compliance. If the sponsor determines such action is needed, it will discuss this with the investigator. When feasible, the sponsor will provide advance notification to the investigator of the impending action prior to it taking effect. The sponsor will promptly inform all the Ethics committee and regulatory bodies and provide the reason for the suspension or termination.

20. STUDY MANAGEMENT

20.1. STUDY MONITORING

The Research Support Centre at the College of Medicine will perform independent monitoring of the study on behalf of the sponsor. Prior to subject enrolment, the monitor will visit the study site to determine the adequacy of facilities, review the protocol and data collection procedures and discuss the responsibilities of the investigator and other study site personnel.

During the study, the monitor will have regular site contacts, including conducting on-site visits to:

6. Confirm that the study is being performed according to the protocol, ICH GCP and applicable regulations, data are being accurately recorded in the CRFs,

samples are being appropriately collected and stored, and that investigational product accountability is being performed.

7. Conduct source data verification
8. Confirm facilities remain acceptable
9. Provide information and support to the investigators
10. Evaluate study progress

Upon completion of the study the monitor will visit the study site to verify that all CRFs are completed and collected, all data queries have been resolved and filed, conduct final accountability, reconciliation and arrangements for investigational product and verify all study site records are complete. The PI and relevant staff will be available at monitoring visits and agree to allocate sufficient time to the monitor to discuss any issues and address their resolution.

20.2. DIRECT ACCESS TO SOURCE DATA/DOCUMENTS

The investigator agrees to allow the sponsor and/or its representatives, including the monitor, the DMC, the IRB/IEC, the regulatory body direct access to source data and other relevant documents.

20.3. QUALITY ASSURANCE

Authorised representatives of the sponsor, an IEC/IRB or regulatory authority may visit the study site to perform audits or inspections, including source data verification.

20.4. TRAINING OF STAFF

The PI is responsible for the conduct of the study at this Study Site, including delegation of specified study responsibilities, and training of study staff. The PI will maintain a record of all individuals involved in the study (medical, nursing and other staff). The PI will ensure that all persons assisting with the trial receive the appropriate training about the protocol, the investigational product(s) and their trial-related duties and functions. During the study the regular spot checks will be conducted to assess the performance of study site staff members and re-training provided where necessary.

20.5. CHANGES TO THE PROTOCOL

No change will be made to the approved protocol without the agreement of the sponsor. If it is necessary for the protocol to be amended, the protocol amendment will be submitted to the IRB/IEC for approval before implementation. Any change to the informed consent form must also be approved by the sponsor and IRB/IEC, before the revised form is used. The sponsor will distribute amendments to the PI, who in turn is responsible for the distribution of these documents to the staff at his/her study site.

20.6. FINANCING AND INSURANCE

Funding for this study will be provided by Bill & Melinda Gates Foundation through a grant to the Walter and Eliza Hall Institute of Medical Research, Melbourne, Australia. The College

of Medicine will act as sponsor and the PI will purchase a liability insurance policy that covers this study.

20.7. STUDY DURATION

The study will start as soon as all pre-study activities and documentation are completed and the IRB/IEC and Regulatory Authority have approved the protocol. The total duration of the trial is 30 months, which includes including 12 months of recruitment, 6 month of additional follow-up phase as per Gantt chart below:

Table 9: Study time plan

ACTIVITY	2018										
	F	M	A	M	J	J	A	S	O	N	D
Site Preparation	■	■	■								
Participant recruitment				■	■	■	■	■	■	■	■
Participant Follow up											■
Data analysis											
Report writing											

ACTIVITY	2019											
	J	F	M	A	M	J	J	A	S	O	N	D
Site Preparation												
Participant recruitment	■	■	■	■	▨	▨	▨	▨	▨	▨	▨	▨
Participant Follow up	■	■	■	■	■	■	■	■	■	■	▨	▨
Data analysis											■	■
Report writing												

ACTIVITY	2020											
	J	F	M	A	M	J	J	A	S	O	N	D
Site Preparation												
Participant recruitment												
Participant Follow up	▨	▨	▨	▨	▨	▨	▨	▨	▨	▨	▨	▨
Data analysis	■											
Report writing		■	■	■								

ACTIVITY	2021											
	J	F	M	A	M	J	J	A	S	O	N	D
Site Preparation												
Participant recruitment												
Participant Follow up	▨	▨	▨	▨	▨							
Data analysis						▨	▨	▨	▨			
Report writing										▨	▨	▨

20.8. RECORD-KEEPING AND ARCHIVING

During the study, an Investigator Site File will be used to store documentation pertaining to the study and it will be kept in a secure location with access only to authorised individuals. It is the PI's responsibility to continuously update the Investigator file. It must be available to the Monitor during monitoring visits.

Following study closure, the investigator study file, CRFs, medical records and other source documents must be retained at the study site per regulatory obligations (two years after the last marketing application of the study drug in the European Union) and thereafter destroyed only after agreement with the sponsor. Documents with a limited shelf life (e.g. printouts on light/heat sensitive paper) will be copied and verified by signing and dating.

20.9. REPORTING AND PUBLICATION OF DATA

The results of the study will be submitted and discussed with the local and national medical authorities. They will then be presented at national and international conferences and submitted for publication in peer-reviewed journals, in accordance with the sponsor' publication policy.

21. ETHICAL CONSIDERATIONS

21.1. ETHICAL REVIEW

This study will be submitted to the following institutional review boards and independent ethics committees (IRB/IEC).

3. College of Medicine, Blantyre, Malawi
4. Walter and Eliza Hall Institute of Medical Research, Melbourne, Australia

Before initiating the trial, written and dated approval/favourable opinion must be obtained from the IRB/IECs. Protocol amendments will be submitted to the IRB/IEC for approval

before implementation. Progress reports, SUSAR reports and safety reports will be submitted to the IRB/IEC in accordance with local requirements.

21.2. ETHICAL CONDUCT OF THE STUDY

This study will be conducted in accordance with The International Conference on Harmonisation of Technical Requirements for Registration of Pharmaceuticals for Human Use (ICH) (ICH) guidelines for "good clinical practice" (GCP) and all applicable regulatory requirements, including, where applicable, the 1996 version of the Declaration of Helsinki.

21.3. INFORMED CONSENT

Informed consent will be obtained from each participant before conducting any study related procedure. The PI will ensure that that the participant (and the subject's parent/guardian in case of assent) is given full and adequate oral and written information about the study. Information will be provided in the local language of the participant. The participant should be given the opportunity to ask questions and allowed time to consider the information provided.

Informed consent will be documented by the use of a written consent form signed by the participant and the person who conducted the informed consent discussion. If the participant is unable to write their signature, then a thumbprint may be used. If the participant is unable to read the information her/himself, full and comprehensive information will be communicated to the participant in the presence of a witness. The witness will be an independent third party i.e. a person not connected with the conduct of the trial. The witness will sign the informed consent form to attest that the information in the consent form was accurately explained to and apparently understood by the participant and that informed consent was freely given. Each original signed informed consent will be kept on file by the investigator. A copy of the informed consent form will be provided to the participant.

Written informed consent obtained at the baseline visit will encompass all study procedures and visits up to and including the 12-month post-partum visit. We will re-discuss with mothers the extension study (mother and infant visits at 3, 6, 9 and 12-months post-partum) during the 1-month postpartum visit, although consent will have already been collected. We will also ask all mothers who would have signed the previous version of the informed consent form, before the extension was added, to sign a new consent form if they are happy to take part in the extension study.

21.4. RISKS TO STUDY PARTICIPANTS

All efforts will be made to minimise pain, discomfort, and fear for the children.

21.4.1. *Blood sampling*

The amount of blood taken for the study is minimal, totalling approximately up to 10 ml per visit over the study period at four time points during pregnancy and five time points post-partum (an additional 5 ml may be taken during sick visits for clinically-relevant laboratory

analyses) for mothers, and <3mL per visit at four time points for infants. Blood will be collected in most cases through a venepuncture and where indicated a finger prick. A small bruise or mild pain on the finger or the venepuncture site from where the blood is taken may develop. Only well trained and fully qualified laboratory staff will be hired for this project. Only new disposable needles and lancets are used for the blood taking procedures, and these will be discarded immediately after their use.

21.4.2. *Intravenous infusion*

Participants allocated to the intervention arm will receive an intravenous infusion. In this case, there will similarly undergo a venepuncture where a cannula will be inserted to allow the infusion of the iron over a period of 15 minutes. A small bruise or mild pain on the venepuncture site may develop. As discussed above, anaphylaxis is not a major risk of ferric carboxymaltose there will always be an emergency resuscitation tray during this procedure. A fully qualified clinician will be available during the entire infusion process (and for a further 45 min after) to monitor the condition of the participant. The cannula will not be removed until the trial participant is certified as well with normal blood pressure and heart rate prior to discharge home.

21.5. BENEFITS FROM PARTICIPATION

Participants enrolled in the study will receive close monitoring of their condition for the study period. A dedicated clinician will see subjects at recruitment and scheduled follow-up visits, for full medical assessments. If a subject has an illness in between scheduled visits, the subject will be seen by the study clinician and treated at the study clinic if the illness is deemed to be study related or referred to other clinics if other care is required.

21.6. SUBJECT DATA PROTECTION

Study data will be stored in computer database maintaining confidentiality in accordance with data legislation. The subject's names will not be recorded in the database but will be identified by subject number. Authorised members of the sponsor, clinical research organisation, DMC, IEC/IRB and regulatory authority may review the subjects' medical records. This is explained in the informed consent form

21.7. OTHER ETHICAL CONSIDERATIONS

21.7.1. *Reimbursement of costs*

The study will provide payment for all study drugs, study procedures, study related visits and reasonable medical expenses that may be incurred as a direct result of the study. This includes compensation for transport for each study visit. Where applicable the participant will be provided with or reimbursed for, refreshments when a scheduled appointment requires them to be out of their home at meal times. In Malawi, in-patient hospital care is free of charge.

22. DISSEMINATION OF RESULTS

At the end of the study, the results will first be disseminated to national policymakers, government departments, academics from local research institutions and universities, COMREC and professional bodies in Malawi at the national stakeholders' meeting or research dissemination conferences to be held in the country. Subject to the findings of the study and based on consensus emerging at these meetings, we will support national policymakers to develop the necessary tools and guidelines to guide national and district level health providers to implement the strategy within hospital services and the health system more broadly.

Research results will also be disseminated to the global research community, technical agencies, and international government bodies via peer-reviewed journals and at international scientific fora. We will also inform other international organisations and funders of large-scale anaemia control initiatives which aim to improve anaemia at regional and local levels and are instrumental in supporting countries to implement anaemia control policies in low and middle-income countries.

23. CAPACITY BUILDING

Research capacity in research in Malawi will be enhanced by the provision of training and mentorship for research staff. By running this study, capacity in trial management will be enhanced. The research study will strengthen the clinical skills of health workers in managing pregnant women with anaemia. There will be 1-2 PhD candidates who will conduct their research as part of this project. Partners from the different institutions forming this research network will jointly supervise them.

In addition, there will be a post-doctoral research scientist who will work closely with the study PIs in the conduct of the study. In the process, the post-doctoral research scientist will be able to carry out various research projects from this study that will be published in peer-reviewed journals. Lastly, all study staff will undertake Good Clinical Practice (GCP) training before starting to work on the research project.

24. BUDGET

REVAMP Study (Including the extend period)

**PERSONNEL**	**Year 1**	**Year 2**	**Year 3**	**Supp study budget (Y4)**	**Total**
Scientific Team					
Project Administration	122,796.00	126,480.00	87,633.00	16,523.85	353,432.85
Data Management	22,476.00	28,317.00	10,464.00		61,257.00
Clinical Personnel	143,154.00	174,220.00	10,357.00	62,203.68	389,934.68
Laboratory Personnel	28,275.00	38,415.00	9,817.00		76,507.00
Research Assistants - supplementary				18,750.00	18,750.00
Cognitive Testers - supplementary				24,970.68	24,970.68
**Total Personnel**	**316,701.00**	**367,432.00**	**118,271.00**	**122,448.21**	**924,852.21**
**EQUIPMENT**					
Vehicle 4X4	108,000.00				108,000.00
Motor-bike	11,000.00				11,000.00
Desk-top computer	7,200.00				7,200.00
Laptops	10,400.00				10,400.00
Printer & accessories	2,200.00				2,200.00
HemoCue machine	3,300.00				3,300.00
Ultrasound machine	50,000.00				50,000.00
Small clinical equipment	9,000.00		9,000.00		18,000.00
Small laboratory equipment	10,000.00		10,000.00		20,000.00
**Total Equipment**	**211,100.00**	**0.00**	**19,000.00**	**0.00**	**230,100.00**
**CONSUMABLES**					
Lab assays	6,720.00	20,160.00	40,320.00	10,125.00	77,325.00

Lab consumables	2,040.00	6,800.00	4,760.00	3,375.00	16,975.00
RDT cuvettes	2,000.00	6,000.00	4,000.00	10,125.00	22,125.00
FBC reagents	5,520.00	5,520.00	5,520.00	4,140.00	20,700.00
FBC machine maintenance	4,000.00	4,000.00	4,000.00	3,375.00	15,375.00
Fuel-bikes & Cars	8,280.00	11,040.00	5,520.00	7,125.00	31,965.00
Maintenance & Insurance	10,400.00	10,400.00	10,400.00	7,800.00	39,000.00
Participants Cost	5,120.00	6,400.00	1,280.00	8,250.00	21,050.00
Communication & Internet	6,456.00	6,456.00	6,456.00	4,875.00	24,243.00
Stationery	6,720.00	6,720.00	3,360.00	5,250.00	22,050.00
Office refurbishments/Rentals	8,000.00	1,000.00	1,000.00	3,750.00	13,750.00
Study Monitoring	4,000.00	10,000.00	6,000.00	4,500.00	24,500.00
Trial Insurance	50,000.00			9,375.00	59,375.00
Ethics Submission fee-Malawi	150				150.00
Regulatory Submission fee-Malawi	6,000.00				6,000.00
Regulatory annual submission fees		1,000.00	1,000.00	750	2,750.00
Short Courses e.g GCP	5,400.00	1,350.00	1,350.00	1,762.50	9,862.50
Dissemination of results				1,875.00	1,875.00
Local Publication				2,250.00	2,250.00
Annual team meetings	1,300.00	1,300.00	1,300.00	1,125.00	5,025.00
**Total Consumables**	**132,106.00**	**98,146.00**	**96,266.00**	**89,827.50**	**416,345.50**
**Sub-Total**	**659,907.00**	**465,578.00**	**233,537.00**	**212,275.71**	**1,571,297.71**
Overheads (10%)	65,990.70	46,557.80	23,353.70	21,227.57	157,129.77
**GRAND TOTAL ($)**	**725,897.70**	**512,135.80**	**256,890.70**	**233,503.28**	**1,728,427.48**

25. REFERENCES

1. World Health Organization, *The global prevalence of anaemia in 2011*. 2015, Geneva: World Health Organization.
2. Haider, B.A., et al., *Anaemia, prenatal iron use, and risk of adverse pregnancy outcomes: systematic review and meta-analysis.* BMJ, 2013. **346**: p. f3443.
3. WHO/UNICEF/UNU, *Iron Deficiency Anaemia: Assessment, Prevention, and Control. A guide for programme managers*. 2001, Geneva: World Health Organization.
4. Nair, M., M. Knight, and J.J. Kurinczuk, *Risk factors and newborn outcomes associated with maternal deaths in the UK from 2009 to 2013: a national case-control study.* BJOG, 2016. **123**(10): p. 1654-62.
5. Drassinower, D., et al., *The effect of maternal haematocrit on offspring IQ at 4 and 7 years of age: a secondary analysis.* BJOG, 2016.
6. World Health Organization, *Global Nutrition Targets 2025: Anaemia Policy Brief*. 2014, World Health Organization: Geneva.
7. Mwangi, M.N., et al., *Effect of Daily Antenatal Iron Supplementation on Plasmodium Infection in Kenyan Women: A Randomized Clinical Trial.* JAMA, 2015. **314**(10): p. 1009-20.
8. Pena-Rosas, J.P., et al., *Daily oral iron supplementation during pregnancy.* Cochrane Database Syst Rev, 2015(7): p. CD004736.
9. WHO, *Essential Nutrition Actions: Improving maternal, newborn, infant and young child health and nutrition*. 2013, Geneva: World Health Organization.
10. World Health Organization, *Iron and folate supplementation*. Integrated management of pregnancy and childbirth (IMPAC). 2006, Geneva: World Health Organization.
11. Low, M.S., et al., *Daily iron supplementation for improving anaemia, iron status and health in menstruating women.* Cochrane Database Syst Rev, 2016. **4**: p. CD009747.
12. Bah, A., et al., *Serum Hepcidin Concentrations Decline during Pregnancy and May Identify Iron Deficiency: Analysis of a Longitudinal Pregnancy Cohort in The Gambia.* J Nutr, 2017. **147**(6): p. 1131-1137.
13. Lyseng-Williamson, K.A. and G.M. Keating, *Ferric carboxymaltose: a review of its use in iron-deficiency anaemia.* Drugs, 2009. **69**(6): p. 739-56.
14. Friedrisch, J.R. and R.D. Cancado, *Intravenous ferric carboxymaltose for the treatment of iron deficiency anemia.* Rev Bras Hematol Hemoter, 2015. **37**(6): p. 400-5.
15. Rognoni, C., et al., *Efficacy and Safety of Ferric Carboxymaltose and Other Formulations in Iron-Deficient Patients: A Systematic Review and Network Meta-analysis of Randomised Controlled Trials.* Clin Drug Investig, 2016. **36**(3): p. 177-94.
16. Moore, R.A., et al., *Meta-analysis of efficacy and safety of intravenous ferric carboxymaltose (Ferinject) from clinical trial reports and published trial data.* BMC blood disorders, 2011. **11**(1): p. 4.
17. Breymann, C., et al., *Ferric carboxymaltose vs. oral iron in the treatment of pregnant women with iron deficiency anemia: an international, open-label, randomized controlled trial (FER-ASAP).* J Perinat Med, 2017. **45**(4): p. 443-453.
18. Van Wyck, D.B., et al., *Intravenous ferric carboxymaltose compared with oral iron in the treatment of postpartum anemia: a randomized controlled trial.* Obstet Gynecol, 2007. **110**(2 Pt 1): p. 267-78.

19. Cortes, X., et al., *Safety of ferric carboxymaltose immediately after infliximab administration, in a single session, in inflammatory bowel disease patients with iron deficiency: a pilot study.* PLoS One, 2015. **10**(5): p. e0128156.
20. Laass, M.W., et al., *Effectiveness and safety of ferric carboxymaltose treatment in children and adolescents with inflammatory bowel disease and other gastrointestinal diseases.* BMC Gastroenterol, 2014. **14**: p. 184.
21. Evstatiev, R., et al., *Ferric carboxymaltose prevents recurrence of anemia in patients with inflammatory bowel disease.* Clin Gastroenterol Hepatol, 2013. **11**(3): p. 269-77.
22. Befrits, R., et al., *Anemia and iron deficiency in inflammatory bowel disease: an open, prospective, observational study on diagnosis, treatment with ferric carboxymaltose and quality of life.* Scand J Gastroenterol, 2013. **48**(9): p. 1027-32.
23. Beigel, F., et al., *Iron status and analysis of efficacy and safety of ferric carboxymaltose treatment in patients with inflammatory bowel disease.* Digestion, 2012. **85**(1): p. 47-54.
24. Evstatiev, R., et al., *FERGIcor, a randomized controlled trial on ferric carboxymaltose for iron deficiency anemia in inflammatory bowel disease.* Gastroenterology, 2011. **141**(3): p. 846-853 e1-2.
25. Kulnigg, S., et al., *A novel intravenous iron formulation for treatment of anemia in inflammatory bowel disease: the ferric carboxymaltose (FERINJECT) randomized controlled trial.* Am J Gastroenterol, 2008. **103**(5): p. 1182-92.
26. Ponikowski, P., et al., *The impact of intravenous ferric carboxymaltose on renal function: an analysis of the FAIR-HF study.* Eur J Heart Fail, 2015. **17**(3): p. 329-39.
27. Szczech, L.A., et al., *Randomized evaluation of efficacy and safety of ferric carboxymaltose in patients with iron deficiency anaemia and impaired renal function (REPAIR-IDA): rationale and study design.* Nephrol Dial Transplant, 2010. **25**(7): p. 2368-75.
28. Anker, S.D., et al., *Ferric carboxymaltose in patients with heart failure and iron deficiency.* N Engl J Med, 2009. **361**(25): p. 2436-48.
29. Khalafallah, A.A., et al., *Intravenous ferric carboxymaltose versus standard care in the management of postoperative anaemia: a prospective, open-label, randomised controlled trial.* Lancet Haematol, 2016. **3**(9): p. e415-25.
30. Nores, J.A., *The Efficacy of IV Ferric Carboxymaltose in the Perioperative Management of Moderate to Severe Iron Deficiency Anemia.* J Minim Invasive Gynecol, 2015. **22**(6S): p. S211-S212.
31. Borstlap, W.A.A., et al., *Multicentre randomized controlled trial comparing ferric(III)carboxymaltose infusion with oral iron supplementation in the treatment of preoperative anaemia in colorectal cancer patients.* BMC Surg, 2015. **15**: p. 78.
32. *A multicentre comparative study on the efficacy of intravenous ferric carboxymaltose and iron sucrose for correcting preoperative anaemia in patients undergoing major elective surgery British Journal of Anaesthesia, 2015; 107(3): 477-78, DOI 10.1093/bja/aer242.* Br J Anaesth, 2015. **115**(1): p. 154.
33. Bernabeu-Wittel, M., et al., *Ferric carboxymaltose with or without erythropoietin for the prevention of red-cell transfusions in the perioperative period of osteoporotic hip fractures: a randomized contolled trial. The PAHFRAC-01 project.* BMC Musculoskelet Disord, 2012. **13**: p. 27.

34. Bisbe, E., et al., *A multicentre comparative study on the efficacy of intravenous ferric carboxymaltose and iron sucrose for correcting preoperative anaemia in patients undergoing major elective surgery.* Br J Anaesth, 2011. **107**(3): p. 477-8.
35. Froessler, B., et al., *Intravenous ferric carboxymaltose for anaemia in pregnancy.* BMC Pregnancy Childbirth, 2014. **14**: p. 115.
36. D, Z., et al., *Intravenous Iron Treatment in Pregnancy: Ferric Carboxymaltose for Correction of Iron Deficiency Anaemia.* Faridpur Med. Coll. J., 2017. **12**(2): p. 54-57.
37. Mishra, V., et al., *Role of Intravenous Ferric Carboxy-maltose in Pregnant Women with Iron Deficiency Anaemia.* J Nepal Health Res Counc, 2017. **15**(2): p. 96-99.
38. Christoph, P., et al., *Intravenous iron treatment in pregnancy: comparison of high-dose ferric carboxymaltose vs. iron sucrose.* J Perinat Med, 2012. **40**(5): p. 469-74.
39. Pavord, S., et al., *UK guidelines on the management of iron deficiency in pregnancy.* Br J Haematol, 2012. **156**(5): p. 588-600.
40. Rao, A., et al., *FERINJECT IN PREGNANCY AND THE POSTPARTUM PERIOD FOR TREATING IRON DEFICIENCY ANAEMIA*. 2015, Mid Essex Hospital Services NHS Trust.
41. Khalafallah, A.A. and A.E. Dennis, *Iron deficiency anaemia in pregnancy and postpartum: pathophysiology and effect of oral versus intravenous iron therapy.* J Pregnancy, 2012. **2012**: p. 630519.
42. Achebe, M.M. and A. Gafter-Gvili, *How I treat anemia in pregnancy: iron, cobalamin, and folate.* Blood, 2017. **129**(8): p. 940-949.
43. http://www.masimo.com/siteassets/us/documents/pdf/plm-11187a-product-information-spot-check-sphb-us.pdf
44. Daru, J., N.A. Cooper, and K.S. Khan, *Systematic review of randomized trials of the effect of iron supplementation on iron stores and oxygen carrying capacity in pregnancy.* Acta Obstet Gynecol Scand, 2016. **95**(3): p. 270-9.
45. Favrat, B., et al., *Evaluation of a single dose of ferric carboxymaltose in fatigued, iron-deficient women--PREFER a randomized, placebo-controlled study.* PLoS One, 2014. **9**(4): p. e94217.
46. Bailie, G.R., N.A. Mason, and T.G. Valaoras, *Safety and tolerability of intravenous ferric carboxymaltose in patients with iron deficiency anemia.* Hemodial Int, 2010. **14**(1): p. 47-54.
47. Breymann, C., et al., *Ferric carboxymaltose vs. oral iron in the treatment of pregnant women with iron deficiency anemia: an international, open-label, randomized controlled trial (FER-ASAP).* J Perinat Med, 2016.

48. Kimani, J., et al., *Efficacy and Safety of Azithromycin-Chloroquine versus Sulfadoxine-Pyrimethamine for Intermittent Preventive Treatment of Plasmodium falciparum Malaria Infection in Pregnant Women in Africa: An Open-Label, Randomized Trial.* PLoS One, 2016. **11**(6): p. e0157045.
49. Bothwell, T.H., *Iron requirements in pregnancy and strategies to meet them.* Am J Clin Nutr, 2000. **72**(1 Suppl): p. 257S-264S.
50. Depression Anxiety Stress Scale. Available from: https://maic.qld.gov.au/wp-content/uploads/2016/07/DASS-21.pdf
51. Perrelli J, Zambaldi C, Cantilino A, Sougey E. Mother-child bonding assessment tools: Review. Elsevier. 2014;32(3):257–65.

52. WHO. Indicators for assessing infant and young child feeding practices: part 1: definitions [Internet]. Geneva (Switzerland): WHO; 2008. Available from: http://apps.who.int/iris/bitstream/10665/43895/1/9789241596664_eng.pdf
53. Hamadani JD, Tofail F, Hilaly A, Huda SN, Engle P, Grantham- McGregor SM. Use of family care indicators and their relationship with child development in Bangladesh. J Health Popul Nutr 2010; 28:23–33.
54. Https://www.fantaproject.org/sites/default/files/resources/HFIAS_ENG_v3_Aug07.pdf
55. Graham, S.M., et al., *Clinical presentation of non-typhoidal Salmonella bacteraemia in Malawian children.* Trans R Soc Trop Med Hyg, 2000. **94**(3): p. 310-4.
56. *Rose SA, Feldman JF, Wallace IF, McCarton C. Information processing at 1 year: Relation to birth status and developmental outcome during the first 5 years. Developmental Psychology 1991; 27:723.*
57. *Bornstein MH, Sigman MD. Continuity in mental development from infancy. Child development 1986; 57:251-74.*
58. *deRegnier RA, Nelson CA, Thomas KM, Wewerka S, Georgieff MK. Neurophysiologic evaluation of auditory recognition memory in healthy newborn infants and infants of diabetic mothers. The Journal of pediatrics 2000; 137:777-84.*
59. *Nuwer MR, Comi G, Emerson R, et al. IFCN standards for digital recording of clinical EEG. Clinical Neurophysiology 1998; 106:259-61.*
60. *Monif M, Seneviratne U. Clinical factors associated with the yield of routine outpatient scalp electroencephalograms: A retrospective analysis from a tertiary hospital. Journal of Clinical Neuroscience 2017; 45:110-4.*
61. *Burden MJ, Westerlund AJ, Armony-Sivan R, et al. An event-related potential study of attention and recognition memory in infants with iron-deficiency anemia. Pediatrics 2007;120: e336-e45.*
62. *Monga M, Walia V, Gandhi A, Chandra J, Sharma S. Effect of iron deficiency anemia on visual evoked potential of growing children. Brain & Development 2010; 32:213-6.*
63. *Geng F, Mai X, Zhan J, et al. Impact of Fetal-Neonatal Iron Deficiency on Recognition Memory at 2 Months of Age. The Journal of pediatrics 2015; 167:1226-32.*
64. *Algarin C, Nelson CA, Peirano P, Westerlund A, Reyes S, Lozoff B. Iron-deficiency anemia in infancy and poorer cognitive inhibitory control at age 10 years. Developmental medicine and child neurology 2013; 55:453-8.*
65. *Algarin C, Peirano P, Garrido M, Pizarro F, Lozoff B. Iron deficiency anemia in infancy: long-lasting effects on auditory and visual system functioning. Pediatric research 2003; 53:217-23.*
66. European Commission*. Detailed guidance on the collection, verification and presentation of adverse reaction reports arising from clinical trials on medicinal products for human use, Revision 1, ENTR/CT* 3.http://eudract.emea.eu.int/docs/Detailed%20guidance%20collection%20of%20adverse%20events.pdf.
67. Single market: management & legislation for consumer goods. Pharmaceuticals : regulatory framework and market authorisations 2004; Available from: http://eudract.emea.eu.int/docs/Detailed%20guidance%20collection%20of%20adverse%20events.pdf
68. Bager, P. and J.F. Dahlerup, *Randomised clinical trial: oral vs. intravenous iron after upper gastrointestinal haemorrhage--a placebo-controlled study.* Aliment Pharmacol Ther, 2014. **39**(2): p. 176-87.

69. Breymann, C., et al., *Comparative efficacy and safety of intravenous ferric carboxymaltose in the treatment of postpartum iron deficiency anemia.* Int J Gynaecol Obstet, 2008. **101**(1): p. 67-73.
70. Onken, J.E., et al., *Ferric carboxymaltose in patients with iron-deficiency anemia and impaired renal function: the REPAIR-IDA trial.* Nephrol Dial Transplant, 2014. **29**(4): p. 833-42.
71. Onken, J.E., et al., *A multicenter, randomized, active-controlled study to investigate the efficacy and safety of intravenous ferric carboxymaltose in patients with iron deficiency anemia.* Transfusion, 2014. **54**(2): p. 306-15.
72. Qunibi, W.Y., et al., *A randomized controlled trial comparing intravenous ferric carboxymaltose with oral iron for treatment of iron deficiency anaemia of non-dialysis-dependent chronic kidney disease patients.* Nephrol Dial Transplant, 2011. **26**(5): p. 1599-607.
73. Schatz, U., et al., *Iron deficiency and its management in patients undergoing lipoprotein apheresis. Comparison of two parenteral iron formulations.* Atheroscler Suppl, 2013. **14**(1): p. 115-22.
74. Seid, M.H., et al., *Ferric carboxymaltose injection in the treatment of postpartum iron deficiency anemia: a randomized controlled clinical trial.* Am J Obstet Gynecol, 2008. **199**(4): p. 435 e1-7.
75. Van Wyck, D.B., et al., *Large-dose intravenous ferric carboxymaltose injection for iron deficiency anemia in heavy uterine bleeding: a randomized, controlled trial.* Transfusion, 2009. **49**(12): p. 2719-28.

26. APPENDIX A - Safety and Efficacy of FCM from randomised controlled trials

Safety and Efficacy of FCM from randomised controlled trials – from Rognoni et al [15]

Study (year)	n	Condition	Inclusion Criteria	Intervention arm	Control arm	Efficacy	Safety
**Bager et al. (2014) [68]**	**97**	**Non-variceal acute upper gastrointestinal bleeding**	**Hb <12 g/dl for women and <13 g/dl for men**	**Intravenous ferric carboxymaltose (1000 mg; single infusion)**	**Oral ferrous sulphate (100 mg; twice daily; 3 months) or intravenous placebo (saline)**	**At week 13, mean Hb (g/dl) in ferric carboxymaltose group was 13.9 (13.4–14.3) vs. 13.5 (12.9–14.1) in oral iron and 11.5 (10.3–12.9) in placebo (*p* < 0.01)**	**Re-admission for bleeding was reported in 2.4 % of patients in both the oral and intravenous groups (p = NS). Admission due to cardiovascular symptoms was reported in 4.9, 4.8 and 7.1 % of oral, intravenous and placebo groups, respectively (p = NS)**
**Bailie et al. (2010) [44]**	**559**	**Iron deficiency anaemia (various conditions)**	**Hb <12 g/dl; TSAT ≤25 %; serum ferritin <300 ng/ml (CKD and IBD patients) or ≤100 ng/ml (other)**	**Intravenous ferric carboxymaltose (1000 mg; single infusion)**	**Intravenous placebo (normal saline, 100–250 ml)**	**NR**	**During the first 24 h of the treatment, at least one TEAE was experienced by 15.0 % of subjects after receiving ferric carboxymaltose and 11.4 % after receiving placebo (*p* = 0.066). During the 7-day treatment period, at least one TEAE was experienced by 29.3 % of the subjects after receiving ferric carboxymaltose and 19.7 % after receiving placebo (*p* < 0.001). The most common events in the ferric carboxymaltose group were headache (5.4 %), nausea (3.8 %) and dizziness (1.8 %)**
**Breymann et al. (2008) [69]**	**349**	**Post-partum anaemia**	**Hb ≤10.5 g/dl**	**Intravenous ferric carboxymaltose (1000 mg; up to 3 weekly**	**Oral ferrous sulphate (100 mg; twice per day; 12 weeks')**	**At week 12, Hb levels increased to a mean of 130.4 g/l in ferric carboxymaltose vs. 128.9 g /l in ferrous sulphate (*p* = NS). Mean ferritin was 161.2 µg/l vs.**	**Overall, AEs were experienced by 26.0 % in ferric carboxymaltose vs. 22.2 % in ferrous sulphate (*p* = 0.51). Statistically significant differences (ferric carboxymaltose vs. ferrous sulphate) were seen in gastrointestinal disorders (3.5 vs. 10.3 %; *p* = 0.015), general disorders and administration site conditions (6.2 vs.0.0 %; *p* = 0.003), and musculoskeletal and**

				doses)		43.3 μg/l ($p < 0.0001$)	connective tissue disorders (0.0 vs. 2.6 %; $p = 0.039$). Constipation was less common in ferric carboxymaltose group (0.4 vs. 6.8 %). Two patients in ferric carboxymaltose group and none in the control group experienced severe AEs (i.e. hypersensitivity). There were no cases of anaphylactic shock/reaction
Evstatiev et al. (2013) [21]	256	Inflammatory bowel disease	Hb ≥12 g/dl in women and ≥13 g/dl in men	Intravenous ferric carboxymaltose (500 mg; single infusion)	Placebo (saline solution; 500 mg)	Anaemia recurred in 26.7 % of patients given ferric carboxymaltose vs. 39.4 % given placebo. Time to anaemia recurrence was longer in the ferric carboxymaltose group ($p = 0.049$). Ferritin and TSAT increased by 30.3 μg/l and 0.6 %, respectively, in ferric carboxymaltose group; the same parameters decreased by 36.1 μg/l and 4.0 %, respectively, in placebo group	AEs were reported in 59.0 % in ferric carboxymaltose group vs. 50.5 % of placebo group, and serious AEs in 6.7 and 8.1 %, respectively. The most common AEs were ulcerative colitis-specific symptoms and nasopharyngitis (7.8 vs. 7.3 %). Gastrointestinal symptoms and flares of ulcerative colitis were somewhat less frequent with ferric carboxymaltose compared with placebo: 20.0 vs 28.3 % ($p = 0.17$) and 6.7 vs. 12.1 % ($p = 0.18$), respectively. No anaphylactic reaction was reported and no death occurred
Evstatiev et al. (2011) [24]	85	Inflammatory bowel disease	Hb = 7–12 g/dl (women) and 7–13 g/dl (men)	Intravenous ferric carboxymaltose (500 or 1000 mg; up to 3 infusions)	Intravenous iron sucrose (200 mg; up to 11 infusions, twice weekly)	More patients with ferric carboxymaltose than iron sucrose achieved Hb response (65.8 vs. 53.6 %; $p = 0.004$) or Hb normalisation (72.8 vs. 61.8 %; $p = 0.015$)	The frequency of treatment-related AEs was comparable between the two groups ($p = 0.413$). One treatment-related serious AE (i.e. pulmonary embolism) was reported in ferric carboxymaltose group. Skin and subcutaneous tissue disorders such as rash, dermatitis and pruritus were reported in 3.7 % of ferric carboxymaltose patients and 0.8 % of the iron sucrose group ($p = 0.063$). No true hypersensitivity reactions were reported
Favrat et al. (2014) [45]	94	Fatigue in premenopausal, non-	Serum ferritin <50 μg/l and	Intravenous ferric carboxymalt	Intravenous placebo (saline,	Fatigue was reduced in 65.3 % (ferric carboxymaltose) and 52.7	TEAEs were experienced by 57.2 % of ferric carboxymaltose and 49.0 % of placebo-treated patients ($p = 0.16$). Five ferric carboxymaltose -

		anaemic women	TSAT <20 %; Hb ≥115 g/l. Piper Fatigue Scale (PFS) score ≥5	ose (1000 mg; single infusion)	1000 mg)	% (placebo) of patients (OR = 1.68; *p*= 0.03). All ferric carboxymaltose -treated patients (vs. 86 % in placebo group) had haemoglobin levels ≥120 g/l	treated patients reported severe AEs (in four, the events were considered drug-related)
Kulnigg et al. (2008) [25]	200	Inflammatory bowel disease	Hb ≤10 g/dl; TSAT <20 % or serum ferritin <100 μg/l	Intravenous ferric carboxymaltose (maximum 1000 mg repeated weekly until deficit was corrected)	Oral ferrous sulphate (100 mg; twice daily; 12 weeks')	The median Hb improved from 8.7 to 12.3 g/dl (ferric carboxymaltose) vs. from 9.1 to 12.1 g/dl (ferrous sulphate, *p* = 0.70). Median ferritin increased from 5.0 to 43.5 μg/l (ferric carboxymaltose) vs. from 6.5 to 28.5 μg/l (ferrous sulphate)	Treatment-related AEs occurred in 28.5 % (ferric carboxymaltose) and 22.2 % (ferrous sulphate) of patients, respectively. Most commonly reported (>2 % of patients overall) treatment-related AEs for ferric carboxymaltose and ferrous sulphate respectively were abdominal pain (2.9 vs. 3.2 %), nausea (2.2 vs. 4.8 %), headache (2.9 vs. 1.6 %) and diarrhoea (0.7 vs. 6.3 %)
Onken et al. (2014) [70]	2584	Non-dialysis-dependent CKD	Hb ≤11.5 g/dl; glomerular filtration rate (GFR) <60 ml/min/1.73 m^2	Intravenous ferric carboxymaltose (750 mg; twice weekly)	Intravenous iron sucrose (200 mg; five infusions in 14 days)	The mean Hb increase was 1.13 g/dl (ferric carboxymaltose) vs. 0.92 g/dl (iron sucrose; 95 % CI 0.13–0.28). More patients in the ferric carboxymaltose group achieved Hb increase ≥1.0 g/dl (48.6 vs. 41.0 %; 95 % CI 3.6–11.6)	During the study, at least one drug-related TEAE occurred in 298 of 1276 (23.4 %) subjects in ferric carboxymaltose group and 202 of 1285 (15.7 %) subjects in the iron sucrose group. The most common events were nausea (8.6 % in ferric carboxymaltose group vs. 1.6 % in iron sucrose), hypertension (4.6 vs. 2.0 %), flushing (3.0 vs. 0.1 %), dizziness (2.4 vs. 1.2 %) and dysgeusia (2.4 vs. 1.2 %). The majority of drug-related TEAEs were mild or moderate in severity. At least one serious AE was experienced by 202 of 1276 (15.8 %) participants receiving ferric carboxymaltose and 197 of 1285 (15.3 %) participants receiving iron sucrose (*p* = 0.74), with congestive heart failure being the most commonly reported (2.4 vs. 2.3 %; *p* = 0.90)
Onken et al. (2014) [71]	507 (A vs.	Iron deficiency	Hb ≤11 g/dl; ferritin	(A) or (C) intravenous	(B) Oral ferrous	Mean (±SD) Hb was significantly higher in	During the treatment phase, at least one drug-related TEAE was experienced by 22.8 % of

	B)<br><br>504 (C vs. D)	anaemia (various conditions)	<100 ng/ml (or <300 ng/ml when TSAT <30 %)	ferric carboxymaltose (750 mg; twice weekly)	sulphate (325 mg; three times a day; 14 days); (D) intravenous iron standard-of-care (IVSC)	group A than in group B: 1.57 (±1.19) vs. 0.80 (±0.80) g/dl (*p* = 0.001). Group C vs. group D: Hb = 2.90 (±1.64) vs. 2.16 (±1.25) g/dl (*p* = 0.001)	participants in Group A, 6.3 % in Group B, 25.3 % in Group C, and 26.5 % in Group D. The most common events were hypophosphatemia (Group C 5.5 %; Group A 3.7 %), nausea (Group A 4.1 %; Group D 3.3 %), protocol-defined hypotension (Group D 3.7 %), and constipation (Group B 3.2 %). Serious events were described in eight (3.3 %) patients in group A, 10 (4.0 %) in group B, 17 (6.7 %) in group C, and 16 (6.5 %) in group D. Hypersensitivity events were reported in eight participants: three (0.8 %) were in group A, two (0.8 %) were in group C and six (2.4 %) in group D
Quinibi et al. (2011) [72]	55	Non-dialysis-dependent CKD	Hb ≤11 g/dl; TSAT ≤25 %; serum ferritin ≤300 ng/ml; glomerular filtration rates ≤45 ml/min/1.73 m^2	Intravenous ferric carboxymaltose (1000 mg; up to two additional doses at two-week intervals)	Oral ferrous sulphate (325 mg; 3 times daily; 56 days)	Mean increase in Hb was 0.95 ± 1.12 (ferric carboxymaltose) vs. 0.50 ± 1.23 g/dl (ferrous sulphate; *p* = 0.005); mean increase in ferritin was 432 ± 189 vs. 18 ±45 ng/ml (*p* < 0.001); mean increase in TSAT was 13.6 ± 11.9 % vs. 6.1 ± 8.1 % (*p* < 0.001)	The proportion of subjects who experienced at least one possibly drug-related AE was significantly lower in ferric carboxymaltose group compared with the oral iron group: 2.7 % in ferric carboxymaltose group and 26.2 % in the oral iron group (*p* = 0.0001). The most commonly AEs the ferric carboxymaltose group were peripheral oedema (6.1 %), hyperkalaemia (4.1 %), urinary tract infection (3.4 %), hypotension (3.4 %), bronchitis, headache and infusion site reaction (2.0 % each). The most commonly experienced AEs in the oral iron group were constipation (17.5 %), nausea (4.9 %), diarrhoea, upper respiratory tract infection (3.9 % each), discoloured faeces and gastrointestinal haemorrhage (2.9 % each). Serious AEs were recorded in 13 (8.8 %) subjects in ferric carboxymaltose group and ten (9.7 %) in the oral iron group
Schatz et al. (2013) [73]	50	Apheresis-related anaemia	Serum ferritin <100 μg/l; or ferritin <300 μg/l;	Intravenous ferric carboxymaltose (500–1000 mg;	Intravenous ferric gluconate (62.5 mg; once weekly	Serum ferritin (mean) at week 8: 117.9 μg/l in ferric gluconate vs. 140.2 μg/l in ferric carboxymaltose (*p* = 0.4). TSAT: 22 %	There were no serious AEs in either group, especially no anaphylactic shocks. Six patients per group showed AEs, mainly gastrointestinal distress and flu-like symptoms. All AEs were mild, transient and self-limiting. There were no injection site

			TSAT <20 %	single infusion)	until deficit was corrected)	(ferric gluconate) vs. 26.4 % (ferric carboxymaltose; $p = 0.02$)	reactions in either group
Seid et al. (2008) [74]	89	Post-partum anaemia	Hb ≤10 g/dl	Intravenous ferric carboxymaltose (1000 mg; weekly for a maximum of 2500 mg)	Oral ferrous sulphate (325 mg; 3 times daily; 6 weeks')	Ferric carboxymaltose - treated patients were significantly more likely to achieve Hb >12 g/dl (91 vs. 67 %; $p < 0.0001$) in a shorter time period (14 vs. 27 days; $p < 0.0002$) and attain higher TSAT (36 vs. 28 %) and ferritin (647 vs. 12 ng/ml) levels ($p < 0.0001$)	Patients reporting one or more drug-related AEs were lower in ferric carboxymaltose (10.6 %) than in oral ferrous sulphate (21.8 %) group. Urticaria (2 %) was the only AE occurring in ≥2 % of the ferric carboxymaltose -treated patients. Four (2.8 %) subjects in ferric carboxymaltose group and four (2.7 %) subjects in the oral iron group experienced at least one serious AE, none of which was considered to be related to study medication or led to premature discontinuation
Van Wyck et al. (2009) [75]	477	Uterine bleeding	Hb ≤11 g/dl; serum ferritin ≤100 ng/ml; TSAT ≤25 %	Intravenous ferric carboxymaltose (maximum 1000 mg repeated weekly to achieve a total replacement dose)	Oral ferrous sulphate (325 mg; 3 times daily; 6 weeks')	Compared to ferrous sulphate, more patients assigned to ferric carboxymaltose achieved correction (Hb ≥12 g/dl) of anaemia (73 % vs. 50 %; $p < 0.001$) and experienced greater improvement in symptoms of fatigue ($p < 0.05$)	No hypotensive or serious drug-related AEs were reported in either treatment group. There were no deaths. Patients assigned to oral iron therapy were more likely to experience drug-related gastrointestinal complaints, particularly constipation (14.2 vs. 3.0 %), diarrhoea (4.4 vs. 1.7 %), nausea (11.9 vs. 3.5 %), and vomiting (3.1 vs. 0.4 %). Patients assigned to intravenous iron therapy were more likely to report transient fatigue (2.2 vs. 0 %), headache (6.5 vs. 4.4 %), dizziness (2.2 vs. 0.4 %), dysgeusia (2.6 vs. 0.9 %), and rash (2.2 vs. 0 %)
Van Wyck et al. (2007) [18]	52	Post-partum anaemia	Hb ≤10 g/dl	Intravenous ferric carboxymaltose (maximum 1000 mg repeated weekly to achieve a total replacement	Oral ferrous sulphate (325 mg; 3 times daily; 6 weeks')	Patients assigned to intravenous ferric carboxymaltose compared with those assigned to oral iron were more likely to achieve Hb >12 g/dl (90.5 vs. 68.6 %; $p < 0.001$)	No serious drug-related AEs occurred in either treatment group. Patients assigned to oral iron therapy were more likely to report gastrointestinal complaints, particularly constipation (11.2 vs. 3.4 %; $p = 0.07$), diarrhoea (3.9 vs. 0 %; $p = 0.015$) and nausea (7.3 vs. 1.1 %; $p = 0.006$) in comparison to ferric carboxymaltose. Patients assigned to intravenous ferric carboxymaltose were more likely to experience skin disorders (5.2 vs. 2.2 %; $p = 0.164$), principally mild pruritus and rash that usually resolved within 15 min from infusion

				**dose)**			

Changes to the Protocol (between Version 2.1 and Version 6.0)
Trial opened with trial protocol version 2.1

Subsequent changes to the protocol were to:

1. Add new substudies to the trial based on additional grant funding received.
2. Amend the timepoints and primary outcome due to changes necessitated by cessation of home visits due to the Pandemic.

In detail:

From Version 2.1 to 3.1:

- Addition of substudies:
    - Incorporated sub-studies to extend the follow-up period of women and their infants from 1-month post-partum to 12 months post-partum to assess the longer-term benefits and safety of intravenous iron (FCM). Participants would undergo study visits at 3,6,9 and 12 months, and mothers and babies would be tested for hematologic, iron, nutritional, infection and immunologic status. Women would undergo testing for wellbeing and depression, measured by the Depression, Anxiety and Stress Scale (DASS-21) and Edinburg Postpartum Depression Scale (EPDS). Babies would undergo cognitive testing, measured by the Bayley Scales of Infant Development, Event Related Potentials (ERPs), and Auditory Brainstem Responses (ABRs).
    - Added substudy to evaluate non-invasive test of haemoglobin (pulse oximetry-SpHb) against usual invasive tests (venous Hb), which could be of future benefit to diagnosing anaemia in settings like Malawi.

From Version 3.1 to 4.0:

- Main trial: Per COMREC Covid-19 Guidelines, included a proposed plan to maintain scientific validity and protect staff and research participants throughout the Covid-19 pandemic. The main change as part of the COVID-19 plan was the omission of home visits as part of follow-up.

From Version 4.0 to 5.0:

- Main trial: Included an adjustment to the statistical analysis section of the protocol.
- Substudies:
    - Adjusted the Maternal Psychological Health assessment tool (inclusion of the Mother-Infant Bonding Scale to assess mother-child interaction).
    - Included additional testing for maternal breast milk and malaria parasite parameters.

From Version 5.0 to 6.0:

- Main trial: Updated the primary outcome as the prevalence of anaemia (Hb<11g/dl) at 36 weeks' gestation.
- Additional substudies:
    - Added neuroimaging and bone assessment sub-studies The bone assessment (REVAMP-BONE) involved expanding measurement of bone health markers, as well as radiological examination of infants.
    - Measurement of hepcidin.
    - The neuroimaging assessment (REVAMP-Neuro) involved addition of a neurodevelopmental assessment tool (low field Magnetic Resonance Imaging) and neuroimaging assessments in selected participants.

Statistical Analysis Plan

TRIAL FULL TITLE	Randomized controlled trial of the Effect of intraVenous iron on Anaemia in Malawian Pregnant women
TRIAL SHORT TITLE	REVAMP
TRIAL REGISTRATION	Australian New Zealand Clinical Trials Registry Number: ACTRN12618001268235<br>WHO Universal Trial Number: U1111-1217-3954
PROTOCOL VERSION	5.0
TRIAL CHIEF INVESTIGATORS	Professor Kamija Phiri, University of Malawi<br>Professor Sant-Rayn Pasricha, Walter and Eliza Hall Institute of Medical Research
TRIAL STATISTICIAN	Dr Rebecca Harding, Walter and Eliza Hall Institute of Medical Research
SAP AUTHORS	Dr Rebecca Harding, Walter and Eliza Hall Institute of Medical Research<br>Ms Sabine Braat, University of Melbourne/Walter and Eliza Hall Institute of Medical Research<br>Professor Julie A Simpson, University of Melbourne<br>Dr Ricardo Ataide, Walter and Eliza Hall Institute of Medical Research
SAP VERSION	2.0
SAP VERSION DATE	11 April 2022

SAP Revision History

Version	Reason(s) for change	Date
1.0	Initial version for Gates open Research Publication	26 October 2021
2.0	Version before database unblinding incorporating reviewers' comments from the Gates open Research Publication	11 April 2022

SAP Signatures

I give my approval for the attached SAP entitled REVAMP dated 11 April 2022

Chief Investigators

Name / Affiliation	Signature	Date / time / time zone
Prof Kamija Phiri, University of Malawi		
Prof Sant-Rayn Pasricha, Walter and Eliza Hall Institute of Medical Research		

Trial Coordinator

Name / Affiliation	Signature	Date / time / time zone
Dr Ricardo Ataide, Walter and Eliza Hall Institute of Medical Research		

Trial Statistician

Name / Affiliation	Signature	Date / time / time zone
Dr Rebecca Harding, Walter and Eliza Hall Institute of Medical Research		

Senior Statisticians

Name / Affiliation	Signature	Date / time / time zone
Ms Sabine Braat, University of Melbourne/Walter and Eliza Hall Institute of Medical Research		
Prof Julie A Simpson, University of Melbourne		

Table of Contents

1 INTRODUCTION....178

1.1 PREFACE 178
1.2 PURPOSE OF THE ANALYSES 178

2 STUDY OBJECTIVES AND ENDPOINTS....178

2.1 STUDY OBJECTIVES 178
2.2 ENDPOINTS 179

3 STUDY METHODS....180

3.1 GENERAL STUDY DESIGN AND PLAN 180
3.2 INCLUSION-EXCLUSION CRITERIA AND GENERAL STUDY POPULATION 181
3.3 RANDOMISATION AND BLINDING 181
3.4 STUDY VISIT SCHEDULE 182
3.5 STUDY VARIABLES 185
3.5.1 *Maternal Study Variables*....*185*
3.5.2 *Neonate/Infant Study Variables*....*185*

4 SAMPLE SIZE....186

5 GENERAL CONSIDERATIONS....186

5.1 TIMING OF ANALYSES 186
5.2 ANALYSIS POPULATIONS 187
5.2.1 *Intention-to-Treat Population*....*187*
5.2.1.1 Mothers....187
5.2.1.2 Neonates/infants....187
5.2.2 *Per Protocol Population*....*187*
5.2.2.1 Mothers....187
5.2.2.2 Neonates/infants....188
5.2.3 *Safety Population*....*188*
5.2.3.1 Mothers....188
5.2.3.2 Neonates/infants....188
5.2.4 *Modified Safety Population*....*188*
5.2.4.1 Mothers....188
5.2.4.2 Neonates/infants....188
5.3 COVARIATES AND SUBGROUPS 188
5.3.1 *Covariates*....*188*
5.3.1.1 Maternal Covariates....189
5.3.1.2 Neonate/Infant Covariates....189
5.3.2 *Subgroups*....*189*
5.4 MISSING DATA 190
5.4.1 *Maternal outcomes*....*190*
5.4.2 *Neonate/Infant Outcomes*....*191*
5.5 INTERIM ANALYSES AND DATA MONITORING 191
5.6 MULTI-CENTRE STUDIES 191
5.7 MULTIPLE TESTING 192

6 SUMMARY OF STUDY DATA....192

6.1 SUBJECT DISPOSITION 192
6.2 DEMOGRAPHIC AND BASELINE VARIABLES 194
6.3 TREATMENT COMPLIANCE 195

7 EFFECTIVENESS ANALYSES....195

7.1 MATERNAL STUDY VARIABLES 195
7.1.1 *Maternal Anaemia*....*195*
7.1.2 *Secondary Maternal Analyses*....*196*
7.2 NEONATE/INFANT STUDY VARIABLES 196
7.2.1 *Birthweight*....*196*
7.2.2 *Secondary Neonate/Infant Analyses*....*197*

8 SAFETY ANALYSES 197

8.1 (SERIOUS) ADVERSE EVENTS 197

8.1.1 Maternal 198

8.1.2 Neonates/Infants 198

8.2 UNPLANNED CLINIC VISITS 199

8.2.1 Maternal 199

8.2.2 Neonates/infants 199

8.3 SAFETY BIOMARKERS/CLINICAL LABORATORY EVALUATIONS 199

8.3.1 Maternal 199

8.3.2 Neonates/infants 199

9 IMPACT OF COVID-19 PANDEMIC 199

10 TECHNICAL DETAILS 200

11 VALIDATION OF OUTPUT 200

12 SUMMARY OF CHANGES TO THE REGISTRY AND PROTOCOL 200

13 SUMMARY OF CHANGES BETWEEN VERSIONS 201

14 REFERENCES 202

15 LISTING OF KEY TABLES, LISTINGS AND FIGURES 204

Abbreviations and Definitions

ABBREVIATION	DEFINITION
AE	Adverse Event
ANC	Antenatal Care
CoM	College of Medicine
CONSORT	Consolidated Standards of Reporting Trials
CRF	Case Report Form
dL	Decilitre
DMC	Data Monitoring Committee
FCM	Ferric Carboxymaltose
Hb	Haemoglobin
HC	Health Centre
HIV	Human Immunodeficiency Virus
ID	Iron Deficiency
IDA	Iron Deficiency Anaemia
IPTp	Intermittent Preventive Treatment in pregnancy
ITT	Intention-To-Treat
IV	Intravenous
LMICs	Low- and Middle-Income Countries
MAR	Missing At Random
MCAR	Missing Completely At Random
MNAR	Missing Not At Random
NNT	Number-Needed-to-Treat
PO4	Phosphate
PP	Per Protocol
RCT	Randomised Controlled Trial
RDT	Rapid Diagnostic Test
SAE	Serious Adverse Event
SAP	Statistical Analysis Plan
SP	Sulfadoxine-Pyrimethamine
SpHb	Non-invasive Haemoglobin
WHO	World Health Organization
ZCH	Zomba Central Hospital

1 Introduction

1.1 Preface

Anaemia during pregnancy remains a critical global health problem. Almost 40% of pregnant women worldwide are anaemic, including 46% of pregnant women in Africa and 49% in Asia (2). Anaemia in pregnancy is very common and mostly results from iron deficiency and is associated with critical risks for both mother (e.g., life-threatening complications of postpartum haemorrhage) and child (especially prematurity and low birth weight, which are associated with increased risk of neonatal and infant mortality, and reduced iron stores in infancy with increased risk of subsequent anaemia and impaired development). (3-6)

Control of maternal anaemia, an important contributor to maternal mortality, is a key World Health Organization (WHO) nutrition target and a component of the third Sustainable Development Goal (7). Treatment with oral iron is poorly tolerated (8) and requires extended contact with a well-functioning health system; this is difficult to achieve, and few women receive full courses of iron. A new intravenous (IV) iron formulation, ferric carboxymaltose (FCM), now routinely available and used in developed countries, is safe and provides the opportunity to give high doses of iron in a single rapid infusion (9). In low and middle-income countries (LMICs), IV iron could present a novel, innovative opportunity to rapidly cure moderate and severe antenatal anaemia, thereby improving crucial maternal and neonatal outcomes.

REVAMP (Randomized controlled trial of the effect of intravenous iron on anaemia in Malawian Pregnant women) is a multicentre, open-label, two-arm, parallel-group randomised control trial (RCT) in antenatal clinics (ANC) in urban and rural areas of Blantyre and Zomba in Malawi (10) (ACTRN12618001268235). Women are eligible if they are in the second trimester of pregnancy and have a capillary haemoglobin concentration below 10.0g/dL. The study comprises two arms: (a) Intravenous Ferric Carboxymaltose (1000mg for women with weight >50kg, and 20mg/kg for women with weight <50kg) given once after randomisation (N=431), and (b) oral iron (65mg elemental iron twice daily) for 90 days (or the duration of pregnancy, whichever is shorter), provided according to local health care practices (N=431). In addition, both arms receive sulfadoxine-pyrimethamine (SP) as IPTp according to national guidelines. Participants are followed up until one month postpartum, after which we will report on the primary and secondary objectives.

1.2 Purpose of the analyses

This Statistical Analysis Plan (SAP) aims to outline the pre-planned analyses to be completed to support the main publication of the REVAMP study. Versions of the SAP will be tracked pre and post-unblinding, with a clear distinction between the changes before and after unblinding. Any analyses not identified in the SAP after unblinding of the randomised study treatment will be clearly identified as such in the main publication and will be considered post-hoc. In addition, extended follow-up of mothers and infants to 12-months postpartum is underway, as is a range of exploratory economic, biological and clinical outcomes. We will report these analyses separately as they are exploratory outcomes collected up to one month postpartum and thus are beyond the scope of this SAP.

2 Study Objectives and Endpoints

2.1 Study Objectives

The **primary objective** is to determine whether a single dose of FCM up to 1000mg is superior to standard-of-care (i.e., oral iron provided through local health services) in

reducing maternal anaemia before delivery (at 36 weeks' gestation) in Malawian women in the second trimester of pregnancy with moderate or severe anaemia.

The **secondary objectives** are to determine the effects of FCM (compared with standard-of-care) during pregnancy and up to one month postpartum on:

Effectiveness:

- Maternal haemoglobin concentration and iron status (measured through iron biomarkers), and
- Critical neonatal outcomes including birth weight (low birth weight), gestation duration (prematurity), small for gestational age and other perinatal outcomes,

Safety:

- Maternal and neonatal adverse events, including infection episodes, serious maternal complications, and hypophosphatemia.

2.2 Endpoints

To address the primary objective, we use the **primary endpoint** of maternal anaemia, defined as a venous haemoglobin concentration less than 11.0g/dL at 36 weeks' gestation (11).

To address the secondary objectives, we use the **secondary endpoints** as per below:

Effectiveness:

- Mother: anaemia (Hb<11.0g/dL) and moderate/severe anaemia (Hb<10g/dL) at 4 weeks' post-randomisation, and delivery: anaemia (Hb <12.0g/dL) and moderate/severe anaemia (Hb<11g/dL) at 1 month postpartum; haemoglobin concentration at 4 weeks post-randomisation, 36 weeks' gestation, delivery and 1 month postpartum; iron deficiency (ferritin <15 µg/L adjusted for C reactive protein), iron-deficiency anaemia and serum ferritin concentration all at 4 weeks post-randomisation, 36 weeks' gestation, and 1 month postpartum.
- Neonate: birthweight, gestation duration (weeks), birth length, low birth weight, fetal loss or stillbirth, premature birth, small for gestational age (both individually and as a composite) within 24 hours of birth, haemoglobin concentration, infant weight, infant length, and infant growth (weight for age, length for age, weight for length) at 1-month postpartum.

Safety:

- Mother: (serious) adverse events related to administration of FCM (events occurring during the enrolment visit), (serious) adverse events (within 14 days of randomisation, antenatal and postpartum reported separately), all-cause sick visits to the clinic (antenatal and postpartum reported separately), diarrhoea-related visits to the clinic (antenatal and postpartum reported separately), clinical malaria-specific visits to the clinic (antenatal and postpartum reported separately), hypophosphatemia (biochemical) (at four weeks' post-randomisation and 36 weeks' gestation), inflammation (elevated C-reactive protein) (at four weeks' post-randomisation and 36 weeks' gestation), and severe medical events including haemorrhage, need for transfusion, ICU admission, or mortality.
- Neonate: (serious) adverse events, all-cause visits to the clinic, diarrhoea-related visits to the clinic, respiratory-related visits to the clinic, and clinical malaria-specific visits to the clinic.

3 Study Methods

3.1 General Study Design and Plan

This study is a multicentre, open label, two-arm, parallel-group individually randomized controlled effectiveness and safety trial carried out in real-life settings in health facilities in Blantyre and Zomba districts of Southern Malawi. An open-label trial was chosen as it was considered unfeasible to deliver dark-coloured placebo intravenous infusions to Malawian pregnant women in this field context.

The trial randomized 862 pregnant women in their second trimester equally to (a) IV ferric carboxymaltose 1000mg (for women with weight >50kg), or 20mg/kg (for women with weight <50kg) once during the second trimester; or (b) oral iron 200mg ferrous sulphate (approx. 65mg elemental iron) twice daily for 90 days or the duration of pregnancy, whichever is shorter. Women received interventions and then were followed up until 1 month postpartum. The database will be locked, and the trial unblinded to report prespecified outcomes once all participants reach one-month postpartum. Planned extended follow-up of mothers and infants to 12-months postpartum will be reported in subsequent documents. The trial design is summarised in **Figure 1**.

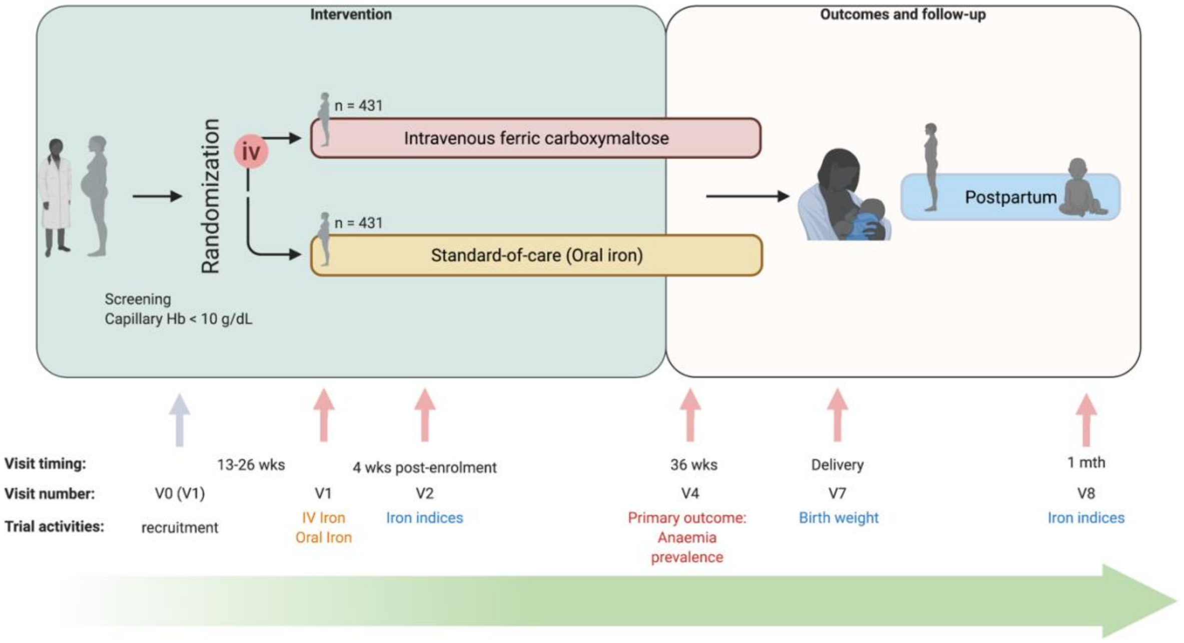

Figure 1 Study design and timeline of the Randomized controlled trial of the Effect of intraVenous iron on Anaemia in Malawian Pregnant women (REVAMP) (created with BioRender).

V0 – visit 0; V1 – Visit 1; V2 – Visit 2; V4 – Visit 4; V7 – Visit 7; V8 – Visit 8; Abbreviations: iv – intravenous; wks – weeks. The study was designed as a two-arm trial (intravenous ferric carboxymaltose versus standard-of-care (oral iron)) where women were randomized in their second trimester of pregnancy. Study visits occurred over pregnancy, at

birth, and follow-up to one-month postpartum. Women were scheduled to be visited in their home at 34 weeks' gestation (Visit 3), and every two weeks from 38 weeks' gestation until delivery (Visit 5 and 6) to measure capillary haemoglobin (Hb), not included in the study design schema.

3.2 Inclusion-Exclusion Criteria and General Study Population

Women were randomized only if they fulfilled all the inclusion criteria and none of the exclusion criteria.

Inclusion criteria:

1. Confirmed singleton pregnancy at 13-26 weeks' gestation
2. Moderate to severe anaemia (capillary haemoglobin <10g/dl measured by HemoCue 301+) not clinically deemed to require an immediate blood transfusion
3. Negative malaria parasitaemia at screening and no positive malaria rapid diagnostic test within the previous 7 days
4. Resident in the study catchment area of Blantyre and Zomba district
5. Plan to deliver at health facility
6. Written informed consent, including assent if <18 years old (consent of a parent or guardian plus the participant's assent).

Exclusion criteria:

1. Hypersensitivity to any of the study drugs
2. Clinical symptoms of malaria or positive malaria rapid diagnostic test within the previous seven days or symptoms of bacterial infection, at screening
3. Any condition requiring hospitalization or serious concomitant illness
4. Chronic illness that may adversely affect foetal growth and viability
5. Severe anaemia clinically judged as requiring a blood transfusion (usually Hb<5.0 g/dL)
6. Pre-eclampsia at screening (new onset of hypertension, proteinuria and swelling in the legs, feet, and hands).

3.3 Randomisation and Blinding

Participants are randomly allocated to one of the two treatments arms with 1:1 allocation via a computer-generated randomisation schedule of randomly permuted blocks stratified by site (Blantyre, Zomba) to achieve balance between the arms within each site. The randomisation list was generated by an independent statistician at the University of Melbourne (Australia) who will not reveal the block size until the database is ready for unblinding.

Individual participant codes are pre-packed in envelopes by an independent researcher not associated with the study and held securely at research sites. Eligible participants who meet all inclusion/exclusion criteria are sequentially allocated participant identification numbers within the research site, and their allocation to study group is revealed after opening the corresponding sealed envelope.

Although the trial is open-label, laboratory scientists measuring haemoglobin concentration, midwives collecting birth outcome data, investigators and researchers in Australia (including data managers and statisticians in Melbourne) are blinded to the allocation during the conduct of the trial until the database is locked and ready for unblinding.

An open-label (rather than blinded) trial was chosen as it was deemed that it would be ethically and operationally challenging to provide placebo infusions to women during pregnancy. In particular, FCM is black in colour, and thus would require either use of opaque

tubing, a curtain and veil to conceal the tubing or a blindfold for the participant – none of which are likely to be feasible in practice.

3.4 STUDY VISIT SCHEDULE

Table 1 shows the schedule of activities per visit for the mother, and Table 2 shows the schedule of activities per visit for the neonate/infant.

Table 1: Maternal Study Visit Schedule

Protocol Activity	Visit 0	Visit 1	Visit 2	Visit 3[j]	Visit 4	Visit 5 – 6[j]	Visit 7	Visit 8
	Day -7 to 0	Day 0	Day 28 ±2 days	34 wks. gestation ±2 days	36 wks. gestation ±2 days	38/40 wks. gestation ± 2 days	Delivery + 1 day	Day 28 Postpartum ±2 days
Location of visit	***Antenatal Clinic***	***Research Site[a]***	***Research Site[a]***	***Home[b]***	***Research Site[a]***	***Home[b]***	***Research Site[a]***	***Research Site[a]***
**Informed consent process for pre-screening**	X							
**Pre-screening**	X							
**Detailed Informed consent process**	X	X						
**Screening**		X						
**Medical & obstetric history**		X						
**Demographics**		X						
**Maternal physical examination[d]**		X[h]	X[i]		X[i]		X[h]	X[i]
**Ultrasound scan (Foetal Biometry)[d]**		X	X		X			
**Randomise participant**		X						
**Administer treatment**		X						
**Intermittent preventive treatment[c]**		X	X		X			
Laboratory procedures								
**Capillary Haemoglobin[d]**	X	X	X	X	X	X	X	X

**Full Blood Count[d]**		**X**	**X**		**X**		**X**	**X**
**Malaria diagnostics [d, e]**	**X**	**X**	**X**		**X**		**X**	**X**
**Serum for iron markers tests[f]**		**X**	**X**		**X**		**X**	**X**
**Serum for inflammatory markers tests[g]**		**X**	**X**		**X**		**X**	**X**
**Phosphate**		**X**	**X**		**X**		**X**	**X**
**Adverse events[d]**		**X**	**X**		**X**		**X**	**X**
**Morbidities**								**X**
**Missed Visits[d]**			**X**	**X**	**X**	**X**	**X**	**X**
**End of Study**			**X**	**X**	**X**	**X**	**X**	**X**

[a] Visit conducted at the research site/health facility; [b] Visit conducted at the participant's home; [c] Assessing if intermittent preventive treatments Sulphadoxine Pyrimethamine and Albendazole were given; [d] Protocol activities are also collected at any unscheduled visits; [e] Malaria diagnostics include Malaria rapid diagnostic test, Malaria microscopy, [f] Serum for iron markers tests include Serum ferritin; [g] Serum for inflammatory markers tests include C-reactive protein and alpha-1 glycoprotein; [h] Complete Maternal physical examination includes general appearance, throat, neck, thyroid, musculoskeletal, skin, lymph nodes, extremities, pulses, pulmonary, cardiac, abdominal, and neurological examination; [I] Limited Maternal physical examination includes general appearance, brief pulmonary, cardiac, abdominal and neurological examination. [j] Since the opening of the trial, visits 3, 5 and 6 have been suspended since April 2020 due to the risk of exposing communities to COVID-19 during home visits.

Table 2 Neonate Study Visit Schedule

Protocol Activity	Visit 7	Visit 8
	Delivery +1 day	Day 28 Postpartum ±2 days
Location of visit	*Research Site*[a]	*Research Site*[a]
Pregnancy outcome	X	
Physical examination and anthropometry [b, c]	X[f]	X[g]
Laboratory procedures		
Capillary Haemoglobin	X[h]	
Full Blood Count[b]	X[h]	X
Malaria diagnostics[d]	X[h]	X
Serum for iron markers tests[e]	X[h]	X
Vaccination and Vit A supplementation status		X
Adverse events[b]	X	X
Morbidities		X
Missed Visits[b]		X
End of Study	X	X

[a] Visit done at the research site/ health facility; [b] Protocol activities are also collected at any unscheduled visits; [c] Anthropometry includes assess the infant's weight, length, and head circumference; [d] Malaria diagnostics include Malaria rapid diagnostic test, Malaria microscopy, Malaria filter paper for polymerase chain reaction and histology at delivery; [e] Serum for iron markers tests include Serum ferritin; [f] The Complete physical examination includes general appearance, throat, neck, thyroid, musculoskeletal, skin, lymph nodes, extremities, pulses, pulmonary, cardiac, abdominal, and neurological examination [g] The Limited examination includes general appearance, brief pulmonary, cardiac, abdominal and neurological examination; [h] These assessments are collected via cord blood.

In accordance with the study visit schedule, we will use time-windows to convert visit dates into visit numbers. Relative days will be derived as the visit date minus the randomisation date. The visit windows in Table 3 will be applied to the relative days.

Table 3 Visit windows

Visit	Target Day	Lower limit	Upper limit
Visit 1 (Clinic)<br>Day 0 – Randomisation date	Day 0	-7 days	0 days
Visit 2 (Clinic)<br>28 days post-randomisation	Day 28	18 days post-randomisation	38 days post randomisation
Visit 4 (Clinic)<br>36 weeks' gestation	36 weeks + 0 days gestation	34 weeks + 0 days	38 weeks + 0 days
Visit 7 (Clinic)*<br>Delivery	Day of delivery	-2 days **	2 days post-delivery
Visit 8 (Clinic)*<br>28 days postpartum	28 days post-delivery	21 days post-delivery	42 days post-delivery

*This visit window applies to both mother and neonates
**This lower limit only applies to mothers, the lower limit of delivery for neonates is the day of delivery.

Assessments performed outside these visit windows will not be included in the analyses. If a study participant has more than one assessment done within the same visit window, then the assessment closest to the target date will be used. The visit windows will be applied to the study variables described below in Section 3.5.

3.5 Study Variables

A detailed description of the effectiveness study variables, grouped according to domain, is presented in Sections 3.5.1 to 3.5.2. We define the domains as maternal (including women who experienced pregnancy loss) and neonate/infants. All safety study variables are detailed in Section 8. Note that while C-reactive protein is discussed in Section 3.5.1, it is considered a safety study variable.

3.5.1 Maternal Study Variables

At baseline, 28 days post randomisation, 36 weeks' gestation, delivery and 1-month postpartum, venous blood samples are collected from the mother to obtain the mothers haemoglobin (g/dL), ferritin (µg/L), and C-reactive protein (µg/L). Values of C-reactive protein < 0.2 µg/L (lower limit of detection), will be replaced by the value 0.1 µg/L. In the case of haemolysis (rare occurrence), C-reactive protein will be set to missing. We will then derive the following variables:

- anaemia (haemoglobin<11.0 g/dL) or not up to and including delivery,
- anaemia (haemoglobin<12.0 g/dL/0 or not postpartum,
- mild anaemia (10.0 g/dL≤haemoglobin<11.0 g/dL), moderate anaemia (7.0 g/dL≤haemoglobin<10.0 g/dL), severe anaemia (haemoglobin<7.0 g/dL), or not up to and including delivery,
- mild anaemia (11.0 g/dL≤haemoglobin<12.0 g/dL), moderate anaemia (8.0 g/dL≤haemoglobin<11.0 g/dL), severe anaemia (haemoglobin<8.0 g/dL), or not postpartum,
- iron deficient (ID) (ferritin<15 ug/L or ferritin <30 ug/L if C-reactive protein >5 µg/L) or not,
- iron deficient anaemia (IDA) (iron deficient and anaemia) or not,
- inflammation (C-reactive protein >5µg/L) or not, and
- anaemia and inflammation (anaemia and inflammation).

3.5.2 Neonate/Infant Study Variables

Fetal loss (pregnancy loss before 22 completed weeks' gestation), stillbirth (pregnancy loss after 22 weeks and prior to delivery with no evidence of life at delivery) and neonatal death (death in the first 28 days of life of a child with evidence of life following delivery) will be recorded. Within 24 hours of delivery, live-born neonates will have a full physical examination including measurement of birth weight (grams) and birth length (cm). Using these physical examination indices the study variable low birthweight (birthweight < 2500g) or not will be derived. Using gestation duration (in weeks) (as derived by Second-trimester obstetric ultrasonography), study variables indicating premature birth (<37 weeks gestation) or not, and small for gestational age (according to INTERGROWTH-21 standards) (12) or not will be derived. A composite outcome of adverse birth outcomes will be defined as the presence of pregnancy loss or stillbirth, low birthweight, premature birth, or small for gestational age.

At 1-month of age, venous blood samples are collected from the child to obtain the child's haemoglobin (g/dL). Also, the child's weight and length will be collected. Together with the age and sex of the child, z-scores will be derived for length-for-age, weight-for-age, and weight-for-length according to age and sex-specific WHO international reference growth standards (13). We will be using the WHO Anthro Software on the WHO website using the web-links available on the webpage https://www.who.int/childgrowth/software/en/.

4 Sample Size

The sample size calculation is based on the maternal primary outcome of the proportion of women with pre-delivery anaemia (defined as venous Hb<11.0g/dL at 36 weeks' gestation). A systematic review and meta-analysis identified a 50% reduction in anaemia prevalence in women receiving oral iron (3). In a cohort of pregnant women in the Gambia, we observed that 60% of pregnant women with Hb<10g/dL at 20 weeks' gestation who then receive iron interventions continue to have Hb<10g/dL by 30 weeks (14). The pivotal Fer-ASAP trial (which compared FCM to oral iron in women with iron deficiency anaemia in high-income settings) demonstrated a 14% reduction in absolute anaemia prevalence compared with oral iron (15). We hypothesise that routine iron supplementation will reduce anaemia prevalence from 100% to 60% (as seen in the Gambia (14), and similar to data from Haider and colleagues (3)). We hypothesise that ferric carboxymaltose will result in a 10% absolute improvement in anaemia cure (to a prevalence of 50%). A total sample size of 862 pregnant women or 431 per arm will provide 80% power to detect this difference, allowing for a prevalence of 50%). A total sample size of 862 pregnant women or 431 per arm will provide 80% power to detect this difference, allowing for a 10% lost to follow-up and assuming a two-sided alpha of 5%. The sample size also has at least 80% power to detect an absolute difference between standard-of-care and ferric carboxymaltose of 100g in the neonatal outcome of birth weight (assuming a standard deviation of 450g and a two-sided alpha of 5%), similar to the effect size seen in a trial of Kenyan women receiving oral iron when compared to placebo (16). Sample size calculations were performed using Stata/SE (StataCorp. 2019. College Station, TX: StataCorp LLC).

5 General Considerations

5.1 Timing of Analyses

Study data are entered and stored in the study database at the Training and Research Unit of Excellence (TRUE), Malawi. Laboratory indices are analysed at TRUE and Meander Medical Centre laboratory, accreditation number M040, EN ISO 15189:2012 (Amersfoort, The Netherlands) (for ferritin, phosphate, C-reactive protein). After all data supporting the main results are available and have been cleaned, data (except post-randomisation ferritin,

phosphate, and C-reactive protein) will be transferred to the trial statistician located at the Walter and Eliza Hall Institute of Medical Research, Melbourne, Australia. A blinded data review meeting will be held prior to database lock (defined as its state is kept constant). During this blinded data review meeting, at least the following topics will be discussed and decided upon without knowledge of the underlying treatment code:

1. Study participants (mothers or neonates/infants) who have withdrawn consent, in relation to the use of the study participant's data (or part of it) in any of the analysis populations (Section 5.2). Data will be used up to the withdrawal of consent.
2. Study participants (mothers or neonates/infants) with protocol violations as defined in Section 5.2.2, in relation to the use of the study participant's data (or part of it) in the per protocol population (Section 5.2.2).
3. Study participant's (mothers and neonates/infants) inclusion or exclusion status with regard to each analysis population (Section 5.2) guided by items 1-2 listed earlier.
4. Study participant's (mothers and neonates/infants) as-randomised vs as-treated (e.g. randomised to standard-of-care but received IV iron).
5. Auxiliary variables to be included in the additional analyses outlined in Section 5.4.

After database lock, the randomized treatment allocation will be obtained from the independent statistician who generated the randomisation code. The trial statistician will then add the post-baseline laboratory data of ferritin, phosphate, C-reactive protein from Meander Medical Centre laboratory, accreditation number M040, EN ISO 15189:2012 (Amersfoort, The Netherlands) to the study database. No database may be locked, randomised treatment unblinded, or analyses completed until the SAP has been approved (in case of updates since its initial approval). The planned analyses in this SAP will be conducted after unblinding of the database and any changes to this SAP after unblinding will be documented in an amendment of this SAP and considered post-hoc analyses.

5.2 Analysis Populations

Participating mothers and their neonates/infants will be reported and analysed according to their randomised study treatment ("as-randomised") for 5.2.1 and 5.2.2 and according to their actual study treatment ("as-treated") for 5.2.3. The following analysis populations are planned.

5.2.1 Intention-to-Treat Population

5.2.1.1 Mothers

This will consist of all women who were randomised, excluding those who have withdrawn informed consent for the use of all their data. This population will be used in the analysis of the maternal effectiveness study variables.

5.2.1.2 Neonates/infants

This will consist of all live-born neonates/infants (with the exception of the stillbirth outcome) of mothers who were randomised, excluding those who have withdrawn informed consent for the use of all their data. This population will be used in the analysis of the infant effectiveness study variables.

5.2.2 Per Protocol Population

5.2.2.1 Mothers

This will consist of all women who were randomised, and without protocol violations. A protocol violation is defined as those who have withdrawn informed consent for the use of all their data or violating in/exclusion criteria (e.g., twin pregnancy). These protocol violations are based on pre-randomisation characteristics only and expected to be balanced between treatment groups. Mothers who are found to be non-adherent to treatment (e.g., refused or not provided treatment after randomisation) will be excluded from the per-protocol (PP) population analysis. This population will be used in the analysis of the maternal effectiveness study variables.

5.2.2.2 Neonates/infants

This will consist of all neonates/infants born to mothers who were randomised, and without protocol violations. A protocol violation is defined as those who have withdrawn informed consent for the use of all their data or the mother being a protocol violation. Neonates of mothers who are found to be non-adherent to treatment (e.g., refused or not provided treatment after randomisation) will be excluded from the per-protocol (PP) population analysis. This population will be used in the analysis of the infant effectiveness study variables.

5.2.3 Safety Population

5.2.3.1 Mothers

This will consist of all women who received at least one study treatment (either IV iron or standard-of-care), excluding study participants who have withdrawn informed consent for use of all their data. This population will be used in the analysis of the maternal safety study variables.

5.2.3.2 Neonates/infants

This will consist of all neonates/infants born to mothers who received at least one study treatment (either IV iron or standard-of-care), excluding study participants who have withdrawn informed consent for use of all their data. This population will be used in the analysis of the infant safety study variables.

5.2.4 Modified Safety Population

5.2.4.1 Mothers

This will consist of all women who received at least one study treatment (either IV iron or standard-of-care), and without a singleton pregnancy protocol violation, excluding study participants who have withdrawn informed consent for use of all their data. This population will be used in the analysis of the maternal safety study variables.

5.2.4.2 Neonates/infants

This will consist of all neonates/infants born to mothers who received at least one study treatment (either IV iron or standard-of-care), and without a singleton pregnancy violation, excluding study participants who have withdrawn informed consent for use of all their data. This population will be used in the analysis of the infant safety study variables.

5.3 Covariates and Subgroups

5.3.1 Covariates

The analysis model for all maternal and neonate/infant outcomes will be adjusted for the randomisation stratification variable of site as a main effect. This model will be referred to as the unadjusted model.
In addition, adjusted analysis models will be fitted to some of the effectiveness outcomes as specified in Section 7, separately for mothers and neonates/infants.

5.3.1.1 Maternal Covariates

1. First adjusted model: Adding to the unadjusted model demographic and/or baseline covariates as main affects assumed to be (strongly) prognostic or predictive of maternal anaemia. These covariates are: parity (primiparous vs. multiparous), gestational age at baseline (continuous), and BMI at baseline (continuous).
2. Second adjusted model: Adding to the first adjusted model laboratory biomarkers as main effects assumed to be (strongly) prognostic or predictive of maternal anaemia. These covariates are: inflammation status at baseline, iron deficient status at baseline, haemoglobin at baseline (continuous), HIV positive status at baseline, and re-screened post positive malaria RDT status.
3. Third adjusted model: Given the large sample size, imbalance in demographic and/or baseline characteristics is not expected. However, in case of unexpected imbalance a third adjusted model will be fitted by adding those covariates identified after unblinding as main effects to the second adjusted model.

5.3.1.2 Neonate/Infant Covariates

1. First adjusted model: Adding to the unadjusted model demographic and/or baseline covariates as main effects assumed to be (strongly) prognostic or predictive of birth weight. These covariates are: sex (female or male), gestational age at baseline (continuous), and maternal BMI at baseline (continuous).
2. Second adjusted model: Adding to the first adjusted model laboratory biomarkers as main effects assume to be (strongly) prognostic or predictive of birthweight. These covariates are: maternal haemoglobin at baseline (continuous).
3. Third adjusted model: Given the large sample size, we do not expect an imbalance in demographic and/or baseline characteristics. However, a third adjusted model will be fitted in case of unexpected imbalance by adding those covariates identified after unblinding as main effects to the second adjusted model.

5.3.2 Subgroups

Exploratory subgroup analyses will be performed for the outcomes of maternal anaemia at 36 weeks' gestation, haemoglobin concentration at 36 weeks' gestation, birth weight, low birth weight, gestation duration, and premature birth for the ITT population. The eight subgroups analyses listed below will be performed irrespective of whether the study's primary objective based on maternal anaemia at 36 weeks' gestation was achieved.

a) Parity (primiparous vs. multiparous)
b) Baseline HIV status (positive vs negative)
c) Baseline severe anaemia status (yes vs no severe anaemia)
d) Baseline iron deficient status (yes vs no ID)
e) Baseline iron deficient anaemia status (yes vs no IDA)
f) Baseline inflammation status (yes vs no elevated CRP)
g) Re-screened after positive malaria RDT at pre-screening (yes vs no)

h) Site (Blantyre, Zomba)

We hypothesise that mothers with severe anaemia, ID, or IDA at baseline will have a larger treatment effect compared with those who are non-severe anaemic, non-iron deficient, or non-iron deficient anaemic, respectively.

Subgroup (main effect) and the subgroup-by-treatment interactions term will be added to the unadjusted model to evaluate whether the treatment effect (IV iron versus standard-of-care) differs between subgroup categories. Results of the subgroup analyses will be displayed using Forest plots.

5.4 MISSING DATA

5.4.1 Maternal outcomes

To describe the missing data, the frequency and percentage of study participants with missing data at baseline, 28 days post randomisation, 36 weeks' gestation, delivery and one month postpartum will be summarised for anaemia (mothers) by treatment group using the ITT population. In addition, baseline and demographic characteristics will be summarised by those with baseline only, incomplete data at any visit, and complete data at all visits for anaemia (mothers) to explore the missing data assumption(s) and identify any study variables not included in the target analyses that are potentially associated with missing/not missing of these study variables (known as auxiliary variables).

As the primary strategy to handle missing data, the analysis of maternal anaemia will use a likelihood based approach; detailed in Section 7.1.1. This approach relies on the underlying assumption that the probability of missing outcome data is not related to the missing data after conditioning on observed data in the model (Missing at Random [MAR]). As a rule of thumb, if the proportion of missing data is below approximately 5%, those values are considered to be missing at random. Missing data will be considered neglible in this case for maternal anaemia (17), in which case we will refer to the analysis as a modified intention-to-treat analysis.

If the missing data is not negligible, an additional analysis will be performed separately by treatment group whereby missing maternal haemoglobin data will be multiply imputed using chained equations. The imputation model will include site, visit (categorical), and all variables listed in the second adjusted model described in Section 5.3.1.1. In addition, auxiliary variables identified during the blinded data review meeting may be included. None of the characteristics were noteworthy different between those with and without missing data. Therefore, no auxiliary variables were identified during the blinded data review. Maternal haemoglobin will be imputed using a linear regression model. The missing outcome data at baseline, 28 days post-randomisation, 36 weeks' gestation, delivery and one month postpartum will be imputed using the "just another variable" approach (also known as imputing in wide format) which requires a separate imputation model for imputing the variable at each assessment time (18). The number of imputed data sets will be greater than or equal to the percentage of missing data in the available case analyses. Using these imputed data sets, an analysis based on a pattern-mixture model (19) consisting of applying a delta-adjustment to the imputed values by treatment group will be conducted. Within the standard-of-care group participants with missing data will be assumed having both a poorer and better response than those with observed data. No *a priori* difference is anticipated in the mean response for the IV iron group. Differences in baseline participant characteristics between those with and without data will inform delta-values to explore in both treatment groups. After comparing participants with and without haemoglobin at 36 weeks' gestation (primary timepoint) during the blinded data review, it will be assumed that a participant with missing data responds similarly to either iron intervention. Based on the baseline participant

characteristics, a delta-adjustment of -2g/dL to +2g/dL (in steps of 0.5g/dL) will be applied to the imputed values of both the standard-of-care (oral iron) and IV iron groups. After deriving maternal anaemia from the imputed haemoglobin values, the imputed data sets will be analyzed using the model described in Section 7.1.1. The estimates from the analyses of the imputed data sets will be combined to obtain a pooled common estimate and corresponding confidence interval for the effect of the iron intervention on maternal anaemia using Rubin's rules. The delta-adjustment method within the multiple imputation framework assumes a Missing Not At Random (MNAR) assumption for the outcome.

5.4.2 Neonate/Infant Outcomes

The analysis of birthweight will use a complete-case analysis among those live-born; details hereof are described in Section 7. This approach relies on the underlying assumption that the probability of missing outcome data is not related to the observed or missing data (Missing Completely at Random [MCAR]). Missing data will be considered negligible if approximately less than 5% of live-born infants have missing birthweight, in which case we will refer to the analysis as a modified intention-to-treat analysis.

If the missing data is not negligible, an additional analysis will be performed whereby missing birthweight data will be multiply imputed, separately by treatment group. The imputation model will include site, visit (categorical), and all variables listed in the first adjusted model described in Section 5.3.1.2. In addition, we may include auxiliary variables identified during the blinded data review meeting. Birthweight will be imputed using a linear regression model. The number of imputed data sets will be greater than or equal to the percentage of missing data in the complete case analyses. The estimates from the analyses of the m imputed data sets will be combined to obtain a pooled common estimate and corresponding confidence interval for birthweight using Rubin's rules. This approach relies on the MAR assumption for the outcome, birthweight.

5.5 Interim Analyses and Data Monitoring

An independent Data Monitoring Committee (DMC) was constituted and provided oversight of the study. Interim analyses to stop the trial early were neither planned nor conducted.

A total of two DMC meetings were held before unblinding the randomised treatment allocation. The recommendation of both was continue the study as planned without any modification.

**Meeting**	**Date**	**Outcome**
1st meeting	19 June 2019	Continue as planned
2nd meeting*	20 March 2021	Continue as planned

* Due to COVID-19 associated clinical workloads for DSMB members, the DSMB report was shared electronically with the committee, who communicated that the study should continue.

5.6 Multi-centre Studies

This is a multicentre study that was carried out in the following two sites:

- Zomba Central Hospital (ZCH), in southern Malawi, was the main coordinating site with three main satellite health centres (within a 5-minute drive distance) and other health centres as approved by the District Health Office where participants will be screened.

- Blantyre (the hub of health research in Malawi) involved three major health centres (HCs) namely Limbe HC, Bangwe HC, and Ndirande HC which are all less than 10 kilometres apart and other health centres as approved by the District Health Office. Limbe HC was the recruitment site, with the other two as satellite screening sites.

5.7 Multiple Testing

We ordered study variables by degree of importance. The following comparison and hypothesis is defined for the effectiveness study variables:

H0: There is no difference between IV iron and Standard-of-care

H1: There is a difference between IV iron and Standard-of-care

For the primary maternal endpoint (anaemia at 36 weeks' gestation) and key neonate endpoint (birthweight), no adjustment for multiplicity is planned. The above hypothesis will be tested at a two-sided 5% significance level for each endpoint separately.

The above hypothesis will be tested using the Holm procedure (20) to control the Type I error rate for following set of maternal and neonate/infant outcomes separately:

- Maternal: Hb at 36 week's gestation, moderate/severe anaemia at 36 weeks' gestation, ferritin at 36 weeks' gestation, ID at 36 week's gestation, IDA at 36 week's gestation.
- Neonate/infant: gestation duration, composite adverse birth outcome, Hb at 1-month postpartum, weight at 1-month postpartum.

We will report multiplicity unadjusted P-values along with the estimate and 95% confidence intervals and footnote the comparisons meeting the statistical significance threshold according to the Holm procedure.

For effectiveness endpoints/study variables not listed above, we will present the estimate and two-sided 95% confidence intervals (no P-Values). No adjustment is planned. This includes the components of the composite several medical event and adverse birth outcome.

No multiplicity adjustment is planned for the safety endpoints/study variables, P-Values will be presented.

No multiplicity adjustment is planned for the subgroup analyses (Section 5.3.2) because they are considered exploratory, P-Values will be presented.

6 Summary of Study Data

6.1 Subject Disposition

The flow of study participants will be presented in a Consolidated Standards of Reporting Trials (CONSORT) diagram. The diagram will include the number of study participants within the following categories:

Pre-Screening	• Pregnant women assessed for eligibility<br>• Women excluded<br>• Did not meet eligibility criteria (reasons)*
Screening & Enrolment	• Women excluded<br>• Did not meet eligibility criteria (reasons)*<br>• Declined to participate†<br>• Other reasons
Allocation	• Randomised IV iron (plus IPTp) or oral iron (plus IPTp)

	• Received allocated intervention‡<br>• Did not receive allocated intervention (reasons)<br>• Did not receive IPTp<br>• For IV iron only: Discontinued IV
Day 28 follow-up	• Assessed for maternal Hb at 28 days follow-up, if not:<br>• Lost-to follow-up (reasons)<br>• Discontinued (reasons)<br>• Attended the visit<br>• Not attended the visit
36 weeks' gestation	• Assessed for maternal Hb at 36 week's gestation, if not:<br>• Lost-to follow-up (reasons)<br>• Discontinued (reasons)<br>• Attended the visit<br>• Not attended the visit
Delivery	• Assessed for maternal Hb at delivery, if not:<br>• Lost-to follow-up (reasons)<br>• Discontinued (reasons)<br>• Assessed for birth weight at delivery, if not:<br>• Lost-to follow-up (reasons)<br>• Discontinued (reasons)<br>• Mothers:<br>• Attended the visit<br>• Not attended the visit<br>• No. of deliveries:<br>• Stillbirth<br>• Neonatal deaths
Day 28 postpartum	• Assessed for maternal Hb at 28 days postpartum, if not:<br>• Lost-to follow-up (reasons)<br>• Discontinued (reasons)

	• Mothers:<br>• Attended the visit<br>• Not attended the visit<br>• Assessed for child's Hb at 28 days postpartum, if not:<br>• Lost-to follow-up (reasons)<br>• Discontinued (reasons)<br>• Children:<br>• Attended the visit<br>• Not attended the visit
Analysis	• Mothers analysed (i.e. at least one maternal Hb value)<br>• Excluded from analysis (reasons)<br>• Neonates/infants analysed (i.e. at least birthweight)<br>• Excluded from analysis (reasons)

*Reasons for not meeting eligibility were assessed on the questions on the eligibility data collection form.
†Defined as those who answered No to the question "Accepts study procedures?" at enrolment.
‡Reasons for not receiving or not provided with the treatment were collected on the participant randomisation form.

6.2 Demographic and Baseline Variables

Demographic and baseline variables will be summarised for the ITT and PP population and presented by treatment group in the order of (IV iron, standard-of-care) using frequencies and percentages (based on the non-missing sample size) for categorical variables, mean and standard deviation for continuous variables, or median and quartiles (25th and 75th percentile) for non-symmetrical continuous variables. If variables have missing values, we will report the non-missing sample size either in the table or in the footnote of the table. No P-Values will be reported to compare treatment arms.
We will present the following variables:

- General: site, age (years) at enrolment*, primiparous, primigravid, gestational age at enrolment, religion, maternal education (education level), marital status, income source, re-screened post positive malaria RDT.
- Maternal laboratory indices: HIV positive, malaria RDT positive at enrolment, capillary haemoglobin < 10.0 g/dL, venous haemoglobin, (venous) anaemia (mild, moderate, severe), ferritin (ug/L), iron deficient, iron deficient anaemia, C-reactive protein (µg/L), inflammation, (venous) anaemia and inflammation.
- Maternal anthropometry: maternal height (cm), maternal weight (kg), maternal BMI (kg/m^2).

*derived using the date of randomisation visit and maternal date of birth

6.3 Treatment Compliance

All randomised women were planned to be provided with SP as IPTp according to national guidelines, those who received at least one dose or with contraindications will be reported as part of the CONSORT diagram.

In addition:

- FCM/IV iron group: After randomisation, all participants in the IV iron arm had FCM administered by the field team unless the participant refused the treatment. Discontinuation of IV iron during administration was recorded. Information on IV administration, refusal, and discontinuation will be reported as part of CONSORT diagram.
- Standard-of-care/oral iron group: After randomisation, all participants in the oral iron arm were planned to be provided with oral iron to be taken twice daily for 90 days or the duration of pregnancy, whichever is shorter. Information on distribution of oral iron will be reported as part of the CONSORT diagram.

7 Effectiveness Analyses

For all effectiveness analyses, we will use the following principles:

- We will use the ITT population for all effectiveness analyses. In addition, we will use the PP population for selected study variables.
- Due to the low number of multiple pregnancies (exclusion criterion), we will not account for the correlation between neonates from the same mother in the ITT analysis but will exclude these mothers/children from the PP analysis.
- Comparisons of interest are IV iron versus standard-of-care at 28 days post randomisation, 36 weeks' gestation, delivery, and 28 days postpartum, whereby the maternal primary endpoint is 36 weeks' gestation, and the neonate key endpoint is delivery.
- Confidence intervals and P-Values will be presented following the multiple testing strategies described in Section 5.6.
- All analyses will be adjusted for the stratification variable used during the randomisation (site), referred to as the unadjusted analyses.
- An available case analysis will be performed for repeated time point outcomes and a complete case analysis for single time point outcomes.

7.1 Maternal Study Variables

7.1.1 Maternal Anaemia

Maternal anaemia (primary outcome) at 28 days follow-up, 36 weeks' gestation (primary timepoint), delivery and 28 days postpartum will be analysed for the ITT population using a log-binomial regression model, including study participants (mothers) as a random intercept to account for the multiple time points. The model will include a treatment and treatment by study visit interaction and adjust for the stratification variable used during the randomization (site). The model will include the standard-of-care (oral iron) group as the reference group. We will report the estimate of the prevalence ratio of IV iron versus standard-of-care (oral iron), two-sided 95% confidence interval extracted at 36 weeks' gestation (primary time

point) and P-Value. From this same model we will also extract the treatment effect at 28 days follow-up, delivery and 28 days postpartum. In case of non-convergence, we will fit a modified Poisson regression model with robust error variance, including study participants (mothers) as a random intercept to account for the multiple time points. The treatment effect will be estimated from this model as the prevalence ratio of IV iron versus standard-of-care (oral iron). We have selected the prevalence ratio as an effect measure because its interpretation as the ratio change in prevalence is easier to understand than the interpretation of the odds ratio for clinical researchers.

In addition to the analysis described above, we will perform:

- The three adjusted analyses described in Section 3.5.1.1
- The missing data additional analyses described in Section 5.4.1
- The model described above on the PP population
- The model described above on the PP population adjusted for baseline characteristics considered not balanced between the arms for the PP population

Furthermore, we will report the number-needed-to-treat (NNT) and 95% confidence interval for maternal anaemia at 36 week's gestation.

7.1.2 Secondary Maternal Analyses

The repeated time point continuous outcomes haemoglobin concentration and ferritin concentration will be analysed using a likelihood-based longitudinal data analysis model for the ITT population (21). The outcome (dependent variable) will consist of the baseline, 28 days follow-up, 36 weeks' gestation, delivery, and 28 days postpartum values. The model will assume a common baseline mean across the two treatment groups due to random allocation. It will incorporate time point (study visit) as a categorical variable; thus it will not assume a specific trajectory over time. In addition to study visit, the model will include the stratification factor (site) and treatment as main factors and the treatment by study visit interaction. The variance–covariance among the repeated measurements (baseline, 28 days follow-up, 36 weeks' gestation, delivery, and 28 days postpartum) will be defined as unstructured. In case of non-convergence, we will consider alternative structures (first-order autoregressive, Toeplitz, compound symmetry). Using this model, study participants with a missing baseline or a missing post-baseline outcome will be included in the analysis but study participants with no outcome values will be excluded. We will obtain the estimate and two-sided 95% confidence interval of the mean difference in change from baseline to postbaseline between IV iron and standard-of-care (oral iron) and P-Values, whereby 36 weeks' gestation is the primary timepoint. Furthermore, we will display the estimate and 95% confidence interval of haemoglobin and anaemia over time in a Figure. Ferritin will be log base e transformed before fitting the model due to skewness and as a result we will report the treatment effect as a geometric mean ratio.

The repeated time point binary outcomes ID and IDA will be analysed similarly to anaemia for the ITT population. We will extract the treatment effect from this same model at 28 days follow-up, 36 weeks' gestation, and 28 days postpartum.

In addition to the analysis described above, we will perform:

- The three adjusted analyses described in Section 3.5.1.1
- The model described above on the PP population
- The model described above on the PP population adjusted for baseline characteristics considered not balanced between the arms for the PP population

7.2 NEONATE/INFANT STUDY VARIABLES

7.2.1 Birthweight

Birthweight will be analysed by fitting a linear regression model for the ITT population. We will examine the assumptions underlying the model, including normally distributed error terms, homoscedasticity, and influential observations. We will report the absolute difference in mean birth weight between IV iron and standard-of-care (oral iron) along with a corresponding two-sided 95% confidence interval and P-Value.
In addition to the analysis described above, we will perform:

- The three adjusted analyses described in Section 3.5.1.2
- The missing data additional analyses described in Section 5.4.2
- The model described above on the PP population
- The model described above on the PP population adjusted for baseline characteristics considered not balanced between the arms for the PP population

7.2.2 Secondary Neonate/Infant Analyses

The single time point continuous outcomes gestation duration, birth length, haemoglobin concentration, weight, length, length-for-age z-score, weight-for-age z-score, weight-for-length z-score will be analysed similarly to birthweight for the ITT population. Appropriate transformations may be applied to the variables before fitting the model if considered skewed.
The single time point binary outcome of the composite adverse birth outcome, low birthweight, pregnancy/stillbirth, premature birth, and small for gestational age will be analysed using a log-binomial regression model for the ITT population. We will report the estimate of the risk ratio of IV iron versus standard-of-care (oral iron), two-sided 95% confidence interval and P-Value. In case of non-convergence, we will fit a modified Poisson regression model with robust error variance.
In addition to the analysis described above, we will perform:

- The three adjusted analyses described in Section 3.5.1.2
- The model described above on the PP population
- The model described above on the PP population adjusted for baseline characteristics considered not balanced between the arms for the PP population

Furthermore, we will report the number-needed-to-treat (NNT) and 95% confidence interval for low birthweight.

8 Safety Analyses

For all safety analyses, we will use the following principles:

- We will use the safety and modified safety populations for all safety analyses
- Comparisons of interest are IV iron versus standard-of-care (oral iron)
- All tests will be two-sided at the 5% level of significance
- All confidence intervals will be two-sided 95%
- All analyses will be adjusted for the stratification variable used during the randomisation (site), referred to as the unadjusted analyses.
- A complete case analysis will be used for all outcomes.

8.1 (Serious) Adverse Events

(Serious) adverse event (SAEs) were collected at any time after randomisation.
Adverse events (AEs) will be coded using version 5.0 of the Common Terminology Criteria for Adverse Events (CTCAE) (US Department of Health Human Services, 27 November 2020) and use an appropriate Preferred Term (PT) and System Organ Class (SOC) for each AE verbatim term. AEs will be coded as one AE per presentation, unless clearly unrelated. We will report the most 'upstream' condition: Fetal loss (pregnancy loss before 22 completed weeks' gestation), stillbirth (pregnancy loss after 22 weeks and prior to delivery with no

evidence of life at delivery) and neonatal death (death in the first 28 days of life of a child with evidence of life following delivery). Where the underlying cause is not discernible, AEs will be coded as pregnancy loss and neonatal death where appropriate. All AEs were coded on an ongoing basis by two clinicians – blinded to each other's decisions – and without knowledge of the treatment-code. Discrepancies regarding the selection of an appropriate PT or SOC will be resolved via a discussion between the two clinicians. All AE coding will be finalised before unblinding of the study database.

8.1.1 Maternal

We will report the following for women:

- n (%) of women with at least one AE (overall, and for each of the following time intervals: within 24 hours of randomisation, within 14 days of randomisation, antenatal, postpartum)
- n (%) of women with at least one SAE (overall, and for each of the following time intervals: within 24 hours of randomisation, within 14 days of randomisation, antenatal, postpartum)
- n (%) of women who died
- number of AE
- number of SAE
- n (%) of women for the composite severe medical events including its components haemorrhage, need for transfusion, ICU admission, or mortality
- n (%) of women with at least one common AE (>5% in any group)
- n (%) of women with at least one AE by system organ class
- n (%) of women with at least one AE by preferred term

Except for number of AE/SAE, each of the above listed adverse event outcomes will be compared between treatment arms using a log-binomial regression model. We will report the estimate of the risk ratio of IV iron versus standard-of-care (oral iron), two-sided 95% confidence interval and P-Value. In case of non-convergence, we will fit a modified Poisson regression model with robust error variance.

In addition, we will report infusion-related AEs separately for the IV iron group only:

- n (%) of women with at least one treatment-related AE (including immediately [while observed during IV], 14 days post-IV).
- number of treatment-related AE.
- n (%) of women with at least one headache, dizziness, dysgeusia, discolouration of the skin, nausea, vomiting, constipation, upper abdominal pain, dyspepsia, anaphylactic shock, flushing, shortness of breath, chest pains, other.

8.1.2 Neonates/Infants

We will report the following for neonates/infants:

- n (%) of neonates/infants with at least one AE
- n (%) of neonates/infants with at least one SAE
- n (%) of neonates/infants who died
- number of AE
- number of SAE
- n (%) of neonates/infants with at least on common AE (>5% in any group)

- n (%) of neonates/infants with at least one AE by system organ class
- n (%) of neonates/infants with at least one AE by preferred term

Except for number of AE/SAE, these outcomes will be analysed using a log-binomial regression model. In case of non-convergence, we will fit a modified Poisson regression model with robust error variance. Prevalence ratios and 95% confidence intervals will be presented for IV iron versus standard-of-care (oral iron).

8.2 UNPLANNED CLINIC VISITS

8.2.1 Maternal

We will report the n (%) of women with at least one:

- All cause sick visit (antenatal, postpartum)
- Clinical malaria-related visit (antenatal, postpartum)

These outcomes will be analysed using a log-binomial regression model. We will report the estimate of the risk ratio of IV iron versus standard-of-care (oral iron), two-sided 95% confidence interval and P-Value. In case of non-convergence, we will fit a modified Poisson regression model with robust error variance.

8.2.2 Neonates/infants

We will report the n (%) of neonates/infants with at least one:

- All cause sick visit (including infection)
- Diarrhoea-related visit
- Respiratory-related visit
- Clinical malaria-related visit

  This will be analysed using a log-binomial regression model. We will report the estimate of the risk ratio of IV iron versus standard-of-care (oral iron), two-sided 95% confidence interval and P-Value. In case of non-convergence, we will fit a modified Poisson regression model with robust error variance.

8.3 SAFETY BIOMARKERS/CLINICAL LABORATORY EVALUATIONS

8.3.1 Maternal

Hypophosphatemia will be derived from phosphate (PO4) (22, 23) as:

- Mild: 0.64 mmol/L < PO4 <0.80 mmol/L
- Moderate: 0.32 mmol/L < PO4 <0.64 mmol/L
- Severe: PO4 < 0.32 mmol/L

Hypophosphatemia will be analysed by time-point (28 days follow-up, 36 weeks' gestation) using an ordered logistic regression model. We will report the estimate of the odds ratio of IV iron versus standard-of-care (oral iron), two-sided 95% confidence interval and P-Value.
Inflammation and malaria RDT positive will be analysed by time-point (28 days follow-up, 36 weeks' gestation) using a log-binomial regression model. We will report the estimate of the risk ratio of IV iron versus standard-of-care (oral iron), two-sided 95% confidence interval and P-Value. In case of non-convergence, we will fit a modified Poisson regression model with robust error variance.

8.3.2 Neonates/infants

Not applicable.

9 Impact of COVID-19 Pandemic

The oral iron was given under real-life health service delivery conditions. This strategy was employed as it is a key hypothesis in this trial that there is a reduced effect of oral iron on maternal anaemia due to poor adherence to the full course of treatment. The original protocol included second-weekly home visits from 34 weeks' gestational age by a research nurse during which the participant was to be asked for the tablet pack and the nurse was to count the number the participant had left in order to determine adherence to iron in the oral iron arm. Capillary haemoglobin concentration levels at these timepoints was also to be collected. Notably, since the opening of the trial, fieldwork has been affected by the COVID-19 pandemic. Because of the risks of study workers introducing COVID-19 into remote villages, home visits were removed from the trial protocol after April 2020 and thus no data on pill adherence was collected. Likewise, capillary haemoglobin concentration at these home visit timepoints was not collected.

10 Technical Details

Analysis will be conducted using Stata/SE for Windows version 17.0 (64-bit x86-64) or higher. We will report the software and version used at the time of reporting.

11 Validation of Output

The analysis of the maternal anaemia and birthweight will be checked by a senior statistician. Discrepancies will be discussed and resolved by consensus. All other analyses will be conducted by the trial statistician only and reviewed by a senior statistician.

12 Summary of Changes to the Registry and Protocol

This statistical analysis plan outlined the details of the analyses beyond what was specified in the registry.

Compared to the registry, the following was changed:

- The registry states that the primary maternal outcome anaemia at pre-delivery (based on the capillary Hb closest prior to delivery) will be analysed using a binomial log-linear regression. Instead, the primary maternal outcome of anaemia at 36 weeks' gestation (measured on venous blood) will be analysed using a log-binomial regression model. This change was made due to the suspension of home visits due to the. COVID-19 pandemic. Home visit-collected capillary data will not be included in the analysis.
- The registry states that multiple imputation under the missing at random assumption will be performed as a secondary analysis for handling missing data in the primary maternal and neonate outcome. Instead, multiple imputation under the missing not at random assumption (MNAR) will be performed as an additional analysis for handling missing data in the primary maternal outcome because the primary analysis model assumes MAR.

Compared to the protocol (version 5.0), the following was changed:

- The protocol states that the primary maternal outcome anaemia at pre-delivery will be analysed using a binomial log-linear regression. Instead, the primary maternal outcome anaemia at 36 weeks' gestation will be analysed using a log-binomial regression model.
- The protocol states that multiple imputation under the missing at random assumption will be performed as a secondary analysis for handling missing data in the primary maternal and neonate outcome. Instead, multiple imputation under the missing not at random assumption (MNAR) will be performed as an additional analysis for handling

missing data in the primary maternal outcome because the primary analysis model assumes MAR.

13 Summary of Changes Between Versions

- Added a reference for the cut-off point for the primary outcome of maternal anaemia and clarified its definition across different timepoints
- Added a reference for the cut-off points for hypophosphatemia
- Added secondary neonatal continuous outcomes for weight and length at 1 month postpartum to complement the infant growth z-scores
- Added that sample size calculations were performed using Stata software
- Clarified primary analyses will be undertaken on an intention-to-treat (ITT) basis and the definition of the ITT and per-protocol populations throughout
- Added a modified safety population definition excluding women who did not have a singleton pregnancy
- Clarified the model for the primary and secondary repeated timepoint binary outcomes, added the measure of effect for a modified Poisson regression model and added insight into why we selected a log-binomial model rather than a logistic regression model
- Clarified the description for the reporting and analysis of adverse events
- Clarified we will refer to the analysis as a modified ITT analysis if not all randomised participants will have data contributing to the analysis due to missing data
- Clarified about signing of the final version of SAP
- Revised the CONSORT flow chart footnotes to indicate those who were included in the ITT population
- Removal of section Extent of exposure section as a version of this will be captured within the CONSORT flow chart
- Removal of the terms sepsis and shock form the in maternal safety section defining composite severe medical events as an oversight from
- Missing data section was updated following blinded data review discussions

14 References

1. Source of SAP template: Cambridge University Hospitals – Clinical Trials Unit https://www.cuh.nhs.uk/document-library/cctu-standard-operating-procedures-sops.
2. World Health Organization. The global prevalence of anaemia in 2011. . Geneva: 2015.
3. Haider BA, Olofin I, Wang M, Spiegelman D, Ezzati M, Fawzi WW. Anaemia, prenatal iron use, and risk of adverse pregnancy outcomes: Systematic review and meta-analysis. BMJ (Online). 2013;347(7916). doi: 10.1136/bmj.f3443.
4. Pasricha SR, Tye-Din J, Muckenthaler MU, Swinkels DW. Iron deficiency. Lancet. 2021;397(10270):233-48. Epub 2020/12/08. doi: 10.1016/s0140-6736(20)32594-0. PubMed PMID: 33285139.
5. Premru-Srsen T, Verdenik I, Ponikvar BM, Steblovnik L, Geršak K, Cerar LK. Infant mortality and causes of death by birth weight for gestational age in non-malformed singleton infants: a 2002-2012 population-based study. J Perinat Med. 2018;46(5):547-53. Epub 2017/06/11. doi: 10.1515/jpm-2017-0103. PubMed PMID: 28599397.
6. World Health Organization, United Nations Children's Fund, United Nations University. Iron Deficiency Anaemia: Assessment, Prevention, and Control. A guide for programme managers. Geneva: 2001.
7. World Health Organization. Global Nutrition Targets 2025: Anaemia Policy Brief. . Geneva: 2014.
8. Low MSY, Speedy J, Styles CE, De-Regil LM, Pasricha SR. Daily iron supplementation for improving anaemia, iron status and health in menstruating women. Cochrane Database of Systematic Reviews. 2016;2016(4). doi: 10.1002/14651858.CD009747.pub2.
9. Medicinewise N. Ferinject - Ferric carboxymaltose. Consumer medicine information (CMI) leaflet: NPS Medicinewise; 2019 [cited 2019 14 Oct]. Available from: https://www.nps.org.au/medicine-finder/ferinject-solution-for-injection.
10. Mwangi MNM, G., Moya E, Braat S, Harding R, Robberstad B, Simpson JA, et al. Protocol for a multicentre, parallel-group, open-label randomised controlled trial comparing ferric carboxymaltose with the standard of care in anaemic Malawian pregnant women: the REVAMP trial. BMJ Open. 2021;0:e053288. doi: doi:10.1136/bmjopen-2021-053288.
11. World Health Organization. Haemoglobin concentrations for the diagnosis of anaemia and assessment of severity. Vitamin and Mineral Nutrition Information System. Geneva, World Health Organization, 2011 (WHO/NMH/NHD/MNM/11.1) 2011 [cited 2022 17 Feburary]. Available from: https://www.who.int/vmnis/indicators/haemoglobin.pdf.
12. Villar J, Cheikh Ismail L, Victora CG, Ohuma EO, Bertino E, Altman DG, et al. International standards for newborn weight, length, and head circumference by gestational age and sex: the Newborn Cross-Sectional Study of the INTERGROWTH-21st Project. Lancet. 2014;384(9946):857-68. Epub 2014/09/12. doi: 10.1016/s0140-6736(14)60932-6. PubMed PMID: 25209487.
13. World Health Organization. WHO child growth standards: length/height-for-age, weight-for-age, weight-for-length, weight-for-height and body mass index-for-age: methods and development. 2006.
14. Bah A, Pasricha SR, Jallow MW, Sise EA, Wegmuller R, Armitage AE, et al. Serum hepcidin concentrations decline during pregnancy and may identify iron deficiency: Analysis of a longitudinal pregnancy cohort in the Gambia. Journal of Nutrition. 2017;147(6):1131-7. doi: 10.3945/jn.116.245373.
15. Breymann C, Milman N, Mezzacasa A, Bernard R, Dudenhausen J, investigators F-A. Ferric carboxymaltose vs. oral iron in the treatment of pregnant women with iron deficiency anemia: an international, open-label, randomized controlled trial (FER-ASAP). J Perinat Med. 2017;45(4):443-53. Epub 2016/06/10. doi: 10.1515/jpm-2016-0050. PubMed PMID: 27278921.
16. Mwangi MN, Roth JM, Smit MR, Trijsburg L, Mwangi AM, Demir AY, et al. Effect of daily antenatal iron supplementation on plasmodium infection in kenyan women: A randomized clinical

trial. JAMA - Journal of the American Medical Association. 2015;314(10):1009-20. doi: 10.1001/jama.2015.9496.
17. Jakobsen JC, Gluud C, Wetterslev J, Winkel P. When and how should multiple imputation be used for handling missing data in randomised clinical trials – a practical guide with flowcharts. BMC Medical Research Methodology. 2017;17(1):162. doi: 10.1186/s12874-017-0442-1.
18. Huque MH, Carlin JB, Simpson JA, Lee KJ. A comparison of multiple imputation methods for missing data in longitudinal studies. BMC Med Res Methodol. 2018;18(1):168. Epub 2018/12/14. doi: 10.1186/s12874-018-0615-6. PubMed PMID: 30541455; PubMed Central PMCID: PMC6292063.
19. Rezvan PH, Lee K, Simpson JA. Sensitivity analysis within multiple imputation framework using delta-adjustment: Application to Longitudinal Study of Australian Children. Longitudinal and life course studies. 2018;9:259-78.
20. Holm S. A Simple Sequentially Rejective Multiple Test Procedure. Scandinavian Journal of Statistics. 1979;6(2):65-70.
21. Liang K-Y, Zeger SL. Longitudinal data analysis of continuous and discrete responses for pre-post designs. Sankhyā: The Indian Journal of Statistics, Series B. 2000:134-48.
22. Koyfman A. Hypophosphatemia in Emergency Medicine. MedScape. 2021.
23. Yu AS, JR S. Hypophosphatemia: Clinical manifestations of phosphate depletion. UpToDate2022.

15 Listing of Key Tables, Listings and Figures

Number	Title	Population
Table 1	Characteristics at enrolment	ITT population – mothers
Table 2	Maternal outcomes	ITT population – mothers
Table 3	Neonate/infant outcomes	ITT population – neonates/infants
Table 4	Maternal adverse events	Safety population – mothers
Table 5	Infusion-related adverse events	Safety population – mothers
Table 6	Maternal unplanned clinic visits	Safety population – mothers
Table 7	Maternal safety biomarkers	Safety population – mothers
Table 8	Neonate/Infant adverse events	Safety population –neonates/infants neonateneonates/infants neonates/infants
Table 9	Neonate/Infant unplanned clinic visits	Safety population –neonates/infants neneonates/infants
Figure 1	CONSORT flow chart	All screened mothers
Figure 2	Estimate and 95% confidence interval of haemoglobin and anaemia over time	ITT population – mothers
Figure(s) 3a	Forest plots for subgroup analyses of maternal outcomes	ITT population – mothers
Figure(s) 3b	Forest plots for subgroup analyses of neonate/infant outcomes	ITT population – neonates/infants

Table 1 – Maternal characteristics at Enrolment *

	IV Iron<br>N = XXX	Standard of care – Oral Iron<br>N = XXX
Age (years)	X.X ± X.X	X.X ± X.X
Primiparous, no. (%)‡	n (%)	n (%)
Primigravid, no. (%)‡	n (%)	n (%)
Gestational age (weeks), mean(SD)	X.X ± X.X	X.X ± X.X
Height (cm)	X.X ± X.X	X.X ± X.X
Weight (kg)	X.X ± X.X	X.X ± X.X
Body mass index (kg/m^2) †	X.X ± X.X	X.X ± X.X
Religion, no. (%)‡		
None	n (%)	n (%)
Christian	n (%)	n (%)
Muslim	n (%)	n (%)
Other	n (%)	n (%)
Education, no. (%)‡		
None	n (%)	n (%)
Lower Primary (1-5)	n (%)	n (%)
Upper Primary (6-8)	n (%)	n (%)
Lower Secondary (1-2)	n (%)	n (%)
Upper Secondary (3-4)	n (%)	n (%)
Tertiary	n (%)	n (%)
Marital status, no. (%)‡		
Single	n (%)	n (%)
Married	n (%)	n (%)
Widowed	n (%)	n (%)
Divorced/Separated	n (%)	n (%)
Other	n (%)	n (%)
Income source, no. (%)‡		
None	n (%)	n (%)
Subsistence farming	n (%)	n (%)
Large scale farming	n (%)	n (%)
Employed	n (%)	n (%)
Casual work for wages	n (%)	n (%)
Business	n (%)	n (%)
Other	n (%)	n (%)
Re-screened post positive malaria RDT, no. (%)‡‡	n (%)	n (%)
HIV positive, no. (%)‡	n (%)	n (%)
Malaria RDT positive, no. (%)	n (%)	n (%)
Capillary Hb<10 g/dL, no. (%)	n (%)	n (%)
Venous Hb, mean (SD)	X.X ± X.X	X.X ± X.X
Anaemia (venous), no. (%)§		
Mild	n (%)	n (%)
Moderate	n (%)	n (%)
Severe	n (%)	n (%)
Ferritin, median (IQR)	X.X (X.X – X.X)	X.X (X.X – X.X)
C-reactive protein, median (IQR)	X.X (X.X – X.X)	X.X (X.X – X.X)
Iron deficient, no. (%)¶	n (%)	n (%)
Iron deficient anaemia, no. (%)¶	n (%)	n (%)
Inflammation, no. (%) ‖	n (%)	n (%)
Anaemia and inflammation, no. (%) ‖	n (%)	n (%)

*Plus-minus values are means ±SD. Participants were enrolled across two sites, X = Blantyre and X=Zomba
Hb denotes haemoglobin, HIV denotes human immunodeficiency virus, IQR inter-quartile range (25th to 75th percentile), and SD standard deviation.

† Body mass index is the weight in kilograms divided by the square of the height in meters.
‡ Religion, Education, Marital status, Income source, parity, gravidity and HIV status were self reported.
§ Mild anaemia indicates 10g/dL>Hb<11g/dL, moderate anaemia indicates 7g/dL≤Hb<10g/dL, and severe anaemia indicates Hb<7g/dL.
¶ Iron deficient indicates serum ferritin<15 ug/L or ferritin <30 ug/L if C-reactive protein >5 µg/L, and iron deficient anaemia indicates Hb<11g/dL and serum ferritin<15 ug/L or ferritin <30 ug/L if C-reactive protein >5 µg/L
|| Inflammation indicates C-reactive protein >5µg/L, and anaemia and inflammation indicates Hb<11.0g.dL and C-reactive protein >5µg/L
‡‡ women who were enrolled after an initial pre-screening positive malaria test, re-screened for malaria a week later and if negative then enrolled (with no other exclusion criteria present)

Table 2 – Primary and Secondary Maternal endpoints*

	IV Iron N = XXX	Standard of care – Oral Iron N = XXX	Prevalence Ratio or Mean Difference or Geometric Mean Ratio (95% CI) †	P-value‡
**Primary maternal outcome**				
Anaemia at 36 weeks' gestation §‡‡	n (%)	n (%)	X.X (X.X – X.X)	X.XXX
**Secondary maternal outcomes**				
Moderate/Severe anaemia at 36 weeks' gestation §	n (%)	n (%)	X.X (X.X – X.X)	X.XXX**
Venous Hb (g/dL) change from baseline at 36 weeks' gestation	X.X ± X.X	X.X ± X.X	X.X (X.X – X.X)	X.XXX**
Ferritin (µg/L) change from baseline at 36 weeks' gestation	X.X (X.X – X.X)	X.X (X.X – X.X)	X.X (X.X – X.X)	X.XXX**
Iron deficiency at 36 weeks gestation¶	n (%)	n (%)	X.X (X.X – X.X)	X.XXX**
Iron deficient anaemia at 36 weeks gestation¶	n (%)	n (%)	X.X (X.X – X.X)	X.XXX**
**Other secondary maternal outcomes**				
Anaemia §				
28 days post randomisation	n (%)	n (%)	X.X (X.X – X.X)	
Delivery	n (%)	n (%)	X.X (X.X – X.X)	
28 days postpartum	n (%)	n (%)	X.X (X.X – X.X)	
Moderate/Severe anaemia §				
28 days post randomisation	n (%)	n (%)	X.X (X.X – X.X)	
Delivery	n (%)	n (%)	X.X (X.X – X.X)	
28 days postpartum	n (%)	n (%)	X.X (X.X – X.X)	
Venous Hb (g/dL) change from baseline				
28 days post randomisation	X.X ± X.X	X.X ± X.X	X.X (X.X – X.X)	
Delivery	X.X ± X.X	X.X ± X.X	X.X (X.X – X.X)	
28 days postpartum	X.X ± X.X	X.X ± X.X	X.X (X.X – X.X)	
Ferritin (µg/L) change from baseline to baseline				
28 days post randomisation	X.X (X.X – X.X)	X.X (X.X – X.X)	X.X (X.X – X.X)	
28 days postpartum	X.X (X.X – X.X)	X.X (X.X – X.X)	X.X (X.X – X.X)	
Iron deficiency ¶				
28 days post randomisation	n (%)	n (%)	X.X (X.X – X.X)	
28 days postpartum	n (%)	n (%)	X.X (X.X – X.X)	
Iron deficient anaemia ¶				
28 days post randomisation	n (%)	n (%)	X.X (X.X – X.X)	
28 days postpartum	n (%)	n (%)	X.X (X.X – X.X)	

* Plus-minus values are means ±SD. CI denotes confidence interval, ID iron deficiency, and IDA iron deficieny anaemia.

§ Anaemia indicates Hb<11.0 g/dL up to and including delivery and Hb<12.0 g/dL postpartum. Moderate/Severe anaemia indicates Hb<10g/dL up to and including delivery and Hb<11.0 g/dL postpartum.

¶ Iron deficient indicates serum ferritin<15 ug/L or ferritin <30 ug/L if C-reactive protein >5 µg/L, and iron deficient anaemia indicates Hb<11g/dL and serum ferritin<15 ug/L or ferritin <30 ug/L if C-reactive protein >5 µg/L

† A prevalence ratio of IV iron versus standard-of-care (oral iron) is displayed for anaemia, ID and IDA and risk ratio for moderate/severe anaemia at 36 weeks gestation, 28 days post randomisation, delivery (for anaemia and moderate/severe anaemia) and 28 days postpartum

following analyses using a log-binomial regression model, including random effect for participant. A mean difference of IV iron versus standard-of-care (oral iron) of the estimated change from baseline to at 36 weeks gestation, 28 days post randomisation, delivery (for continuous haemoglobin) and 28 days postpartum respectively is displayed for continuous haemoglobin and ferritin concentration following analyses using a likelihood-based longitudinal data analysis model. A geometric mean ratio is displayed for ferritin concentration after a log base e transformation due to skewnesss.
‡ The P-values and 95% confidence intervals presented have not been adjusted for multiple comparisions.
** The P-value for X and Y remained significant after controlling for multiple comparsions with the Holm procedure.
‡‡Number needed to treat (NNT) for anaemia at 36 weeks' gestation is …

Table 3 – Primary and Secondary Neonate endpoints*

	IV Iron Number (percent)	IV Iron Mean ± SD	Standard of care – Oral Iron Number (percent)	Standard of care – Oral Iron Mean ± SD	Prevalence ratio or Mean Difference (95% CI) †	P-value‡
**Key neonatal outcome**						
Birthweight (grams)	n (%)	X.X ± X.X	n (%)	X.X ± X.X	X.X (X.X – X.X)	X.XXX
**Secondary neonatal outcomes**						
Delivery						
Gestation duration	n (%)	X.X ± X.X	n (%)	X.X ± X.X	X.X (X.X – X.X)	X.XXX**
Composite adverse birth outcome	n (%)		n (%)	N/A	X.X (X.X – X.X)	X.XXX**
Low birthweight (<2500g) ‡‡	n (%)	N/A	n (%)	N/A	X.X (X.X – X.X)	
Pregnancy loss or stillbirth	n (%)	N/A	n (%)	N/A	X.X (X.X – X.X)	
Premature birth (<37weeks GA)	n (%)	N/A	n (%)	N/A	X.X (X.X – X.X)	
Small for GA	n (%)	N/A	n (%)	N/A	X.X (X.X – X.X)	
28 days postpartum						
Venous Hb (g/dL)	n (%)	X.X ± X.X	n (%)	X.X ± X.X	X.X (X.X – X.X)	X.XXX**
Weight (grams) §	n (%)	X.X ± X.X	n (%)	X.X ± X.X	X.X (X.X – X.X)	X.XXX**
**Other neonatal outcomes**						
Delivery						
Birthlength (cm)	n (%)	X.X ± X.X	n (%)	X.X ± X.X	X.X (X.X – X.X)	
28 days postpartum						
Length (cm) §	n (%)	X.X ± X.X	n (%)	X.X ± X.X	X.X (X.X – X.X)	
Weight for age z score§	n (%)	X.X ± X.X	n (%)	X.X ± X.X	X.X (X.X – X.X)	
Length for age z score§	n (%)	X.X ± X.X	n (%)	X.X ± X.X	X.X (X.X – X.X)	
Weight for length z score§	n (%)	X.X ± X.X	n (%)	X.X ± X.X	X.X (X.X – X.X)	

* Plus-minus values are means ±SD. CI denotes confidence interval. GA denotes gestational age.

† An absolute mean difference for birthweight, gestation duration, and birth length at deliviery, and haemoglobin concentration, length-for-age z-score, weight-for-age z-score, ans weight-for-length z-score at 28 days postpartum between IV iron and standard-of-care (oral iron) is displayed following fitting a linear regression model. A risk ratio of IV iron versus standard of care is displayed for the composite adverse birth outcome, low birthweight, pregnancy/stillbirth, premature birth, and small for gestational age following analyses using a log-binomial regression model.

§ Total number of neonates reduced due to procedural missing data (version control)

‡ The P-values and 95% confidence intervals presented have not been adjusted for multiple comparisions.
** The P-value for X and Y remained significant after controlling for multiple comparsions with the Holm procedure.
‡‡ Number needed to treat (NNT) for low birthweight is …

Table 4 – Maternal Adverse events *

	IV Iron<br>N = XXX	Standard of care – Oral Iron<br>N = XXX	Risk Ratio (95% CI)†	P-value‡
**Summary of subjects with AEs**				
**At least one AE**	n (%)	n (%)	X.X (X.X – X.X)	X.XXX
Within 24 hours of randomisation	n (%)	n (%)	X.X (X.X – X.X)	X.XXX
Within 14 days of randomisation	n (%)	n (%)	X.X (X.X – X.X)	X.XXX
Antenatal (prior to the 3rd stage of labour)	n (%)	n (%)	X.X (X.X – X.X)	X.XXX
Postpartum	n (%)	n (%)	X.X (X.X – X.X)	X.XXX
**At least one SAE**	n (%)	n (%)	X.X (X.X – X.X)	X.XXX
Within 24 hours of randomisation	n (%)	n (%)	X.X (X.X – X.X)	X.XXX
Within 14 days of randomisation	n (%)	n (%)	X.X (X.X – X.X)	X.XXX
Antenatal (prior to the 3rd stage of labour)	n (%)	n (%)	X.X (X.X – X.X)	X.XXX
Postpartum	n (%)	n (%)	X.X (X.X – X.X)	X.XXX
**Death**	n (%)	n (%)		
**Summary of number of AEs**				
**AE**	n	n		N/A
**SAE**	n	n		N/A
**Subjects with at least one AE of special interest**				
Composite Severe medical event§‡‡	n (%)	n (%)	X.X (X.X – X.X)	X.XXX
Death	n (%)	n (%)	X.X (X.X – X.X)	X.XXX
Haemorrhage	n (%)	n (%)	X.X (X.X – X.X)	X.XXX
Blood transfusion¶	n (%)	n (%)	X.X (X.X – X.X)	X.XXX
ICU care	n (%)	n (%)	X.X (X.X – X.X)	X.XXX
**Subjects with common AEs occuring in >5% in any treatment group**				
E.g. Headache	n (%)	n (%)	X.X (X.X – X.X)	X.XXX
<data driven>				
…				
**Subjects with at least one AE by SOC**				
Infections and infestations	n (%)	n (%)	X.X (X.X – X.X)	X.XXX
Congenital, familial and genetic disorders	n (%)	n (%)	X.X (X.X – X.X)	X.XXX
Nervous system disorders	n (%)	n (%)	X.X (X.X – X.X)	X.XXX
Musculoskeletal and connective tissue disorders	n (%)	n (%)	X.X (X.X – X.X)	X.XXX
Vascular disorders	n (%)	n (%)	X.X (X.X – X.X)	X.XXX
Respiratory, thoracic and mediastinal disorders	n (%)	n (%)	X.X (X.X – X.X)	X.XXX
Gastrointestinal disorders	n (%)	n (%)	X.X (X.X – X.X)	X.XXX
Pregnancy, puerperium and perinatal conditions	n (%)	n (%)	X.X (X.X – X.X)	X.XXX
Ear and labyrinth disorders	n (%)	n (%)	X.X (X.X – X.X)	X.XXX
Blood and lymphatic disorders	n (%)	n (%)	X.X (X.X – X.X)	X.XXX
Skin and subcutaneous disorders	n (%)	n (%)	X.X (X.X – X.X)	X.XXX
General disorders and administration site conditions	n (%)	n (%)	X.X (X.X – X.X)	X.XXX
Cardiac disorders	n (%)	n (%)	X.X (X.X – X.X)	X.XXX
Neoplasms benign, malignant and unspecified (incl cysts and polyps)	n (%)	n (%)	X.X (X.X – X.X)	X.XXX
Injury, poisoning and procedural complications	n (%)	n (%)	X.X (X.X – X.X)	X.XXX
Reproductive system and breast disorders	n (%)	n (%)	X.X (X.X – X.X)	X.XXX

	IV Iron<br>N = XXX	Standard of care – Oral Iron<br>N = XXX	Risk Ratio (95% CI)†	P-value‡
…				
**Subjects with at least one AE by SOC preferred term**				
E.g. Headache	n (%)	n (%)	X.X (X.X – X.X)	X.XXX
….				

*CI denotes confidence interval, AE Adverse Event, SAE Serious Adverse Event, SOC System Organ Class, ICU Itensive Care Unit.
†Except for number of AE/SAE, these outcomes are analysed using a log-binomial regression model and display the estimate of the risk ratio of IV iron versus standard-of-care (oral iron) and 95% CI and P-Value.
‡ Unadjusted P-values and 95% confidence intervals are presented here.
§ The composite severe medical event outcome was at least one severe medical event of dealth, haemorrhage, blood transfusion or ICU care recorded in the mother. Death, haemorrhage, blood transfusion and admission to ICU are captured across the whole trial period.
¶Blood transfusion denotes that a blood tansfusion was required by the mother.
ICU care denotes that ICU care was required by the mother.
‡‡ Number needed to harm (NNH) for the composite sereve medical event is …

Table 5 – Maternal Infusion related Adverse events *

	IV Iron<br>N = XXX	
**Summary of subjects with infusion related AEs**		
**At least one infusion related adverse event**		
Immediately (while observed) †	n (%)	X.X – X.X
14 days post infusion ‡	n (%)	X.X – X.X
**Summary of number of infusion related AEs**		
**Infusion related AE**	n	n
**Summary of infusion related AEs by characteristics of treatment** † §		
Headache	n (%)	X.X – X.X
Dizziness	n (%)	X.X – X.X
Dysgeusia	n (%)	X.X – X.X
Discolouration of the skin	n (%)	X.X – X.X
Nausea	n (%)	X.X – X.X
Vomiting	n (%)	X.X – X.X
Constipation	n (%)	X.X – X.X
Upper abdominal pain	n (%)	X.X – X.X
Dyspepsia	n (%)	X.X – X.X
Anaphylaxis	n (%)	X.X – X.X
Flushing	n (%)	X.X – X.X
Shortness of breath	n (%)	X.X – X.X
Chest pains	n (%)	X.X – X.X
Other	n (%)	X.X – X.X
None	n (%)	X.X – X.X

*reported for IV iron group only. AE denotes Adverse Event

†AEs as captured on the participant randomisation form at the time IV iron given

‡AEs as captured on the AE form within 14 days of when IV iron was received.

§Events sorted according to frequency from high to low.

Table 6 – Maternal Unplanned Clinic visits

	IV Iron N = XXX	Standard of care – Oral Iron N = XXX	Risk Ratio (95% CI)†	P-value‡
**All cause sick visits**				
Antenatal	n (%)	n (%)	X.X (X.X – X.X)	X.XXX
Postpartum	n (%)	n (%)	X.X (X.X – X.X)	X.XXX
**Clinical malaria-related visits**				
Antenatal	n (%)	n (%)	X.X (X.X – X.X)	X.XXX
Postpartum	n (%)	n (%)	X.X (X.X – X.X)	X.XXX

CI denotes confidence interval

†These outcomes were analysed using a log-binomial regression model and display the estimate of the risk ratio of IV iron versus standard-of-care (oral iron) and 95% CI and P-Value.

‡ Unadjusted P-values and 95% confidence intervals are presented here.

Table 7 – Maternal Safety Biomarkers

	IV Iron N = XXX	Standard of care – Oral Iron N = XXX	Risk Ratio (95% CI)	P-value‡
**Hypophosphatemia ***				
4 weeks post randomisation				
Mild	n (%)	n (%)	X.X (X.X – X.X)	X.XXX
Moderate	n (%)	n (%)	-	-
Severe	n (%)	n (%)	-	-
36 weeks' gestation				
Mild	n (%)	n (%)	X.X (X.X – X.X)	X.XXX
Moderate	n (%)	n (%)	-	-
Severe	n (%)	n (%)	-	-
**Inflammation (elevated CRP) †**				
4 weeks post randomisation	n (%)	n (%)	X.X (X.X – X.X)	X.XXX
36 weeks' gestation	n (%)	n (%)	X.X (X.X – X.X)	X.XXX
**Malaria RDT positive†**				
4 weeks post randomisation	n (%)	n (%)	X.X (X.X – X.X)	X.XXX
36 weeks' gestation	n (%)	n (%)	X.X (X.X – X.X)	X.XXX

CI denotes confidence interval, CRP C-reactive protein, RDT rapid diagnostic test

* Mild hypophosphatemia is defined as (0.64<PO4 <0.80 mmol/L), Moderate hypophosphatemia is defined as (0.32<PO4<0.64 mmol/L) and severe hypophosphatemia is defined as (PO4 (<0.32 mmol/L). Hypophosphatemia is analysed by time-point (28 days follow-up, 36 weeks' gestation) using ordered logistic regression.

†These outcomes are analysed by time point (28 days follow-up, 36 weeks' gestation) using a log-binomial regression model and display the estimate of the risk ratio of IV iron versus standard-of-care (oral iron) and 95% CI and P-Value.

‡ Unadjusted P-values and 95% confidence intervals are presented here.

Table 8 – Neonate Adverse events*

	IV Iron N = XXX	Standard of care – Oral Iron N = XXX	Risk Ratio (95% CI)†	P-value‡
**Summary of subjects AEs**				
**At least one AE**	n (%)	n (%)	X.X (X.X – X.X)	X.XXX
**At least one SAE**	n (%)	n (%)	X.X (X.X – X.X)	X.XXX
**Death**	n (%)	n (%)	X.X (X.X – X.X)	X.XXX
**Death & pregnancy loss**	n (%)	n (%)	X.X (X.X – X.X)	X.XXX
**Summary of number of AEs**				
**AE**	n	n		N/A
**SAE**	n	n		N/A
**Subjects with common AEs occuring in >5% in any treatment group**				
E.g. Infection	n (%)	n (%)	X.X (X.X – X.X)	X.XXX
<data driven>				
**Subjects with at least one AE by SOC**				
General disorders and administration site conditions	n (%)	n (%)	X.X (X.X – X.X)	X.XXX
Pregnancy, puerperium and perinatal conditions	n (%)	n (%)	X.X (X.X – X.X)	X.XXX
Congenital, familial and genetic disorders	n (%)	n (%)	X.X (X.X – X.X)	X.XXX
Infections and Infestations	n (%)	n (%)	X.X (X.X – X.X)	X.XXX
Respiratory, thoracic and mediastinal disorders	n (%)	n (%)	X.X (X.X – X.X)	X.XXX
…				
**Subjects with at least one AE by preferred term**				
E.g Fever	n (%)	n (%)	X.X (X.X – X.X)	X.XXX
….				

*CI denotes confidence interval, AE Adverse Event, SAE Serious Adverse Event, SOC System Organ Class

†Except for number of AE/SAE, these outcomes are analysed using a log-binomial regression model and display the estimate of the risk ratio of IV iron versus standard-of-care (oral iron) and 95% CI and P-Value.

‡ Unadjusted P-values and 95% confidence intervals are presented here.

Table 9 – Neonate Unplanned clinic visits

	IV Iron N = XXX	Standard of care – Oral Iron N = XXX	Risk Ratio (95% CI)†	P-value‡
All-cause sick visits (including infection)	n (%)	n (%)	X.X (X.X – X.X)	X.XXX
Diarrhoea related visits	n (%)	n (%)	X.X (X.X – X.X)	X.XXX
Respiratory related visits	n (%)	n (%)	X.X (X.X – X.X)	X.XXX
Clinical malaria-specific visits	n (%)	n (%)	X.X (X.X – X.X)	X.XXX

CI denotes confidence interval,

†These outcomes are analysed using a log-binomial regression model and display the estimate of the risk ratio of IV iron versus standard-of-care (oral iron) and 95% CI and P-Value.

‡ Unadjusted P-values and 95% confidence intervals are presented here.

Figure 1 – CONSORT flow chart

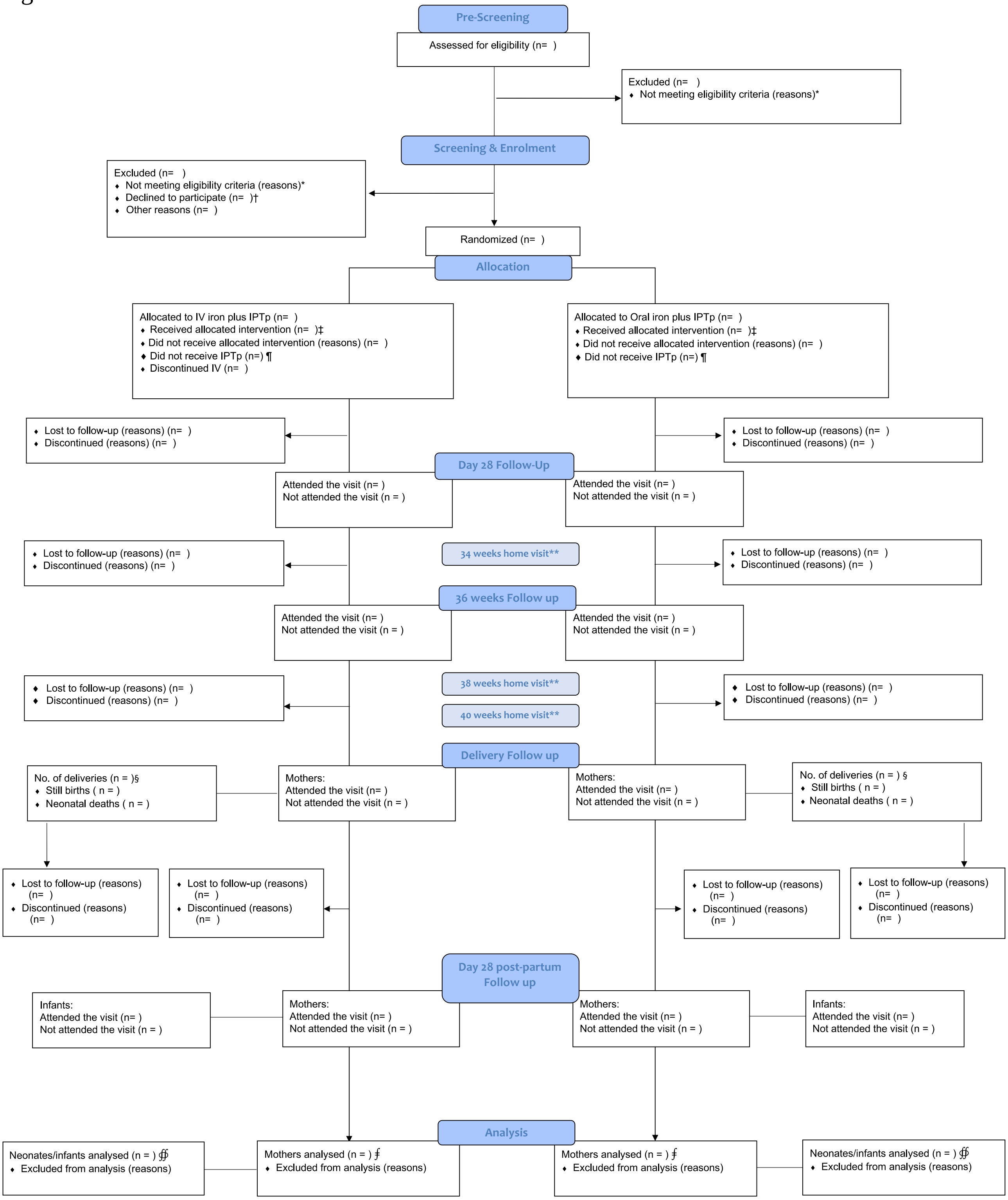

*Reasons for not meeting eligibility were assessed on the questions on the eligibility data collection form
†Defined as those who answered No to the question "Accepts study procedures?" at enrolment.
‡Reasons for not receiving the treatment were collected on the participant randomisation form.
¶ Defined as those who received at least one dose of Intermittent Preventive Treatment in pregnancy (IPTp) with suldadoxine pyrimethamine (SP) or with a contraindication to receiving SP
**Home visits collecting capillary Hb and adherence discontinued due to COVID restrictions.
§ there were X twins born in the IV iron group and X in the standard-of-care oral iron group
∱ XXX mothers in the IV iron group and XXX mothers in the standard-of-care oral iron group were included in the Intention-to-treat population
∯ XXX neonates in the IV iron group and XXX neonates in the standard-of-care oral iron group were included in the Intention-to-treat population

Changes to the Statistical Analysis Plan
The changes comprised:

After unblinding:

- A post-hoc definition of the primary endpoint was analysed. This new definition consisted of the outcome anemia at 36 weeks' gestation during pregnancy or anemia at delivery if the woman delivered prior to or at 36 weeks' gestation and aimed to provide a more complete capture of the effect of FCM treatment on anaemia prior to the high-risk period of delivery, including women who delivered prematurely. This endpoint was defined in addition to the primary endpoint which consisted of the outcome anemia at 36 weeks' gestation while being pregnant. No P-values were presented for this post-hoc analysis.

- The planned ordered logistic regression model analysis for hypophosphatemia could not be performed due to sparse data, i.e., we did not have cases in more than one category of hypophosphatemia, and as a result the underlying assumptions for the model may not be fulfilled. Phosphate data was only available after breaking the blind as it was considered potentially able to unblind treatment assignment.

- Maternal and neonatal unplanned clinic visit analyses were omitted. The unplanned visit data did not provide any additional value over AE data already collected by the 24-hour cover of the study research sites. It was felt AE data provided the most complete capture of illnesses and symptoms experienced by women; it was recognised that some women may have presented for care at a non-study health centre, and this visit would not be captured in the unplanned visits outcome; however, these data were still captured as AEs occurring between fixed study visit timepoints.

Ferric Carboxymaltose for anemia in the second trimester of pregnancy in Malawian women: Supplementary Data

Supplemental Table 1. Baseline Characteristics of the Participating Pregnant Women – (Per - Protocol Population)* ……………………………………………………………………222

Supplemental Table 2. Subgroup Analyses of Effect of Ferric Carboxymaltose on Key Maternal Outcomes at 36 Weeks' Gestation and Neonate Outcomes at Delivery According to Baseline Characteristics*…………………………………………………………………224

Supplemental Table 3. Maternal Safety Outcomes - Adverse Reactions During Ferric Carboxymaltose Administration ……………………………………………………………231

Supplemental Table 4. Effects of Ferric Carboxymaltose on Maternal and Neonatal Adverse Events Classified by Systemic Organ Class or Preferred Term*……………………………………………………………………………………232

Supplemental Table 5. Additional Analyses on Maternal and Neonate Outcomes………238

Supplemental Table 1. Baseline Characteristics of the Participating Pregnant Women – (Per -Protocol Population)*

	Ferric carboxymaltose N = 428	Standard-of-care N = 430
Age (years)	22.2 ± 6.0	22.6 ± 6.3
Primiparous – no. (%)‡	231 (54)	238 (55)
Primigravid – no. (%)‡	229 (53)	234 (54)
Gestational age – weeks		
Median	21.9	21.8
Interquartile range	19.6-24.2	18.9-24.0
Height (cm)	155.4 ± 5.8	155.3 ± 6.9
Weight (kg)	55.8 ± 8.0	55.6 ± 8.6
Body mass index (kg/m^2) †	23.1 ± 2.9	23.0 ± 3.2
Religion – no. /total no. (%)‡		
None	1/427 (<1)	1/428 (<1)
Christian	291/427 (68)	323/428 (75)
Muslim	132/427 (31)	99/428 (23)
Other	3/427 (<1)	5/428 (1)
Education – no. /total no. (%)‡		
None	1/415 (<1)	1/411 (<1)
Lower Primary	87/415 (21)	87/411 (21)
Upper Primary	171/415 (41)	167/411 (41)
Lower Secondary	56/415 (13)	59/411 (14)
Upper Secondary	90/415 (22)	87/411 (21)
Tertiary	10/415 (2)	10/411 (2)
Marital status – no. /total no. (%)‡		
Single	65/427 (15)	68/428 (16)
Married	357/427 (84)	349/428 (82)
Widowed	1/427 (<1)	3/428 (<1)
Divorced/Separated	4/427 (1)	6/428 (1)
Other	0/427 (0)	2/428 (<1)
Income source – no. /total no. (%)‡		
None	22/427 (5)	31/428 (7)
Subsistence farming	79/427 (19)	72/428 (17)
Large scale farming	1/427 (<1)	1/428 (<1)
Employed	84/427 (20)	67/428 (16)
Casual work for wages	134/427 (31)	128/428 (30)
Business	101/427 (24)	123/428 (29)
Other	6/427 (1)	6/428 (1)
Re-screened post positive malaria RDT – no. /total no. (%)§	129/428 (30)	123/430 (29)
Malaria RDT positive – no. /total no. (%)¶	6/418 (1)	5/424 (1)
HIV positive – no. /total no. (%)‡	70/425 (16)	74/426 (17)
Capillary Hb<10g/dL – no. /total no. (%)	427/427 (100)	429/429 (100)
Venous Hb – g/dL‖	8.82 ± 1.28	8.83 ± 1.23
Anemia (based on venous Hb) – no. /total no. (%)		

	Ferric carboxymaltose<br>N = 428	Standard-of-care<br><br>N = 430
No (venous Hb≥11g/dL)	24/424 (6)	18/430 (4)
Mild (10g/dL≤venous Hb<11g/dL)	49/424 (12)	56/430 (13)
Moderate (7g/dL≤venous Hb<10g/dL)	320/424 (75)	325/430 (76)
Severe (Hb<7g/dL)	31/424 (7)	31/430 (7)
Ferritin – ug/L**		
Median	26.10	27.50
Interquartile range	9.80-78.70	10.00-63.60
C-reactive protein – mg/L**		
Median	5.20	5.20
Interquartile range	2.60-10.60	3.00-10.60
Iron deficient – no. /total no. (%)††	180/417 (43)	185/423 (44)
Iron deficiency anemia – no. /total no. (%)††	169/413 (41)	179/423 (42)
Inflammation – no. /total no. (%)‡‡	214/417 (51)	217/423 (51)
Anemia and inflammation – no. /total no. (%)‡‡	203/413 (49)	207/423 (49)

The Per-protocol population excludes 4 women due to multiple pregnancy.
Participants were enrolled across two sites, Blantyre (N=139) and Zomba (N=723).
* No./total no. (%) are counts and percentages. Plus-minus values are means ±SD. RDT denotes rapid diagnostic test (for *Plasmodium* parasitaemia), Hb hemoglobin, and HIV human immunodeficiency virus.
† Body mass index is the weight in kilograms divided by the square of the height in meters.
‡ Religion, Education, Marital status, Income source, parity, gravidity and HIV status were self reported.
§ If women met the anemia criteria but had a positive RDT, they were treated for malaria as per local protocols and deferred from enrolment. These women were able to present for re-screening – no earlier than seven days later – and be enrolled if they met the eligibility criteria (in these cases, parasitemia was assessed using microscopy due to persistence of antigen detection via RDT).
¶ Malaria RDT positive based on confirmatory RDT testing by lab personal on venous blood collected at enrollment.
|| Data are missing for 4 participants in the Ferric carboxymaltose group and 0 participants in the standard-of-care group.
** Data are missing for 11 participants in the Ferric carboxymaltose group and 7 participants in the standard-of-care group.
†† Iron deficient indicates serum ferritin<15ug/L or ferritin<30ug/L if C-reactive protein>5mg/L, and iron deficiency anemia indicates Hb<11g/dL and serum ferritin<15ug/L or ferritin<30ug/L if C-reactive protein>5mg/L.
‡‡ Inflammation indicates C-reactive protein>5mg/L, and anemia and inflammation indicates Hb<11.0g/dL and C-reactive protein>5mg/L.

Supplemental Table 2. Subgroup Analyses of Effect of Ferric Carboxymaltose on Key Maternal Outcomes at 36 Weeks' Gestation and Neonate Outcomes at Delivery According to Baseline Characteristics*

Anemia at 36 weeks' gestation	Ferric carboxymaltose N = 430	Standard-of-care N = 432	Prevalence Ratio† (95% CI)	P-value§§ (Subgroup)	P-value§§ (Interaction)
Overall	179/341 (52)	189/333 (57)	0.92 (0.81, 1.06)	0.27	-
Parity					0.97
Primiparous	88/182 (48)	95/182 (52)	0.93 (0.75, 1.14)	0.46	
Multiparous	91/159 (57)	94/151 (62)	0.92 (0.77, 1.11)	0.37	
HIV status‖					0.74
Positive	30/55 (55)	33/53 (62)	0.88 (0.64, 1.21)	0.42	
Negative	148/284 (52)	155/277 (56)	0.93 (0.80, 1.08)	0.36	
Severe anaemia**					0.84
Yes	16/22 (73)	21/28 (75)	0.97 (0.70, 1.35)	0.86	
No	163/317 (51)	168/305 (55)	0.93 (0.81, 1.08)	0.36	
Iron deficient††					0.28
Yes	75/154 (49)	82/144 (57)	0.86 (0.69, 1.06)	0.15	
No	98/176 (56)	103/185 (56)	1.00 (0.83, 1.20)	0.99	
Iron deficient anaemia‡‡					0.44
Yes	75/146 (51)	81/139 (58)	0.88 (0.71, 1.09)	0.24	
No	98/182 (54)	104/190 (55)	0.98 (0.82, 1.19)	0.87	
Inflammation††					0.69
Yes	91/171 (53)	99/169 (59)	0.91 (0.75, 1.10)	0.32	
No	82/159 (52)	86/160 (54)	0.96 (0.78, 1.18)	0.71	
Re-screened post positive malaria RDT					0.83
Yes	50/100 (50)	51/92 (55)	0.90 (0.69, 1.18)	0.46	
No	129/241 (54)	138/241 (57)	0.93 (0.80, 1.10)	0.41	
Site					0.67
Blantyre	26/52 (50)	25/50 (50)	1.00 (0.68, 1.47)	>0.99	
Zomba	153/289 (53)	164/283 (58)	0.91 (0.79, 1.06)	0.23	

Change from baseline in hemoglobin at 36 weeks' gestation (g/dL)	Ferric carboxymaltose N = 430	Standard-of-care N = 432	Mean Difference‡ (95% CI)	P-value§§ (Subgroup)	P-value§§ (Interaction)
Overall	2.02 ± 1.41	1.85 ± 1.49	0.15 (-0.02, 0.33)	0.077	-
Parity					0.048
Primiparous	2.15 ±1.42	2.22 ±1.47	0.01 (-0.23, 0.24)	0.96	
Multiparous	1.88 ±1.38	1.40 ±1.39	0.35 (0.10, 0.60)	0.0063	
HIV status‖					0.66
Positive	1.97 ±1.15	1.83 ±1.34	0.24 (-0.18, 0.66)	0.27	
Negative	2.04 ±1.46	1.86 ±1.52	0.14 (-0.05, 0.32)	0.16	
Severe anaemia**					0.11
Yes	3.50 ±1.55	3.55 ±1.75	-0.34 (-0.97, 0.29)	0.29	
No	1.92 ±1.34	1.69 ±1.36	0.19 (0.01, 0.37)	0.035	
Iron deficient††					0.069
Yes	2.19 ±1.37	1.92 ±1.63	0.34 (0.08, 0.60)	0.011	
No	1.89 ±1.41	1.80 ±1.37	0.01 (-0.22, 0.25)	0.91	
Iron deficient anaemia‡‡					0.057
Yes	2.27 ±1.35	1.98 ±1.62	0.35 (0.09, 0.62)	0.0092	
No	1.84 ±1.42	1.76 ±1.38	0.01 (-0.22, 0.24)	0.91	
Inflammation††					0.58
Yes	2.04 ± 1.50	1.80 ± 1.51	0.20 (-0.04, 0.44)	0.11	
No	2.01± 1.29	1.91 ± 1.46	0.10 (-0.15, 0.35)	0.43	
Re-screened post positive malaria RDT					0.51
Yes	2.15 ± 1.41	2.04 ± 1.35	0.06 (-0.26, 0.38)	0.70	
No	1.97 ± 1.40	1.78 ± 1.54	0.19 (-0.01, 0.39)	0.068	
Site					0.78
Blantyre	2.08 ± 1.43	1.80 ± 1.92	0.09 (-0.35, 0.54)	0.68	
Zomba	2.01 ± 1.40	1.86 ± 1.41	0.16 (-0.02, 0.35)	0.088	

Birthweight (grams)	Ferric carboxymaltose N = 395	Standard-of-care N = 401	Mean Difference§ (95% CI)	P-value§§ (Subgroup)	P-value§§ (Interaction)
Overall	2893.2 ± 496.7	2896.2 ± 515.65	-3.1 (-75.0, 68.9)	0.93	-
Parity					0.87
Primiparous	2826.0 ± 438.5	2830.2 ± 512.2	-4.17 (-101.0, 92.7)	0.93	
Multiparous	2966.7 ± 545.0	2982.3 ± 508.9	-15.7 (-121.5, 90.2)	0.77	
HIV status‖					0.75
Positive	2949.2 ± 647.7	2924.2 ± 503.3	22.9 (-155.3, 201.1)	0.80	
Negative	2880.1 ± 462.4	2889.6 ± 518.7	-9.19 (-88.4, 70.0)	0.82	
Severe anaemia**					0.067
Yes	2763.4 ± 585.0	3017.0 ± 583.4	-253.0 (-526.1, 20.1)	0.069	
No	2898.4 ± 488.9	2886.5 ± 509.5	11.7 (-63.0, 86.4)	0.76	
Iron deficient††					0.93
Yes	2966.3 ± 466.8	2974.7 ± 452.7	-8.33(-118.9, 102.2)	0.88	
No	2834.3 ± 520.2	2835.8 ± 551.2	-1.53 (-97.9, 94.8)	0.98	
Iron deficient anaemia‡‡					0.85
Yes	2970.9 ± 452.1	2969.6 ± 450.7	1.42 (-112.1, 115.0)	0.98	
No	2830.2 ± 525.3	2843.0 ± 551.2	-13.0 (-107.9, 81.9)	0.79	
Inflammation††					0.074
Yes	2831.1 ± 491.9	2899.5 ± 538.8	-67.7 (-169.9, 34.4)	0.19	
No	2955.3 ± 503.7	2889.0 ± 491.7	65.5 (-38.9, 169.8)	0.22	
Re-screened post positive malaria RDT					0.33
Yes	2840.4 ± 464.0	2787.2 ± 610.7	52.9 (-77.2, 183.0)	0.43	
No	2917.2 ± 509.8	2942.1 ± 463.6	-24.9 (-110.7, 61.0)	0.57	
Site					0.53
Blantyre	2893.8 ± 504.8	2953.9 ± 430.8	-60.1 (-251.5, 131.3)	0.54	
Zomba	2893.1 ± 496.1	2886.8 ± 528.2	6.34 (-71.4, 84.1)	0.87	

Low birthweight (<2500grams)	Ferric carboxymaltose N = 395	Standard-of-care N = 401	Risk Ratio¶ (95% CI)	P-value§§ (Subgroup)	P-value§§ (Interaction)
Overall	67/385 (17)	61/378 (16)	1.08 (0.79, 1.48)	-	-
Parity					0.035
Primiparous	33/201 (16)	43/214 (20)	0.82 (0.54, 1.23)	0.34	
Multiparous	34/184 (18)	18/164 (11)	1.68 (0.99, 2.86)	0.055	
HIV status‖					0.13
Positive	14/63 (22)	7/62 (11)	1.97 (0.85, 4.55)	0.11	
Negative	53/320 (17)	53/312 (17)	0.97 (0.69, 1.38)	0.89	
Severe anaemia**					0.96
Yes	5/25 (20)	5/28 (18)	1.12 (0.37, 3.42)	0.84	
No	62/356 (17)	56/350 (16)	1.09 (0.78, 1.51)	0.61	
Iron deficient††					0.50
Yes	22/165 (13)	16/157 (10)	1.31 (0.72, 2.41)	0.38	
No	44/209 (21)	44/215 (20)	1.03 (0.71, 1.49)	0.89	
Iron deficient anaemia‡‡					0.67
Yes	19/154 (12)	15/151 (10)	1.25 (0.66, 2.36)	0.50	
No	47/216 (22)	45/221 (20)	1.07 (0.74, 1.53)	0.73	
Inflammation††					0.22
Yes	41/189 (22)	32/192 (17)	1.30 (0.86, 1.98)	0.21	
No	25/185 (14)	28/180 (16)	0.87 (0.53, 1.43)	0.58	
Re-screened post positive malaria RDT					0.012
Yes	22/120 (18)	31/112 (28)	0.66 (0.40, 1.06)	0.087	
No	45/265 (17)	30/266 (11)	1.51 (0.98, 2.32)	0.060	
Site					0.41
Blantyre	11/55 (20)	7/53 (13)	1.51 (0.63, 3.61)	0.35	
Zomba	56/330 (17)	54/325 (17)	1.02 (0.73, 1.44)	0.90	

Gestation duration (weeks)	Ferric carboxymaltose N = 395	Standard-of-care N = 401	Mean Difference§ (95% CI)	P-value§§ (Subgroup)	P-value§§ (Interaction)
Overall	39.49 ± 1.95	39.41 ± 2.29	0.08 (-0.22, 0.38)	0.59	-
Parity					0.56
Primiparous	39.43 ± 2.04	39.43 ± 2.34	0.00 (-0.41, 0.41)	0.99	
Multiparous	39.55 ± 1.83	39.37 ± 2.24	0.18 (-0.27, 0.62)	0.43	
HIV status‖					0.40
Positive	39.59 ± 2.00	39.21 ± 2.56	0.38 (-0.36, 1.13)	0.31	
Negative	39.47 ± 1.94	39.44 ± 2.24	0.03 (-0.30, 0.36)	0.86	
Severe anaemia**					0.11
Yes	38.99 ± 2.45	39.82 ± 1.54	-0.83 (-1.97, 0.31)	0.15	
No	39.52 ± 1.91	39.37 ± 2.34	0.14 (-0.17, 0.45)	0.37	
Iron deficient††					0.60
Yes	39.70 ± 1.62	39.55 ± 2.14	0.15 (-0.31, 0.61)	0.53	
No	39.29 ± 2.17	39.31 ± 2.41	-0.02 (-0.42, 0.39)	0.94	
Iron deficient anaemia‡‡					0.26
Yes	39.76 ± 1.59	39.50 ± 2.13	0.26 (-0.21, 0.74)	0.28	
No	39.26 ± 2.17	39.36 ± 2.42	-0.10 (-0.50, 0.30)	0.63	
Inflammation††					0.59
Yes	39.32 ± 2.02	39.35 ± 2.33	-0.02 (-0.45, 0.40)	0.91	
No	39.63 ± 1.87	39.49 ± 2.27	0.14 (-0.29, 0.58)	0.52	
Re-screened post positive malaria RDT					0.15
Yes	39.39 ± 2.20	38.97 ± 2.58	0.42 (-0.12, 0.96)	0.13	
No	39.54 ± 1.82	39.59 ± 2.14	-0.06 (-0.42, 0.30)	0.76	
Site					0.81
Blantyre	39.51 ± 2.04	39.34 ± 2.11	0.17 (-0.63, 0.97)	0.67	
Zomba	39.49 ± 1.93	39.42 ± 2.32	0.07 (-0.26, 0.39)	0.68	

Premature birth (<37 weeks GA)	Ferric carboxymaltose N = 395	Standard-of-care N = 401	Risk Ratio¶ (95% CI)	P-value§§ (Subgroup)	P-value§§ (Interaction)
Overall	31/387 (8)	35/385 (9)	0.88 (0.55, 1.40)	-	-
Parity					0.68
Primiparous	17/202 (8)	19/218 (9)	0.96 (0.52, 1.80)	0.91	
Multiparous	14/185 (8)	16/167 (10)	0.79 (0.40, 1.57)	0.50	
HIV status‖					0.56
Positive	6/64 (9)	5/63 (8)	1.19 (0.38, 3.70)	0.77	
Negative	25/321 (8)	30/318 (9)	0.82 (0.50, 1.37)	0.46	
Severe anaemia**					0.21
Yes	3/25 (12)	1/29 (3)	3.47 (0.39, 31.33)	0.27	
No	28/358 (8)	34/356 (10)	0.82 (0.51, 1.32)	0.41	
Iron deficient††					0.68
Yes	9/166 (5)	11/162 (7)	0.80 (0.34, 1.88)	0.61	
No	22/210 (10)	23/217 (11)	0.99 (0.57, 1.72)	0.96	
Iron deficient anaemia‡‡					0.35
Yes	7/155 (5)	11/156 (7)	0.64 (0.26, 1.61)	0.34	
No	24/217 (11)	23/223 (10)	1.07 (0.62, 1.84)	0.80	
Inflammation††					0.42
Yes	19/190 (10)	18/196 (9)	1.09 (0.59, 2.01)	0.79	
No	12/186 (6)	16/183 (9)	0.74 (0.36, 1.52)	0.41	
Re-screened post positive malaria RDT					0.47
Yes	13/120 (11)	17/114 (15)	0.72 (0.37, 1.42)	0.34	
No	18/267 (7)	18/271 (7)	1.02 (0.54, 1.91)	0.96	
Site					0.87
Blantyre	4/55 (7)	5/55 (9)	0.80 (0.23, 2.82)	0.73	
Zomba	27/332 (8)	30/330 (9)	0.89 (0.54, 1.47)	0.66	

* No./total no. (%) are counts and percentages, plus-minus values are means ±SD, CI confidence interval and GA gestational age. Anemia indicates venous Hb<11.0g/dL. Severe anemia indicates venous Hb<7.0g/dL. Iron deficient indicates serum ferritin<15ug/L or ferritin<30ug/L if C-reactive protein>5mg/L, and iron deficient anemia indicates Hb<11g/dL and serum ferritin<15ug/L or ferritin<30ug/L if C-reactive protein>5mg/L. Inflammation indicates C-reactive protein>5mg/L. Re-screened post positive malaria RDT indicates women who were enrolled after an initial pre-screening positive malaria test, re-screened for malaria a week later and if negative then enrolled (with no other exclusion criteria present).

† A prevalence ratio of Ferric carboxymaltose versus standard-of-care is displayed for anemia at 36 weeks' gestation using a Poisson model with robust standard errors. Subgroup (main effect) and subgroup-by-treatment-by-visit interaction (as well as subgroup-by-treatment and subgroup-by-visit interaction) have been added to the model to evaluate where the treatment effect differs between subgroup categories.
‡ A mean difference of ferric carboxymaltose versus standard-of-care of the estimated change from baseline to at 36 weeks' gestation is displayed for continuous hemoglobin concentration following analyses using a likelihood-based longitudinal data analysis model. Subgroup (main effect) and subgroup-by-treatment-by-visit interaction (as well as subgroup-by-treatment and subgroup-by-visit interaction) have been added to the model to evaluate where the treatment effect differs between subgroup categories.
§ An absolute mean difference for birthweight, gestation duration at delivery between ferric carboxymaltose and standard-of-care is displayed following fitting a linear regression model. Subgroup (main effect) and subgroup-by-treatment interactions terms have been added to the model to evaluate where the treatment effect differs between subgroup categories.
¶ A risk ratio of IV iron versus standard of care is displayed for low birthweight, premature birth, following analyses using a log-binomial regression model. Subgroup (main effect) and subgroup-by-treatment interactions terms have been added to the model to evaluate where the treatment effect differs between subgroup categories.
|| Data are missing for 3 participants in the Ferric carboxymaltose group and 4 participants in the standard-of-care group.
** Data are missing for 4 participants in the Ferric carboxymaltose group and 0 participants in the standard-of-care group.
†† Data are missing for 11 participants in the Ferric carboxymaltose group and 7 participants in the standard-of-care group.
‡‡ Data are missing for 15 participants in the Ferric carboxymaltose group and 7 participants in the standard-of-care group.
§§ The P-values and 95% confidence intervals presented have not been adjusted for multiple comparisons. The intervals may not be used in place of hypothesis testing.

Supplemental Table 3. Maternal Safety Outcomes - Adverse Reactions During Ferric Carboxymaltose Administration

Outcome	Ferric carboxymaltose N = 430
Composite any Ferric Carboxymaltose -related event – no. (%)	28 (7)
Individual components of composite any Ferric Carboxymaltose -related event – no. (%)	
Headache	10 (2)
Dizziness	8 (2)
Dysgeusia	3 (<1)
Discoloration of the skin	0 (0)
Nausea	12 (3)
Vomiting	2 (<1)
Constipation	2 (<1)
Upper abdominal pain	2 (<1)
Dyspepsia	3 (<1)
Anaphylaxis	0 (0)
Flushing	2 (<1)
Shortness of breath	4 (1)
Chest pains	2 (<1)
Other†	2 (<1)

Includes all women who were randomised to the Ferric carboxymaltose group.
†Other Ferric Carboxymaltose-related events indicated as urticaria

Supplemental Table 4. Effects of Ferric Carboxymaltose on Maternal and Neonatal Adverse Events Classified by Systemic Organ Class or Preferred Term*

	Ferric carboxymaltose N = 430	Standard-of-care N = 432	Risk Ratio¶ (95% CI)	P-value‖
**Maternal safety outcome†**				
**System Organ Class – no. (%)‡**				
Infections and Infestations	103 (24)	87 (20)	1.19 (0.92, 1.52)	0.19
Pregnancy, Puerperium and Perinatal Conditions	72 (17)	75 (17)	0.97 (0.72, 1.30)	0.83
Musculoskeletal and connective tissue disorders	8 (2)	4 (1)	2.02 (0.61, 6.65)	0.25
Nervous system disorders	8 (2)	6 (1)	1.33 (0.47, 3.79)	0.60
Gastrointestinal disorders	5 (1)	6 (1)	0.84 (0.26, 2.72)	0.77
Blood and lymphatic system disorders	4 (1)	4 (1)	1.00 (0.25, 3.98)	>0.99
General disorders and administration site conditions	3 (<1)	1 (<1)	3.02 (0.32, 28.91)	0.34
Vascular disorders	3 (<1)	2 (<1)	1.51 (0.25, 8.97)	0.65
Skin and subcutaneous tissue disorders	3 (<1)	2 (<1)	1.50 (0.25, 8.95)	0.65
Injury, poisoning and procedural complications	2 (<1)	3 (<1)	0.67 (0.11, 3.97)	0.66
Cardiac disorders	2 (<1)	0 (0)	-	-
Reproductive system and breast disorders	1 (<1)	0 (0)	-	-
Neoplasms benign, malignant and unspecified (incl cysts and polyps)	1 (<1)	0 (0)	-	-
Respiratory, thoracic and mediastinal disorders	0 (0)	3 (<1)	-	-
Congenital, familial and genetic disorders	0 (0)	1 (<1)	-	-
Eye disorders	0 (0)	1 (<1)	-	-
**Preferred Term – no. (%)‡**				
Pregnancy, puerperium and perinatal conditions, other	66 (15)	69 (16)	0.96 (0.71, 1.31)	0.81
Perineal Tear	44 (10)	47 (11)	0.94 (0.64, 1.38)	0.74
Postpartum haemorrhage	8 (2)	7 (2)	1.15 (0.42, 3.13)	0.79
Cord around neck	3 (<1)	0 (0)	-	-
Eclampsia	2 (<1)	1 (<1)	2.01 (0.18, 22.02)	0.57

Retained placenta	2 (<1)	1 (<1)	2.01 (0.18, 21.92)	0.57
Fetal distress	2 (<1)	0 (0)	-	-
Abnormal fetal heart rate	1 (<1)	1 (<1)	1.00 (0.06, 15.97)	>0.99
Pre-eclampsia	1 (<1)	4 (1)	0.25 (0.03, 2.23)	0.22
Episiotomy	1 (<1)	1 (<1)	1.00 (0.06, 15.97)	>0.99
Threatened abortion	1 (<1)	0 (0)	-	-
Antepartum haemorrhage	1 (<1)	0 (0)	-	-
Abnormal fetal lie	1 (<1)	0 (0)	-	-
Premature rupture of membranes	0 (0)	1 (<1)	-	-
Puerperal sepsis	0 (0)	1 (<1)	-	-
Placental abruption	0 (0)	1 (<1)	-	-
Placenta praevia	0 (0)	1 (<1)	-	-
Obstructed labor	0 (0)	1 (<1)	-	-
Prolonged labour	0 (0)	1 (<1)	-	-
Vaginal warts preventing vaginal delivery; caesarean section	0 (0)	1 (<1)	-	-
Birth asphyxia	0 (0)	1 (<1)	-	-
Urinary Tract Infection	51 (12)	45 (10)	1.14 (0.78, 1.66)	0.50
Infections and infestations, other	44 (10)	31 (7)	1.42 (0.92, 2.21)	0.11
Malaria	26 (6)	20 (5)	1.31 (0.74, 2.30)	0.36
Gastroenteritis	8 (2)	5 (1)	1.63 (0.55, 4.86)	0.38
Syphillis	5 (1)	2 (<1)	2.51 (0.49, 12.84)	0.27
Schistosomiasis	4 (1)	2 (<1)	2.01 (0.37, 10.90)	0.42
Infection without evident site	2 (<1)	3 (<1)	0.67 (0.11, 3.98)	0.66
Cryptococcal infection	1 (<1)	0 (0)	-	-
Lung infection	7 (2)	5 (1)	1.39 (0.45, 4.31)	0.57
Myalgia	6 (1)	3 (<1)	2.01 (0.51, 8.00)	0.32
Upper respiratory infection	6 (1)	4 (1)	1.51 (0.43, 5.31)	0.52
Pregnancy loss	5 (1)	6 (1)	0.84 (0.26, 2.72)	0.77
Headache	5 (1)	2 (<1)	2.51 (0.49, 12.88)	0.27
Anemia	4 (1)	4 (1)	1.00 (0.25, 3.98)	>0.99

Kidney infection	4 (1)	1 (<1)	4.04 (0.45, 35.97)	0.21
Abdominal pain	3 (<1)	4 (1)	0.75 (0.17, 3.34)	0.71
Vaginal infection	3 (<1)	2 (<1)	1.51 (0.25, 8.97)	0.65
Dizziness	3 (<1)	1 (<1)	3.01 (0.31, 28.82)	0.34
Edema limbs	2 (<1)	0 (0)	-	-
Vomiting	2 (<1)	0 (0)	-	-
Premature delivery	2 (<1)	0 (0)	-	-
Injury, poisoning and procedural complications, other	2 (<1)	0 (0)	-	-
Physical assault	1 (<1)	0 (0)	-	-
Laceration	1 (<1)	0 (0)	-	-
Hypertension	2 (<1)	2 (<1)	1.00 (0.14, 7.09)	>0.99
Urticaria	2 (<1)	0 (0)	-	-
Uterine infection	1 (<1)	0 (0)	-	-
Mitral valve disease	1 (<1)	0 (0)	-	-
Chest pain - cardiac	1 (<1)	0 (0)	-	-
Urethral infection	1 (<1)	0 (0)	-	-
Flushing	1 (<1)	0 (0)	-	-
Skin papilloma	1 (<1)	0 (0)	-	-
Anal ulcer	1 (<1)	0 (0)	-	-
Skin infection	1 (<1)	0 (0)	-	-
Skin and subcutaneous tissue disorders - other	1 (<1)	0 (0)	-	-
Scalp rash	1 (<1)	0 (0)	-	-
Breast pain	1 (<1)	0 (0)	-	-
Fatigue	1 (<1)	0 (0)	-	-
Thrush	1 (<1)	3 (<1)	0.33 (0.04, 3.19)	0.34
Otitis media	1 (<1)	0 (0)	-	-
Arthralgia	1 (<1)	1 (<1)	1.01 (0.06, 16.12)	0.99
Flank pain	1 (<1)	0 (0)	-	-
Breast infection	1 (<1)	0 (0)	-	-
Peripheral sensory neuropathy	0 (0)	2 (<1)	-	-
Gastric ulcer	0 (0)	1 (<1)	-	-

Wound complication	0 (0)	1 (<1)	-	-
Conjunctivitis infective	0 (0)	1 (<1)	-	-
Gastrointestinal disorders, other	0 (0)	1 (<1)	-	-
Anal prolapse	0 (0)	1 (<1)	-	-
Non cardiac chest pain	0 (0)	1 (<1)	-	-
Congenital, familial and genetic disorders, other	0 (0)	1 (<1)	-	-
Undeveloped left hand	0 (0)	1 (<1)	-	-
Respiratory, thoracic and mediastinal disorders – other	0 (0)	1 (<1)	-	-
Asthma	0 (0)	1 (<1)	-	-
Cough	0 (0)	1 (<1)	-	-
Epistaxis	0 (0)	1 (<1)	-	-
Conjunctivitis	0 (0)	1 (<1)	-	-
Rash maculo-papular	0 (0)	2 (<1)	-	-
Wound dehiscence	0 (0)	1 (<1)	-	-
Fall	0 (0)	1 (<1)	-	-
Syncope	0 (0)	1 (<1)	-	-
Blurred vision	0 (0)	1 (<1)	-	-
**Neonatal safety outcome**§				
**System Organ Class – no. (%)**‡				
Infections and infestations	13 (3)	11 (3)	1.20 (0.54, 2.65)	0.65
Pregnancy, puerperium and perinatal conditions	12 (3)	13 (3)	0.94 (0.43, 2.03)	0.87
Congenital, familial and genetic disorders	2 (<1)	4 (1)	0.51 (0.09, 2.74)	0.43
Skin And Subcutaneous Tissue Disorders	1 (<1)	2 (<1)	0.51 (0.05, 5.55)	0.58
Respiratory, thoracic and mediastinal disorders	1 (<1)	1 (<1)	1.01 (0.06, 16.11)	0.99
Eye disorders	1 (<1)	0 (0)	-	-
General disorders and administration site conditions	0 (0)	3 (<1)	-	-
Metabolism and nutrition disorders	0 (0)	1 (<1)	-	-
**Preferred Term – no. (%)**‡				
Pregnancy, puerperium and perinatal conditions, other	12 (3)	8 (2)	1.52 (0.63, 3.69)	0.35
Birth asphyxia	10 (2)	8 (2)	1.27 (0.51, 3.18)	0.61
Meconium aspiration	2 (<1)	0 (0)	-	-

Amniotic fluid aspiration	1 (<1)	0 (0)	-	-
Infections and infestations – other	6 (1)	3 (<1)	2.03 (0.51, 8.06)	0.31
Infection without evident site	3 (<1)	3 (<1)	1.02 (0.21, 5.00)	0.99
Malaria	2 (<1)	0 (0)	-	-
Gastroenteritis	1 (<1)	0 (0)	-	-
Sepsis	5 (1)	1 (<1)	5.06 (0.59, 43.08)	0.14
Congenital, familial and genetic disorders, other	2 (<1)	4 (1)	0.51 (0.09, 2.74)	0.43
Cleft Lip	1 (<1)	0 (0)	-	-
Polydactyly	1 (<1)	4 (1)	0.25 (0.03, 2.25)	0.22
Lung infection	2 (<1)	3 (<1)	0.68 (0.11, 4.03)	0.67
Skin and subcutaneous tissue disorders - other	1 (<1)	2 (<1)	0.51 (0.05, 5.55)	0.58
Non infected stump	1 (<1)	0 (0)	-	-
Rash	0 (0)	1 (<1)	-	-
Scalp rash	0 (0)	1 (<1)	-	-
Aspiration	1 (<1)	0 (0)		
Eye disorders – other	1 (<1)	0 (0)	-	-
Premature delivery	0 (0)	5 (1)	-	-
Sudden death nos	0 (0)	2 (<1)	-	-
Breast infection	0 (0)	1 (<1)		
Conjunctivitis	0 (0)	1 (<1)	-	-
Cough	0 (0)	1 (<1)	-	-
Dehydration	0 (0)	1 (<1)	-	-
General disorders and administration site conditions, other	0 (0)	1 (<1)	-	-
Birth injuries	0 (0)	1 (<1)	-	-
Meningitis	0 (0)	1 (<1)	-	-
Upper respiratory infection	0 (0)	1 (<1)	-	-

* CI denotes confidence interval.
† Includes all women who were treated, presented according to treated group. This includes 4 mothers with multiple pregnancies.
‡ Adverse events (AEs) were coded using version 5.0 of the Common Terminology Criteria for Adverse Events (CTCAE) (US Department of Health Human Services, 27 November 2020) and use an appropriate Preferred Term and System Organ Class for each AE verbatim term.

§ Includes all live-born neonates (with the exception of the death and pregnancy loss outcome) born to women who were treated, presented according to treated group of the mother. This includes 8 twins.
¶ A risk ratio of Ferric carboxymaltose iron versus standard-of-care following analyses using a log-binomial regression model.
|| The P-values and 95% confidence intervals presented have not been adjusted for multiple comparisons. The intervals may not be used in place of hypothesis testing.

Supplemental Table 5. Additional Analyses on Maternal and Neonate Outcomes*

Visit/Outcome	Additional analysis	Prevalence Ratio|||| or Mean Difference*** or Geometric Mean Ratio ††† (95% CI)	P value¶¶¶
**Maternal efficacy outcome**			
**Primary outcome**			
Anemia† at 36 weeks' gestation	Maternal first adjusted model¶	0.93 (0.81, 1.06) ||||	0.27
	Maternal second adjusted model||	0.94 (0.82, 1.07) ||||	0.37
	Adjusted for site, PP	0.92 (0.80, 1.06) ||||	0.25
	Adjusted for site, multiple imputation (delta -2.0) ¶¶	0.94 (0.85, 1.04) ||||	0.24
	Adjusted for site, multiple imputation (delta -1.5) ¶¶	0.94 (0.85, 1.05) ||||	0.27
	Adjusted for site, multiple imputation (delta -1.0) ¶¶	0.94 (0.84, 1.05) ||||	0.28
	Adjusted for site, multiple imputation (delta -0.5) ¶¶	0.94 (0.83, 1.06) ||||	0.30
	Adjusted for site, multiple imputation (delta 0) ¶¶	0.94 (0.82, 1.07) ||||	0.36
	Adjusted for site, multiple imputation (delta +0.5) ¶¶	0.94 (0.82, 1.08) ||||	0.38
	Adjusted for site, multiple imputation (delta +1.0) ¶¶	0.94 (0.81, 1.09) ||||	0.38
	Adjusted for site, multiple imputation (delta +1.5) ¶¶	0.94 (0.81, 1.10) ||||	0.45
	Adjusted for site, multiple imputation (delta +2.0) ¶¶	0.95 (0.82, 1.11) ||||	0.52
**Secondary outcomes**			
Moderate/Severe anemia† at 36 weeks' gestation ||||	Maternal first adjusted model¶	0.81 (0.61, 1.08) ||||	0.15
	Maternal second adjusted model||	0.81 (0.61, 1.08) ||||	0.15
	Adjusted for site, PP	0.81 (0.61, 1.07) ||||	0.14
Hb change from baseline at 36 weeks' gestation – g/dL	Maternal first adjusted model¶	0.15 (-0.02, 0.32) ***	0.085

	Maternal second adjusted model**<br><br>Adjusted for site, PP	0.15 (-0.05, 0.34) ***<br><br>0.15 (-0.02, 0.33) ***	0.14<br><br>0.080
Ferritin change from baseline at 36 weeks' gestation – μg/L	Maternal first adjusted model¶<br><br>Maternal second adjusted model‖<br><br>Adjusted for site, PP	2.56 (2.29, 2.86) †††<br><br>2.50 (2.19, 2.85) †††<br><br>2.57 (2.30, 2.87) †††	<0.0001<br><br><0.0001<br><br><0.0001
Iron deficient‡ at 36 weeks' gestation	Maternal first adjusted model¶<br><br>Maternal second adjusted model††<br><br>Adjusted for site, PP	0.43 (0.33, 0.55) ‖‖<br><br>0.43 (0.34, 0.55) ‖‖<br><br>0.43 (0.33, 0.56) ‖‖	<0.0001<br><br><0.0001<br><br><0.0001
Iron deficient anemia‡ at 36 weeks' gestation	Maternal first adjusted model¶<br><br>Maternal second adjusted model‖<br><br>Adjusted for site, PP	0.29 (0.20, 0.42) ‖‖<br><br>0.29 (0.20, 0.41) ‖‖<br><br>0.30 (0.21, 0.44) ‖‖	<0.0001<br><br><0.0001<br><br><0.0001
**Other secondary outcomes**			
Anemia†			
4-weeks post-treatment	Maternal first adjusted model¶<br><br>Maternal second adjusted model‖<br><br>Adjusted for site, PP<br><br>Adjusted for site, multiple imputation (delta -2.0) ¶¶<br><br>Adjusted for site, multiple imputation (delta -1.5) ¶¶<br><br>Adjusted for site, multiple imputation (delta -1.0) ¶¶<br><br>Adjusted for site, multiple imputation (delta -0.5) ¶¶<br><br>Adjusted for site, multiple imputation (delta 0) ¶¶<br><br>Adjusted for site, multiple imputation (delta +0.5) ¶¶	0.91 (0.85, 0.97) ‖‖<br><br>0.89 (0.84, 0.96) ‖‖<br><br>0.91 (0.85, 0.97) ‖‖<br><br>0.91 (0.86, 0.97) ‖‖<br><br>0.91 (0.86, 0.97) ‖‖<br><br>0.91 (0.85, 0.97) ‖‖<br><br>0.91 (0.85, 0.97) ‖‖<br><br>0.91 (0.85, 0.97) ‖‖<br><br>0.91 (0.85, 0.98) ‖‖	

	Adjusted for site, multiple imputation (delta +1.0) ¶¶	0.91 (0.85, 0.98) ||||	
	Adjusted for site, multiple imputation (delta +1.5) ¶¶	0.91 (0.85, 0.99) ||||	
	Adjusted for site, multiple imputation (delta +2.0) ¶¶	0.92 (0.85, 0.99) ||||	
Delivery	Maternal first adjusted model¶	0.88 (0.70, 1.10) ||||	
	Maternal second adjusted model||	0.86 (0.69, 1.07) ||||	
	Adjusted for site, PP	0.88 (0.70, 1.10) ||||	
	Adjusted for site, multiple imputation (delta -2.0) ¶¶	0.87 (0.72, 1.04) ||||	
	Adjusted for site, multiple imputation (delta -1.5) ¶¶	0.87 (0.72, 1.05) ||||	
	Adjusted for site, multiple imputation (delta -1.0) ¶¶	0.86 (0.70, 1.04) ||||	
	Adjusted for site, multiple imputation (delta -0.5) ¶¶	0.85 (0.69, 1.05) ||||	
	Adjusted for site, multiple imputation (delta 0) ¶¶	0.85 (0.68, 1.06) ||||	
	Adjusted for site, multiple imputation (delta +0.5) ¶¶	0.86 (0.68, 1.07) ||||	
	Adjusted for site, multiple imputation (delta +1.0) ¶¶	0.86 (0.69, 1.09) ||||	
	Adjusted for site, multiple imputation (delta +1.5) ¶¶	0.87 (0.69, 1.10) ||||	
	Adjusted for site, multiple imputation (delta +2.0) ¶¶	0.88 (0.70, 1.12) ||||	
4-weeks post-partum	Maternal first adjusted model¶	0.86 (0.74, 1.01) ||||	
	Maternal second adjusted model||	0.83 (0.71, 0.97) ||||	
	Adjusted for site, PP	0.86 (0.74, 1.01) ||||	
	Adjusted for site, multiple imputation (delta -2.0) ¶¶	0.89 (0.80, 1.00) ||||	
	Adjusted for site, multiple imputation (delta -1.5) ¶¶	0.88 (0.79, 0.99) ||||	
	Adjusted for site, multiple imputation (delta -1.0) ¶¶	0.87 (0.77, 0.98) ||||	

	Adjusted for site, multiple imputation (delta -0.5) ¶¶<br>Adjusted for site, multiple imputation (delta 0) ¶¶<br>Adjusted for site, multiple imputation (delta +0.5) ¶¶<br>Adjusted for site, multiple imputation (delta +1.0) ¶¶<br>Adjusted for site, multiple imputation (delta +1.5) ¶¶<br>Adjusted for site, multiple imputation (delta +2.0) ¶¶	0.85 (0.75, 0.98) |||| 0.85(0.73, 0.98) |||| 0.84 (0.72, 0.97) |||| 0.84 (0.72, 0.98) |||| 0.84 (0.72, 1.00) |||| 0.85 (0.72, 1.01) ||||	
Moderate/Severe anemia†			
4-weeks post- treatment	Maternal first adjusted model¶<br>Maternal second adjusted model|| Adjusted for site, PP	0.81 (0.61, 1.08) |||| 0.86 (0.74, 0.99) |||| 0.89 (0.76, 1.04) ||||	
Delivery	Maternal first adjusted model¶<br>Maternal second adjusted model|| Adjusted for site, PP	0.89 (0.60, 1.32) |||| 0.90 (0.62, 1.32) |||| 0.87 (0.59, 1.29) ||||	
4-weeks post-partum	Maternal first adjusted model¶<br>Maternal second adjusted model|| Adjusted for site, PP	0.92 (0.69, 1.24) |||| 0.82 (0.61, 1.10) |||| 0.90 (0.67, 1.22) ||||	
Hb change from baseline – g/dL			
4-weeks post- treatment	Maternal first adjusted model¶<br>Maternal second adjusted model**<br>Adjusted for site, PP	0.19 (0.05, 0.32) ***<br>0.18 (0.04, 0.32) ***<br>0.19 (0.06, 0.33) ***	
Delivery	Maternal first adjusted model¶<br>Maternal second adjusted model**	0.18 (-0.05, 0.41) ***<br>0.18 (-0.02, 0.38) ***	

	Adjusted for site, PP	0.19 (-0.04, 0.42) ***	
4-weeks post-partum	Maternal first adjusted model¶<br><br>Maternal second adjusted model**<br><br>Adjusted for site, PP	0.25 (0.05, 0.45) ***<br><br>0.27 (0.07, 0.48) ***<br><br>0.25 (0.05, 0.45) ***	
Ferritin change from baseline – µg/L			
4-weeks post- treatment	Maternal first adjusted model¶<br><br>Maternal second adjusted model‖<br><br>Adjusted for site, PP	6.50 (5.98, 7.06) †††<br><br>6.49 (5.81, 7.26) †††<br><br>6.53 (6.02, 7.08) †††	
Delivery	Maternal first adjusted model¶<br><br>Maternal second adjusted model‖<br><br>Adjusted for site, PP	1.89 (1.66, 2.16) †††<br><br>1.91 (1.68, 2.16) †††<br><br>1.89 (1.65, 2.15) †††	
4-weeks post-partum	Maternal first adjusted model¶<br><br>Maternal second adjusted model‖<br><br>Adjusted for site, PP	1.96 (1.71, 2.25) †††<br><br>2.00 (1.74, 2.29) †††<br><br>1.96 (1.71, 2.24) †††	
Iron deficient‡			
4-weeks post- treatment	Maternal first adjusted model¶<br><br>Maternal second adjusted model††<br><br>Adjusted for site, PP	0.01 (0.00, 0.07) ‖‖<br><br>0.01 (0.00, 0.07) ‖‖<br><br>0.01 (0.00, 0.07) ‖‖	
Delivery	Maternal first adjusted model¶<br><br>Maternal second adjusted model††<br><br>Adjusted for site, PP	0.52 (0.41, 0.65) ‖‖<br><br>0.53 (0.42, 0.66) ‖‖<br><br>0.53 (0.42, 0.66) ‖‖	
4-weeks post-partum	Maternal first adjusted model¶<br><br>Maternal second adjusted model††<br><br>Adjusted for site, PP	0.42 (0.29, 0.59) ‖‖<br><br>0.41 (0.29, 0.57) ‖‖<br><br>0.43 (0.30, 0.61) ‖‖	

Iron deficient anemia‡			
4-weeks post- treatment	Maternal first adjusted model¶<br>Maternal second adjusted model‖<br>Adjusted for site, PP	0.01 (0.00, 0.08) ‖‖<br>0.01 (0.00, 0.08) ‖‖<br>0.01 (0.00, 0.08) ‖‖	
Delivery	Maternal first adjusted model¶<br>Maternal second adjusted model‖<br>Adjusted for site, PP	0.38 (0.25, 0.59) ‖‖<br>0.38 (0.25, 0.59) ‖‖<br>0.40 (0.25, 0.62) ‖‖	
4-weeks post-partum	Maternal first adjusted model¶<br>Maternal second adjusted model‖<br>Adjusted for site, PP	0.40 (0.26, 0.62) ‖‖<br>0.40 (0.26, 0.61) ‖‖<br>0.42 (0.27, 0.65) ‖‖	
Visit/Outcome	Additional analysis	Mean Difference‡‡‡ or Risk Ratio §§§ (95% CI)	P value¶¶¶
**Neonatal efficacy outcome**			
**Key outcome**			
Birthweight – grams	Neonate first adjusted model‡‡<br>Neonate second adjusted model§§<br>Adjusted for site, PP	-6.67 (-78.28, 64.93) ‡‡‡<br>-6.67 (-78.28, 64.93) ‡‡‡<br>-5.07 (-75.56, 65.42) ‡‡‡	0.85<br>0.85<br>0.89
**Secondary outcomes**			
Gestation duration – weeks	Neonate first adjusted model‡‡<br>Neonate second adjusted model§§<br>Adjusted for site, PP	0.09 (-0.21, 0.39) ‡‡‡<br>0.09 (-0.21, 0.39) ‡‡‡<br>0.06 (-0.23, 0.35) ‡‡‡	0.57<br>0.57<br>0.70
Composite adverse birth outcome	Neonate first adjusted model‡‡<br>Neonate second adjusted model§§<br>Adjusted for site, PP	1.06 (0.89, 1.26) §§§<br>1.07 (0.90, 1.28) §§§<br>1.04 (0.88, 1.25) §§§	0.50<br>0.42<br>0.63

Individual components of composite adverse birth outcome			
Low birthweight (<2500grams)	Neonate first adjusted model‡‡<br><br>Neonate second adjusted model§§<br><br>Adjusted for site, PP	1.09 (0.80, 1.50) §§§<br><br>1.10 (0.80, 1.51) §§§<br><br>1.10 (0.79, 1.53) §§§	
Pregnancy loss or stillbirth	Neonate first adjusted model‡‡<br><br>Neonate second adjusted model§§<br><br>Adjusted for site, PP	0.81 (0.30, 2.15) §§§<br><br>0.82 (0.31, 2.17) §§§<br><br>0.79 (0.30, 2.10) §§§	
Premature birth (<37weeks GA)	Neonate first adjusted model‡‡<br><br>Neonate second adjusted model§§<br><br>Adjusted for site, PP	0.86 (0.54, 1.36) §§§<br><br>0.87 (0.55, 1.38) §§§<br><br>0.93 (0.57, 1.51) §§§	
Small for GA (<10th percentile)	Neonate first adjusted model‡‡<br><br>Neonate second adjusted model§§<br><br>Adjusted for site, PP	1.09 (0.89, 1.34) §§§<br><br>1.10 (0.89, 1.35) §§§<br><br>1.07 (0.87, 1.32) §§§	
Venous Hb 4-weeks post-partum – g/dL	Neonate first adjusted model‡‡<br><br>Neonate second adjusted model§§<br><br>Adjusted for site, PP	0.03 (-0.32, 0.37) ‡‡‡<br><br>0.02 (-0.32, 0.37) ‡‡‡<br><br>-0.03 (-0.37, 0.32) ‡‡‡	0.90<br><br>0.90<br><br>0.89
Weight 4-weeks post-partum – grams §	Neonate first adjusted model‡‡<br><br>Neonate second adjusted model§§<br><br>Adjusted for site, PP	-115.04 (-230.86, 0.78) ‡‡‡<br><br>-115.04 (-230.86, 0.78) ‡‡‡<br><br>-80.72 (-195.39, 33.95) ‡‡‡	0.052<br><br>0.052<br><br>0.17
**Other secondary outcomes**			
Birthlength – cm	Neonate first adjusted model‡‡<br><br>Neonate second adjusted model§§<br><br>Adjusted for site, PP	0.05 (-0.45, 0.56) ‡‡‡<br><br>0.05 (-0.45, 0.56) ‡‡‡<br><br>0.08 (-0.43, 0.58) ‡‡‡	

Length 4-weeks post-partum – cm §	Neonate first adjusted model‡‡<br><br>Neonate second adjusted model§§<br><br>Adjusted for site, PP	-0.04 (-0.70, 0.62) ‡‡‡<br><br>-0.04 (-0.70, 0.62) ‡‡‡<br><br>0.11 (-0.53, 0.76) ‡‡‡	
Weight for age z score 4-weeks post-partum §	Neonate first adjusted model‡‡<br><br>Neonate second adjusted model§§<br><br>Adjusted for site, PP	-0.22 (-0.43, 0.00) ‡‡‡<br><br>-0.22 (-0.43, 0.00) ‡‡‡<br><br>-0.15 (-0.36, 0.06) ‡‡‡	
Length for age z score 4-weeks post-partum §	Neonate first adjusted model‡‡<br><br>Neonate second adjusted model§§<br><br>Adjusted for site, PP	-0.03 (-0.36, 0.30) ‡‡‡<br><br>-0.03 (-0.36, 0.30) ‡‡‡<br><br>0.05 (-0.28, 0.37) ‡‡‡	
Weight for length z score 4-weeks post-partum §	Neonate first adjusted model‡‡<br><br>Neonate second adjusted model§§<br><br>Adjusted for site, PP	-0.38 (-0.73, -0.02) ‡‡‡<br><br>-0.38 (-0.73, -0.02) ‡‡‡<br><br>-0.35 (-0.71, 0.01) ‡‡‡	

The intention-to-treat population includes all participants who were randomized.
The Per-protocol population excludes 4 women due to multiple pregnancy.
* CI confidence interval, Hb hemoglobin, PP per-protocol, BMI body mass index, HIV human immunodeficiency virus, and RDT denotes rapid diagnostic test (for Plasmodium parasitaemia).
† Anemia indicates venous Hb<11.0g/dL up to and including delivery and venous Hb<12.0g/dL postpartum. Moderate/Severe anemia indicates venous Hb<10g/dL up to and including delivery and venous Hb<11.0g/dL postpartum.
‡ Iron deficient indicates serum ferritin<15ug/L or ferritin<30ug/L if C-reactive protein>5mg/L, and iron deficient anemia indicates Hb<11g/dL and serum ferritin<15 ug/L or ferritin<30 ug/L if C-reactive protein>5mg/L.
§ Total number of neonates reduced due to procedural missing data (version control).
¶ Adjusted for site, parity (primiparous vs. multiparous), gestational age at baseline (continuous), and BMI at baseline (continuous).
|| Adjusted for site, parity (primiparous vs. multiparous), gestational age at baseline (continuous), BMI at baseline (continuous), inflammation status at baseline, iron deficient status at baseline, haemoglobin at baseline (continuous), HIV positive status at baseline, and re-screened post positive malaria RDT status.
**Adjusted for site, parity (primiparous vs. multiparous), gestational age at baseline (continuous), BMI at baseline (continuous), inflammation status at baseline, iron deficient status at baseline, HIV positive status at baseline, and re-screened post positive malaria RDT status. Excluding adjusting for haemoglobin at baseline (continuous) due to collinearity with the outcome of Hb change from baseline – g/dL.
†† Adjusted for site, parity (primiparous vs. multiparous), gestational age at baseline (continuous), BMI at baseline (continuous), inflammation status at baseline, haemoglobin at baseline (continuous), and re-screened post positive malaria RDT status. Excluding adjusting for iron deficient status at baseline, due to collinearity with the outcome of Iron deficiency.
‡‡ Neonate first adjusted model: Adjusted for site, sex (female or male), gestational age at baseline (continuous), and maternal BMI at baseline (continuous).

§§ Neonatal second adjusted model: Adjusted for site, sex (female or male), gestational age at baseline (continuous), maternal BMI at baseline (continuous) and maternal

hemoglobin at baseline (continuous).
¶¶ Missing maternal haemoglobin data were multiply imputed according to treatment group using chained equations. The imputation model included site, parity (primiparous vs multiparous), gestational age at baseline, BMI at baseline, inflammation status at baseline, iron deficient status at baseline, haemoglobin at baseline, HIV positive status at baseline, and re-screened post positive malaria RDT status. A total of 45 imputations were used, as approximately 45% of the participants had missing data in haemoglobin values at any one time-point. An analysis based on a pattern-mixture model was conducted to assess sensitivity to outcome data not missing at random. A delta-adjustment to the imputed values was added before obtaining the multiple imputation estimate, confidence interval and P-value. The shifts were assumed as -2, -1.5, -1, -0.5, 0, +0.5, +1, +1.5, +2 g/dL for all imputed values in both treatment groups. A prevalence ratio of ferric carboxymaltose versus standard-of-care is displayed for maternal anemia derived from the imputed haemoglobin values following analyses using a modified Poisson model with robust standard errors and random intercept for participant.
|||| A prevalence ratio of ferric carboxymaltose versus standard-of-care is displayed following analyses using a modified Poisson model with robust standard errors and random intercept for participant.
*** A mean difference of Ferric carboxymaltose versus standard-of-care following analyses using a likelihood-based longitudinal data analysis model.
††† A geometric mean ratio is displayed for ferritin concentration after a log base e transformation due to skewness.
‡‡‡ An absolute mean difference between ferric carboxymaltose and standard-of-care is displayed following fitting a linear regression model.
§§§ A risk ratio of ferric carboxymaltose iron versus standard of care following analyses using a log-binomial regression model.
¶¶¶ The P-values and 95% confidence intervals presented have not been adjusted for multiple comparisons for the secondary and other maternal and neonatal outcomes. The intervals may not be used in place of hypothesis testing.
